# Synthesis of chiral α-amino acids *via* Pd(ii)-catalyzed enantioselective C–H arylation of α-aminoisobutyric acid†

**DOI:** 10.1039/d4sc05378h

**Published:** 2024-09-18

**Authors:** Zi-Yu Zhang, Tao Zhang, Yuxin Ouyang, Peng Lu, Jennifer X. Qiao, Jin-Quan Yu

**Affiliations:** a Department of Chemistry, The Scripps Research Institute 10550 North Torrey Pines Road, La Jolla California 92037 USA yu200@scripps.edu; b Small Molecule Drug Discovery, Bristol Myers Squibb Research and Early Development Cambridge Massachusetts 02140 USA

## Abstract

Non-natural chiral α,α-disubstituted α-amino acids (α,α-AAs) constitute an attractive α-aminoisobutyric acid (Aib) replacement for improving bioavailability of linear peptides as therapeutics due to the ability of these amino acids to induce the peptides to form helical structures. Enantioselective β-C(sp^3^)–H arylation of Aib could potentially provide a versatile one-step strategy for accessing diverse α,α-AAs, but the installation and removal of external directing groups was found in our previously reported work to reduce the efficiency of this approach. Herein we report a Pd(ii)-catalyzed enantioselective C–H arylation of *N*-phthalyl-protected Aib enabled by a *N*-2,6-difluorobenzoyl aminoethyl phenyl thioether (MPAThio) ligand, affording α,α-AAs with up to 72% yield and 98% ee. Use of this newly developed chiral catalyst has also significantly improved enantioselective C(sp^3^)–H arylation of cyclopropanecarboxylic acids by expanding the substrate scope to heterocyclic coupling partners and increasing enantioselectivity to 99% ee.

## Abstract 2

Non-natural chiral α,α-disubstituted α-amino acids (α,α-AAs) constitute an attractive α-aminoisobutyric acid (Aib) replacement for improving bioavailability of linear peptides as therapeutics due to the helical structures of the formed peptides.

## Graphical abstract

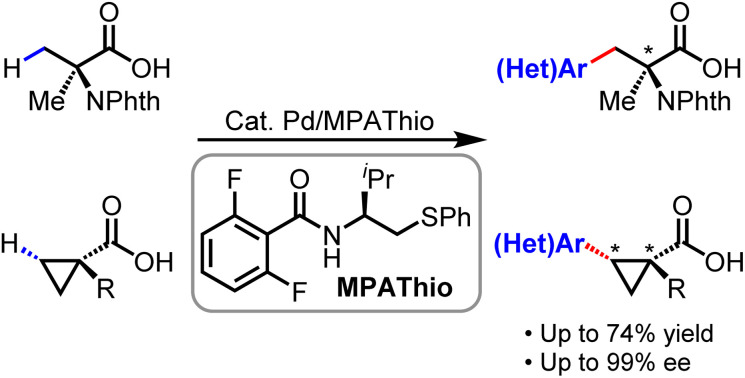

## Introduction

**Scheme 1:**
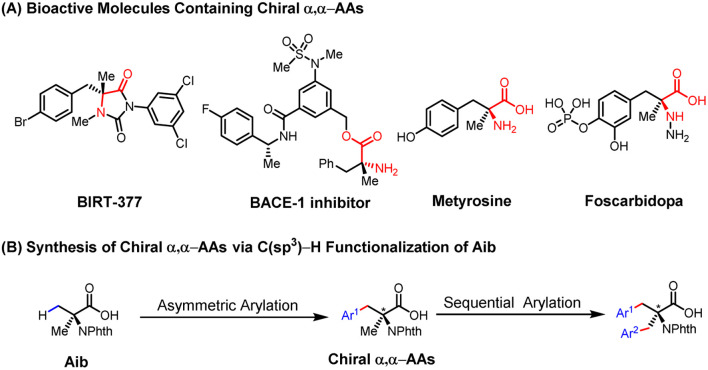
Construction of a sequential C–H activation platform for α,α-AAs.

Non-natural chiral α,α-disubstituted α-amino acids (α,α-AAs) are crucial building blocks in biochemistry and medicinal chemistry^1^ (Scheme 1A) for the assembly of peptides and proteins with restrictive conformational flexibility and high metabolic resistance.^2^ This unique property of α,α-AAs has been applied to the development of enzyme inhibitors and bioactive molecules that have received the extensive attention of industry and academia.^3^ In the area of asymmetric catalysis, various methods have been developed for the construction of chiral α,α-AAs, among which the classical Strecker reaction,^4^ Mannich reaction,^5^ alkylation of amino acids,^6^ and α-amination of carboxylic acids are the most significant developments.^7,8^ From the viewpoint of expanding chemical space in drug discovery through high-throughput synthesis, we envision that a wide range of chiral α,α-AAs can be obtained by carrying out various examples of a one-step enantioselective β-C–H functionalization of a single readily available α-aminoisobutyric acid (Aib), followed by functionalization of the remaining methyl group (Scheme 1B).

**Scheme 2:**
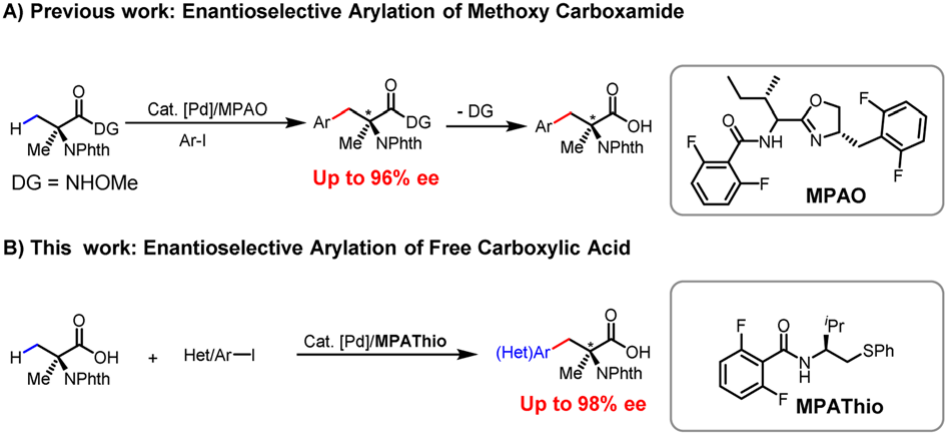
Pd(ii)-catalyzed enantioselective arylation of Aib.

Carboxylic acids constitute one of the most abundant classes of substrates in organic synthesis.^9^ The recently developed ligand-enabled β- and γ-C–H activation reactions of free acids have provided new avenues for harnessing the synthetic utility of aliphatic acids.^10^ We have envisioned that the enantioselective β-C(sp^3^)–H functionalization of Aib could serve as a versatile platform for the diversity-oriented synthesis of chiral α,α-AAs.^11^ In 2017, we reported on enantioselective arylation of *N*-phthalyl-protected Aib to access a series of chiral α,α-AAs with *N*-methoxy amide as the directing group in up to 96% ee (Scheme 2A).^12^ However, the reaction necessitates the installation and removal of a directing group that enhances the coordination with Pd to facilitate C(sp^3^)–H cleavage. These additional two steps make the application of this method in high-throughput synthesis of chiral amino acids less attractive. In our previous efforts, enantioselective arylation of the C(sp^3^)–H of *N*-phthalyl-protected Aib with various chiral ligands only yielded chiral α,α-AAs with moderate enantioselectivity (72–86% ee). Furthermore, the success of the reaction was found to be heavily contingent on the structure of the aryl iodide, resulting in limited functional group compatibility.^13^ Herein, we report a significantly improved Pd(ii)-catalyzed enantioselective β-C–H arylation of *N*-phthalyl-protected Aib, affording a wide range of chiral α,α-AAs. Also, the method could provide an effective way to prepare α,α-disubstituted cyclopropyl α-amino acids with two chiral centers (Scheme 2B). The development of a chiral *N*-2,6-difluorobenzoyl aminoethyl phenyl thioether (MPAThio) ligand was crucial for this enantioselective transformation.

## Results and discussion

We commenced our investigations with the commercially available *N*-phthalyl-protected Aib (1a) as the main substrate and methyl 4-iodobenzoate (2a) as the coupling partner for optimization studies (Table 1). No desirable products were observed in the absence of ligand, demonstrating ligand development to be essential for this reaction. The chiral bidentate *N*-acetyl aminoethyl amine (MPAAM) ligand (L1) and amino acid (MPAA) ligand (L2) developed for the enantioselective arylation of cyclopropanecarboxylic acid were found to be active for this transformation, with 20–47% yields and moderate enantioselectivities (52–72% ee). Other chiral ligands such as the *N*-acetyl amino quinoline (MPAQ) (L3), amino *O*-methylhydroxamic acid (MPAA) (L4) and amino oxazoline (MPAO) (L5) did not provide any products. To our delight, when we employed another chiral bidentate ligand, namely *N*-acetyl aminoethyl phenyl thioether (MPAThio) (L6), initially developed for the enantioselective γ-C(sp^3^)–H functionalization of cyclopropylmethylamines,^14^ the desired arylation product 3a was obtained in 56% yield with 80% ee. The ligand with a benzyl substituent on the side chain (L7) provided good stereoselectivity but a lower yield (32%). Surprisingly, replacing the acetyl group with an *N*-Boc protected group (L8) resulted in a complete loss of reactivity, showing the critical function of the acetyl group. L9 and L10 showed inefficient performances, which could be attributed to *ortho*-C–H activation of the benzoyl group of the ligands as previously observed with MPAA ligands.^15^ The *N*-2,6-difluorobenzoyl aminoethyl phenyl thioether (L11) with two *ortho*-positions blocked by fluorine atoms was found to be the optimal ligand, affording the desired product in 71% yield and 91% ee. The replacement of the isopropyl group on the side chain with other groups decreased the reactivity and enantioselectivity (L12 and L13), illustrating the influence of the steric environment.

**Table 1:** Investigation of ligands for enantioselective arylation of Aib^a^

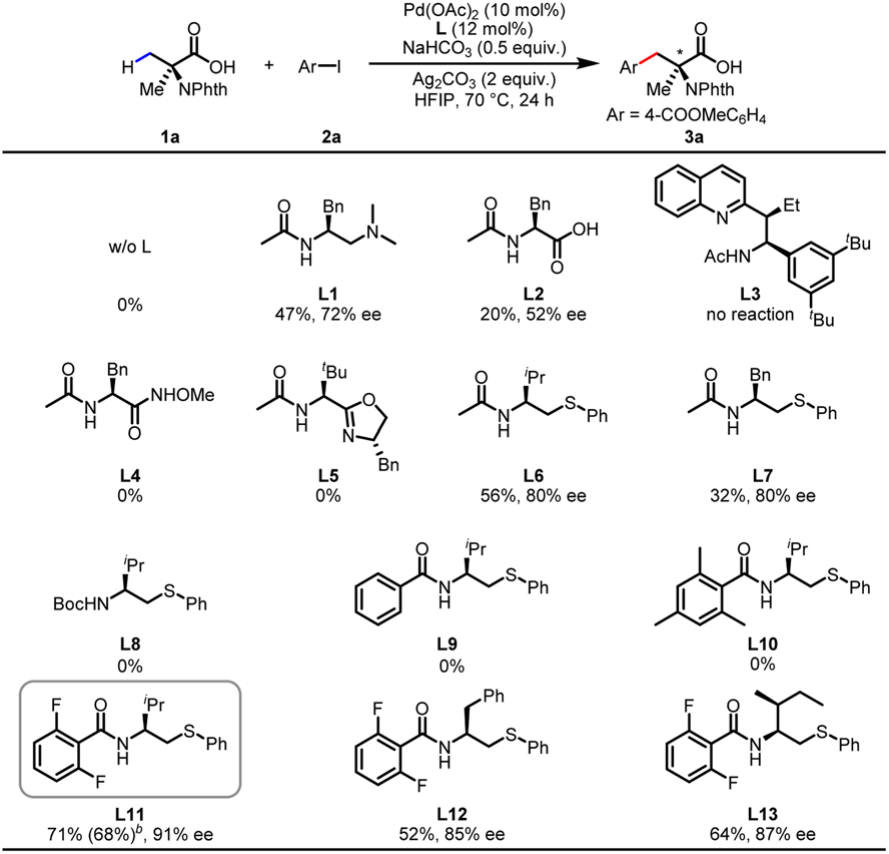

a Conditions: 1a (0.1 mmol), 2a (0.2 mmol), Pd(OAc)_2_ (10 mol%), ligand (12 mol%), Ag_2_CO_3_ (2.0 equiv.), NaHCO_3_ (0.5 equiv.) in HFIP (0.5 mL) 70 °C, 24 h. Yields were determined from the results of ^1^H NMR spectroscopy using CH_2_Br_2_ as the internal standard.

b Isolated yield.

With the optimized conditions in hand, we proceeded to survey the scope of aryl iodides using *N*-phthalyl-protected Aib (1a) as the main substrate (Table 2). Under the standard conditions, use of the simple iodobenzene (2b) resulted in a 64% yield with 88% ee. The aryl iodides bearing electron-donating substituents (2c–g) or electron-withdrawing CF_3_ (2h) and halide substituents at the *para* position (2i–k) provided the arylated products in 48–74% yields with 86–98% ee. Notably, the arylation product 3k was previously shown to be a key intermediate for the synthesis of BIRT-377, a potent LFA-1 inhibitor for the treatment of inflammatory and immune disorders.^16^ Other *para*-substituents including NO_2_ (2l), COMe (2m) and COPh (2n) were also compatible, affording 62–65% yields with 88–93% ee. The reactions with *meta*-substituted aryl iodides proceeded smoothly to give the arylated products (3o–u) with 56–68% yields and 87–92% ee. The 3,5-disubstituted aryl iodide produced the corresponding product (3v) in 63% yield with 92% ee. While heteroaryl iodides are often less effective,^17^ the thioether ligand as a donor can enhance the Pd(ii)/Pd(iv) oxidative addition process, thereby improving the reactivity,^14*a*^ also, 2-substituted iodopyridine gave excellent enantioselectivity (3w–3y) (up to 98% ee) under the optimal reaction conditions.

**Table 2:** Scope of the enantioselective arylation of Aib^a^

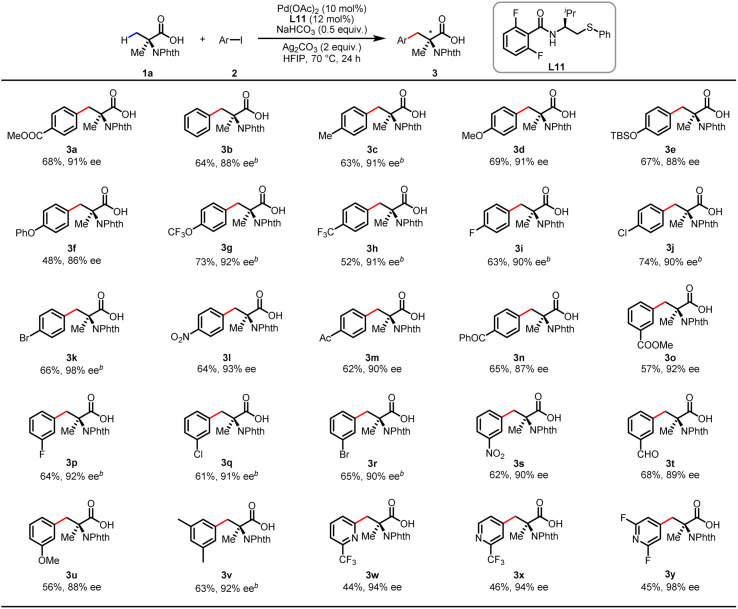

a Conditions: carboxylic acid 1 (0.1 mmol), 2 (0.2 mmol), Pd(OAc)_2_ (10 mol%), L11 (12 mol%), Ag_2_CO_3_ (2.0 equiv.), NaHCO_3_ (0.5 equiv.) in HFIP (0.5 mL), 70 °C, 24 h. Isolated yields. The ee values were determined from the results of SFC analysis on a chiral stationary phase.

b Isolated as the corresponding methyl ester.

Although our group previously reported stereoselective C(sp^3^)–H arylation of cyclopropanecarboxylic acids enabled by chiral MPAAM ligands, heterocyclic coupling partners and α-amino-substituted cyclopropanecarboxylic acid are largely incompatible.^18^ Since such incompatibility has substantially restricted applications in drug discovery, we decided to re-evaluate the enantioselective heteroarylation of cyclopropanecarboxylic acids with our newly developed MPAThio ligands (Table 3). The arylation of 4a with 2a afforded the coupling product in 47% yield and 95% ee. The *para*- or *meta*-substituted aryl iodides and even heteroaryl iodide were found to be compatible, providing the arylated products (5a–d) with moderate yields and excellent ee. The heteroarylation of α-*H*-substituted cyclopropanecarboxylic acids with 5-iodo-2-(trifluoromethyl)pyridine provided the desired chiral product (5e) in 68% yield and 98% ee under the optimized conditions. The position of the iodo substituent on the pyridine was found to impact the reactivity and enantioselectivity. In general, the iodo substituent at the C3 position gave higher ee than it did at the C2 and C4 positions (5f–g). Pyridines bearing fluoro, chloro and bromo groups at the C2 position were all found to be compatible, affording excellent enantioselectivity levels (5h, 5i) (97 and 98% ee). The α-phenyl-substituted cyclopropanecarboxylic acid also reacted successfully, providing a moderate product yield (5k) (62%) with high enantioselectivity (97% ee). Although the desired arylated product was obtained for the α-amino-substituted cyclobutanecarboxylic acid (4l), the yield (24%) and ee (48%) were significantly reduced.

**Table 3:** Scope of the enantioselective (hetero)arylation of cyclopropanecarboxylic acids^a^

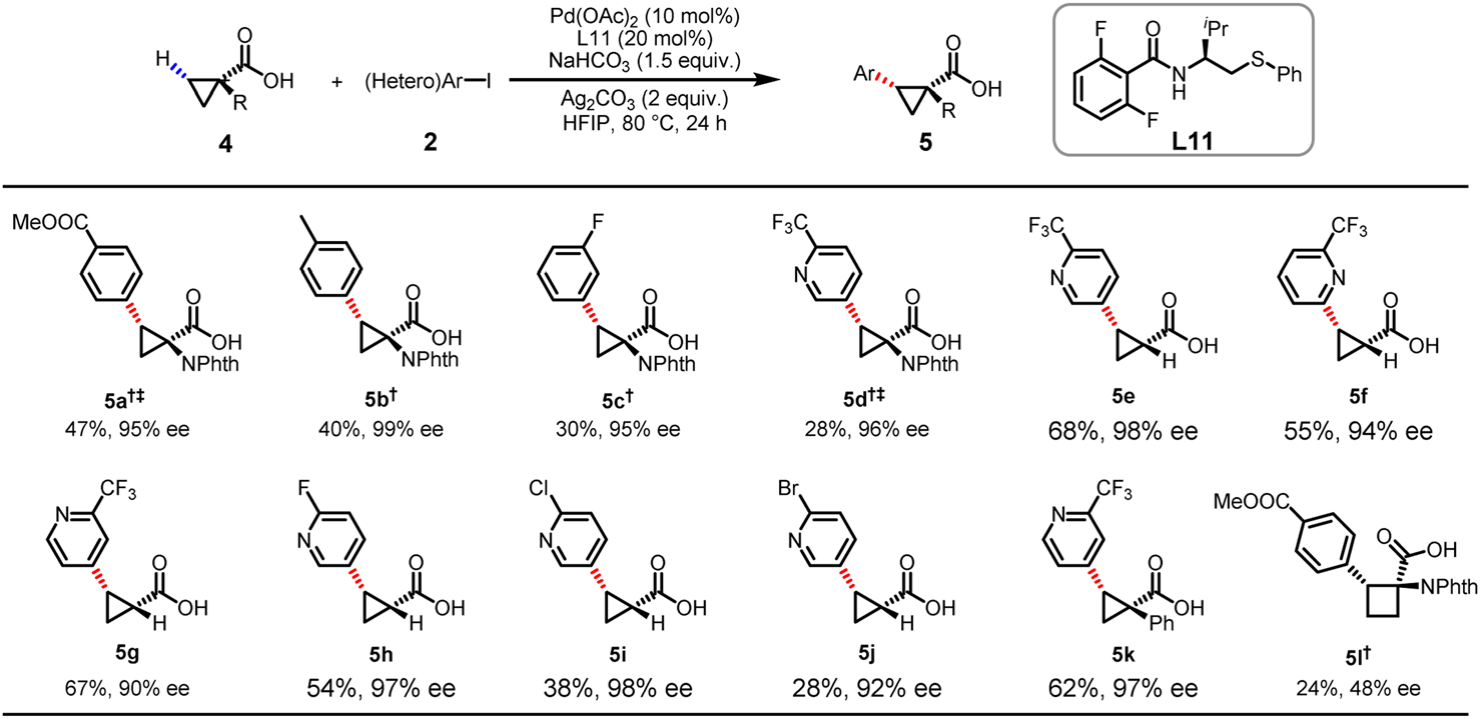

a Conditions: carboxylic acid 4 (0.1 mmol), 2 (0.2 mmol), Pd(OAc)_2_ (10 mol%), L11 (12 mol%), Ag_2_CO_3_ (2.0 equiv.), NaHCO_3_ (1.5 equiv.) in HFIP (1.0 mL), 80 °C, 24 h. Isolated yields. The ee values were determined from the results of SFC analysis on a chiral stationary phase. ^†^ Isolated as the corresponding methyl ester. ^‡^ K_2_CO_3_ (1.0 equiv.) was used and the reaction was carried out a temperature of 100 °C.

The enantioselective β-C–H arylation of Aib followed by sequential C–H arylation of the remaining methyl group could lead to a diverse range of chiral α,α-AAs. The diarylation product of 6 was obtained with 65% yield using the MPAA ligand (Table 4A). We also applied this enantioselective arylation reaction to the syntheses of drug molecules. As a therapeutic agent for hypertension, metyrosine has been employed to alleviate blood pressure elevation in individuals afflicted with adrenal tumors.^19^ In the current work, the synthesis of metyrosine was achieved through a two-step process from 3d, with this process involving demethylation utilizing BBr_3_ in anhydrous dichloromethane followed by acidic hydrolysis to remove the *N*-phthalimide group (68% in two steps) (Table 4B). The methyl ester of 3k was also successfully deprotected to give chiral amino acid 9, a key intermediate for the synthesis of BIRT-377 (Table 4C).^16^ Given the importance and common use of Boc-protected amino acids in peptide chemistry, we set out to synthesize the Boc-protected α,α-AAs (10) using free amine (9) under mild conditions, and did so successfully (Table 4D).

**Table 4:** Practical applications

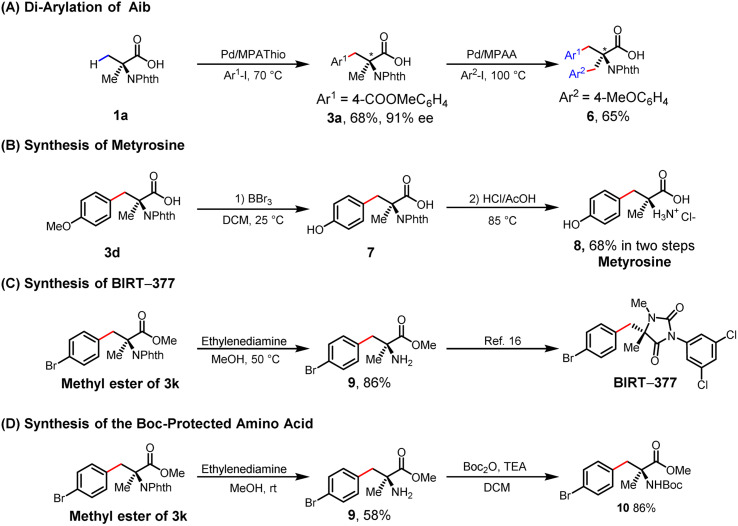

## Conclusions

In summary, we have developed Pd(ii)-catalyzed enantioselective β-C–H arylation of *N*-phthalyl-protected Aib using the newly developed *N*-2,6-difluorobenzoyl aminoethyl phenyl thioether (MPAThio) ligand L11. Various aryl iodides and C2-substituted pyridyl iodides were tolerated, affording chiral non-natural α,α-AAs in 44–74% yields with 86–98% ee. The *N*-phthalyl group of the product can be removed under mild conditions for synthetic applications as demonstrated in the synthesis of metyrosine. Notably, use of this newly developed chiral MPAThio ligand has also substantially improved the scope and enantioselectivity of β-C(sp^3^)–H arylation of cyclopropanecarboxylic acids.

## Author contributions

Z.-Y. Z. and T. Z. discovered and developed the reactions. Y. O. and P. L. performed a portion of the synthetic experiments. J. X. Q. participated in substrate scope surveys and discussions. J.-Q. Y. directed the project. Z.-Y. Z. and T. Z. wrote the ESI.†

## Conflicts of interest

The authors declare no competing financial interests.

## Supplementary Material

SC-015-D4SC05378H-s001

## Data Availability

All the data have been presented in the manuscript and ESI.†
