# Supplementary material for: Synthesis of chiral α-amino acids via Pd(ii)-catalyzed enantioselective C–H arylation of α-aminoisobutyric acid
Source: Chem Sci. 2024 Sep 18;15(41):17092–6. doi: 10.1039/d4sc05378h (PMC11427991; doi:10.1039/d4sc05378h)
Supplement: SC-015-D4SC05378H-s001 [file SC-015-D4SC05378H-s001.pdf]

Supplementary Information (SI) for Chemical Science.
This journal is © The Royal Society of Chemistry 2024

Supporting Information

Synthesis of Chiral *α*-Amino Acids via Pd(II)-Catalyzed Enantioselective C–H Arylation of *α*-Aminoisobutyric Acid

Zi-Yu Zhang,[1†] Tao Zhang,[1†] Yuxin Ouyang,[1] Peng Lu,[1] Jennifer X. Qiao[2] and Jin-Quan Yu[1]*

[1]The Scripps Research Institute, 10550 N. Torrey Pines Road, La Jolla, California 92037, USA.

[2]Small Molecule Drug Discovery, Bristol Myers Squibb Research and Early Development, Cambridge, Massachusetts 02140, USA.

[†]These authors contributed equally to this work.

*yu200@scripps.edu

Table of Contents

1. General Information..3

2. General procedure of the preparation of thioether ligands 3

3. Ligands investigation..7

4. Enantioselective Arylation of 2-Aminoisobutyric Acid...8

5. Enantioselective Arylation of Cylcopropanecarboxylic acid...................................42

6. Sequential diarylation of 2-Aminoisobutyric Acid..62

7. Synthesis of Metyrosine..63

8. Synthesis of BIRT-377...64

9. Synthesis of Boc-amino acid 64

10. Reference 65

11. NMR...66

1. General Information

Commercially available reagents were purchased at the highest commercial quality and used without further purification, unless otherwise stated. Analytical thin layer chromatography was performed on 0.25 mm silica gel 60-F254. ^{1}H NMR spectra were recorded on Bruker AMX-400 or Bruker DRX-600 instruments. The following abbreviations (or combinations thereof) were used to explain multiplicities: s = singlet, d = doublet, t = triplet, q = quartet, m = multiplet, br = broad. Coupling constants, J, were reported in Hertz unit (Hz). ^{13}C NMR spectra were recorded on Bruker DRX-600 or JEOL instruments (100 MHz) and were fully decoupled by broad band proton decoupling. ^{19}F NMR Spectra were recorded on Bruker AMX-399 spectrometer (376 MHz) or JEOL-400 (376 MHz) and were fully decoupled by broad band proton decoupling. Chemical shifts were referenced to the appropriate residual solvent peaks. Column chromatography was carried out automated using Biotage Isolera One with Biotage SNAP Ultra Column.

2. General procedure of the preparation of thioether ligands[1]

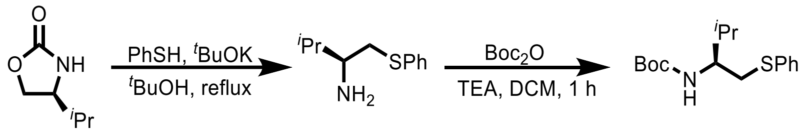

To the ^{t}BuOH (10.0 mL) solution of ^{t}BuOK (1.23 g, 11.0 mmol) was added thiophenol (1.54 mL, 15.0 mmol), and the mixture was stirred at room temperature for 10 min. Then Evans oxazolidone chiral auxiliary (10.0 mmol) in ^{t}BuOH (5 mL) was added and the mixture was stirred under 80 °C for 12 h. After being allowed to cool to room temperature, the reaction was quenched by sat. NH_4Cl (5 mL). The aqueous phase was extracted with EA (15 mL×3) and the combined organic phase was dried with anhydrous Na_2SO_4, concentrated under reduced pressure. The residue was used without purification.

To a stirred solution of substrate in dry DCM (30 mL) was added Boc_2O (2.6 g, 12 mmol) and TEA (2.0 g, 20 mmol) at 0 °C. After stirring at room temperature for 1 h, then the mixture was concentrated in vacuo, diluted with ethyl acetate, and washed

with sat. NH_4Cl and $NaHCO_3$. The organic layer was dried over $MgSO_4$, concentrated in vacuo, and purified by column with ethyl acetate/*n*-hexane (1/10) as the eluent to afford thioether ligand **L8**.

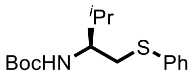

***tert*-butyl (*S*)-(3-methyl-1-(phenylthio)butan-2-yl)carbamate (L8)**

White solid. ^{1}H NMR (600 MHz, $CDCl_3$) δ 7.45 – 7.35 (m, 2H), 7.32 – 7.27 (m, 2H), 7.21 (t, *J* = 7.4 Hz, 1H), 4.59 (d, *J* = 7.8 Hz, 1H), 3.74 – 3.73 (m, 1H), 3.10 (d, *J* = 5.4 Hz, 2H), 2.04 – 1.85 (m, 1H), 1.45 (s, 9H), 0.94 (dd, *J* = 14.8, 6.8 Hz, 6H). ^{13}C NMR (150 MHz, $CDCl_3$) δ 155.75, 136.60, 129.82, 129.06, 126.33, 79.30, 55.30, 37.67, 30.95, 28.49, 19.57, 17.98. HRMS (ESI-TOF) Calcd for $C_{16}H_{24}NO_2S$ $[M-H]^-$: 294.1528; found: 294.1523.

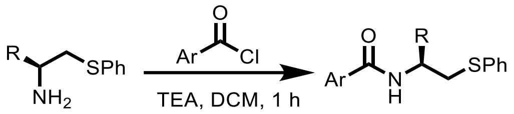

To a stirred solution of substrate (10 mmol) in dry DCM (30 mL) was added benzoyl chloride (12 mmol) and TEA (2.0 g, 20 mmol) at 0 °C. After stirring at room temperature for 1 h, then the mixture was concentrated in vacuo, diluted with ethyl acetate, and washed with sat. NH_4Cl and $NaHCO_3$. The organic layer was dried over $MgSO_4$, concentrated in vacuo and purified by column with ethyl acetate/*n*-hexane (1/10) as the eluent to afford thioether ligand.

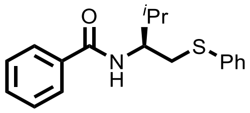

(*S*)-*N*-(3-methyl-1-(phenylthio)butan-2-yl)benzamide (L9)

White solid. ^{1}H NMR (600 MHz, $CDCl_3$) δ 7.68 – 7.62 (m, 2H), 7.53 – 7.48 (m, 1H), 7.45 – 7.39 (m, 4H), 7.28 (d, *J* = 6.2 Hz, 2H), 7.21 – 7.16 (m, 1H), 6.15 (d, *J* = 8.7 Hz, 1H), 4.25 (dq, *J* = 9.2, 5.6 Hz, 1H), 3.27 (d, *J* = 5.4 Hz, 2H), 2.12 (dq, *J* = 13.6, 6.8 Hz, 1H), 1.02 (t, *J* = 6.8 Hz, 6H). ^{13}C NMR (150 MHz, $CDCl_3$) δ 167.33, 136.27, 134.77, 131.51, 129.89, 129.23, 128.62, 126.94, 126.52, 54.60, 37.18, 30.89, 19.58, 18.60. HRMS (ESI-TOF) Calcd for $C_{18}H_{20}NOS$ $[M-H]^-$: 298.1266; found: 298.1253.

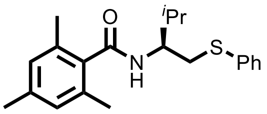

(*S*)-mesityl((3-methyl-1-(phenylthio)butan-2-yl)-l2-azaneyl)methanone (L10)

White solid. ^{1}H NMR (600 MHz, $CDCl_3$) δ 7.42 (d, *J* = 7.6 Hz, 2H), 7.32 (t, *J* = 7.8 Hz, 2H), 7.22 (t, *J* = 7.4 Hz, 1H), 6.87 (s, 2H), 5.76 (d, *J* = 8.7 Hz, 1H), 4.28 (dq, *J* = 11.9, 5.9 Hz, 1H), 3.22 (d, *J* = 5.7 Hz, 2H), 2.34 (s, 6H), 2.30 (s, 3H), 2.15 – 2.05 (m, 1H), 1.01 (dd, *J* = 13.1, 6.8 Hz, 6H). ^{13}C NMR (100 MHz, $CDCl_3$) δ 170.51, 138.56, 136.16, 135.15, 134.36, 129.79, 129.16, 128.32, 126.49, 53.86, 37.15, 30.39, 21.17, 19.46, 19.41, 18.38. HRMS (ESI-TOF) Calcd for $C_{21}H_{26}NOS$ $[M-H]^-$: 340.1735; found: 340.1722.

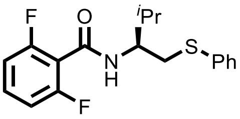

(*S*)-2,6-difluoro-*N*-(3-methyl-1-(phenylthio)butan-2-yl)benzamide (L11)

White solid. ^{1}H NMR (400 MHz, $CDCl_3$) δ 7.47 – 7.40 (m, 2H), 7.40 – 7.33 (m, 1H), 7.32 – 7.25 (m, 2H), 7.21 – 7.14 (m, 1H), 7.01 – 6.91 (m, 2H), 5.99 (d, *J* = 8.4 Hz, 1H) 4.25 (dq, *J* = 9.4, 5.8 Hz, 1H), 3.29 – 3.15 (m, 2H), 2.09 (h, *J* = 6.8 Hz, 1H), 1.02 (dd, *J* = 6.8, 5.4 Hz, 6H). ^{13}C NMR (100 MHz, $CDCl_3$) δ 160.27, 160.04 (dd, *J* = 275.3, 6.8 Hz), 136.0, 131.69 (t, *J* = 10.1 Hz), 130.13, 129.15, 126.60, 112.09 (d, *J* = 25.0 Hz), 112.09 (dd, *J* = 18.2, 3.0 Hz), 54.59, 37.22, 30.53, 19.54, 18.05. ^{19}F NMR (376 MHz, $CDCl_3$) δ -114.58. HRMS (ESI-TOF) Calcd for $C_{18}H_{18}F_2NOS$ $[M-H]^-$: 334.1077; found: 334.1065.

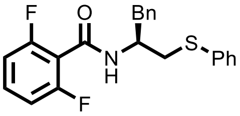

(*S*)-2,6-difluoro-*N*-(1-phenyl-3-(phenylthio)propan-2-yl)benzamide (L12)

White solid. ^{1}H NMR (400 MHz, $CDCl_3$) δ 7.46 – 7.39 (m, 2H), 7.39 – 7.17 (m, 9H), 6.94 (t, *J* = 8.1 Hz, 2H), 6.12 (d, *J* = 7.6 Hz, 1H), 4.61 (h, *J* = 6.2 Hz, 1H), 3.24 – 3.03 (m, 4H). ^{13}C NMR NMR (100 MHz, $CDCl_3$) δ 160.10 (dd, *J* = 252.4, 7.3 Hz), 160.08, 136.94, 135.49, 131.82 (t, *J* = 10.0 Hz), 129.95, 129.57, 129.23, 128.73, 126.90, 126.64, 112.12 (dd, *J* = 17.9, 2.8 Hz), 112.11 (d, *J* = 24.8 Hz), 50.68, 38.76, 37.23. ^{19}F NMR (376 MHz, $CDCl_3$) δ -114.46. HRMS (ESI-TOF) Calcd for $C_{22}H_{18}F_2NOS$ [M-

H]$^-$: 382.1077; found: 382.1062.

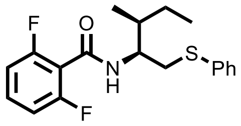

2,6-difluoro-*N*-((2*S*,3*S*)-3-methyl-1-(phenylthio)pentan-2-yl)benzamide (L13)

White solid. ^{1}H NMR (400 MHz, $CDCl_3$) δ 7.46 – 7.40 (m, 2H), 7.39 – 7.33 (m, 1H), 7.31 – 7.25 (m, 2H), 7.22 – 7.15 (m, 1H), 7.02 – 6.90 (m, 2H), 6.02 (d, *J* = 8.6 Hz, 1H), 4.38 – 4.25 (m, 1H), 3.31 – 3.13 (m, 2H), 1.87 (ddt, J = 13.4, 6.8, 3.6 Hz, 1H), 1.59 (dtt, *J* = 15.0, 7.4, 3.8 Hz, 1H), 1.25 – 1.10 (m, 1H), 0.99 (d, *J* = 6.8 Hz, 3H), 0.94 (t, *J* = 7.4 Hz, 3H). ^{13}C NMR (100 MHz, $CDCl_3$) δ 160.28, 160.08 (dd, *J* = 252.0, 6.2 Hz), 135.92, 131.72 (t, *J* = 8.8 Hz), 130.27, 129.15, 126.65, 112.10 (dd, *J* = 15.7, 1.8 Hz), 112.08 (d, *J* = 23.8 Hz), 53.63, 36.98, 36.89, 24.95, 15.48, 11.44. ^{19}F NMR (376 MHz, $CDCl_3$) δ -114.56. HRMS (ESI-TOF) Calcd for $C_{19}H_{20}F_2NOS$ [M-H]$^-$:348.1234; found: 348.1223.

3. Ligands investigation

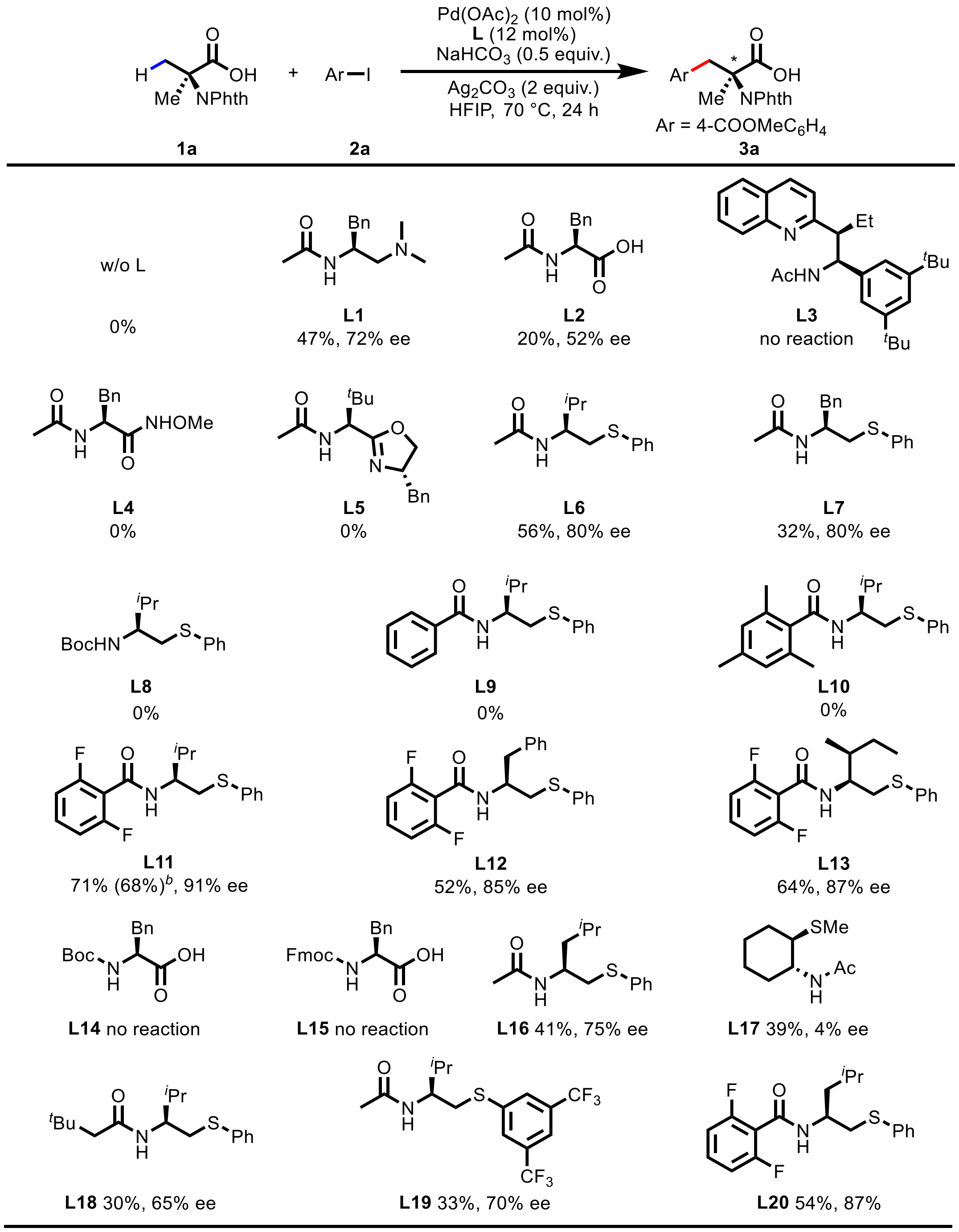

[a]Conditions: **1a** (0.1 mmol), **2a** (0.2 mmol), $Pd(OAc)_2$ (10 mol%), ligand (12 mol%), Ag_2CO_3 (2.0 equiv.), $NaHCO_3$ (0.5 equiv.) in HFIP (0.5 mL) 70 °C, 24 h. Yields were determined by ^{1}H NMR using CH_2Br_2 as the internal standard. [b]Isolated yield

4. Enantioselective Arylation of 2-Aminoisobutyric Acid[2]

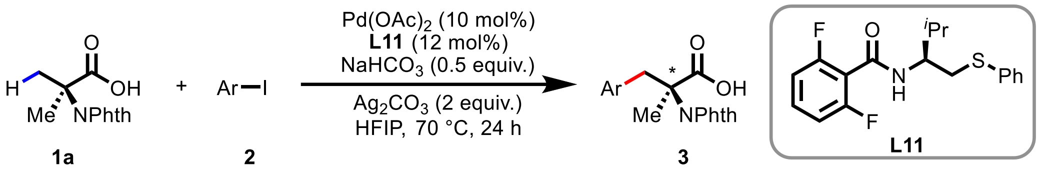

General procedure A for enantioselective arylation of 2-Aminoisobutyric Acid: $Pd(OAc)_2$ (2.2 mg, 0.01 mmol, 10 mol%), **L11** (5.0 mg, 0.015 mmol, 15 mol%), **1a** (0.1 mmol), Ag_2CO_3 (55 mg, 0.2 mmol, 2 equiv.), $NaHCO_3$ (4.2 mg, 0.05 mmol, 0.5 equiv.), and ArI (0.2 mmol, 2.0 equiv.) were weighed and placed in an 12 mL reaction tube. Subsequently, HFIP (0.5 mL) was injected, and the tube was capped and closed tightly. The reaction mixture was then stirred at 70 °C for 48 h. The mixture was allowed to cool to room temperature and acetic acid (0.05 ml) was added. Then, the mixture was passed through a pad of Celite with ethyl acetate as the eluent to remove any insoluble precipitate. The resulting solution was concentrated.

For the compound isolated as an acid: The residual mixture was dissolved with minimal ethyl acetate and loaded onto a preparative TLC plate. The pure acid product was then isolated using preparative TLC with ethyl acetate/hexanes (2/1) with 1% w/w acetic acid as the eluent.

For the compound isolated as an ester: The residual mixture was dissolved in 0.5 ml DMF, and Cs_2CO_3 (99.7 mg, 0.3 mmol), MeI (71.0 mg, 0.50 mmol, 31 μL) were added. The mixture was stirred at room temperature for 3 h and then was diluted with water followed by extraction with ethyl acetate. The organic layer was dried over Na_2SO_4, filtered, and concentrated in vacuo. The residual mixture was dissolved with minimal ethyl acetate and loaded onto a preparative TLC plate. The pure ester product was then isolated using preparative TLC with ethyl acetate/toluene (1/20) as the eluent.

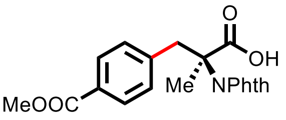

(*S*)-2-(1,3-dioxoisoindolin-2-yl)-3-(4-(methoxycarbonyl)phenyl)-2-methyl propanoic acid (3a)[2]

Following the general arylation procedure A (eluent: hexane/ethyl acetate = 2/1 with 1% v/v of acetic acid). The product of **3a** as acid was obtained (white solid, 25.1 mg, 68% yield).

The enantiomeric purity of the substrate was determined by SFC analysis (CHIRALPAK® OJ-3 column, 15% ^{i}PrOH / CO_2, flow rate 2 mL/min, retention time 7.373 min (minor) and 9.524 min (major), 4.5:95.5 er).

^{1}H NMR (600 MHz, $CDCl_3$) δ 8.90 (s, 1H), 7.85 (d, J = 8.0 Hz, 2H), 7.81 – 7.75 (m, 2H), 7.75 – 7.67 (m, 2H), 7.14 (d, J = 8.0 Hz, 2H), 3.92 – 3.79 (m, 4H), 3.27 (d, J = 13.8 Hz, 1H), 1.95 (s, 3H). ^{13}C NMR (150 MHz, $CDCl_3$) δ 177.78, 168.40, 167.05, 141.02, 134.40, 131.39, 130.62, 129.68, 129.08, 123.51, 63.67, 52.16, 40.96, 21.88. HRMS (ESI-TOF) Calcd for $C_{20}H_{16}NO_6$ $[M-H]^-$: 366.0978; found: 366.0975.

The absolute stereochemistry was assigned based on comparing the specific rotation of **3k** with literature.

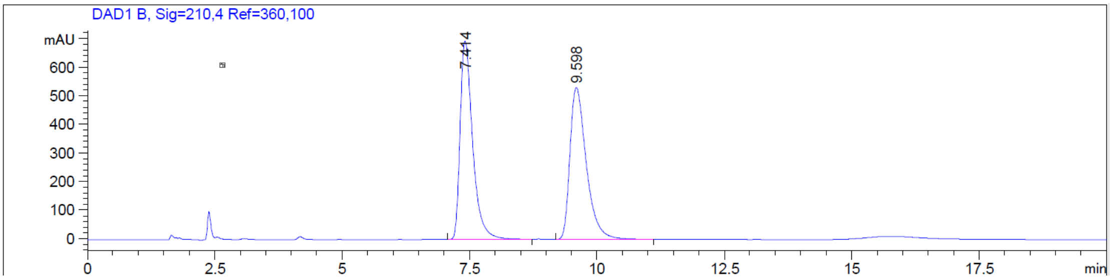

Peak #	RetTime [min]	Type	Width [min]	Area [mAU*s]	Height [mAU]	Area %
1	7.414	BB	0.2588	1.18170e4	693.64398	49.8909
2	9.598	BB	0.3407	1.18687e4	531.17914	50.1091

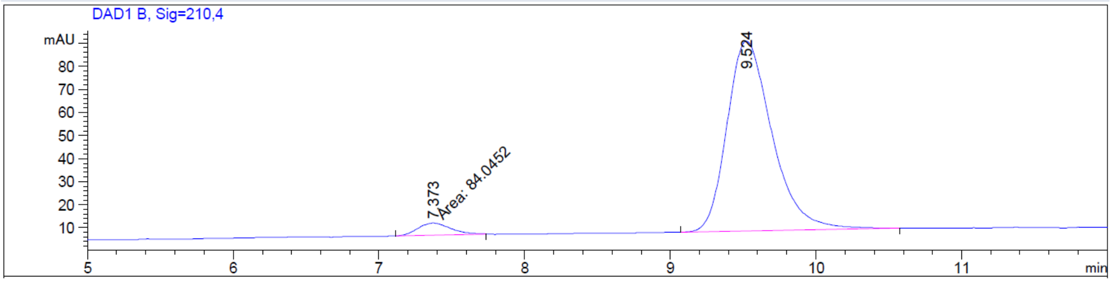

Peak #	RetTime [min]	Type	Width [min]	Area [mAU*s]	Height [mAU]	Area %
1	7.373	MM	0.2640	84.04516	5.30673	4.5170
2	9.524	BB	0.3276	1776.58533	82.38560	95.4830

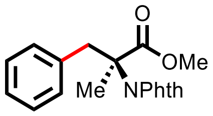

Methyl (*S*)-2-(1,3-dioxoisoindolin-2-yl)-2-methyl-3-phenylpropanoate (3b)[2]

Following the general arylation procedure A (eluent: toluene/ethyl acetate = 20/1). The product of **3b** as ester was obtained (yellow oil, 20.7 mg, 64% yield).

The enantiomeric purity of the substrate was determined by SFC analysis (CHIRALPAK® OJ-3 column, 10% ^{i}PrOH / CO_2, flow rate 2 mL/min, retention time 4.406 min (minor) and 9.467 min (major), 6:94 er). HRMS (ESI-TOF) Calcd for $C_{19}H_{16}NO_4$ $[M-H]^-$: 322.1079; found: 322.1070.

^{1}H NMR (600 MHz, $CDCl_3$) δ 7.80 – 7.74 (m, 2H), 7.73 – 7.68 (m, 2H), 7.21 – 7.14 (m, 3H), 7.04 (d, J = 6.8 Hz, 2H), 3.82 – 3.70 (m, 4H), 3.26 (d, J = 13.8 Hz, 1H), 1.89 (s, 3H). ^{13}C NMR (150 MHz, $CDCl_3$) δ 173.01, 168.48, 135.74, 134.19, 131.64, 130.58, 128.29, 127.17, 123.30, 64.14, 52.77, 41.36, 21.92.

The absolute stereochemistry was assigned based on comparing the specific rotation of **3k** with literature.

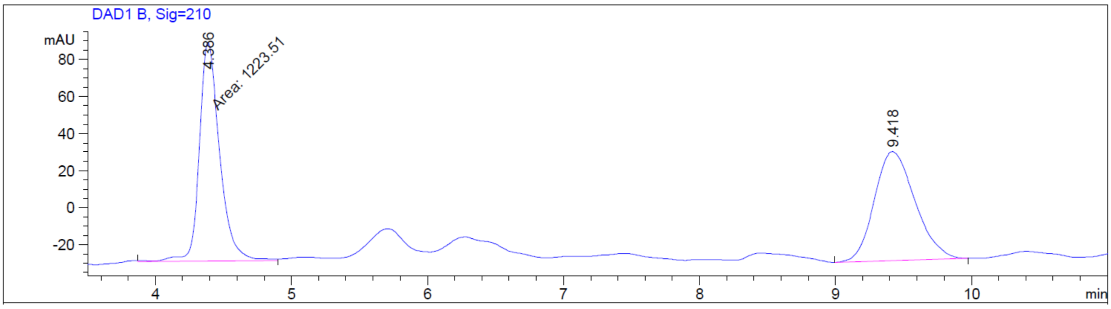

Peak #	RetTime [min]	Type	Width [min]	Area [mAU*s]	Height [mAU]	Area %
1	4.386	MM	0.1725	1223.51050	118.20831	50.0102
2	9.418	BB	0.3203	1223.00989	58.88267	49.9898

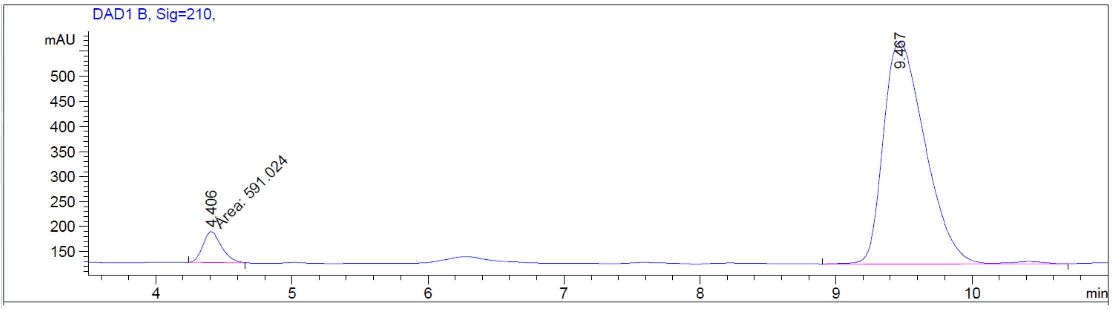

Peak #	RetTime [min]	Type	Width [min]	Area [mAU*s]	Height [mAU]	Area %
1	4.406	MM	0.1596	591.02374	61.72735	5.8810
2	9.467	BV R	0.3319	9458.64551	441.69794	94.1190

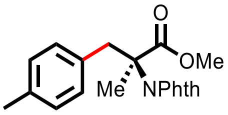

Methyl (*S*)-2-(1,3-dioxoisoindolin-2-yl)-2-methyl-3-(p-tolyl)propanoate (3c)[2]

Following the general arylation procedure A (eluent: toluene/ethyl acetate = 20/1). The product of **3c** as ester was obtained (yellow oil, 21.3 mg, 63% yield).

The enantiomeric purity of the substrate was determined by SFC analysis (CHIRALPAK® OJ-3 column, 10% ^{i}PrOH / CO_2, flow rate 2 mL/min, retention time 4.244 min (minor) and 7.846 min (major), 4.5: 95.5 er).

^{1}H NMR (600 MHz, $CDCl_3$) δ 7.82 – 7.77 (m, 2H), 7.76 – 7.70 (m, 2H), 6.99 (d, *J* = 7.9 Hz, 2H), 6.95 (d, *J* = 8.0 Hz, 2H), 3.77 (s, 3H), 3.77 (d, *J* = 13.8 Hz, 1H), 3.24 (d, *J* = 13.8 Hz, 1H), 2.28 (s, 3H), 1.89 (s, 3H). ^{13}C NMR (150 MHz, $CDCl_3$) δ 173.05, 168.52, 136.68, 134.17, 132.55, 131.69, 130.42, 129.04, 123.30, 64.24, 52.73, 40.93, 21.85, 21.17. HRMS (ESI-TOF) Calcd for $C_{20}H_{18}NO_4$ $[M+H]^+$: 338.1392; found: 338.1385.

The absolute stereochemistry was assigned based on comparing the specific rotation of **3k** with literature.

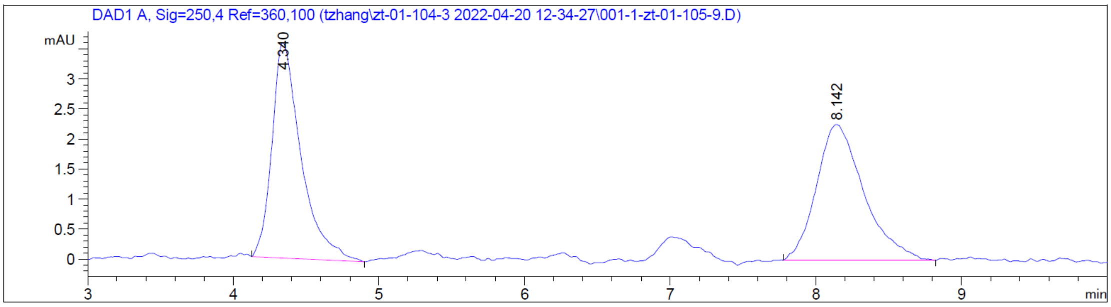

Peak #	RetTime [min]	Type	Width [min]	Area [mAU*s]	Height [mAU]	Area %
1	4.340	BB	0.2022	49.77608	3.57928	49.3677
2	8.142	BB	0.2798	51.05114	2.25771	50.6323

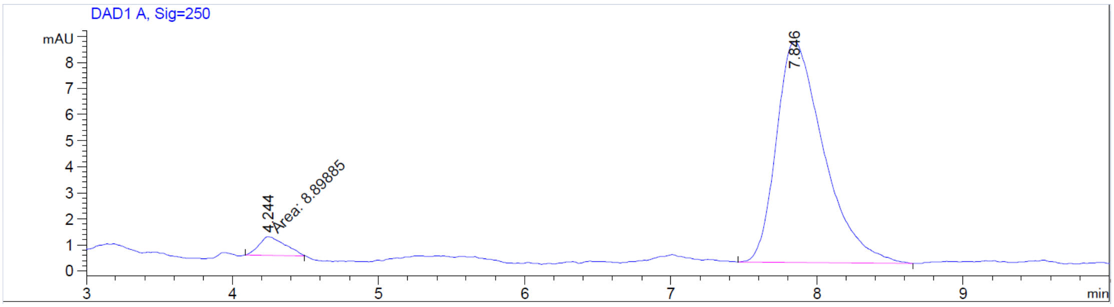

Peak #	RetTime [min]	Type	Width [min]	Area [mAU*s]	Height [mAU]	Area %
1	4.244	MM	0.2064	8.89885	7.18632e-1	4.4542
2	7.846	BB	0.3291	190.88802	8.46409	95.5458

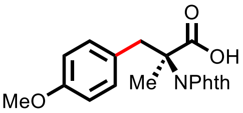

(*S*)-2-(1,3-dioxoisoindolin-2-yl)-3-(4-methoxyphenyl)-2-methylpropanoic acid (3d)[2]

Following the general arylation procedure A (eluent: hexane/ethyl acetate = 2/1 with 1% v/v of acetic acid). The product of **3d** as acid was obtained (white solid, 23.3 mg, 69% yield).

The enantiomeric purity of the substrate was determined by SFC analysis (CHIRALPAK® IC-3 column, 15% ^{i}PrOH / CO_2, flow rate 2 mL/min, retention time 7.373 min (minor) and 9.524 min (major), 4.5:95.5 er).

^{1}H NMR (600 MHz, $CDCl_3$) δ 7.84 – 7.75 (m, 2H), 7.73 – 7.66 (m, 2H), 6.97 (d, *J* = 8.2 Hz, 2H), 6.71 (d, *J* = 8.2 Hz, 2H), 3.78 – 3.64 (m, 4H), 3.17 (d, *J* = 14.1 Hz, 1H), 1.95 (s, 3H). ^{13}C NMR (150 MHz, $CDCl_3$) δ 177.94, 168.47, 158.77, 134.24, 131.59, 131.52, 127.45, 123.38, 113.83, 64.05, 55.23, 40.08, 21.84. HRMS (ESI-TOF) Calcd for $C_{19}H_{16}NO_5$ $[M-H]^-$: 338.1028; found: 338.1028.

The absolute stereochemistry was assigned based on comparing the specific rotation of **3k** with literature.

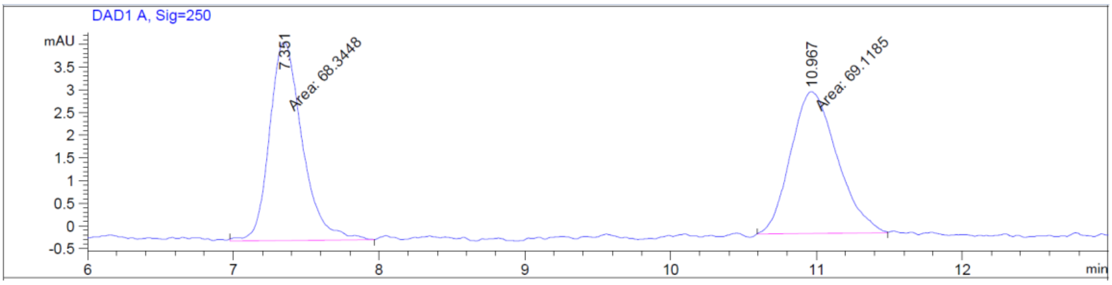

Peak #	RetTime [min]	Type	Width [min]	Area [mAU*s]	Height [mAU]	Area %
1	7.351	MM	0.2609	68.34481	4.36608	49.7186
2	10.967	MM	0.3690	69.11846	3.12206	50.2814

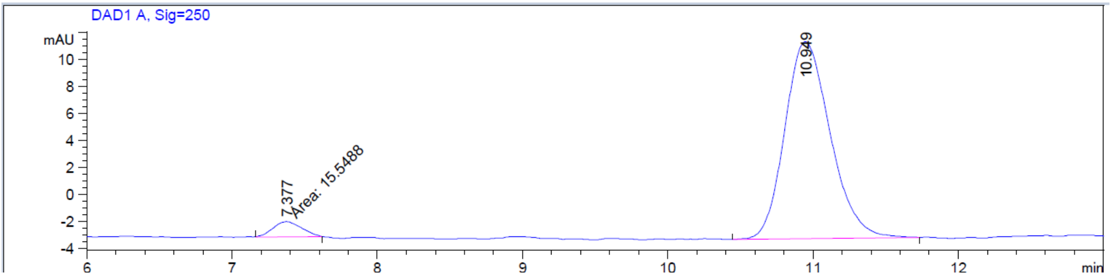

Peak #	RetTime [min]	Type	Width [min]	Area [mAU*s]	Height [mAU]	Area %
1	7.377	MM	0.2278	15.54884	1.13740	4.6601
2	10.949	BB	0.3337	318.10883	14.62985	95.3399

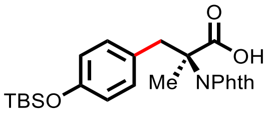

(*S*)-3-(4-((tert-butyldimethylsilyl)oxy)phenyl)-2-(1,3-dioxoisoindolin-2-yl)-2 methyl propanoic acid (3e)[2]

Following the general arylation procedure A (eluent: hexane/ethyl acetate = 2/1 with 1% v/v of acetic acid). The product of **3d** as acid was obtained (white solid, 29.4 mg, 67% yield).

The enantiomeric purity of the substrate was determined by SFC analysis (CHIRALPAK® IC-3 column, 15% ^{i}PrOH / CO_2, flow rate 2 mL/min, retention time 9.533 min (minor) and 10.648 min (major), 6:94 er).

^{1}H NMR (600 MHz, $CDCl_3$) δ 7.79 – 7.13 (m, 2H), 7.72 – 7.66 (m, 2H), 6.89 (d, *J* = 8.3 Hz, 2H), 6.62 (d, *J* = 8.4 Hz, 2H), 3.71 (d, *J* = 14.2 Hz, 1H), 3.12 (d, *J* = 14.2 Hz, 1H), 1.95 (s, 3H), 0.93 (s, 9H), 0.10 (s, 6H). ^{13}C NMR (150 MHz, $CDCl_3$) δ 171.06, 161.40, 147.86, 127.15, 124.59, 124.46, 121.34, 116.31, 113.11, 57.02, 33.14, 18.78, 14.96, 11.30, -11.41. HRMS (ESI-TOF) Calcd for $C_{24}H_{28}NO_5Si$ $[M-H]^-$: 438.1737; found: 438.1737.

The absolute stereochemistry was assigned based on comparing the specific rotation of **3k** with literature.

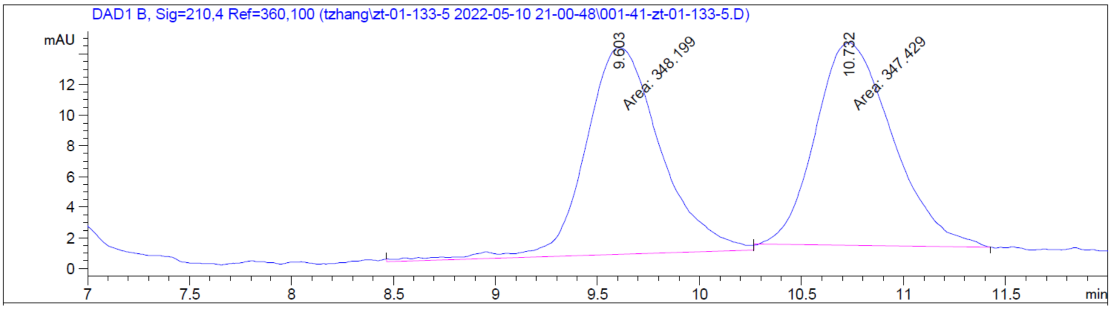

Peak #	RetTime [min]	Type	Width [min]	Area [mAU*s]	Height [mAU]	Area %
1	9.603	MM	0.4303	348.19855	13.48821	50.0553
2	10.732	MM	0.4376	347.42886	13.23219	49.9447

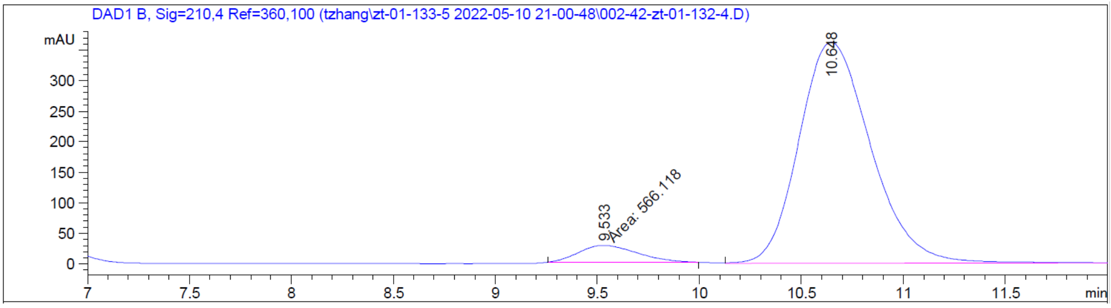

Peak #	RetTime [min]	Type	Width [min]	Area [mAU*s]	Height [mAU]	Area %
1	9.533	MM	0.3391	566.11829	27.82391	5.9982
2	10.648	VB	0.3802	8871.97168	361.68579	94.0018

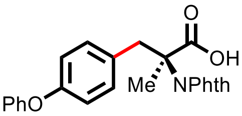

(*S*)-2-(1,3-dioxoisoindolin-2-yl)-2-methyl-3-(4-phenoxyphenyl)propanoic acid (3f)

Following the general arylation procedure A (eluent: hexane/ethyl acetate = 2/1 with 1% v/v of acetic acid). The product of **3f** as acid was obtained (white solid, 19.3 mg, 48% yield).

The enantiomeric purity of the substrate was determined by SFC analysis (CHIRALPAK® IC-3 column, 15% ^{i}PrOH / CO_2, flow rate 2 mL/min, retention time 7.828 min (minor) and 8.750 min (major), 7:93 er).

^{1}H NMR (600 MHz, $CDCl_3$) δ 7.80 – 7.75 (m, 2H), 7.72 – 7.69 (m, 2H), 7.31 (t, *J* = 7.8 Hz, 2H), 7.10 – 7.01 (m, 3H), 6.94 (d, *J* = 7.8 Hz, 2H), 6.84 (d, *J* = 8.0 Hz, 2H), 3.80 (d, *J* = 13.8 Hz, 1H), 3.22 (d, *J* = 14.0 Hz, 1H), 1.99 (s, 3H). ^{13}C NMR (150 MHz, $CDCl_3$) δ 168.45, 157.55, 156.24, 134.27, 131.84, 131.56, 130.65, 129.75, 123.34, 123.09, 119.13, 118.63, 118.59, 40.35, 29.82, 21.97.

HRMS (ESI-TOF) Calcd for $C_{24}H_{18}NO_5$ $[M-H]^-$: 400.1185; found: 400.1173.

The absolute stereochemistry was assigned based on comparing the specific rotation of **3k** with literature.

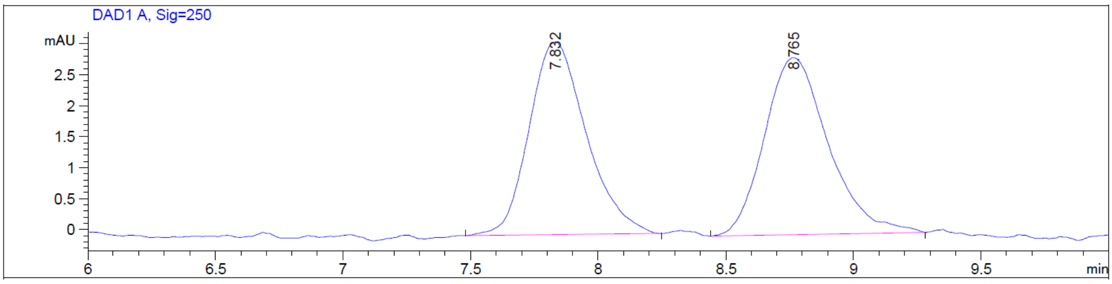

Peak #	RetTime [min]	Type	Width [min]	Area [mAU*s]	Height [mAU]	Area %
1	7.832	BB	0.2357	48.33260	3.10399	49.3113
2	8.765	BB	0.2594	49.68258	2.84940	50.6887

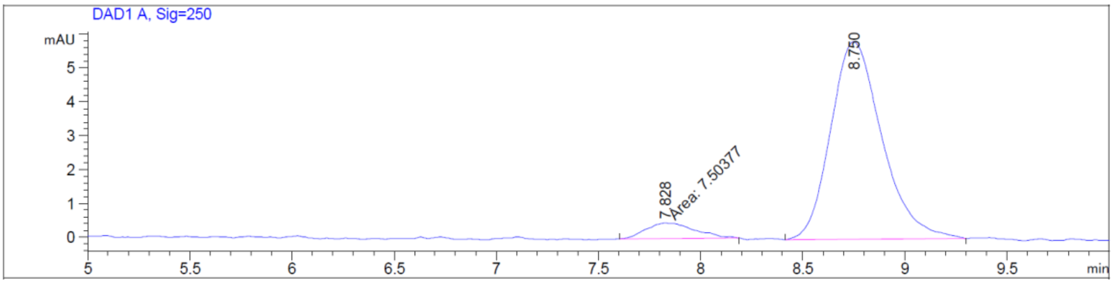

Peak #	RetTime [min]	Type	Width [min]	Area [mAU*s]	Height [mAU]	Area %
1	7.828	MM	0.2767	7.50377	4.52053e-1	6.8638
2	8.750	BB	0.2634	101.82076	5.84066	93.1362

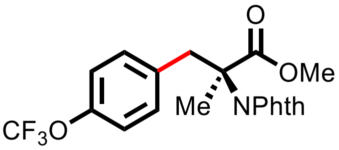

Methyl (S)-2-(1,3-dioxoisoindolin-2-yl)-2-methyl-3-(4-(trifluoromethoxy)phenyl)propanoate (3g)

Following the general arylation procedure A (eluent: toluene/ethyl acetate = 20/1). The product of **3g** as ester was obtained (yellow oil, 29.7 mg, 73% yield).

The enantiomeric purity of the substrate was determined by SFC analysis (CHIRALPAK® AD-3 column, 10% ^{i}PrOH / CO_2, flow rate 2 mL/min, retention time 3.258 min (major) and 3.818 min (minor), 96:4 er).

1H NMR (600 MHz, $CDCl_3$) δ 7.80 – 7.75 (m, 2H), 7.72 – 7.69 (m, 2H), 7.09 (d, *J* = 8.4 Hz, 2H), 7.01 (d, *J* = 8.2 Hz, 2H), 3.78 (d, J = 13.9 Hz, 1H), 3.74 (s, 3H), 3.27 (d, *J* = 13.9 Hz, 1H), 1.89 (s, 3H). ^{13}C NMR (150 MHz, $CDCl_3$) δ 172.68, 168.40, 148.49, 134.57, 134.33, 131.89, 131.52, 123.36, 120.73, 120.49 (q, *J* = 255.0), 63.91, 52.83, 40.77, 22.10. ^{19}F NMR (376 MHz, $CDCl_3$) δ -60.57. HRMS (ESI-TOF) Calcd for $C_{20}H_{15}F_3NO_5$ $[M-H]^-$: 406.0902; found: 406.0894.

The absolute stereochemistry was assigned based on comparing the specific rotation of **3k** with literature.

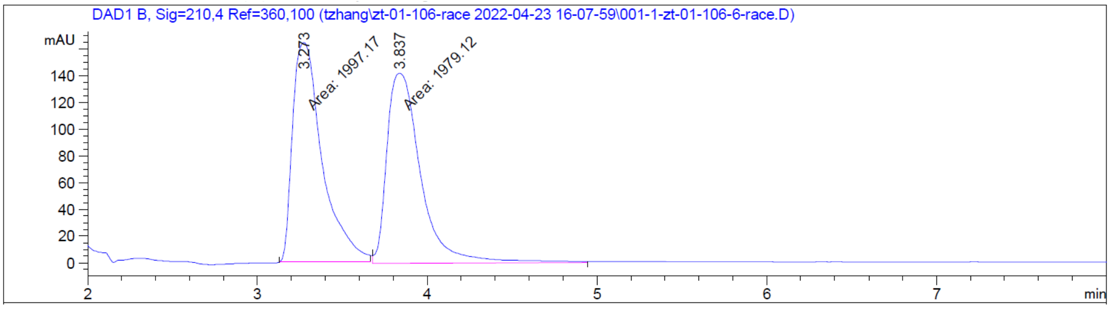

Peak #	RetTime [min]	Type	Width [min]	Area [mAU*s]	Height [mAU]	Area %
1	3.273	MM	0.2025	1997.16846	164.36981	50.2269
2	3.837	MM	0.2319	1979.12439	142.26338	49.7731

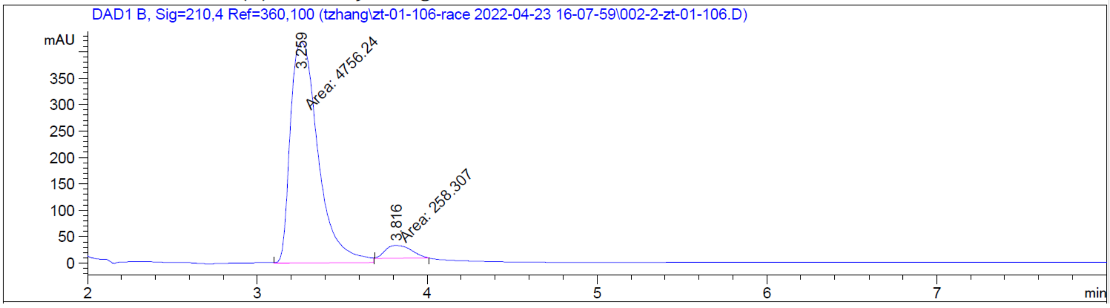

Peak #	RetTime [min]	Type	Width [min]	Area [mAU*s]	Height [mAU]	Area %
1	3.258	MM	0.1930	159.22154	13.74660	95.8802
2	3.818	MM	0.1690	6.84151	6.74858e-1	4.1198

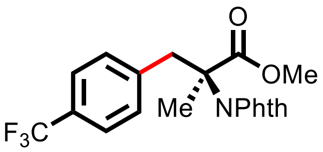

Methyl (S)-2-(1,3-dioxoisoindolin-2-yl)-2-methyl-3-(4-(trifluoromethyl)phenyl)propanoate (3h)[2]

Following the general arylation procedure A (eluent: toluene/ethyl acetate = 20/1). The product of **3h** as ester was obtained (yellow oil, 20.3 mg, 52% yield).

The enantiomeric purity of the substrate was determined by SFC analysis (CHIRALPAK® IC-3 column, 5% ^{i}PrOH / CO_2, flow rate 2 mL/min, retention time 4.345 min (major) and 4.558 min (minor), 95.5:4.5 er).

^{1}H NMR (600 MHz, $CDCl_3$) δ 7.81 – 7.76 (m, 2H), 7.75 – 7.70 (m, 2H), 7.43 (d, *J* = 8.0 Hz, 2H), 7.19 (d, *J* = 8.0 Hz, 2H), 3.84 (d, *J* = 13.8 Hz, 1H), 3.75 (s, 3H), 3.33 (d, *J* = 13.8 Hz, 1H), 1.89 (s, 3H). ^{13}C NMR (150 MHz, $CDCl_3$) δ 172.58, 168.40, 139.98, 134.39, 131.48, 130.92, 129.47 (q, *J* = 31.5 Hz), 125.20 (q, *J* = 3.0 Hz), 123.42 (q, *J* = 270.0 Hz), 123.41, 63.81, 52.88, 41.27, 22.05. ^{19}F NMR (376 MHz, $CDCl_3$) δ -65.16. HRMS (ESI-TOF) Calcd for $C_{20}H_{15}F_3NO_4$ $[M-H]^-$: 390.0953; found: 390.0938.

The absolute stereochemistry was assigned based on comparing the specific rotation of **3k** with literature.

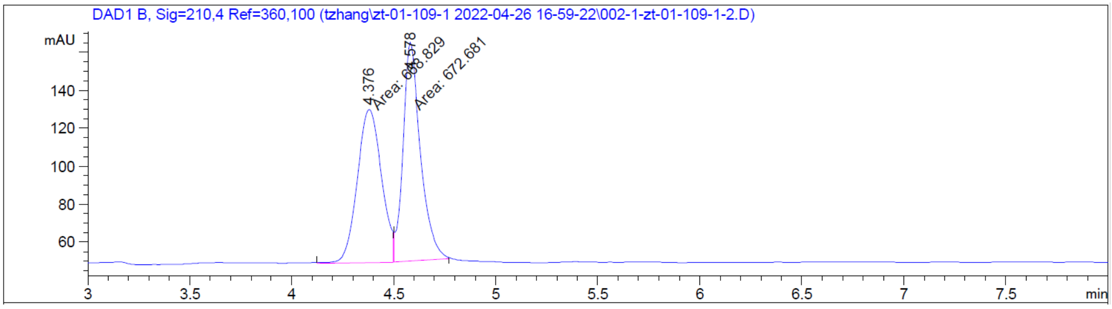

Peak #	RetTime [min]	Type	Width [min]	Area [mAU*s]	Height [mAU]	Area %
1	4.376	MM	0.1379	668.82922	80.82766	49.8564
2	4.578	MM	0.0972	672.68073	115.34596	50.1436

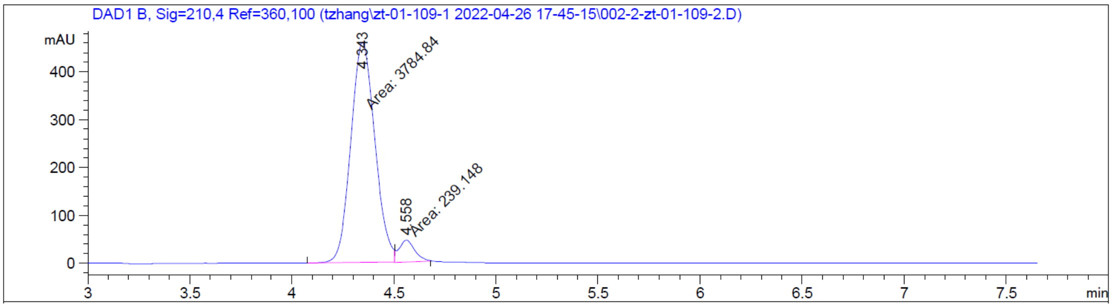

Peak #	RetTime [min]	Type	Width [min]	Area [mAU*s]	Height [mAU]	Area %
1	4.343	MM	0.1445	127.24140	14.67991	95.4131
2	4.558	MM	0.0738	6.11708	1.38096	4.5869

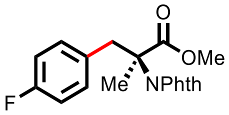

Methyl (*S*)-2-(1,3-dioxoisoindolin-2-yl)-3-(4-fluorophenyl)-2-methylpropanoate (3i)[2]

Following the general arylation procedure A (eluent: toluene/ethyl acetate = 20/1). The product of **3i** as ester was obtained (yellow oil, 21.5 mg, 63% yield).

The enantiomeric purity of the substrate was determined by SFC analysis (CHIRALPAK® OJ-3 column, 5% ^{i}PrOH / CO_2, flow rate 2 mL/min, retention time 5.331 min (minor) and 6.543 min (major), 5:95 er).

^{1}H NMR (600 MHz, $CDCl_3$) δ 7.80 – 7.74 (m, 2H), 7.74 – 7.66 (m, 2H), 7.05 – 6.96 (m, 2H), 6.88 – 6.78 (t, *J* = 8.5 Hz, 2H), 3.84 – 3.64 (m, 4H), 3.23 (d, *J* = 14.0 Hz, 1H), 1.88 (s, 3H). ^{13}C NMR (150 MHz, $CDCl_3$) δ 172.83, 168.44, 162.15 (d, *J* = 244.5 Hz), 134.29, 132.0 (d, *J* = 7.5 Hz), 131.55, 131.49 (d, *J* = 3.0 Hz), 123.36, 115.2 (d, *J* = 21.0 Hz), 64.01, 52.80, 40.61, 21.98. ^{19}F NMR (376 MHz, $CDCl_3$) δ -118.31. HRMS (ESI-TOF) Calcd for $C_{19}H_{15}FNO_4$ $[M-H]^-$: 340.0985; found: 340.0974.

The absolute stereochemistry was assigned based on comparing the specific rotation of **3k** with literature.

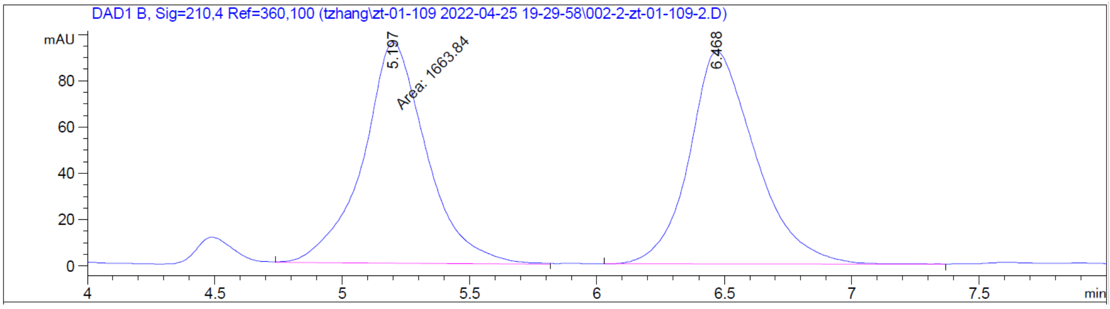

Peak #	RetTime [min]	Type	Width [min]	Area [mAU*s]	Height [mAU]	Area %
1	5.197	MM	0.2902	1663.83972	95.56364	49.9618
2	6.468	BB	0.2674	1666.38220	91.96693	50.0382

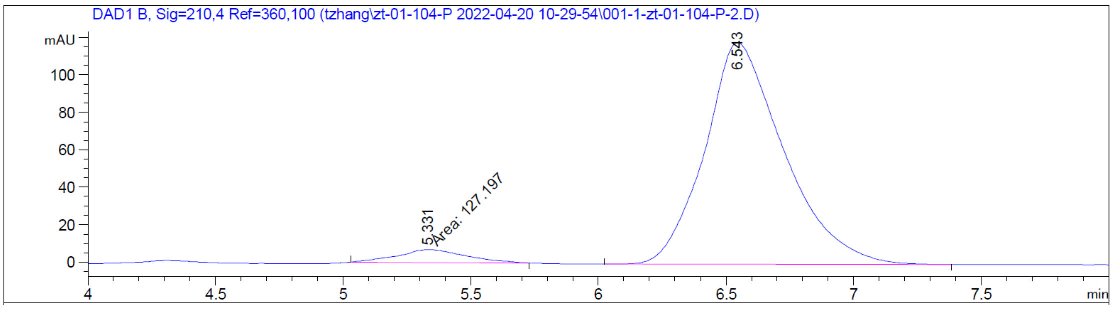

Peak #	RetTime [min]	Type	Width [min]	Area [mAU*s]	Height [mAU]	Area %
1	5.331	MM	0.3077	127.19695	6.88965	4.7437
2	6.543	BB	0.3059	2554.21558	118.31834	95.2563

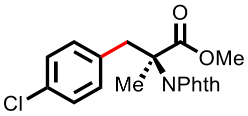

Methyl (S)-3-(4-chlorophenyl)-2-(1,3-dioxoisoindolin-2-yl)-2-methylpropanoate (3g)[2]

Following the general arylation procedure A (eluent: toluene/ethyl acetate = 20/1). The product of **3g** as ester was obtained (yellow oil, 26.4 mg, 74% yield).

The enantiomeric purity of the substrate was determined by SFC analysis (CHIRALPAK® OJ-3 column, 5% ^{i}PrOH / CO_2, flow rate 2 mL/min, retention time 7.777 min (minor) and 12.523 min (major), 5:95 er).

^{1}H NMR (600 MHz, $CDCl_3$) δ 7.80 – 7.74 (m, 2H), 7.74 – 7.66 (m, 2H), 7.16 (d, *J* = 8.4 Hz, 2H), 7.02 (d, *J* = 8.4 Hz, 2H), 3.80 – 3.74 (m, 4H), 3.26 (d, *J* = 13.8 Hz, 1H), 1.90 (s, 3H). ^{13}C NMR (150 MHz, $CDCl_3$) δ 172.75, 168.43, 134.32, 134.26, 133.19, 131.85, 131.53, 128.50, 123.40, 63.92, 52.84, 40.79, 21.98. HRMS (ESI-TOF) Calcd for $C_{19}H_{15}ClNO_4$ $[M-H]^-$: 356.0690; found: 356.0677.

The absolute stereochemistry was assigned based on comparing the specific rotation of **3k** with literature.

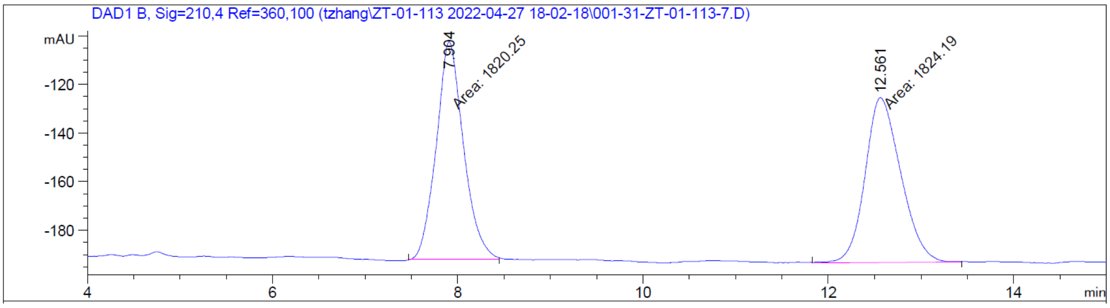

Peak #	RetTime [min]	Type	Width [min]	Area [mAU*s]	Height [mAU]	Area %
1	7.904	MM	0.3399	1820.24670	89.26306	49.9459
2	12.561	MM	0.4481	1824.19165	67.85536	50.0541

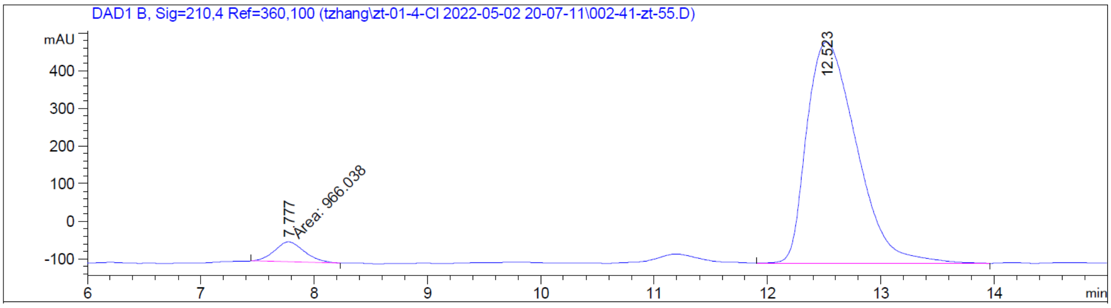

Peak #	RetTime [min]	Type	Width [min]	Area [mAU*s]	Height [mAU]	Area %
1	7.777	MM	0.3040	966.03766	52.96403	5.1014
2	12.523	VB	0.4924	1.79708e4	588.20062	94.8986

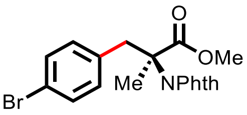

Methyl (S)-3-(4-bromophenyl)-2-(1,3-dioxoisoindolin-2-yl)-2-methylpropanoate (3k)[2]

Following the general arylation procedure A (eluent: toluene/ethyl acetate = 20/1). The product of **3k** as ester was obtained (yellow oil, 26.5 mg, 66% yield).

The enantiomeric purity of the substrate was determined by SFC analysis (CHIRALPAK® OJ-3 column, 5% ^{i}PrOH / CO_2, flow rate 2 mL/min, retention time 3.521 min (minor) and 6.007 min (major), 1:99 er).

^{1}H NMR (600 MHz, $CDCl_3$) δ 7.80 – 7.75 (m, 2H), 7.75 – 7.69 (m, 2H), 7.29 (d, *J* = 8.2 Hz, 2H), 6.93 (d, *J* = 8.2 Hz, 2H), 3.76 – 3.70 (m, 4H), 3.22 (d, *J* = 13.9 Hz, 1H), 1.87 (s, 3H). ^{13}C NMR (150 MHz, $CDCl_3$) δ 172.70, 168.41, 134.77, 134.32, 132.22, 131.51, 131.43, 123.39, 121.34, 63.83, 52.83, 40.85, 21.96. HRMS (ESI-TOF) Calcd for C19H19BrNO5 $[M+H_3O]^+$: 420.0447; found: 420.0443.

The absolute stereochemistry was assigned based on comparing the specific rotation of **3k** with literature.

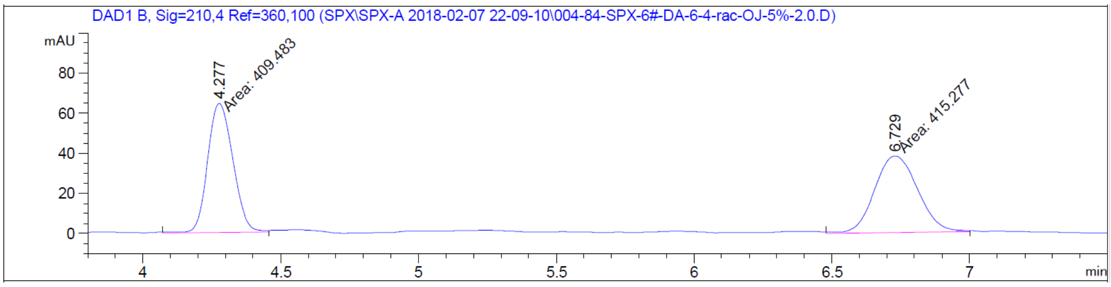

Peak #	RetTime [min]	Type	Width [min]	Area [mAU*s]	Height [mAU]	Area %
1	4.277	MM	0.1059	409.48337	64.43553	49.6488
2	6.729	MM	0.1811	415.27725	38.21263	50.3512

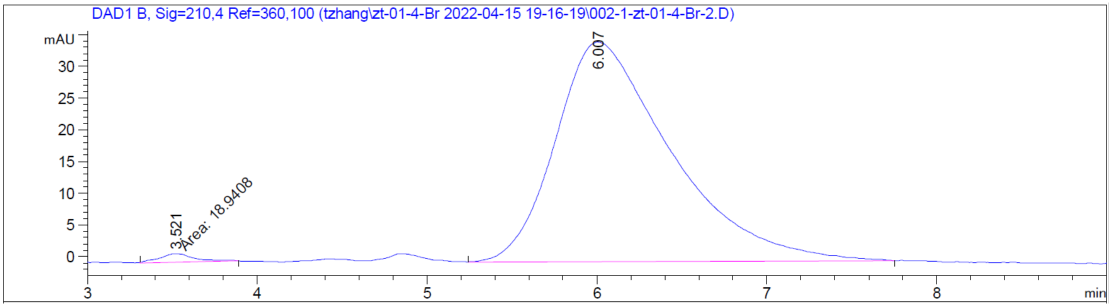

Peak #	RetTime [min]	Type	Width [min]	Area [mAU*s]	Height [mAU]	Area %
1	3.521	MM	0.2342	18.94079	1.34819	1.1427
2	6.007	BB	0.6527	1638.62183	34.58616	98.8573

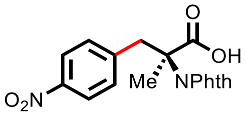

(*S*)-2-(1,3-dioxoisoindolin-2-yl)-2-methyl-3-(4-nitrophenyl)propanoic acid (3l)

Following the general arylation procedure A (eluent: hexane/ethyl acetate = 2/1 with 1% v/v of acetic acid). The product of **3l** as acid was obtained (yellow solid, 22.7 mg, 64% yield).

The enantiomeric purity of the substrate was determined by SFC analysis (CHIRALPAK® OJ-3 column, 15% ^{i}PrOH / CO_2, flow rate 2 mL/min, retention time 5.719 min (minor) and 7.519 min (major), 3.5:96.5er).

^{1}H NMR (600 MHz, $CDCl_3$) δ 8.10 (d, J = 8.5 Hz, 2H), 7.86 – 7.80 (m, 2H), 7.79 – 7.75 (m, 2H), 7.29 (d, J = 8.6 Hz, 2H), 3.93 (d, J = 13.6 Hz, 1H), 3.38 (d, J = 13.6 Hz, 1H), 1.96 (s, 3H). ^{13}C NMR (150 MHz, $CDCl_3$) δ 177.22, 168.37, 147.34, 143.34, 134.66, 131.44, 131.26, 123.64, 123.62, 63.52, 41.04, 21.92. HRMS (ESI-TOF) Calcd for $C_{18}H_{13}N_2O_6$ [M-H]$^-$: 353.0774; found: 353.0764.

The absolute stereochemistry was assigned based on comparing the specific rotation of **3k** with literature.

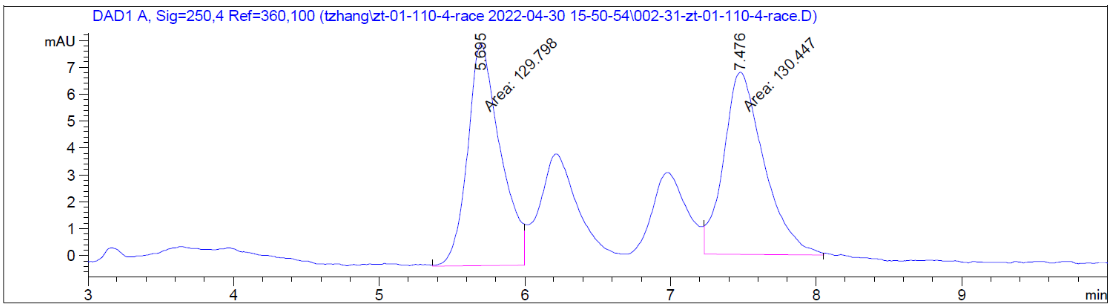

Peak #	RetTime [min]	Type	Width [min]	Area [mAU*s]	Height [mAU]	Area %
1	5.695	MM	0.2624	129.79773	8.24542	49.8753
2	7.476	MM	0.3214	130.44655	6.76474	50.1247

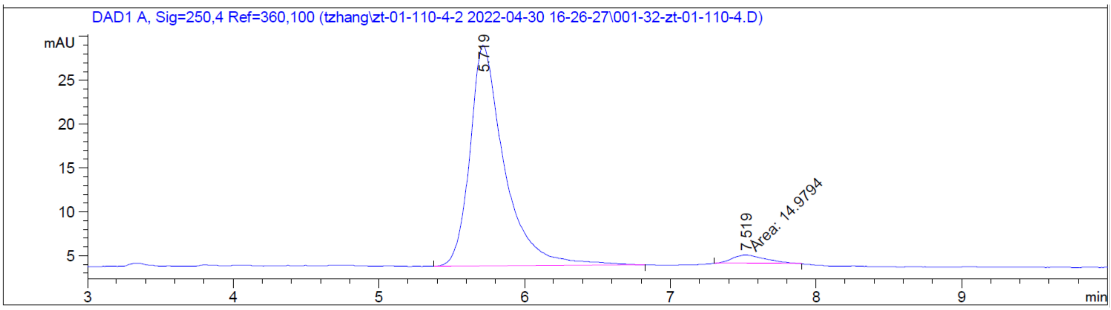

Peak #	RetTime [min]	Type	Width [min]	Area [mAU*s]	Height [mAU]	Area %
1	5.719	BB	0.2358	414.32858	25.19997	96.5108
2	7.519	MM	0.2623	14.97943	9.51787e-1	3.4892

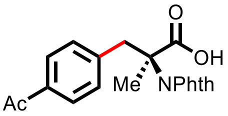

(*S*)-3-(4-acetylphenyl)-2-(1,3-dioxoisoindolin-2-yl)-2-methylpropanoic acid (3m)

Following the general arylation procedure A (eluent: hexane/ethyl acetate = 2/1 with 1% v/v of acetic acid). The product of **3m** as acid was obtained (yellow solid, 21.8 mg, 62% yield).

The enantiomeric purity of the substrate was determined by SFC analysis (CHIRALPAK® OJ-3 column, 15% ^{i}PrOH / CO_2, flow rate 2 mL/min, retention time 10.580 min (minor) and 12.750 min (major), 5:95 er).

^{1}H NMR (600 MHz, $CDCl_3$) δ 7.86 – 7.77 (m, 4H), 7.77 – 7.71 (m, 2H), 7.19 (d, *J* = 8.1 Hz, 2H), 3.87 (d, *J* = 13.7 Hz, 1H), 3.31 (d, *J* = 13.7 Hz, 1H), 2.57 (s, 3H), 1.98 (s, 3H). ^{13}C NMR (150 MHz, $CDCl_3$) δ 198.09, 177.06, 168.45, 141.36, 136.00, 134.44, 131.40, 130.83, 128.47, 123.51, 63.67, 41.02, 26.67, 21.93. HRMS (ESI-TOF) Calcd for $C_{20}H_{16}NO_5$ $[M-H]^-$: 350.1028; found: 350.1027.

The absolute stereochemistry was assigned based on comparing the specific rotation of **3k** with literature.

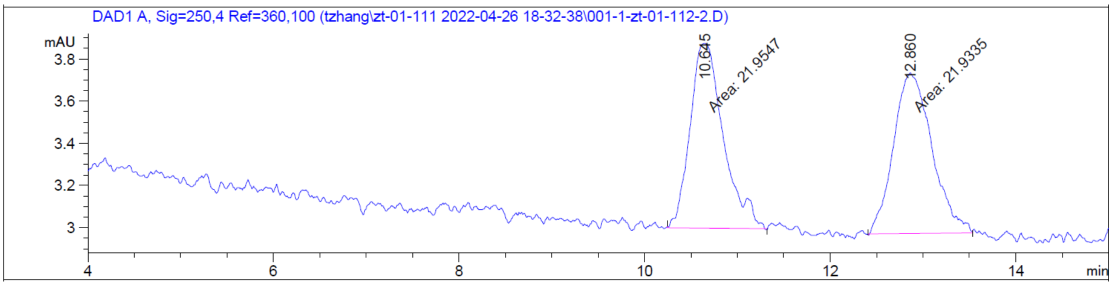

Peak #	RetTime [min]	Type	Width [min]	Area [mAU*s]	Height [mAU]	Area %
1	10.645	MM	0.4174	21.95472	8.76609e-1	50.0242
2	12.860	MM	0.4790	21.93352	7.63213e-1	49.9758

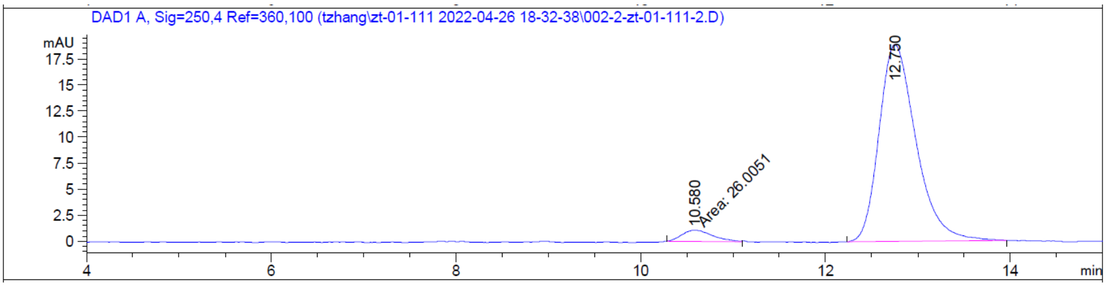

Peak #	RetTime [min]	Type	Width [min]	Area [mAU*s]	Height [mAU]	Area %
1	10.580	MM	0.3918	26.00514	1.10629	4.7162
2	12.750	BB	0.4204	525.39618	18.89387	95.2838

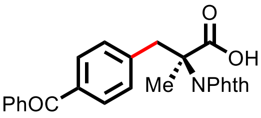

(*S*)-3-(4-benzoylphenyl)-2-(1,3-dioxoisoindolin-2-yl)-2-methylpropanoic acid (3n)

Following the general arylation procedure A (eluent: hexane/ethyl acetate = 2/1 with 1% v/v of acetic acid). The product of **3n** as acid was obtained (white solid, 26.8 mg, 65% yield).

The enantiomeric purity of the substrate was determined by SFC analysis (CHIRALPAK® IC-3 column, 15% ^{i}PrOH / CO_2, flow rate 2 mL/min, retention time 5.926 min (minor) and 6.450 min (major), 6:94 er).

^{1}H NMR (600 MHz, $CDCl_3$) δ 7.79 – 7.74 (m, 2H), 7.73 – 7.68 (m, 4H), 7.63 (d, J = 8.1 Hz, 2H), 7.57 – 7.52 (m, 1H), 7.47 – 7.40 (m, 2H), 7.20 (d, J = 8.2 Hz, 2H), 3.91 (d, J = 13.8 Hz, 1H), 3.33 (d, J = 13.7 Hz, 1H), 1.99 (s, 3H). ^{13}C NMR (150 MHz, $CDCl_3$) δ 196.59, 177.63, 168.40, 140.56, 137.69, 136.34, 134.42, 132.46, 131.40, 130.52, 130.22, 130.10, 128.31, 123.47, 63.71, 41.02, 21.97. HRMS (ESI-TOF) Calcd for $C_{25}H_{20}NO_5$ $[M+H]^+$: 414.1341; found: 414.1344.

The absolute stereochemistry was assigned based on comparing the specific rotation of **3k** with literature.

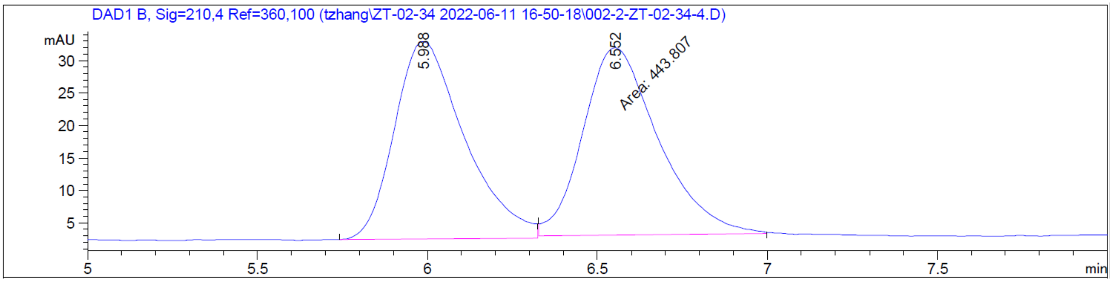

Peak #	RetTime [min]	Type	Width [min]	Area [mAU*s]	Height [mAU]	Area %
1	5.988	BV	0.2134	436.20914	30.40289	49.5683
2	6.552	MM	0.2564	443.80707	28.84396	50.4317

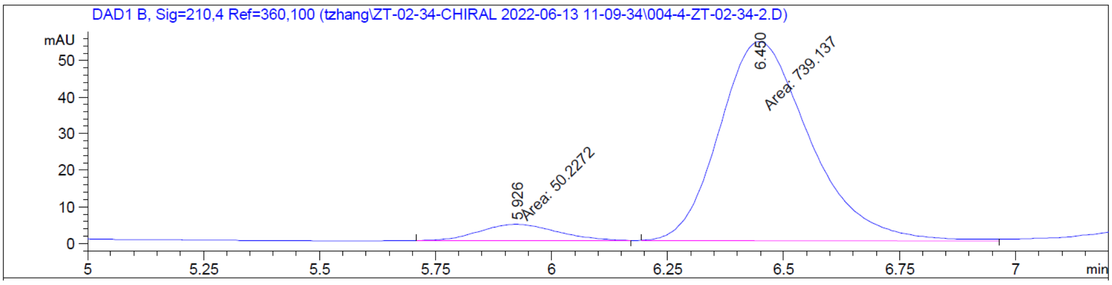

Peak #	RetTime [min]	Type	Width [min]	Area [mAU*s]	Height [mAU]	Area %
1	5.926	MM	0.1915	50.22721	4.37101	6.3630
2	6.450	MM	0.2263	739.13733	54.44294	93.6370

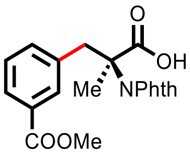

(*S*)-2-(1,3-dioxoisoindolin-2-yl)-3-(3-(methoxycarbonyl)phenyl)-2 methylpropa- -noic acid (3o)[2]

Following the general arylation procedure A (eluent: hexane/ethyl acetate = 2/1 with 1% v/v of acetic acid). The product of **3o** as acid was obtained (yellow oil, 20.7 mg, 57% yield).

The enantiomeric purity of the substrate was determined by SFC analysis (CHIRALPAK® IC-3 column, 15% ^{i}PrOH / CO_2, flow rate 2 mL/min, retention time 7.465 min (minor) and 8.231 min (major), 4:96 er).

^{1}H NMR (600 MHz, $CDCl_3$) δ 7.91– 7.86 (m, 1H), 7.82 – 7.71 (m, 2H), 7.76 – 7.69 (m, 3H), 7.32 – 7.27 (m, 2H), 3.85 (d, *J* = 13.9 Hz, 1H), 3.76 (s, 3H), 3.29 (d, *J* = 13.9 Hz, 1H), 1.98 (s, 3H). ^{13}C NMR (150 MHz, $CDCl_3$) δ 177.45, 168.43, 166.87, 135.98, 135.05, 134.29, 131.61, 131.50, 130.19, 128.58, 128.51, 123.43, 77.34, 52.12, 40.66, 21.84. HRMS (ESI-TOF) Calcd for $C_{20}H_{16}NO_6$ [M-H]$^-$: 366.0978; found: 366.0963.

The absolute stereochemistry was assigned based on comparing the specific rotation of **3k** with literature.

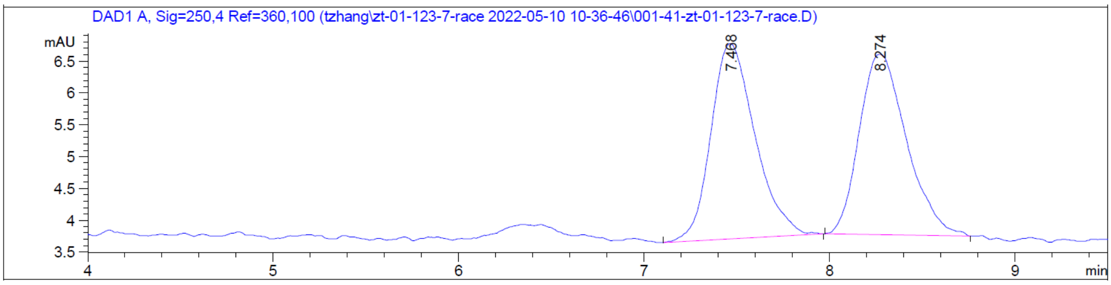

Peak #	RetTime [min]	Type	Width [min]	Area [mAU*s]	Height [mAU]	Area %
1	7.468	BB	0.2507	50.70457	3.06945	50.2126
2	8.274	BB	0.2708	50.27514	2.83656	49.7874

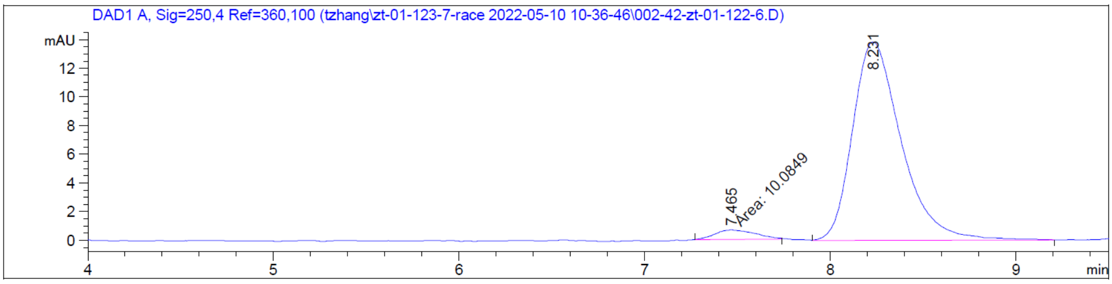

Peak #	RetTime [min]	Type	Width [min]	Area [mAU*s]	Height [mAU]	Area %
1	7.465	MM	0.2529	10.08494	6.64593e-1	3.8490
2	8.231	BB	0.2725	251.93147	13.83190	96.1510

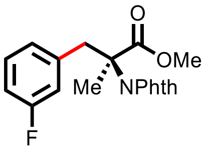

Methyl (S)-2-(1,3-dioxoisoindolin-2-yl)-3-(3-fluorophenyl)-2-methylpropanoate (3p)[2]

Following the general arylation procedure A (eluent: toluene/ethyl acetate = 20/1). The product of **3p** as ester was obtained (yellow oil, 21.8 mg, 64% yield).

The enantiomeric purity of the substrate was determined by SFC analysis (CHIRALPAK® OJ-3 column, 5% ^{i}PrOH / CO_2, flow rate 2 mL/min, retention time 4.963 min (minor) and 5.818 min (major), 4:96 er).

^{1}H NMR (600 MHz, $CDCl_3$) δ 7.80 – 7.74 (m, 2H), 7.74 – 7.66 (m, 2H), 7.17 – 7.08 (m, 1H), 6.88 (td, *J* = 8.4, 1.9 Hz, 1H), 6.82 (d, *J* = 7.6 Hz, 1H), 6.80 – 6.75 (m, 1H), 3.80 – 3.72 (m, 4H), 3.26 (d, *J* = 13.8 Hz, 1H), 1.89 (s, 3H). ^{13}C NMR (150 MHz, $CDCl_3$) δ 172.73, 168.45, 162.66 (d, *J* = 244.5 Hz), 138.26 (d, *J* = 7.5 Hz), 134.32, 131.55, 129.71 (d, *J* = 7.5 Hz), 126.26 (d, *J* = 3.0 Hz), 123.38, 117.5 (d, *J* = 21.0 Hz), 114.16 (d, *J* = 21.0 Hz) 63.92, 52.85, 41.15, 21.97. ^{19}F NMR (376 MHz, $CDCl_3$) δ -116.22. HRMS (ESI-TOF) Calcd for $C_{19}H_{15}FNO$ $[M-H]^-$: 340.0985; found: 340.0974. The absolute stereochemistry was assigned based on comparing the specific rotation of **3k** with literature.

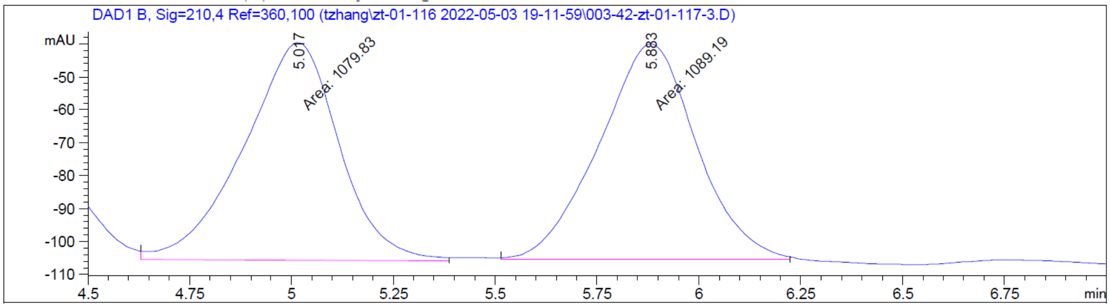

Peak #	RetTime [min]	Type	Width [min]	Area [mAU*s]	Height [mAU]	Area %
1	5.017	MM	0.2723	1079.82739	66.09604	49.7842
2	5.883	MM	0.2771	1089.19092	65.51367	50.2158

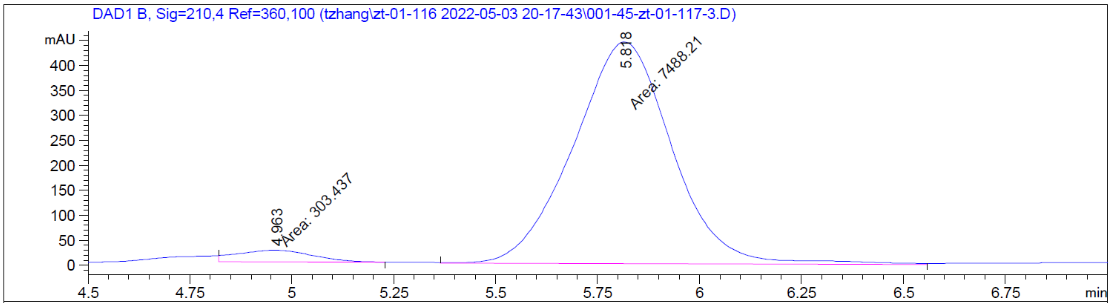

Peak #	RetTime [min]	Type	Width [min]	Area [mAU*s]	Height [mAU]	Area %
1	4.963	MM	0.2160	303.43668	23.41061	3.8944
2	5.818	MM	0.2807	7488.21436	444.55255	96.1056

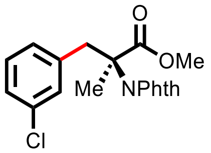

Methyl (*S*)-3-(3-chlorophenyl)-2-(1,3-dioxoisoindolin-2-yl)-2-methylpropanoate (3q)[2]

Following the general arylation procedure A (eluent: toluene/ethyl acetate = 20/1). The product of **3q** as ester was obtained (yellow oil, 21.8 mg, 61% yield).

The enantiomeric purity of the substrate was determined by SFC analysis (CHIRALPAK® OJ-3 column, 5% ^{i}PrOH / CO_2, flow rate 2 mL/min, retention time 8.111 min (minor) and 9.494 min (major), 4.5:95.5 er).

^{1}H NMR (600 MHz, $CDCl_3$) δ 7.80 – 7.74 (m, 2H), 7.74 – 7.66 (m, 2H), 7.20 – 7.16 (m, 1H), 7.12 (t, *J* = 7.8 Hz, 1H), 7.07 – 7.05 (m, 1H), 6.96 (d, *J* = 7.6 Hz, 1H), 3.80 – 3.74 (m, 4H), 3.26 (d, *J* = 13.8 Hz, 1H), 1.90 (s, 3H). ^{13}C NMR (150 MHz, $CDCl_3$) δ 172.66, 168.44, 137.80, 134.32, 134.10, 131.53, 130.71, 129.52, 128.71, 127.39, 123.38, 63.90, 52.84, 41.11, 21.94. HRMS (ESI-TOF) Calcd for $C_{19}H_{15}ClNO_4$ $[M-H]^-$: 356.0690; found: 356.0677.

The absolute stereochemistry was assigned based on comparing the specific rotation of **3k** with literature.

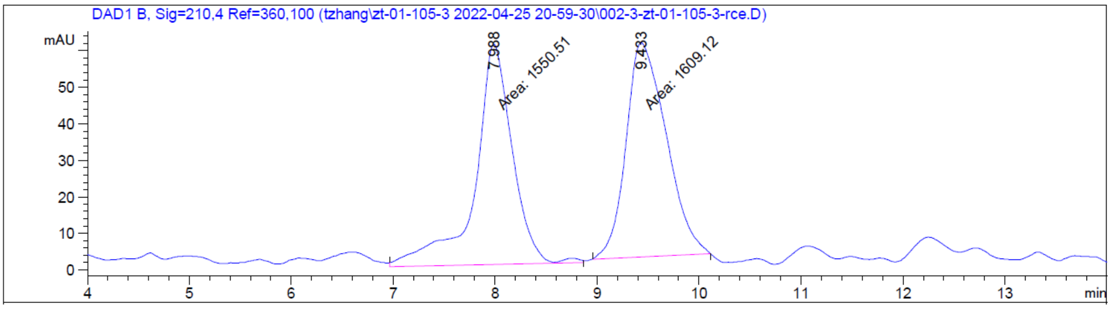

Peak #	RetTime [min]	Type	Width [min]	Area [mAU*s]	Height [mAU]	Area %
1	7.988	MM	0.4283	1550.50964	60.34021	49.0725
2	9.433	MM	0.4570	1609.12183	58.68290	50.9275

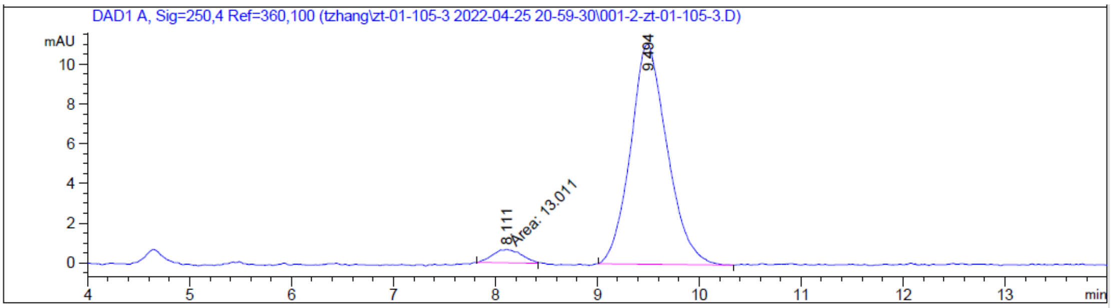

Peak #	RetTime [min]	Type	Width [min]	Area [mAU*s]	Height [mAU]	Area %
1	8.111	MM	0.3208	13.01100	6.75863e-1	4.4998
2	9.494	BB	0.3435	276.13489	11.05087	95.5002

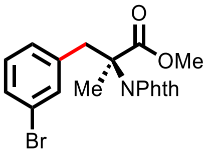

Methyl (*S*)-3-(3-bromophenyl)-2-(1,3-dioxoisoindolin-2-yl)-2-methylpropanoate (3r)[2]

Following the general arylation procedure A (eluent: toluene/ethyl acetate = 20/1). The product of **3r** as ester was obtained (yellow oil, 26.1 mg, 65% yield).

The enantiomeric purity of the substrate was determined by SFC analysis (CHIRALPAK® OJ-3 column, 10% ^{i}PrOH / CO_2, flow rate 2 mL/min, retention time 5.462 min (minor) and 6.010min (major), 5:95 er).

^{1}H NMR (600 MHz, $CDCl_3$) δ 7.80 – 7.74 (m, 2H), 7.74 – 7.66 (m, 2H), 7.32 (d, *J* = 7.9 Hz, 1H), 7.19 – 7.16 (m, 1H), 7.05 (t, *J* = 7.8 Hz, 1H), 7.00 (d, *J* = 7.6 Hz, 1H), 3.78 – 3.71 (m, 4H), 3.22 (d, *J* = 13.8 Hz, 1H), 1.88 (s, 3H). ^{13}C NMR (150 MHz, $CDCl_3$) δ 172.66, 168.45, 138.11, 134.33, 133.61, 131.52, 130.30, 129.82, 129.19, 123.40, 122.33, 63.91, 52.85, 41.09, 21.94. HRMS (ESI-TOF) Calcd for $C_{19}H_{15}BrNO_4$ [M-H]$^-$: 400.0184; found: 400.0168.

The absolute stereochemistry was assigned based on comparing the specific rotation of **3k** with literature.

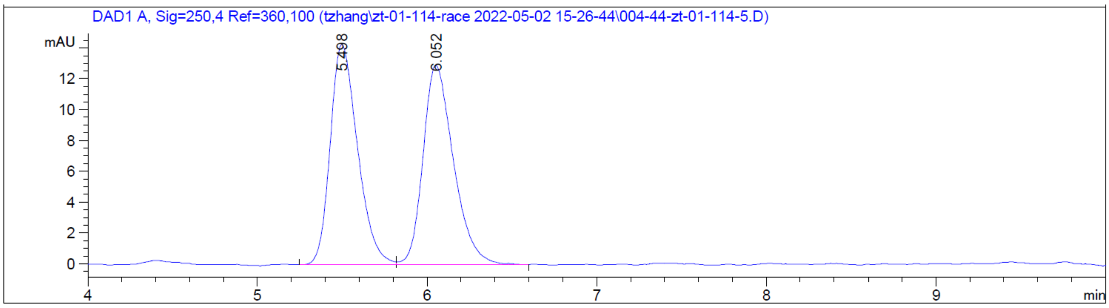

Peak #	RetTime [min]	Type	Width [min]	Area [mAU*s]	Height [mAU]	Area %
1	5.498	BV	0.1734	5434.99756	482.48297	50.1256
2	6.052	VB	0.1886	5407.75781	436.17612	49.8744

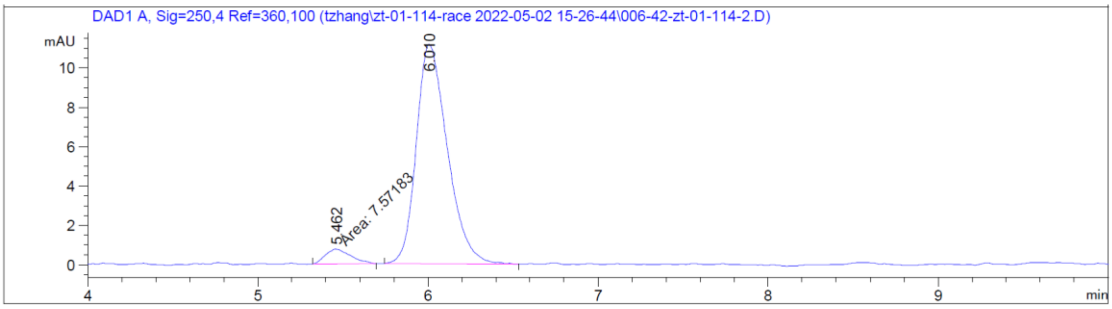

Peak #	RetTime [min]	Type	Width [min]	Area [mAU*s]	Height [mAU]	Area %
1	5.462	MM	0.1706	7.57183	7.39809e-1	5.0722
2	6.010	BB	0.1922	141.70815	11.15666	94.9278

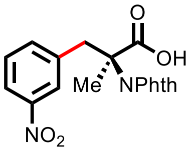

(*S*)-2-(1,3-dioxoisoindolin-2-yl)-2-methyl-3-(3-nitrophenyl)propanoic acid (3s)

Following the general arylation procedure A (eluent: hexane/ethyl acetate = 2/1 with 1% v/v of acetic acid). The product of **3s** as acid was obtained (white solid, 21.9 mg, 62% yield).

The enantiomeric purity of the substrate was determined by SFC analysis (CHIRALPAK® IC-3 column, 15% ^{i}PrOH / CO_2, flow rate 2 mL/min, retention time 5.835 min (minor) and 6.234 min (major), 5:95 er).

^{1}H NMR (600 MHz, $CDCl_3$) δ 8.07 (d, *J* = 8.2 Hz, 1H), 7.96 – 7.92 (m, 1H), 7.80 –

7.74 (m, 2H), 7.74 – 7.66 (m, 2H), 7.45 – 7.41 (m, 1H), 7.38 (t, *J* = 7.8 Hz, 1H), 3.88 (d, *J* = 14.0 Hz, 1H), 3.36 (d, *J* = 14.0 Hz, 1H), 1.98 (s, 3H). ^{13}C NMR (150 MHz, $CDCl_3$) δ 177.23, 168.40, 148.16, 137.65, 136.70, 134.62, 131.25, 129.36, 125.33, 123.59, 122.49, 63.45, 40.72, 21.96. HRMS (ESI-TOF) Calcd for $C_{18}H_{13}N_2O_6$ $[M-H]^-$: 353.0774; found: 353.0764.

The absolute stereochemistry was assigned based on comparing the specific rotation of **3k** with literature.

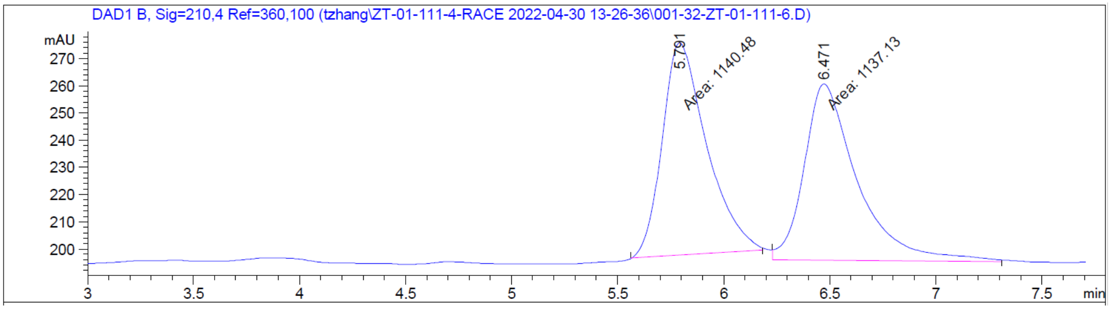

Peak #	RetTime [min]	Type	Width [min]	Area [mAU*s]	Height [mAU]	Area %
1	5.791	MM	0.2430	1140.48047	78.21394	50.0736
2	6.471	MM	0.2920	1137.12646	64.89987	49.9264

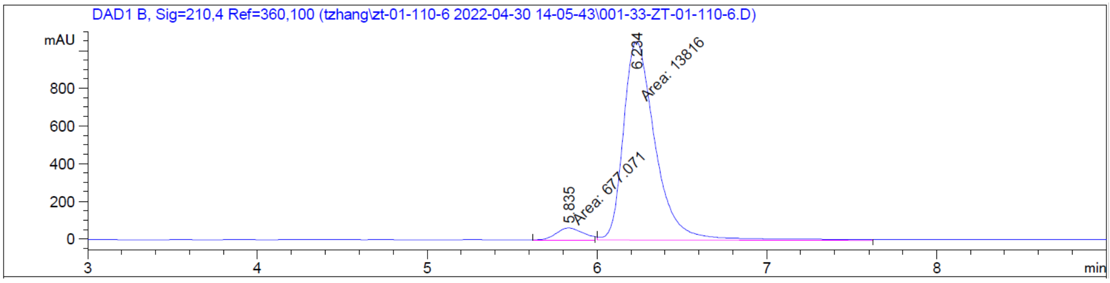

Peak #	RetTime [min]	Type	Width [min]	Area [mAU*s]	Height [mAU]	Area %
1	5.835	BV E	0.1736	123.84888	10.98252	4.8790
2	6.234	VB R	0.1927	2414.53516	189.48596	95.1210

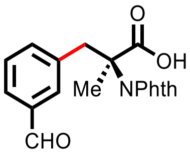

(*S*)-2-(1,3-dioxoisoindolin-2-yl)-3-(3-formylphenyl)-2-methylpropanoic acid (3t)

Following the general arylation procedure A (eluent: hexane/ethyl acetate = 2/1

with 1% v/v of acetic acid). The product of **3t** as acid was obtained (white solid, 22.9 mg, 68% yield).

The enantiomeric purity of the substrate was determined by SFC analysis (CHIRALPAK® IC-3 column, 15% ^{i}PrOH / CO_2, flow rate 2 mL/min, retention time 8.451 min (minor) and 14.604 min (major), 5.5:94.5 er).

^{1}H NMR (600 MHz, $CDCl_3$) δ 9.84 (s, 1H), 7.80 – 7.74 (m, 2H), 7.74 – 7.66 (m, 3H), 7.58 (s, 1H), 7.38 – 7.33 (m, 2H), 3.89 (d, J = 13.9 Hz, 1H), 3.34 (d, J = 13.9 Hz, 1H), 1.97 (s, 3H). ^{13}C NMR (150 MHz, $CDCl_3$) δ 192.34, 177.64, 168.43, 136.82, 136.66, 136.48, 134.51, 131.80, 131.38, 129.17, 128.82, 123.52, 63.69, 40.74, 21.91. HRMS (ESI-TOF) Calcd for $C_{19}H_{14}NO_5$ $[M-H]^-$: 336.0872; found: 336.0869.

The absolute stereochemistry was assigned based on comparing the specific rotation of **3k** with literature.

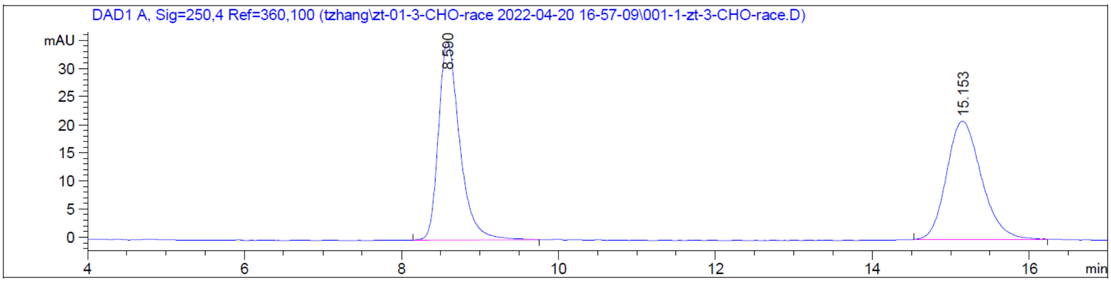

Peak #	RetTime [min]	Type	Width [min]	Area [mAU*s]	Height [mAU]	Area %
1	8.590	BB	0.2840	664.20215	35.22750	50.6118
2	15.153	BB	0.4642	648.14417	20.99567	49.3882

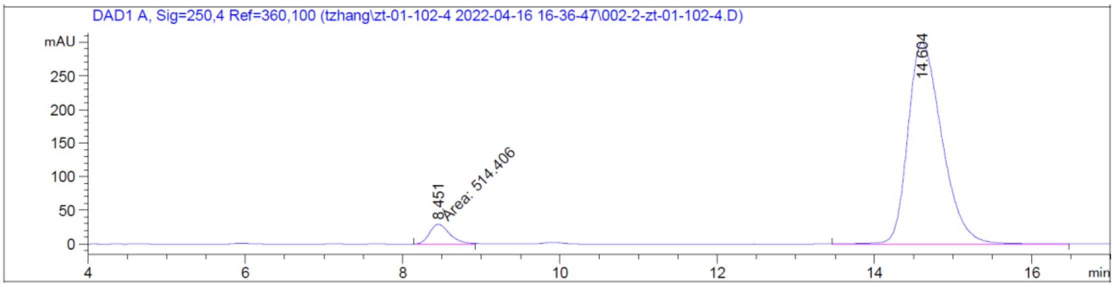

Peak #	RetTime [min]	Type	Width [min]	Area [mAU*s]	Height [mAU]	Area %
1	8.451	MM	0.2919	514.40631	29.37191	5.4027
2	14.604	BB	0.4630	9006.79590	299.36856	94.5973

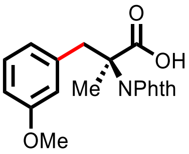

(*S*)-2-(1,3-dioxoisoindolin-2-yl)-3-(3-methoxyphenyl)-2-methylpropanoic acid (3u)

Following the general arylation procedure A (eluent: hexane/ethyl acetate = 2/1 with 1% v/v of acetic acid). The product of **3u** as acid was obtained (yellow oil, 19.0 mg, 56% yield).

The enantiomeric purity of the substrate was determined by SFC analysis (CHIRALPAK® IC-3 column, 15% ^{i}PrOH / CO_2, flow rate 2 mL/min, retention time 5.810 min (minor) and 10.267 min (major), 6:94 er).

^{1}H NMR (600 MHz, $CDCl_3$) δ 7.81 – 7.75 (m, 2H), 7.74 – 7.68 (m, 2H), 7.08 (t, *J* = 7.9 Hz, 1H), 6.73 (d, *J* = 8.2 Hz, 1H), 6.64 (d, *J* = 7.4 Hz, 1H), 6.56 (s, 1H), 3.76 (d, *J* = 13.8 Hz, 1H), 3.54 (s, 3H), 3.18 (d, *J* = 13.8 Hz, 1H), 1.99 (s, 3H). ^{13}C NMR (150 MHz, $CDCl_3$) δ 177.60, 168.44, 159.46, 137.02, 134.27, 131.60, 129.32, 123.39, 123.04, 115.93, 113.08, 63.86, 54.99, 40.91, 21.95. HRMS (ESI-TOF) Calcd for $C_{19}H_{16}NO$ $[M-H]^-$: 338.1028; found: 338.1015.

The absolute stereochemistry was assigned based on comparing the specific rotation of **3k** with literature.

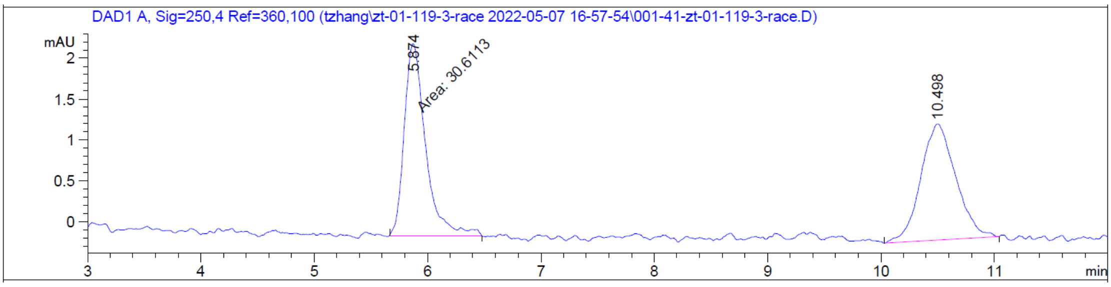

Peak #	RetTime [min]	Type	Width [min]	Area [mAU*s]	Height [mAU]	Area %
1	5.874	MM	0.2172	30.61133	2.34860	50.0430
2	10.498	BB	0.2644	30.55867	1.42368	49.9570

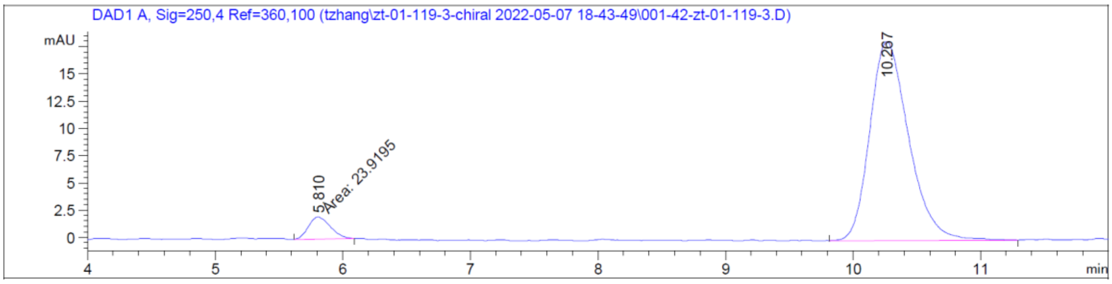

Peak #	RetTime [min]	Type	Width [min]	Area [mAU*s]	Height [mAU]	Area %
1	5.810	MM	0.1973	23.91954	2.02035	5.8183
2	10.267	BB	0.3172	387.18930	18.27238	94.1817

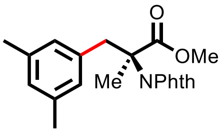

Methyl (*S*)-3-(3,5-dimethylphenyl)-2-(1,3-dioxoisoindolin-2-yl)-2-methylpropanoate (3v)

Following the general arylation procedure A (eluent: toluene/ethyl acetate = 20/1). The product of **3v** as ester was obtained (yellow oil, 22.1 mg, 63% yield).

The enantiomeric purity of the substrate was determined by SFC analysis (CHIRALPAK® OJ-3 column, 5% ^{i}PrOH / CO_2, flow rate 2 mL/min, retention time 5.174 min (major) and 6.513 min (minor), 96:4 er).

^{1}H NMR (600 MHz, $CDCl_3$) δ 7.80 – 7.75 (m, 2H), 7.74 – 7.66 (m, 2H), 6.80 (s, 1H), 6.61 (s, 2H), 3.76 (s, 3H), 3.68 (d, *J* = 13.8 Hz, 1H), 3.15 (d, *J* = 13.8 Hz, 1H), 2.09 (s, 6H), 1.88 (s, 3H). ^{13}C NMR (150 MHz, $CDCl_3$) δ 173.12, 168.50, 137.59, 135.51, 134.11, 131.71, 128.69, 128.48, 123.19, 64.20, 52.69, 41.07, 21.86, 21.15. HRMS (ESI-TOF) Calcd for $C_{21}H_{20}NO_4$ $[M-H]^-$: 350.1392; found: 350.1382.

The absolute stereochemistry was assigned based on comparing the specific rotation of **3k** with literature.

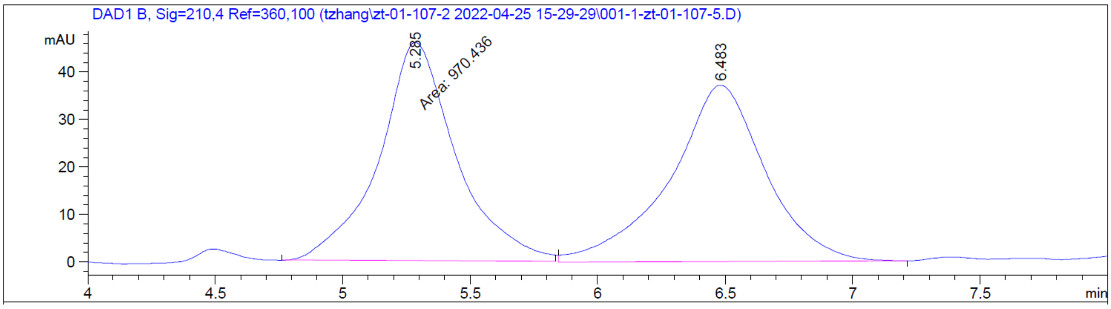

Peak #	RetTime [min]	Type	Width [min]	Area [mAU*s]	Height [mAU]	Area %
1	5.285	MM	0.3514	970.43604	46.03262	50.0306
2	6.483	VB	0.3723	969.24982	37.16303	49.9694

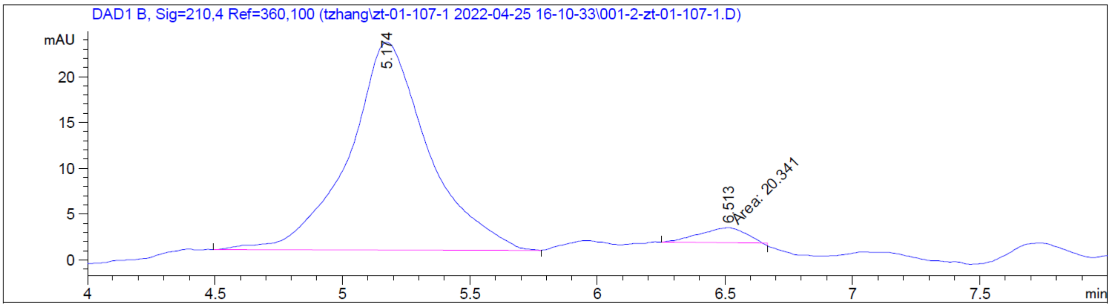

Peak #	RetTime [min]	Type	Width [min]	Area [mAU*s]	Height [mAU]	Area %
1	5.174	BB	0.2937	492.06659	22.63143	96.0303
2	6.513	MM	0.2068	20.34097	1.63942	3.9697

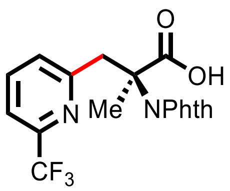

(S)-2-(1,3-dioxoisoindolin-2-yl)-2-methyl-3-(6-(trifluoromethyl)pyridin-2-yl)propanoic acid (3w)

Following the general arylation procedure A (eluent: hexane/ethyl acetate = 2/1 with 1% v/v of acetic acid). The product of **3w** as acid was obtained (yellow oil, 16.6 mg, 44% yield).

The enantiomeric purity of the substrate was determined by SFC analysis (CHIRALPAK® IC-3 column, 15% ^{i}PrOH / CO_2, flow rate 2 mL/min, retention time 6.150 min (minor) and 9.558 min (major), 3:97 er).

^{1}H NMR (600 MHz, $CDCl_3$) δ 8.09 (d, J = 8.5 Hz, 2H), 7.81 (d, J = 8.0 Hz, 2H), 7.73 – 7.68 (m, 2H), 7.29 (d, J = 8.4 Hz, 1H), 3.95 (d, J = 13.8 Hz, 1H), 3.39 (d, J = 13.8 Hz, 1H), 1.96 (s, 3H). ^{13}C NMR (150 MHz, $CDCl_3$) δ 177.28, 168.64, 157.30, 147.52, 137.76, 134.08, 131.77, 128.37, 121.07 (q, J = 273.0 Hz), 112.07 (q, J = 34.5 Hz) 118.76 (q, J = 3.0 Hz), 62.49, 42.10, 22.49. ^{19}F NMR (376 MHz, $CDCl_3$) δ -71.19. HRMS (ESI-TOF) Calcd for $C_{18}H_{14}F_3N_2O_4$ $[M+H]^+$: 379.0906; found: 379.0907.

The absolute stereochemistry was assigned based on comparing the specific rotation of **3k** with literature.

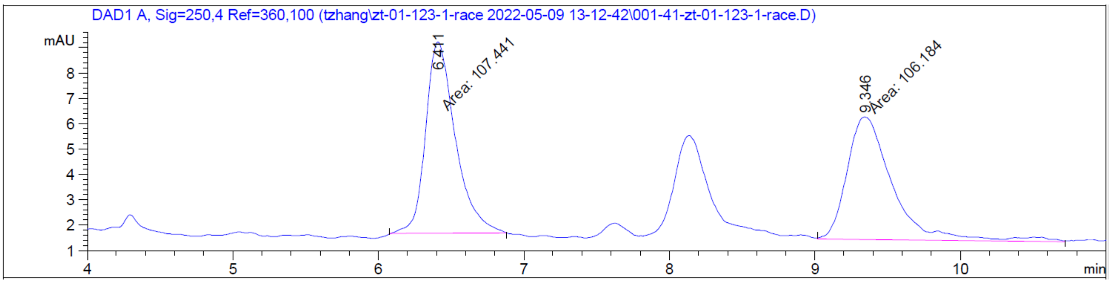

Peak #	RetTime [min]	Type	Width [min]	Area [mAU*s]	Height [mAU]	Area %
1	6.411	MM	0.2371	107.44058	7.55196	50.2941
2	9.346	MM	0.3654	106.18389	4.84345	49.7059

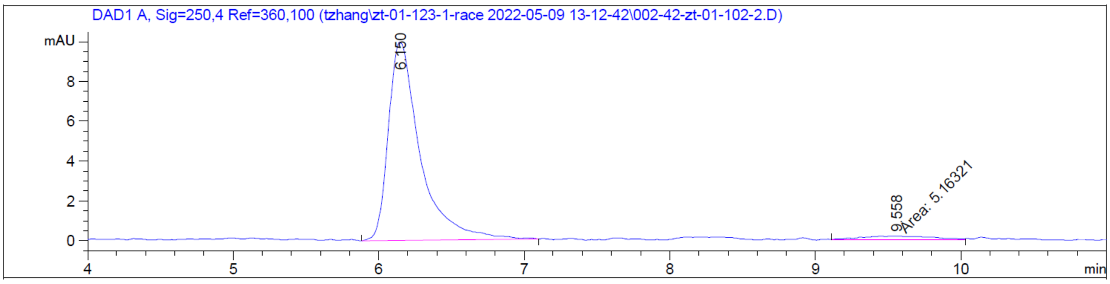

Peak #	RetTime [min]	Type	Width [min]	Area [mAU*s]	Height [mAU]	Area %
1	6.150	BB	0.2250	151.57800	9.98976	96.7059
2	9.558	MM	0.5100	5.16321	1.68742e-1	3.2941

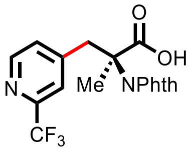

(S)-2-(1,3-dioxoisoindolin-2-yl)-2-methyl-3-(2-(trifluoromethyl)pyridin-4-yl) propanoic acid (3x)

Following the general arylation procedure A (eluent: hexane/ethyl acetate = 2/1 with 1% v/v of acetic acid). The product of **3x** as acid was obtained (yellow solid, 17.4 mg, 46% yield).

The enantiomeric purity of the substrate was determined by SFC analysis (CHIRALPAK® IC-3 column, 15% ^{i}PrOH / CO_2, flow rate 2 mL/min, retention time 7.627 min (major) and 10.286 min (minor), 97:3 er).

^{1}H NMR (600 MHz, $CDCl_3$) δ 8.61 (d, *J* = 4.4 Hz, 1H), 7.89 – 7.71 (m, 4H), 7.40 (s, 1H), 7.32 (d, *J* = 4.0 Hz, 1H), 3.87 (d, *J* = 13.6 Hz, 1H), 3.36 (d, *J* = 13.6 Hz, 1H), 1.99 (s, 3H). ^{13}C NMR (150 MHz, $CDCl_3$) δ 176.02, 168.35, 149.85, 148.06 (q, *J* = 34.5 Hz), 147.36, 134.69, 131.17, 128.61, 123.62, 122.59 (q, *J* = 3.0 Hz), 121.35 (q, *J* = 273 Hz), 63.11, 40.78, 22.22. ^{19}F NMR (376 MHz, $CDCl_3$) δ -70.87. HRMS (ESI-TOF) Calcd for $C_{18}H_{14}F_3N_2O4$ $[M+H]^+$: 379.0906; found: 379.0905.

The absolute stereochemistry was assigned based on comparing the specific rotation of **3k** with literature.

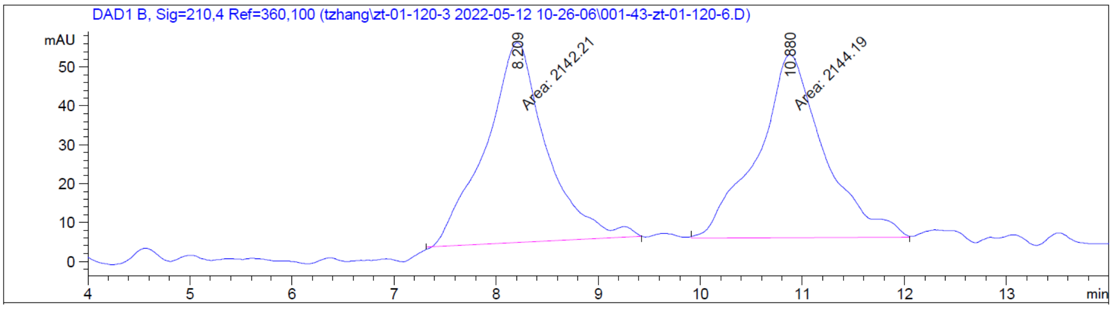

Peak #	RetTime [min]	Type	Width [min]	Area [mAU*s]	Height [mAU]	Area %
1	8.209	MM	0.6939	2142.21387	51.45042	49.9770
2	10.880	MM	0.7573	2144.18848	47.19033	50.0230

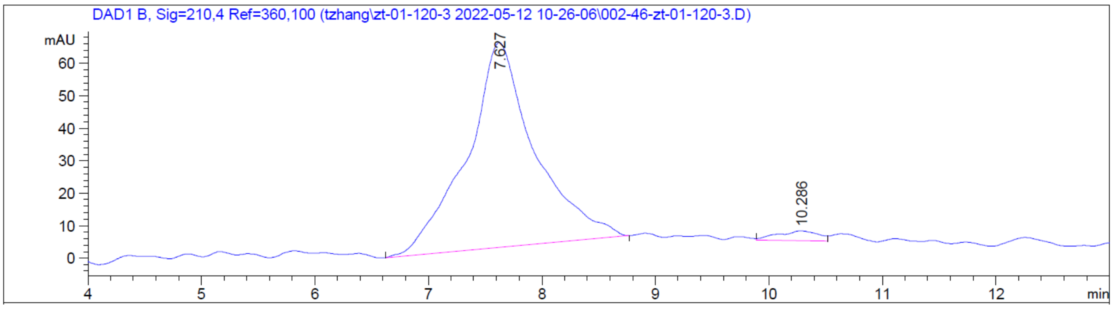

Peak #	RetTime [min]	Type	Width [min]	Area [mAU*s]	Height [mAU]	Area %
1	7.627	BB	0.5610	2624.20972	63.12369	97.2238
2	10.286	VV	0.3182	74.93275	3.03304	2.7762

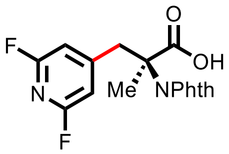

(*S*)-3-(2,6-difluoropyridin-4-yl)-2-(1,3-dioxoisoindolin-2-yl)-2-methylpropanoic acid (3y)

Following the general arylation procedure A (eluent: hexane/ethyl acetate = 2/1 with 1% v/v of acetic acid). The product of **3y** as acid was obtained (yellow oil, 15.6 mg, 45% yield).

The enantiomeric purity of the substrate was determined by SFC analysis (CHIRALPAK® OJ-3 column, 10% ^{i}PrOH / CO_2, flow rate 2 mL/min, retention time 6.498 min (major) and 8.282 min (minor), 99:1 er).

^{1}H NMR (600 MHz, $CDCl_3$) δ 7.80 – 7.74 (m, 2H), 7.76 – 7.67 (m, 3H), 6.78 – 6.72 (m, 1H), 3.77 (d, *J* = 14.4 Hz, 1H), 3.30 (d, *J* = 14.4 Hz, 1H), 1.97 (s, 3H). ^{13}C NMR (150 MHz, $CDCl_3$) δ 176.84, 168.44, 161.28 (dd, *J* = 90.0, 15.0 Hz), 159.65 (dd, *J* = 36.0, 13.5 Hz), 147.59 (dd, *J* = 4.5, 1.5 Hz), 134.53, 131.37, 123.57, 114.06 (dd, *J* = 22.5, 6.0 Hz), 106.43 (dd, *J* = 28.5, 6.0 Hz), 63.05, 33.80, 21.89. ^{19}F NMR (376 MHz, $CDCl_3$) δ -71.38. HRMS (ESI-TOF) Calcd for $C_{17}H_{13}F_2N_2O_4$ $[M+H]^+$: 347.0843; found: 347.0848.

The absolute stereochemistry was assigned based on comparing the specific rotation of **3k** with literature.

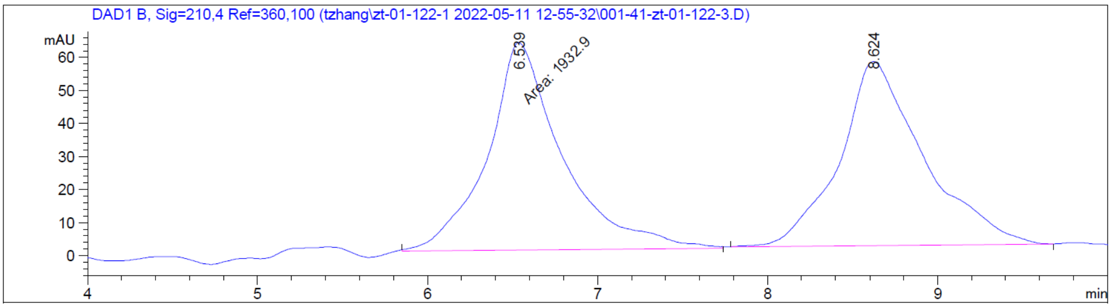

Peak #	RetTime [min]	Type	Width [min]	Area [mAU*s]	Height [mAU]	Area %
1	6.539	MM	0.5128	1932.90076	62.81881	50.3142
2	8.624	BB	0.4638	1908.75732	55.69393	49.6858

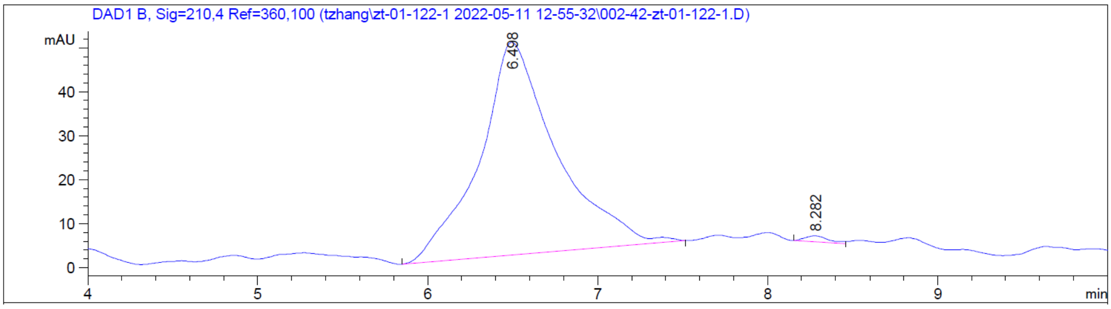

Peak #	RetTime [min]	Type	Width [min]	Area [mAU*s]	Height [mAU]	Area %
1	6.498	BV R	0.4219	1527.13245	48.42899	99.1186
2	8.282	BV	0.1366	13.57988	1.38241	0.8814

5. Enantioselective Arylation of Cylcopropanecarboxylic acid

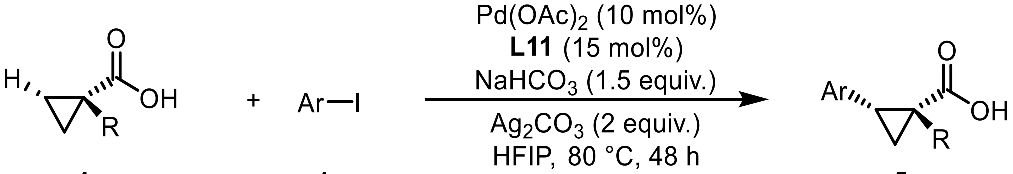

General procedure B for enantioselective arylation of cyclopropanecarboxylic acid:

$Pd(OAc)_2$ (2.2 mg, 0.01 mmol, 10 mol%), **L11** (5.0 mg, 0.015 mmol, 15 mol%), carboxylic acid 1 (0.1 mmol), Ag_2CO_3 (55 mg, 0.2 mmol, 2 equiv.), $NaHCO_3$ (12.6 mg, 0.15 mmol, 1.5 equiv.), and Iodoheterocycle (0.2 mmol, 2.0 equiv.) were weighed and placed in an 12 mL reaction tube. Subsequently, HFIP (1.0 mL) was injected, and the tube was capped and closed tightly. The reaction mixture was then stirred at 80 °C for 48 h. The mixture was allowed to cool to room temperature and acetic acid (0.05 ml) was added. Then, the mixture was passed through a pad of Celite with ethyl acetate as the eluent to remove any insoluble precipitate. The resulting solution was concentrated and the residual mixture was dissolved with a minimal amount of ethyl acetate and loaded onto a preparative TLC plate. The pure product was then isolated

using preparative TLC with ethyl acetate and hexanes (1/3 to 1/1) as the eluent and 1% v/v of acetic acid as the additive.

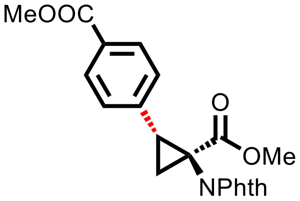

(1*S*,2*R*)-1-(1,3-dioxoisoindolin-2-yl)-2-(4-(methoxycarbonyl)phenyl)cyclopropane-1-carboxylic acid (5a)

Following the general arylation procedure A at 100 °C and K_2CO_3 (1.0 equiv) instead of $NaHCO_3$ (eluent: hexane/ethyl acetate = 2/1 with 1% v/v of acetic acid)**.** The product of **5a** as ester was obtained (yellow oil, 17.8 mg, 47% yield)

The enantiomeric purity of the substrate was determined by SFC analysis (CHIRALPAK® AD-3 column, 20% ^{i}PrOH / CO_2, flow rate 2 mL/min, retention time 4.225 min (minor) and 6.324 min (major), 97.5:2.5 er).

^{1}H NMR (500 MHz, Chloroform-*d*) δ 8.02 (d, *J* = 8.2 Hz, 1H), 7.92 (dd, *J* = 5.4, 3.0 Hz, 1H), 7.79 (dd, *J* = 5.4, 3.0 Hz, 1H), 7.67 (d, *J* = 8.1 Hz, 1H), 3.92 (s, 1H), 3.35 (s, 1H), 3.17 (t, *J* = 9.5 Hz, 1H), 2.52 (dd, *J* = 9.2, 6.3 Hz, 0H), 1.95 (dd, *J* = 9.8, 6.3 Hz, 1H). ^{13}C NMR (151 MHz, Chloroform-*d*) δ 168.1, 168.0, 167.1, 140.1, 134.6, 131.8, 129.8, 129.5, 129.3, 123.8, 52.6, 52.2, 33.7, 29.8, 19.5. HRMS (ESI-TOF) Calculated for $C_{21}H_{18}NO_6$ $[M+H]^+$: 380.1134, Found: 380.1147.

The absolute stereochemistry was assigned based on comparing the specific rotation of **5i** with literature.

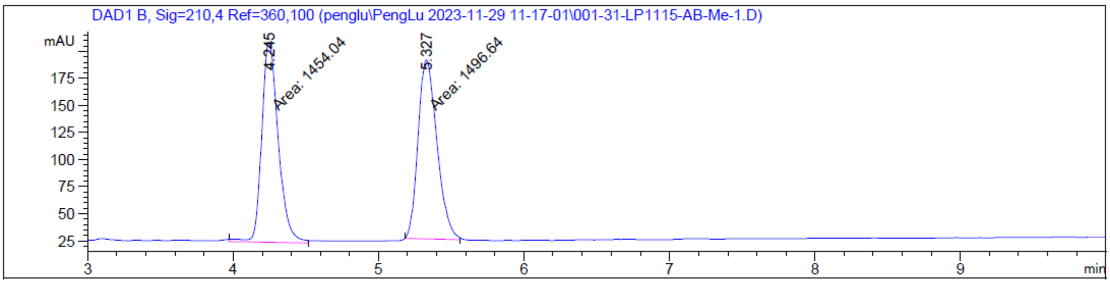

Peak #	RetTime [min]	Type	Width [min]	Area [mAU*s]	Height [mAU]	Area %
1	4.245	MM	0.1313	1454.03516	184.61369	49.2781
2	5.327	MM	0.1507	1496.63806	165.54108	50.7219

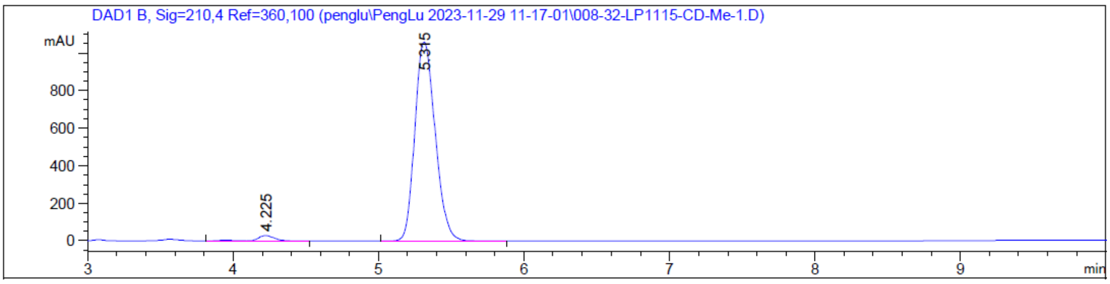

Peak #	RetTime [min]	Type	Width [min]	Area [mAU*s]	Height [mAU]	Area %
1	4.225	VB R	0.1166	258.48376	29.18375	2.4453
2	5.315	BB	0.1515	1.03122e4	1059.99475	97.5547

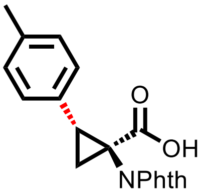

(1*S*,2*R*)-1-(1,3-dioxoisoindolin-2-yl)-2-(p-tolyl)cyclopropane-1-carboxylic acid (5b)

Following the general arylation procedure A at 100 °C and K_2CO_3 (1.0 equiv) instead of $NaHCO_3$ (eluent: hexane/ethyl acetate = 2/1 with 1% v/v of acetic acid)**.** The product of **5b** as acid was obtained (yellow oil, 12.8 mg, 40% yield)

The enantiomeric purity of the substrate was determined by SFC analysis (CHIRALPAK® AD-3 column, 20% ^{i}PrOH / CO_2, flow rate 2 mL/min, retention time 5.879 min (minor) and 6.600 min (major), 99.5:0.5 er).

^{1}H NMR (600 MHz, Chloroform-*d*) δ 7.88 (dd, *J* = 5.4, 3.1 Hz, 2H), 7.75 (dd, *J* = 5.4, 3.0 Hz, 2H), 7.43 (d, *J* = 7.8 Hz, 2H), 7.13 (d, *J* = 7.8 Hz, 2H), 3.14 (s, 1H), 2.42 (dd, *J* = 9.4, 6.1 Hz, 1H), 2.35 (s, 3H), 1.90 (dd, *J* = 9.9, 6.1 Hz, 1H). The NMR data matches the reported data (5). HRMS (ESI-TOF) Calculated for $C_{19}H_{16}NO_4$ $[M+H]^+$: 322.1079, Found: 322.1088.

The absolute stereochemistry was assigned based on comparing the specific rotation of **5i** with literature.

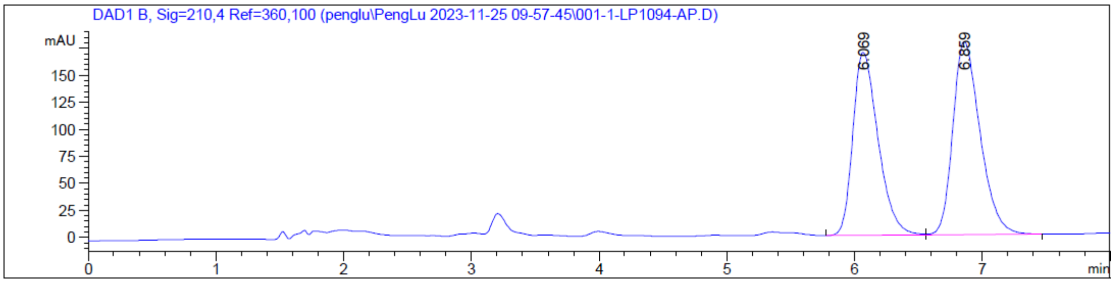

Peak #	RetTime [min]	Type	Width [min]	Area [mAU*s]	Height [mAU]	Area %
1	6.069	BV	0.2102	2323.38159	169.19200	46.7790
2	6.859	VB	0.2288	2643.33936	178.51186	53.2210

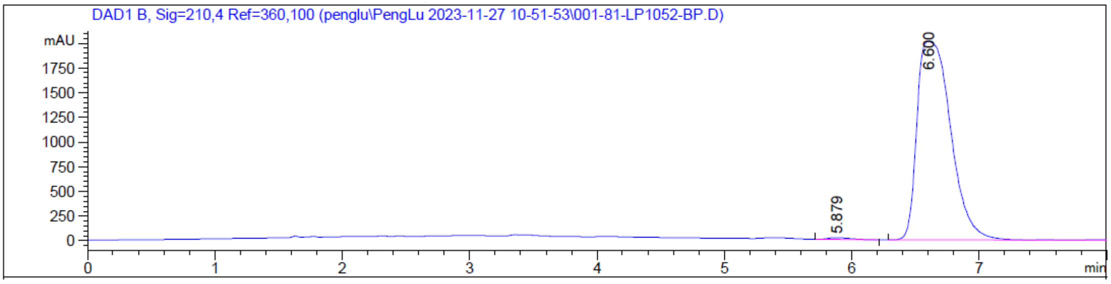

Peak #	RetTime [min]	Type	Width [min]	Area [mAU*s]	Height [mAU]	Area %
1	5.879	BB	0.1817	212.63039	18.28376	0.5695
2	6.600	BV R	0.2903	3.70794e4	2008.85657	99.3196
3	9.040	BB	0.2312	41.38107	2.20270	0.1108

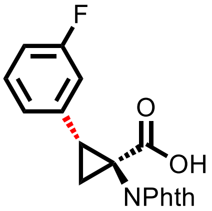

(1*S*,2*R*)-1-(1,3-dioxoisoindolin-2-yl)-2-(3-fluorophenyl)cyclopropane-1-carboxylic acid (5c)

Following the general arylation procedure A at 100 °C and K_2CO_3 (1.0 equiv) instead of $NaHCO_3$ (eluent: hexane/ethyl acetate = 2/1 with 1% v/v of acetic acid)**.** The product of **5c** as acid was obtained (yellow oil, 9.7 mg, 30% yield)

The enantiomeric purity of the substrate was determined by SFC analysis (CHIRALPAK® IC-3 column, 20% ^{i}PrOH / CO_2, flow rate 2 mL/min, retention time 3.333 min (minor) and 3.849 min (major), 97.5:2.5 er).

^{1}H NMR (600 MHz, Chloroform-*d*) δ 7.88 (dd, *J* = 5.4, 3.0 Hz, 2H), 7.75 (dd, *J* = 5.4, 3.0 Hz, 2H), 7.37 – 7.33 (m, 1H), 7.30 – 7.26 (m, 2H), 6.96 – 6.90 (m, 1H), 3.14 (t, *J* = 9.6 Hz, 1H), 2.40 (dd, *J* = 9.2, 6.3 Hz, 1H), 1.92 (dd, *J* = 9.7, 6.2 Hz, 1H). ^{13}C NMR (151 MHz, Chloroform-*d*) δ173.1, 168.0, 162.7 (d, *J* = 246.1 Hz), 136.7, 136.6, 134.6, 131.7, 129.8 (d, *J* = 7.6 Hz), 125.5, 123.8, 116.7 (d, *J* = 22.7 Hz), 114.6 (d, *J* = 21.1 Hz) 37.7, 32.0, 29.8. 20.1 ^{19}F NMR (376 MHz, Chloroform-*d*) δ -116.27. HRMS (ESI-TOF) Calculated for $C_{18}H_{11}FNO_4$ $[M-H]^-$: 324.0672, Found: 324.0674.

The absolute stereochemistry was assigned based on comparing the specific rotation of **5i** with literature.

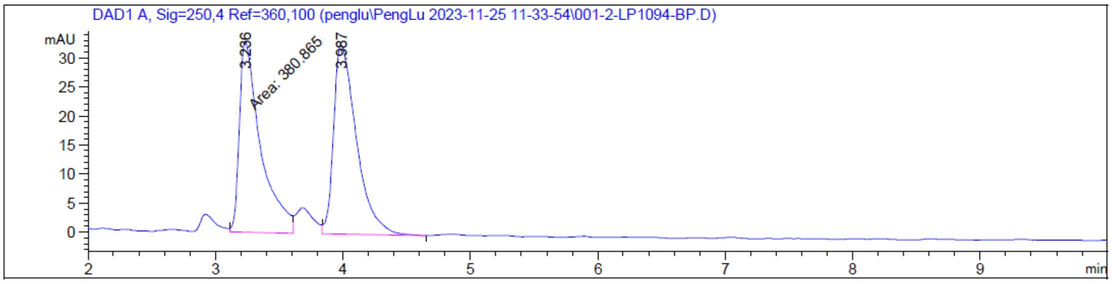

Peak #	RetTime [min]	Type	Width [min]	Area [mAU*s]	Height [mAU]	Area %
1	3.236	MF	0.1930	380.86530	32.89609	48.2837
2	3.987	VB	0.1903	407.94235	32.53595	51.7163

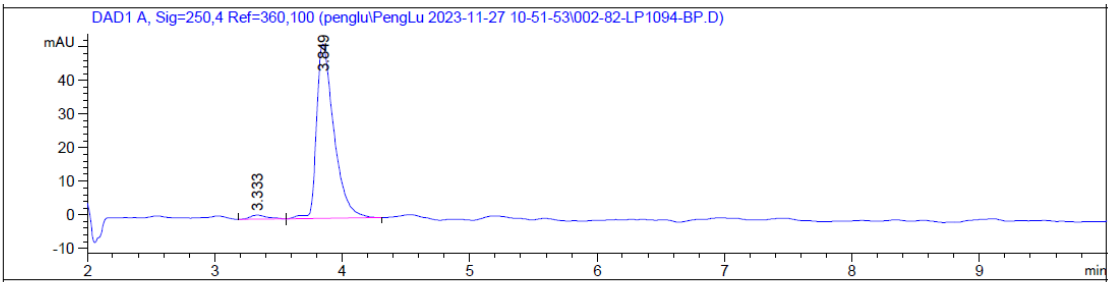

Peak #	RetTime [min]	Type	Width [min]	Area [mAU*s]	Height [mAU]	Area %
1	3.333	BB	0.1324	11.85631	1.20942	2.3615
2	3.849	BB	0.1420	490.20306	51.98320	97.6385

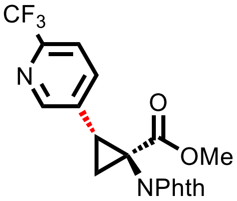

methyl (1*S*,2*R*)-1-(1,3-dioxoisoindolin-2-yl)-2-(6-(trifluoromethyl)pyridin-3-yl)cyclopropane-1-carboxylate (5d)

Following the general arylation procedure B (eluent: hexane/ethyl acetate = 2/1 with 1% v/v of acetic acid). The product of **5d** as ester was obtained (yellow oil, 11.0 mg, 28% yield).

The enantiomeric purity of the substrate was determined by SFC analysis (CHIRALPAK® AD-3 column, 20% ^{i}PrOH / CO_2, flow rate 2 mL/min, retention time 9.422 min (minor) and 11.552 min (major), 2:98 er).

^{1}H NMR (600 MHz, Chloroform-*d*) δ 8.93 (s, 1H), 8.09 (d, *J* = 7.8 Hz, 1H), 7.96 – 7.89 (m, 3H), 7.82 – 7.78 (m, 2H), 7.67 (d, *J* = 8.0 Hz, 1H), 3.43 (s, 3H), 3.11 (t, *J* = 9.4 Hz, 1H), 2.58 – 2.50 (m, 1H), 2.06 (dd, *J* = 9.7, 6.5 Hz, 1H). ^{13}C NMR (151 MHz, Chloroform-*d*) δ 168.0, 167.7, 151.5, 138.7, 134.7, 133.8, 131.7, 123.9, 119.8 (q, *J* = 1.5 Hz), 53.0, 31.1, 29.8, 19.3. ^{19}F NMR (376 MHz, $CDCl_3$) δ -70.41. HRMS (ESI-TOF) Calculated for $C_{19}H_{14}F_3N_2O_4$ $[M+H]^+$: 391.0906, Found: 391.0908.

The absolute stereochemistry was assigned based on comparing the specific rotation of **5i** with literature.

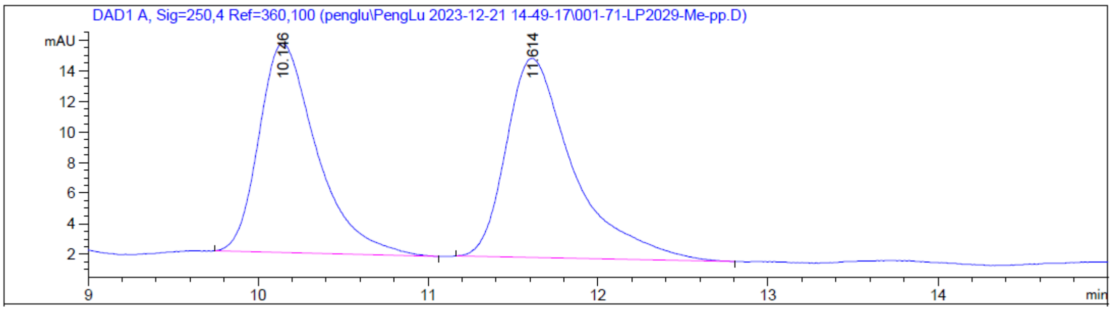

Peak #	RetTime [min]	Type	Width [min]	Area [mAU*s]	Height [mAU]	Area %
1	10.146	BB	0.3292	323.00128	13.69174	47.4042
2	11.614	BB	0.3771	358.37631	13.02291	52.5958

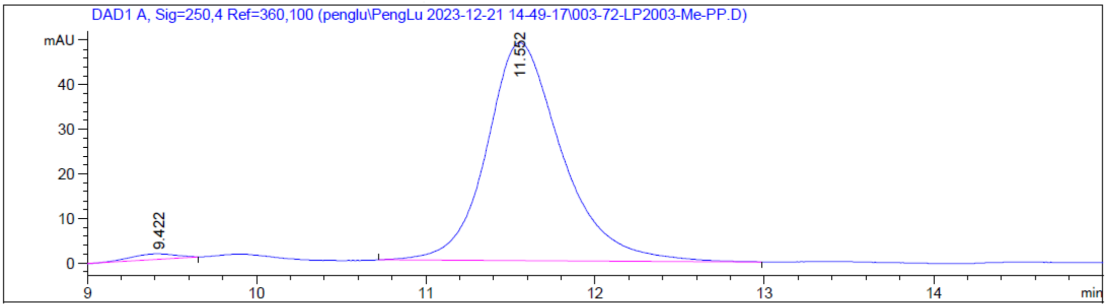

Peak #	RetTime [min]	Type	Width [min]	Area [mAU*s]	Height [mAU]	Area %
1	9.422	BB	0.2320	24.07146	1.23156	1.5606
2	11.552	BB	0.4476	1518.34753	48.98997	98.4394

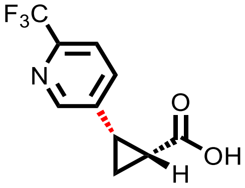

(1*R*,2*S*)-2-(6-(trifluoromethyl)pyridin-3-yl)cyclopropane-1-carboxylic acid (5e)

Following the general arylation procedure B (eluent: hexane/ethyl acetate = 2/1 with 1% v/v of acetic acid). The product of **5e** as acid was obtained (yellow oil, 15.7 mg, 68% yield).

The enantiomeric purity of the substrate was determined by SFC analysis (CHIRALPAK® IC-3 column, 5% ^{i}PrOH / CO_2, flow rate 2 mL/min, retention time 4.610 min (major) and 6.868 min (minor), 99:1 er).

^{1}H NMR (600 MHz, $CDCl_3$) δ 8.63 (s, 1H), 7.74 (d, *J* = 7.8 Hz, 1H), 7.61 (d, *J* = 8.2 Hz, 1H), 2.65 (q, *J* = 8.5 Hz, 1H), 2.20 (q, *J* = 8.4 Hz, 1H), 1.80 – 1.69 (m, 1H), 1.55 (td, *J* = 8.2, 5.4 Hz, 1H). ^{13}C NMR (150 MHz, $CDCl_3$) δ 175.80, 151.09, 146.38 (q, *J* = 34.5 Hz), 138.17, 135.53, 121.70 (q, *J* =273.0 Hz), 119.82 (q, *J* = 3.0 Hz), 23.43, 21.58, 12.24. ^{19}F NMR (376 MHz, $CDCl_3$) δ -71.19. HRMS (ESI-TOF) Calcd for $C_{10}H_9F_3NO_2$ $[M+H]^+$:232.0585; found: 232.0588.

The absolute stereochemistry was assigned based on comparing the specific rotation of **5i** with literature.

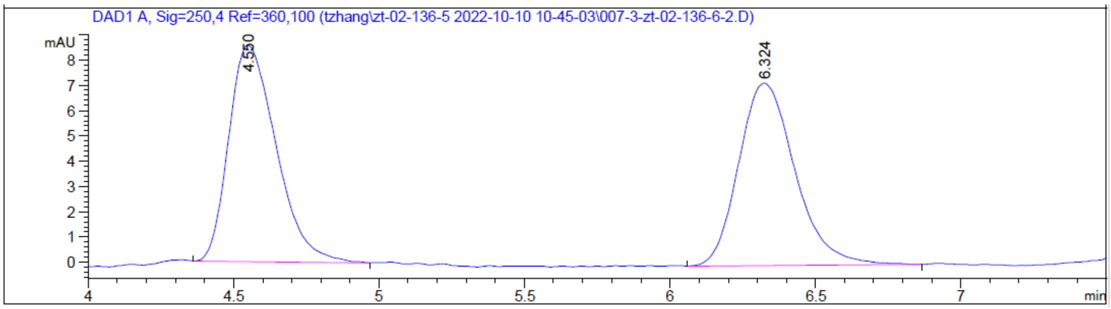

Peak #	RetTime [min]	Type	Width [min]	Area [mAU*s]	Height [mAU]	Area %
1	4.550	BB	0.1796	96.65871	8.56707	49.8654
2	6.324	BB	0.2091	97.18056	7.21503	50.1346

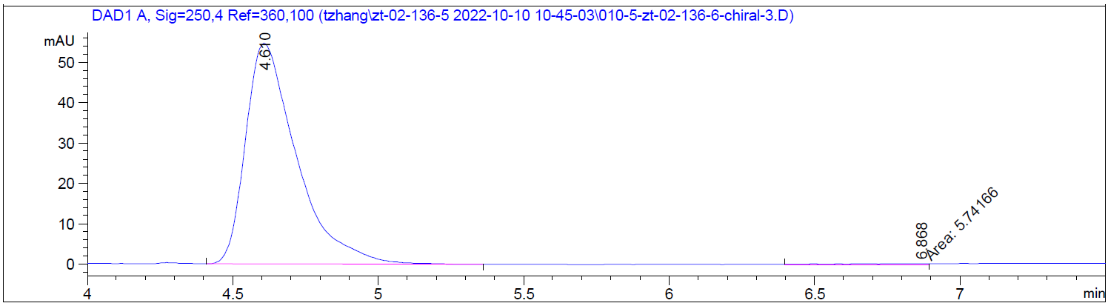

Peak #	RetTime [min]	Type	Width [min]	Area [mAU*s]	Height [mAU]	Area %
1	4.610	BB	0.1778	664.87482	54.69303	99.1438
2	6.868	MM	0.4126	5.74166	2.31952e-1	0.8562

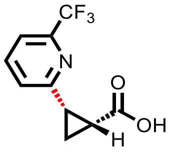

(1*R*,2*S*)-2-(6-(trifluoromethyl)pyridin-2-yl)cyclopropane-1-carboxylic acid (5f)

Following the general arylation procedure B (eluent: hexane/ethyl acetate = 2/1 with 1% v/v of acetic acid). The product of **5f** as acid was obtained (white solid, 12.7 mg, 55% yield).

The enantiomeric purity of the substrate was determined by SFC analysis (CHIRALPAK® OJ-3 column, 5% ^{i}PrOH / CO_2, flow rate 2 mL/min, retention time 3.182 min (major) and 4.130 min (minor), 97:3 er).

^{1}H NMR (600 MHz, $CDCl_3$) δ 7.82 (t, *J* = 7.8 Hz, 1H), 7.54 (d, *J* = 7.8 Hz, 1H), 7.50 (d, *J* = 7.8 Hz, 1H), 2.72 (q, *J* = 8.7 Hz, 1H), 2.25 (td, *J* = 8.7, 6.5 Hz, 1H), 1.85 – 1.79 (m, 1H), 1.64 (td, *J* = 8.6, 5.3 Hz, 1H). ^{13}C NMR (150 MHz, $CDCl_3$) δ 174.77, 158.23, 147.16 (q, *J* = 34.5), 138.10, 126.95, 121.31 (q, *J* = 273.0 Hz), 118.64 (q, *J* = 3.0), 26.99, 23.59, 14.85. ^{19}F NMR (376 MHz, $CDCl_3$) δ -70.69. HRMS (ESI-TOF) Calcd for $C_{10}H_9F_3NO_2$ $[M+H]^+$: 232.0585; found: 232.0591.

The absolute stereochemistry was assigned based on comparing the specific rotation of **5i** with literature.

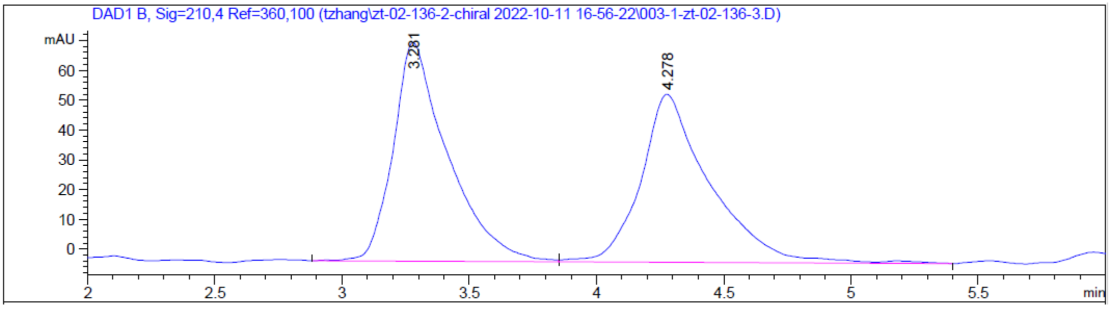

Peak #	RetTime [min]	Type	Width [min]	Area [mAU*s]	Height [mAU]	Area %
1	3.281	VV R	0.2060	1096.74854	73.51857	50.6121
2	4.278	VV R	0.2570	1070.22070	56.48640	49.3879

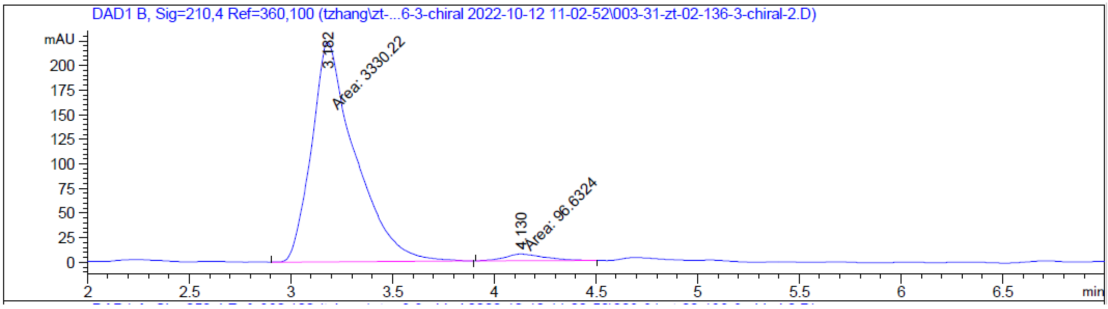

Peak #	RetTime [min]	Type	Width [min]	Area [mAU*s]	Height [mAU]	Area %
1	3.182	MM	0.2483	3330.22095	223.49767	97.1801
2	4.130	MM	0.2311	96.63236	6.96832	2.8199

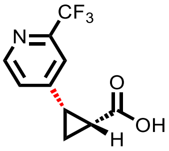

(1*R*,2*S*)-2-(2-(trifluoromethyl)pyridin-4-yl)cyclopropane-1-carboxylic acid (5g)

Following the general arylation procedure B (eluent: hexane/ethyl acetate = 2/1 with 1% v/v of acetic acid). The product of **5g** as acid was obtained (yellow oil, 15.5 mg, 67% yield).

The enantiomeric purity of the substrate was determined by SFC analysis (CHIRALPAK® AD-3 column, 5% ^{i}PrOH / CO_2, flow rate 2 mL/min, retention time 5.771 min (major) and 8.463 min (minor), 95:5 er).

^{1}H NMR (600 MHz, $CDCl_3$) δ 8.57 (d, *J* = 4.8 Hz, 1H), 7.55 (s, 1H), 7.35 (d, *J* = 4.6

Hz, 1H), 2.61 (q, J = 8.4 Hz, 1H), 2.21 – 2.08 (m, 1H), 1.75 (dt, J = 7.6, 5.4 Hz, 1H), 1.52 (td, J = 8.2, 5.2 Hz, 1H). ^{13}C NMR (150 MHz, $CDCl_3$) δ 175.17, 149.46, 147.84, 147.78 (q, J = 33.0 Hz), 127.29, 121.62 (q, J = 271.5 Hz), 121.44 (q, J = 3.0 Hz), 25.27, 22.35, 12.33. ^{19}F NMR (376 MHz, $CDCl_3$) δ -70.63. HRMS (ESI-TOF) Calcd for $C_{10}H_9F_3NO_2$ $[M+H]^+$: 232.0585; found: 232.0592.

The absolute stereochemistry was assigned based on comparing the specific rotation of **5i** with literature.

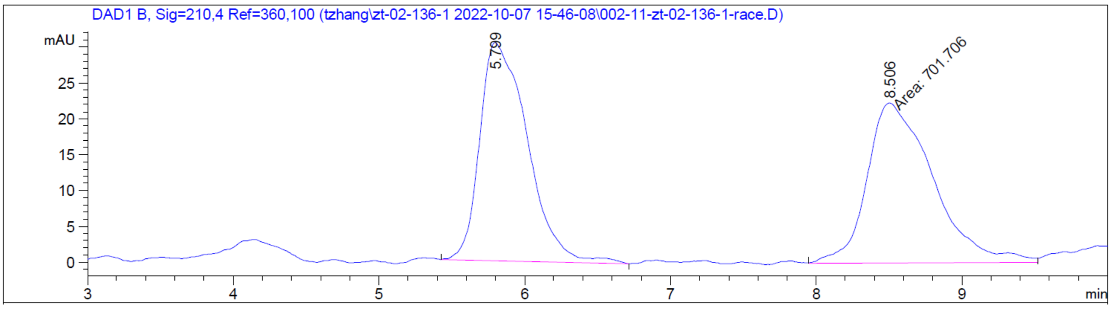

Peak #	RetTime [min]	Type	Width [min]	Area [mAU*s]	Height [mAU]	Area %
1	5.799	BB	0.3116	692.83392	30.43011	49.6819
2	8.506	MM	0.5246	701.70630	22.29336	50.3181

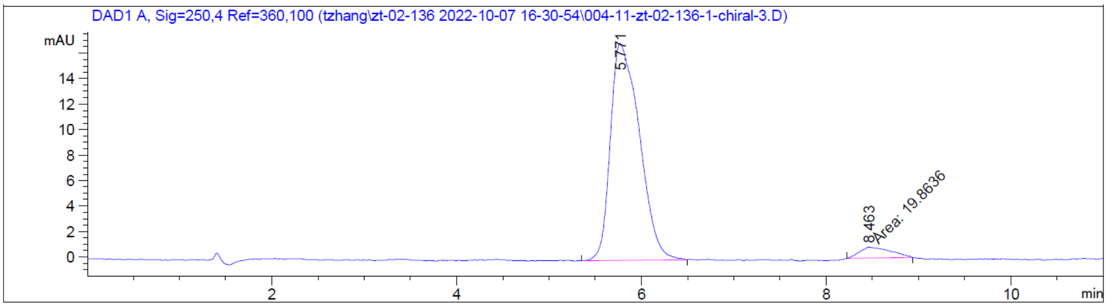

Peak #	RetTime [min]	Type	Width [min]	Area [mAU*s]	Height [mAU]	Area %
1	5.771	BB	0.3021	370.97205	17.03364	94.9177
2	8.463	MM	0.3850	19.86357	8.59808e-1	5.0823

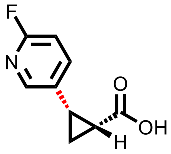

(1*R*,2*S*)-2-(6-fluoropyridin-3-yl)cyclopropane-1-carboxylic acid (5h)

Following the general arylation procedure B (eluent: hexane/ethyl acetate = 2/1 with 1% v/v of acetic acid). The product of **5h** as acid was obtained (yellow oil, 9.8 mg, 54% yield).

The enantiomeric purity of the substrate was determined by SFC analysis (CHIRALPAK® As-3 column, 5% ^{i}PrOH / CO_2, flow rate 2 mL/min, retention time 5.365 min (minor) and 6334 min (major), 98.5:1.5 er).

^{1}H NMR (600 MHz, $CDCl_3$) δ 8.11 (d, *J* = 1.8 Hz, 1H), 7.66 (td, *J* = 8.0, 2.4 Hz, 1H), 6.85 (dd, *J* = 8.4, 2.8 Hz, 1H), 2.57 (q, *J* = 8.6 Hz, 1H), 2.11 (td, *J* = 8.4, 5.6 Hz, 1H), 1.66 (dt, *J* = 7.4, 5.4 Hz, 1H), 1.48 (td, *J* = 8.2, 5.2 Hz, 1H). ^{13}C NMR (150 MHz, $CDCl_3$) δ 176.18, 162.68 (d, *J* = 238.6 Hz), 148.33 (d, *J* = 15.0 Hz), 142.14 (d, *J* = 9.0 Hz), 129.64 (d, *J* = 4.5 Hz), 108.78 (d, *J* = 36.0 Hz), 22.84, 21.18, 12.18. ^{19}F NMR (376 MHz, $CDCl_3$) δ -73.84. HRMS (ESI-TOF) Calcd for $C_9H_9FNO_2$ $[M+H]^+$:182.0617; found: 182.0614.

The absolute stereochemistry was assigned based on comparing the specific rotation of **5i** with literature.

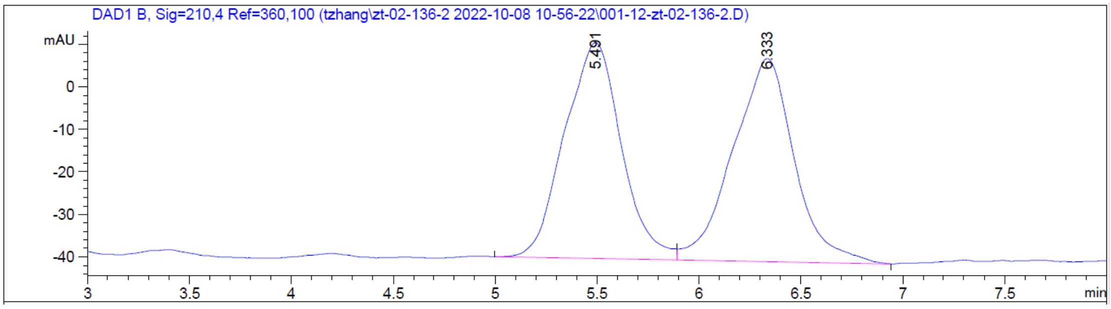

Peak #	RetTime [min]	Type	Width [min]	Area [mAU*s]	Height [mAU]	Area %
1	5.491	BV	0.2747	987.28021	50.81583	49.6521
2	6.333	VB	0.2924	1001.11621	47.79427	50.3479

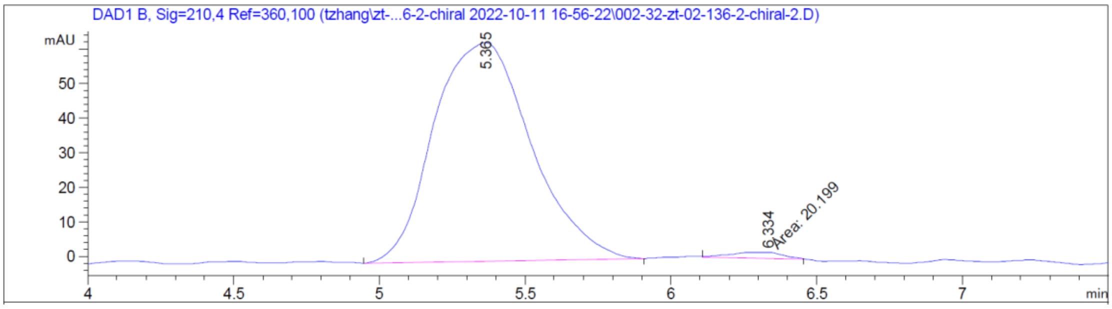

Peak #	RetTime [min]	Type	Width [min]	Area [mAU*s]	Height [mAU]	Area %
1	5.365	BB	0.3734	1470.46863	63.20741	98.6450
2	6.334	MM	0.2051	20.19899	1.64160	1.3550

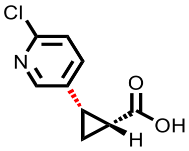

(1*R*,2*S*)-2-(6-chloropyridin-3-yl)cyclopropane-1-carboxylic acid (5i)

Following the general arylation procedure B (eluent: hexane/ethyl acetate = 2/1 with 1% v/v of acetic acid). The product of **5i** as acid was obtained (yellow oil, 7.5 mg, 38% yield).

The enantiomeric purity of the substrate was determined by SFC analysis (CHIRALPAK® OJ-3 column, 5% ^{i}PrOH / CO_2, flow rate 2 mL/min, retention time 4.571 min (major) and 6.505 min (minor), 99:1 er).

^{1}H NMR (400 MHz, $CDCl_3$) δ 8.23 (d, *J* = 5.2 Hz, 1H), 7.22 (s, 1H), 7.13 – 7.05 (m, 1H), 2.52 (q, *J* = 8.5 Hz, 1H), 2.22 – 2.10 (m, 1H), 1.70 (dt, *J* = 7.6, 5.6 Hz, 1H), 1.46 (td, *J* = 8.2, 5.4 Hz, 1H). ^{13}C NMR (150 MHz, $CDCl_3$) δ 174.83, 151.21, 149.20, 149.01, 125.17, 123.45, 24.93, 22.19, 12.14. HRMS (ESI-TOF) Calcd for $C_9H_9ClNO_2$ $[M+H]^+$:198.0322; found: 198.0325.

The absolute stereochemistry was assigned based on comparing the specific rotation of **5i** with literature.

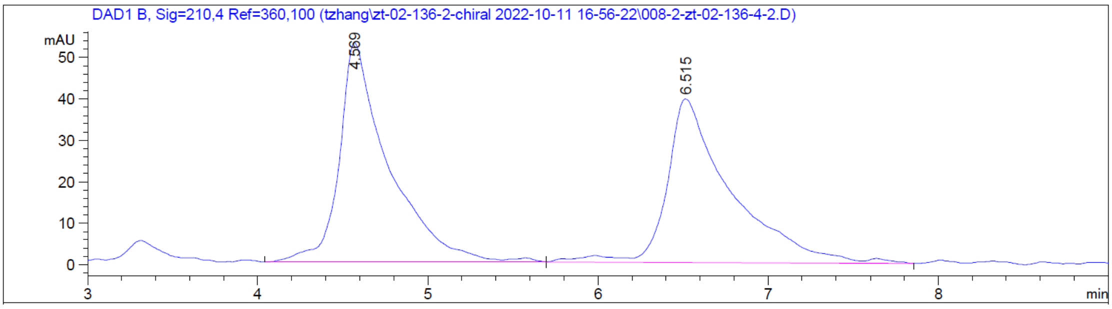

Peak #	RetTime [min]	Type	Width [min]	Area [mAU*s]	Height [mAU]	Area %
1	4.569	BV R	0.2676	1068.90576	52.93317	50.7078
2	6.515	VV R	0.3406	1039.06641	39.45735	49.2922

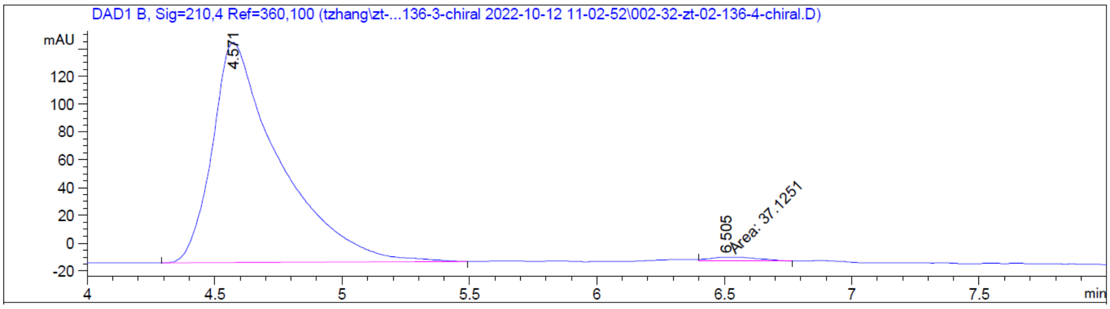

Peak #	RetTime [min]	Type	Width [min]	Area [mAU*s]	Height [mAU]	Area %
1	4.571	BB	0.2496	2903.00537	158.53218	98.7373
2	6.505	MM	0.2196	37.12506	2.81749	1.2627

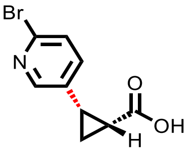

(1*R*,2*S*)-2-(6-bromopyridin-3-yl)cyclopropane-1-carboxylic acid (5j)

Following the general arylation procedure B (eluent: hexane/ethyl acetate = 2/1 with 1% v/v of acetic acid). The product of **5j** as acid was obtained (yellow oil, 6.7 mg, 28% yield).

The enantiomeric purity of the substrate was determined by SFC analysis (CHIRALPAK® OJ-3 column, 5% ^{i}PrOH / CO_2, flow rate 2 mL/min, retention time 5.989 min (major) and 8.926 min (minor), 96:4 er).

^{1}H NMR (400 MHz, $CDCl_3$) δ 8.21 (d, *J* = 5.2 Hz, 1H), 7.39 (s, 1H), 7.16 – 7.01 (m, 1H), 2.50 (q, *J* = 8.5 Hz, 1H), 2.23 – 2.10 (m, 1H), 1.69 (dt, *J* = 7.4, 5.6 Hz, 1H), 1.46 (td, *J* = 8.2, 5.4 Hz, 1H). ^{13}C NMR (150 MHz, $CDCl_3$) δ 174.71, 149.46, 148.95, 141.83, 128.97, 123.82, 24.81, 22.21, 12.14. HRMS (ESI-TOF) Calcd for $C_9H_2BrNO_2$ $[M-H]^-$:239.9660; found: 239.9649.

The absolute stereochemistry was assigned based on comparing the specific rotation of **5i** with literature.

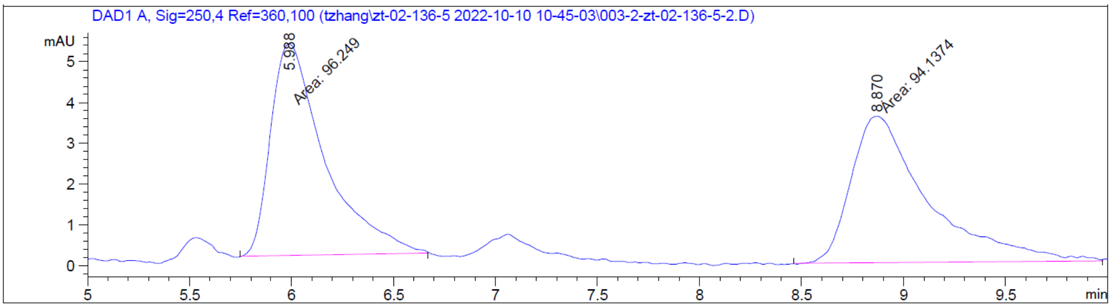

Peak #	RetTime [min]	Type	Width [min]	Area [mAU*s]	Height [mAU]	Area %
1	5.988	MM	0.3085	96.24896	5.20043	50.5545
2	8.870	MM	0.4368	94.13744	3.59216	49.4455

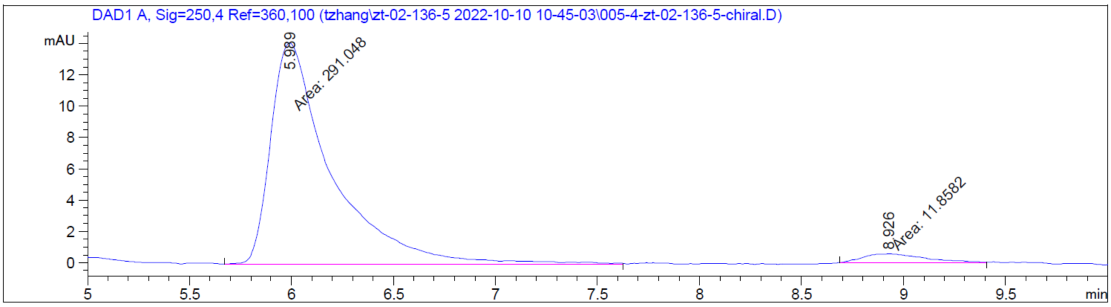

Peak #	RetTime [min]	Type	Width [min]	Area [mAU*s]	Height [mAU]	Area %
1	5.989	MM	0.3437	291.04840	14.11437	96.0852
2	8.926	MM	0.3538	11.85824	5.58583e-1	3.9148

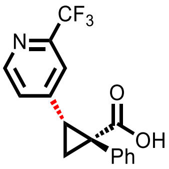

(1*R*,2*R*)-1-phenyl-2-(2-(trifluoromethyl)pyridin-4-yl)cyclopropane-1-carboxylic acid (5k)

Following the general arylation procedure B (eluent: hexane/ethyl acetate = 2/1 with 1% v/v of acetic acid). The product of **5k** as acid was obtained (yellow oil, 19.0 mg, 62% yield).

The enantiomeric purity of the substrate was determined by SFC analysis (CHIRALPAK® IC-3 column, 5% ^{i}PrOH / CO_2, flow rate 2 mL/min, retention time 7.835 min (major) and 9.558 min (minor), 98.5:1.5 er).

^{1}H NMR (600 MHz, $CDCl_3$) δ 8.61 (d, J = 5.0 Hz, 1H), 7.64 (s, 1H), 7.50 – 7.45 (m, 2H), 7.45 – 7.42 (m, 1H), 7.41 – 7.38 (m, 2H), 7.37 – 7.33 (m, 1H), 2.88 (t, J = 8.4 Hz, 1H), 2.35 (dd, J = 7.6, 5.2 Hz, 1H), 1.83 (dd, J = 8.8, 5.2 Hz, 1H). ^{13}C NMR (150 MHz, $CDCl_3$) δ 174.81, 149.58, 147.96, 147.90 (q, J = 34.5 Hz), 138.58, 130.12, 128.69, 128.14, 127.16, 121.59 (q, J = 273.0 Hz), 121.25 (q, J = 3.0 Hz), 38.77, 33.11, 19.12. ^{19}F NMR (376 MHz, $CDCl_3$) δ -70.52. HRMS (ESI-TOF) Calcd for $C_{16}H_{11}F_3NO_2$ $[M-H]^-$:239.9660; found: 239.9649.

The absolute stereochemistry was assigned based on comparing the specific rotation of **5i** with literature.

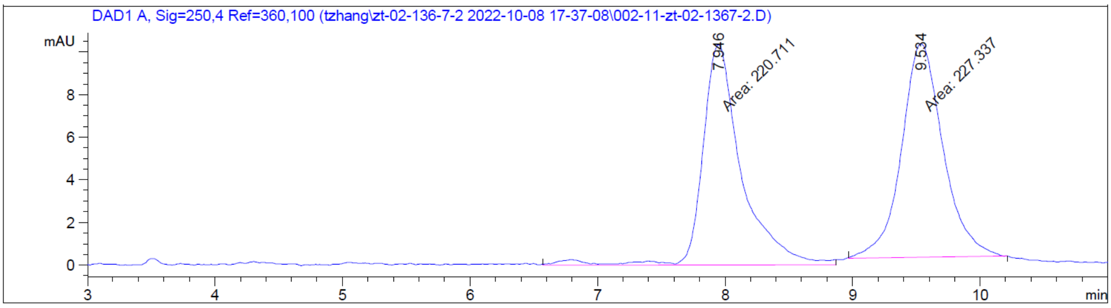

Peak #	RetTime [min]	Type	Width [min]	Area [mAU*s]	Height [mAU]	Area %
1	7.946	MM	0.3555	220.71098	10.34793	49.2605
2	9.534	MM	0.3780	227.33720	10.02421	50.7395

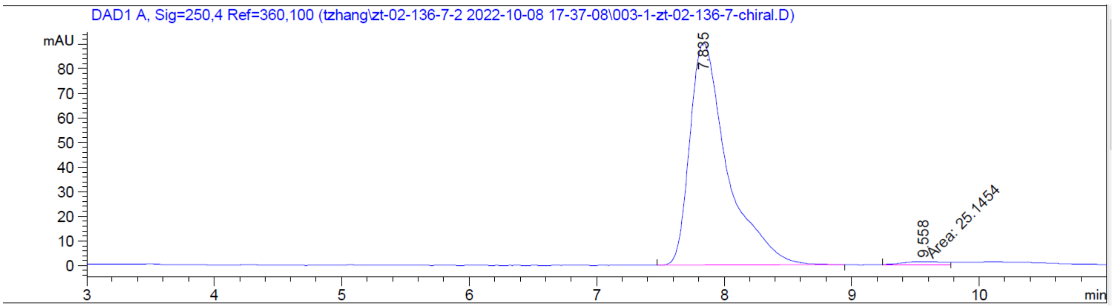

Peak #	RetTime [min]	Type	Width [min]	Area [mAU*s]	Height [mAU]	Area %
1	7.835	BB	0.3048	1848.83484	90.33101	98.6582
2	9.558	MM	0.3786	25.14544	1.10706	1.3418

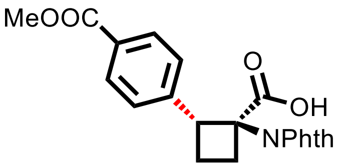

Methyl 4-((1*R*,2*S*)-2-(1,3-dioxoisoindolin-2-yl)-2-(methoxycarbonyl)cyclobutyl) benzoate (5l)

Following the general arylation procedure A at 100 °C and K_2CO_3 (1.0 equiv) instead of $NaHCO_3$ (eluent: hexane/ethyl acetate = 2/1 with 1% v/v of acetic acid)**.** The product of **5l** as ester was obtained (yellow oil, 9.4 mg, 24% yield)

The enantiomeric purity of the substrate was determined by SFC analysis (CHIRALPAK® AD-3 column, 20% ^{i}PrOH / CO_2, flow rate 2 mL/min, retention time 9.487 min (major) and 11.215 min (minor), 74:26 er).

^{1}H NMR (600 MHz, Chloroform-*d*) δ 7.98 (d, *J* = 8.3 Hz, 2H), 7.85 (dd, *J* = 5.3, 3.0 Hz, 2H), 7.74 (dd, *J* = 5.4, 3.0 Hz, 2H), 7.53 (d, *J* = 8.3 Hz, 2H), 5.11 (t, *J* = 10.1 Hz, 1H), 3.90 (s, 3H), 3.14 (t, *J* = 10.3 Hz, 1H), 2.73 (p, *J* = 10.2, 9.7 Hz, 1H), 2.56 – 2.33

(m, 2H). ^{13}C NMR (151 MHz, Chloroform-*d*) δ 168.0, 167.3, 144.9, 134.4, 131.9, 129.4, 128.8, 128.1, 128.1, 123.5, 52.2, 44.6, 31.7, 30.2, 23.0, 22.8. HRMS (ESI-TOF) Calculated for $C_{21}H_{18}NO_6$ [M+H]+: 380.1134, Found: 380.1142.

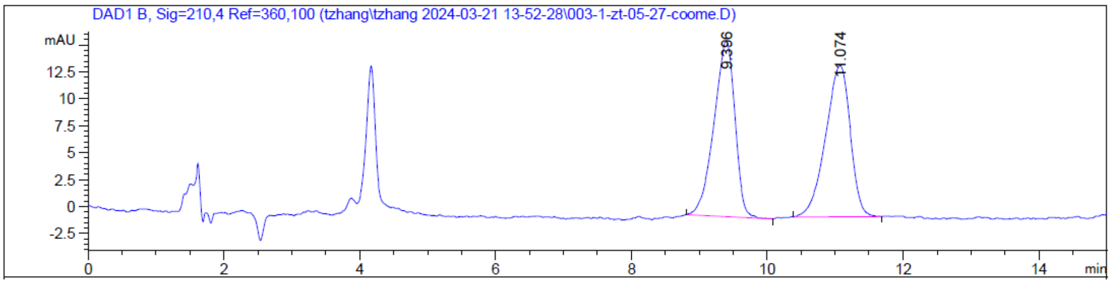

Peak #	RetTime [min]	Type	Width [min]	Area [mAU*s]	Height [mAU]	Area %
1	9.396	BB	0.3254	350.94720	16.28847	49.6532
2	11.074	BB	0.3673	355.84909	14.06066	50.3468

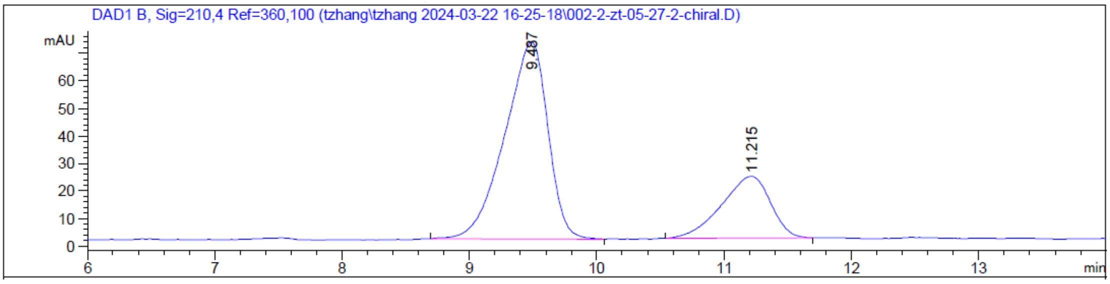

Peak #	RetTime [min]	Type	Width [min]	Area [mAU*s]	Height [mAU]	Area %
1	9.487	BB	0.3421	1681.26526	71.57260	73.6959
2	11.215	BB	0.3799	600.08936	22.44325	26.3041

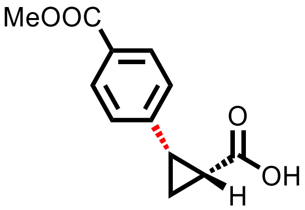

(1*R*,2*S*)-2-(4-(methoxycarbonyl)phenyl)cyclopropane-1-carboxylic acid (5m)[2]

Following the general arylation procedure B (eluent: hexane/ethyl acetate = 2/1 with 1% v/v of acetic acid). The product of **5i** as acid was obtained (white solid, 12.3 mg, 56% yield).

The enantiomeric purity of the substrate was determined by SFC analysis (CHIRALPAK® As-3 column, 30% ^{i}PrOH / CO_2, flow rate 2 mL/min, retention time 1.842 min (minor) and 2.764 min (major), 0.2:99.8 er).

^{1}H NMR (600 MHz, $CDCl_3$) δ 7.93 (d, J = 8.2 Hz, 2H), 7.31 (d, J = 8.2 Hz, 2H), 3.90 (s, 3H), 2.64 (q, J = 8.6 Hz, 1H), 2.14 – 2.08 (m, 1H), 1.71 (dt, J = 7.6, 5.4 Hz, 1H), 1.43 (td, J = 8.2, 5.2 Hz, 1H). ^{13}C NMR (150 MHz, $CDCl_3$) δ 175.1, 167.2, 141.5, 129.4, 129.4, 128.8, 52.2, 26.4, 21.7, 12.3.

The absolute stereochemistry was assigned based on comparing the specific rotation of **5i** with literature.

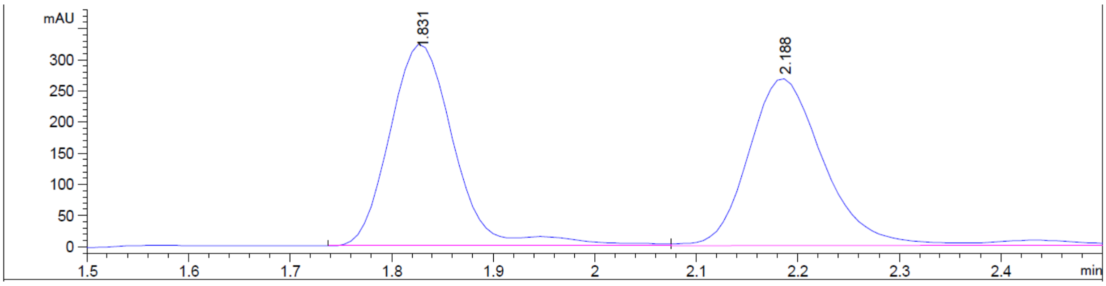

Peak #	RetTime [min]	Type	Width [min]	Area [mAU*s]	Height [mAU]	Area %
1	1.831	BV R	0.0670	1432.96143	323.97009	50.1015
2	2.188	VV R	0.0791	1427.15442	268.27094	49.8985

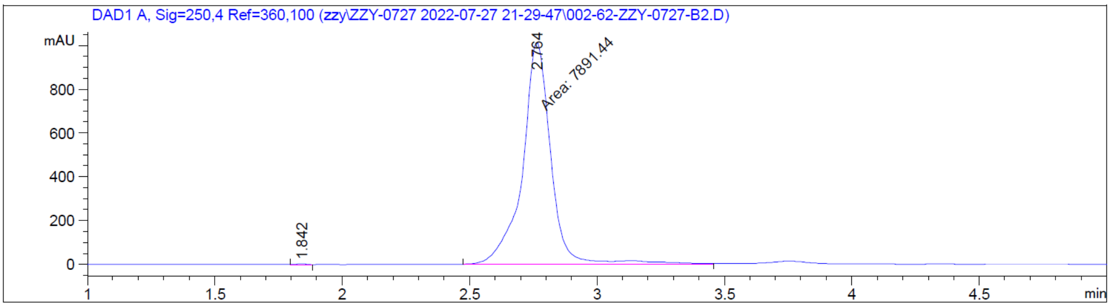

Peak #	RetTime [min]	Type	Width [min]	Area [mAU*s]	Height [mAU]	Area %
1	1.842	VB	0.0473	10.27485	3.39933	0.1300
2	2.764	MM	0.1298	7891.44434	1013.26868	99.8700

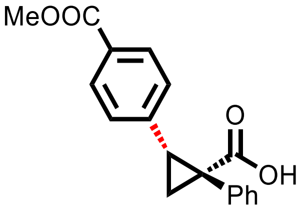

(1*R*,2*R*)-2-(4-(methoxycarbonyl)phenyl)-1-phenylcyclopropane-1-carboxylic acid (5n)[2]

Following the general arylation procedure B (eluent: hexane/ethyl acetate = 2/1 with 1% v/v of acetic acid). The product of **5j** as acid was obtained (white solid, 14.8 mg, 50% yield).

The enantiomeric purity of the substrate was determined by SFC analysis (CHIRALPAK® Ad-3 column, 20% ^{i}PrOH / CO_2, flow rate 2 mL/min, retention time 6.306 min (major) and 7.842 min (minor), 99 : 1 er).

^{1}H NMR (500 MHz, $CDCl_3$) δ 7.97 (d, *J* = 8.3 Hz, 2H), 7.48 – 7.41 (m, 2H), 7.40 – 7.33 (m, 4H), 7.33 – 7.28 (m, 1H), 3.94 (s, 3H), 2.90 (t, *J* = 8.4 Hz, 1H), 2.28 (dd, *J* = 7.8, 5.0 Hz, 1H), 1.71 (dd, *J* = 9.0, 5.0 Hz, 1H). ^{13}C NMR (150 MHz, $CDCl_3$) δ 176.0, 167.2, 141.4, 139.4, 130.3, 129.5, 129.3, 128.8, 128.5, 127.8, 52.2, 37.9, 34.5, 19.4.

The absolute stereochemistry was assigned based on comparing the specific rotation of **5i** with literature.

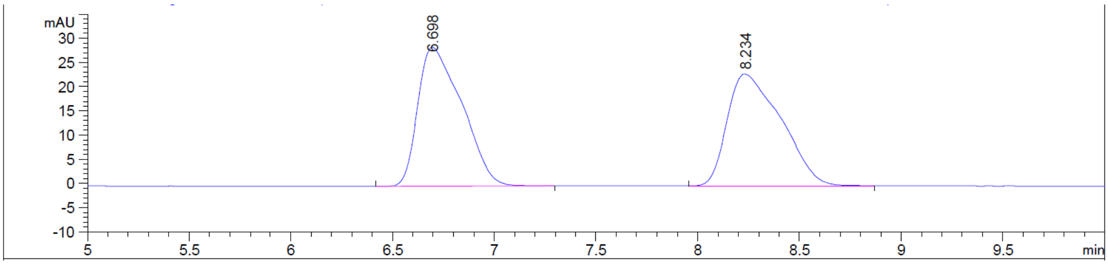

Peak #	RetTime [min]	Type	Width [min]	Area [mAU*s]	Height [mAU]	Area %
1	6.698	BB	0.2132	431.89594	28.76545	50.3701
2	8.234	BB	0.2627	425.54874	23.11917	49.6299

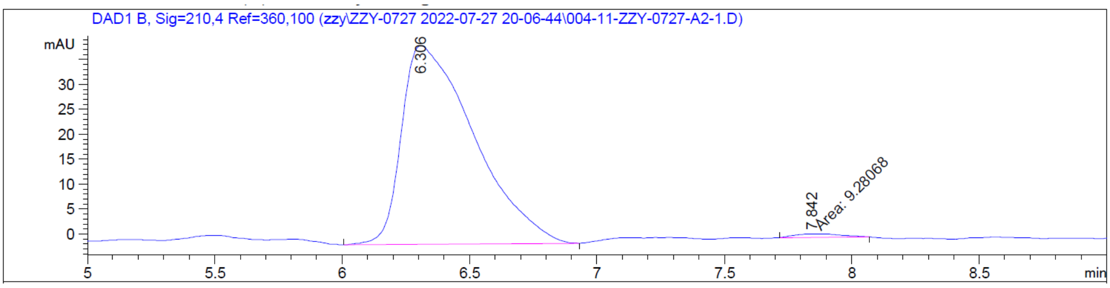

Peak #	RetTime [min]	Type	Width [min]	Area [mAU*s]	Height [mAU]	Area %
1	6.306	BB	0.2650	788.64636	39.82754	98.8369
2	7.842	MM	0.2134	9.28068	7.24885e-1	1.1631

6. Sequential diarylation of 2-Aminoisobutyric Acid

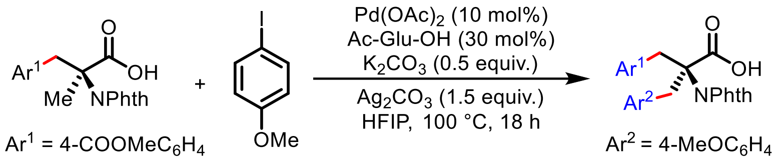

1d **6a**, 65%

$Pd(OAc)_2$ (2.2 mg, 0.01 mmol, 10 mol%), Ac-Gly-OH (3.5 mg, 0.03 mmol, 30 mol%), carboxylic acid **3a** (36.7 mg, 0.1 mmol), Ag_2CO_3 (41.1 mg, 0.15 mmol, 1.5 equiv.), K_2CO_3 (6.9 mg, 0.05 mmol, 0.5 equiv.), and 1-iodo-4-methoxybenzene (35.1 mg, 0.15 mmol, 1.5 equiv.) were weighed and placed in an 12 mL reaction tube. Subsequently, HFIP (1.0 mL) was injected, and the tube was capped and closed tightly. The reaction mixture was then stirred at 100 °C for 18 h. The mixture was allowed to cool to room temperature and acetic acid (0.05 ml) was added. Then, the mixture was passed through a pad of Celite with ethyl acetate as the eluent to remove any insoluble precipitate. The resulting solution was concentrated and the residual mixture was dissolved with minimal ethyl acetate and loaded onto a preparative TLC plate. The

pure product was then isolated using preparative TLC with ethyl acetate and hexanes (1/2) as the eluent and 1% v/v of acetic acid as the additive.

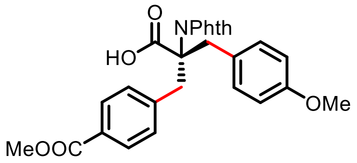

(*R*)-2-(1,3-dioxoisoindolin-2-yl)-2-(4-methoxybenzyl)-3-(4 (methoxycarbonyl) phenyl) propanoic acid (6a)

The product of **6a** as acid was obtained (white solid, 30.7 mg, 65% yield).

^{1}H NMR (600 MHz, $CDCl_3$) δ 7.87 (d, *J* = 8.1 Hz, 2H), 7.76 – 7.65 (m, 4H), 7.32 (d, *J* = 8.1 Hz, 2H), 7.18 (d, *J* = 8.5 Hz, 2H), 6.78 (d, *J* = 8.5 Hz, 2H), 3.95 (d, *J* = 14.0 Hz, 1H), 3.89 (d, *J* = 13.7 Hz, 1H), 3.86 (s, 3H), 3.74 (s, 3H), 3.46 (d, *J* = 13.8 Hz, 1H), 3.38 (d, *J* = 13.8 Hz, 1H). ^{13}C NMR (150 MHz, $CDCl_3$) δ 173.77, 168.36, 167.06, 158.93, 141.17, 134.32, 131.76, 131.30, 130.86, 129.64, 129.06, 127.01, 123.41, 113.96, 68.46, 55.25, 52.13, 39.08, 38.83. HRMS (ESI-TOF) Calcd for $C_{27}H_{23}NO_7$ $[M-H]^-$:472.1396; found: 472.1394.

7. Synthesis of Metyrosine[4]

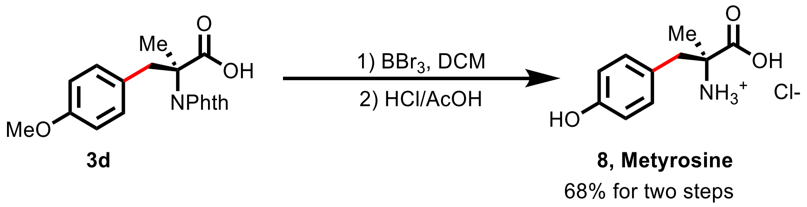

To a stirred solution of **3d** (67.8 mg, 0.2 mmol) in dry DCM (5.0 mL) was added BBr_3 (1 mmol) at 0 °C under N_2. The solution was stirred at room temperature for 2 hours and was quenched by sat. NH_4Cl (5 mL). The aqueous phase was extracted with DCM (5 mL×3) and the combined organic phase were dried with anhydrous Na_2SO_4, concentrated under reduced pressure. The residue was used without purification.

To a stirred solution of 2 mL of glacial acetic acid and 4 mL of 5M HCl was added the substrate. The reaction was then heated to reflux with stirring for 4 hours, then cooled to room temperature and concentrated to dryness under reduced pressure. The crude solid was washed with 3:1 ether: ethyl acetate (5 mL×3) and then dried in vacuum to afford pure amino acid hydrochloride as a fine white powder.

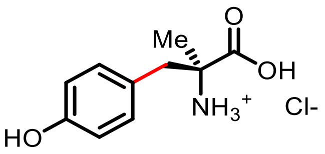

(*S*)-2-carboxy-1-(4-hydroxyphenyl)propan-2-aminium chloride[5]

White solid (31.4 mg, 68% yield for two steps). ^{1}H NMR (600 MHz, D_2O) δ 7.12 (d, *J* = 8.4 Hz, 2H), 6.86 (d, *J* = 8.4 Hz, 2H), 3.26 (d, *J* = 14.6 Hz, 1H), 2.98 (d, *J* = 14.6 Hz, 1H), 1.58 (s, 3H). ^{13}C NMR (150 MHz, D_2O) δ 174.24, 155.25, 131.45, 125.07, 115.78, 61.11, 41.53, 21.60.

8. Synthesis of BIRT-377

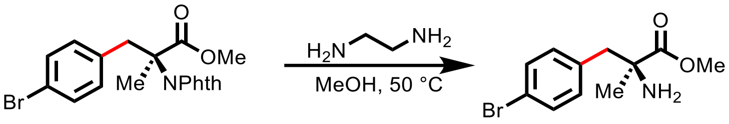

To a stirred solution of Methyl ester of **3k** (80.2 mg, 0.2 mmol) in MeOH (1.0 mL) was added ethylenediamine (72 mg, 1.2 mmol) at 0 ℃ under N_2. The solution was stirred at 50 ℃ for 12 hours and was quenched by sat. NH_4Cl (5 mL). The solvents were removed under reduced pressure and the residue was purified by silica gel column chromatography to afford the desired product.

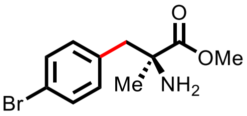

Methyl (*S*)-2-amino-3-(4-bromophenyl)-2-methylpropanoate[6]

Colorless oil (45.0 mg, 83%). ^{1}H NMR (600 MHz, $CDCl_3$) δ 7.40 (d, *J* = 7.0 Hz, 2H), 7.03 (d, *J* = 7.0 Hz, 2H), 3.70 (s, 3H), 3.06 (d, *J* = 13.2 Hz, 1H), 2.75 (d, *J* = 13.2 Hz, 1H), 1.37 (s, 3H). ^{13}C NMR (150 MHz, $CDCl_3$) δ 177.41, 135.67, 131.76, 131.53, 121.13, 58.79, 52.29, 46.33, 26.66.

9. Synthesis of Boc-amino acid

To a stirred solution of Methyl ester of **3k** (80.2 mg, 0.2 mmol) in MeOH (1.0 mL) was added ethylenediamine (72 mg, 1.2 mmol) at 0 ℃ under N_2. The solution was stirred at room temperature for 24 hours and was quenched by sat. NH_4Cl (5 mL). The solvents were removed under reduced pressure and the residue was purified by silica gel column chromatography to afford the desired product **9** (31.6 mg, 58%).

To a stirred solution of **9** (27.1 mg, 0.1 mmol) in DCM (1.0 mL) was added TEA (20.2 mg, 0.2 mmol), and Boc_2O (43.6 mg, 0.2 mmol) at 0 ℃ under N_2. The solution was stirred at room temperature for 12 hours and was quenched by sat. NH_4Cl (5 mL). The solvents were removed under reduced pressure and the residue was purified by silica gel column chromatography to afford the desired product **10** (31.5 mg, 86%).

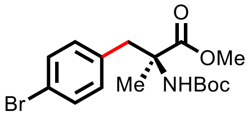

Methyl (*S*)-3-(4-bromophenyl)-2-((tert-butoxycarbonyl)amino)-2-methylpropanoate[7]

^{1}H NMR (600 MHz, Chloroform-*d*) δ 7.39 (d, *J* = 8.3 Hz, 2H), 6.94 (d, *J* = 8.3 Hz, 2H), 5.13 (s, 1H), 3.76 (s, 3H), 3.38 (d, *J* = 12.6 Hz, 1H), 3.17 (d, *J* = 13.6 Hz, 1H), 1.56 (s, 3H), 1.47 (s, 9H). ^{13}C NMR (151 MHz, Chloroform-*d*) δ 174.3, 154.4, 135.6, 131.8, 131.4, 121.0, 60.4, 52.7, 40.9, 28.5, 23.9.

10. Reference

1. Ishibashi, H.; Uegaki, M.; Sakai, M.; Takeda, Y. Base-promoted aminoethylation of thiols with 2-oxazolidinones: a simple synthesis of 2-aminoethyl sulfides. *Tetrahedron* **2001**, 57, 2115–2120.
2. Shen, P.-X.; Hu, L.; Shao, Q.; Hong, K.; Yu, J.-Q. Pd(II)-Catalyzed Enantioselective C(sp^3)–H Arylation of Free Carboxylic Acids. *J. Am. Chem. Soc.* **2018**, *140*, 6545–6549.
3. Alford, J. S.; Davies, H. M. L. Expanding the Scope of Donor/ Acceptor Carbenes to N-Phthalimido Donor Groups: Diastereoselective Synthesis of 1-Cyclopropane α-Amino Acids. *Org. Lett.* **2012**, 14, 6020−6023.
4. Chenault, H. K.; Dahmer, J.; Whitesides, G. M. Kinetic Resolution of Unnatural and Rarely Occurring Amino Acids: Enantioselective Hydrolysis of *N*-Acyl Amino Acids Catalyzed by Acylase I. *J. Am. Chem. Soc.* **1989**, *111*, 6354– 6364.
5. Chen, G.; Shigenari, T.; Jain, P.; Zhang, Z.; Jin, Z.; He, J.; Li, S.; Mapelli, C.; Miller, M. M.; Poss, M. A. et al. Ligand-Enabled *β*-C-H Arylation of Alpha-Amino Acids Using a Simple and Practical Auxiliary. *J. Am. Chem.*

Soc. **2015**, *137*, 3338– 3351.

6. Chowdari, N. S.; Barbas, C. F. Total Synthesis of LFA-1 Antagonist BIRT-377 via Organocatalytic Asymmetric Construction of a Quaternary Stereocenter. *Org. Lett.* **2005**, *7*, 867–870.
7. Chen, G.; Zhuang, Z.; Li, G.-C.; Saint-Denis, T. G.; Hsiao, Y.; Joe, C. L.; Yu, J.-Q. Ligand-Enabled β-C–H Arylation of α-Amino Acids Without Installing Exogenous Directing Groups. *Angew. Chem., Int. Ed.* **2017**, *56*, 1506–1509.

11. NMR

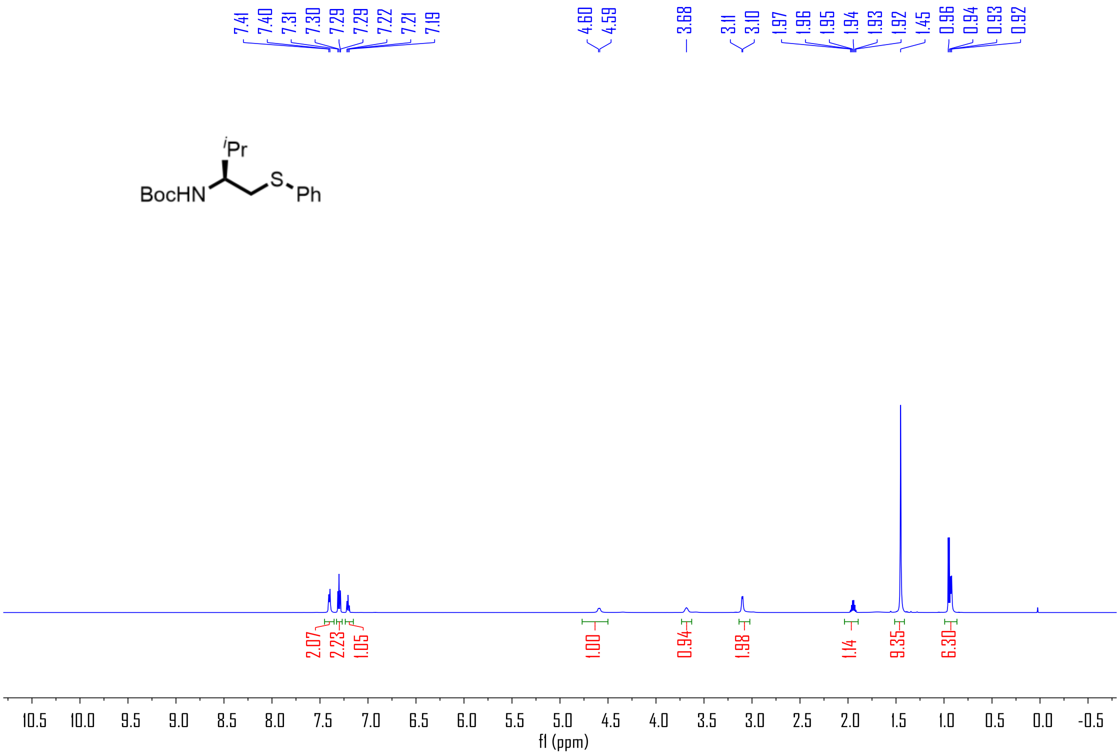

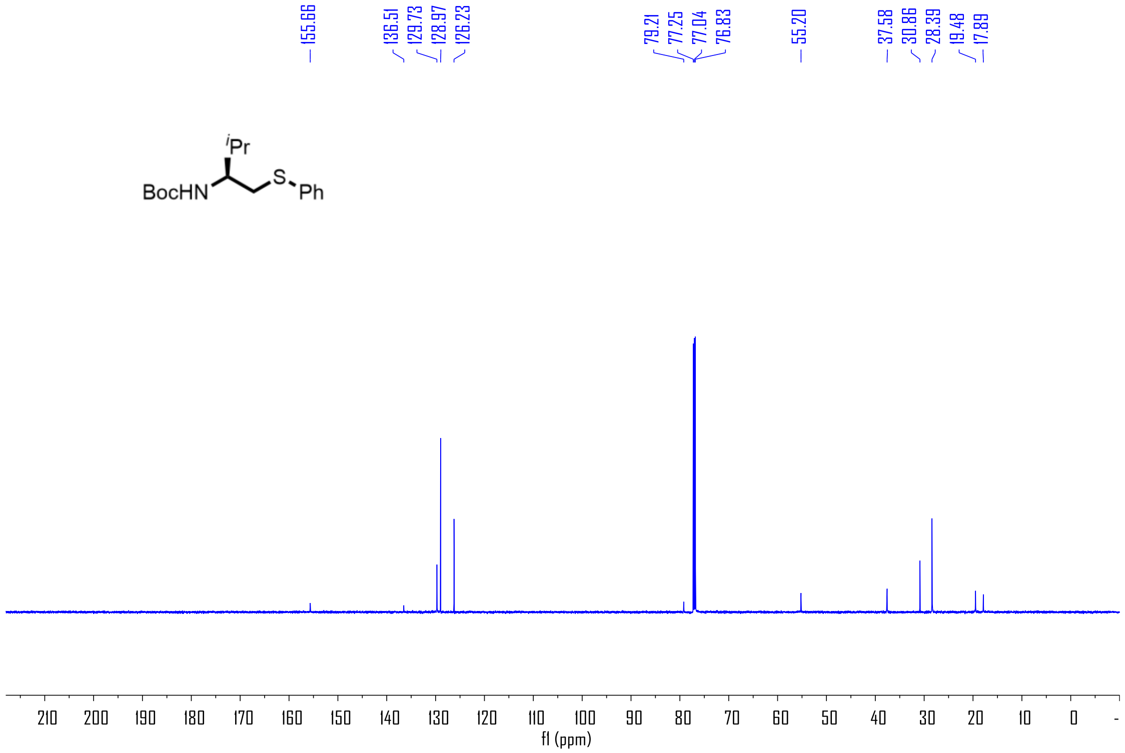
155.66
136.51
129.73
128.97
126.23
79.21
77.25
77.04
76.83
55.20
37.58
30.86
28.39
19.48
17.89
BocHN
iPr
S
Ph
fl (ppm)

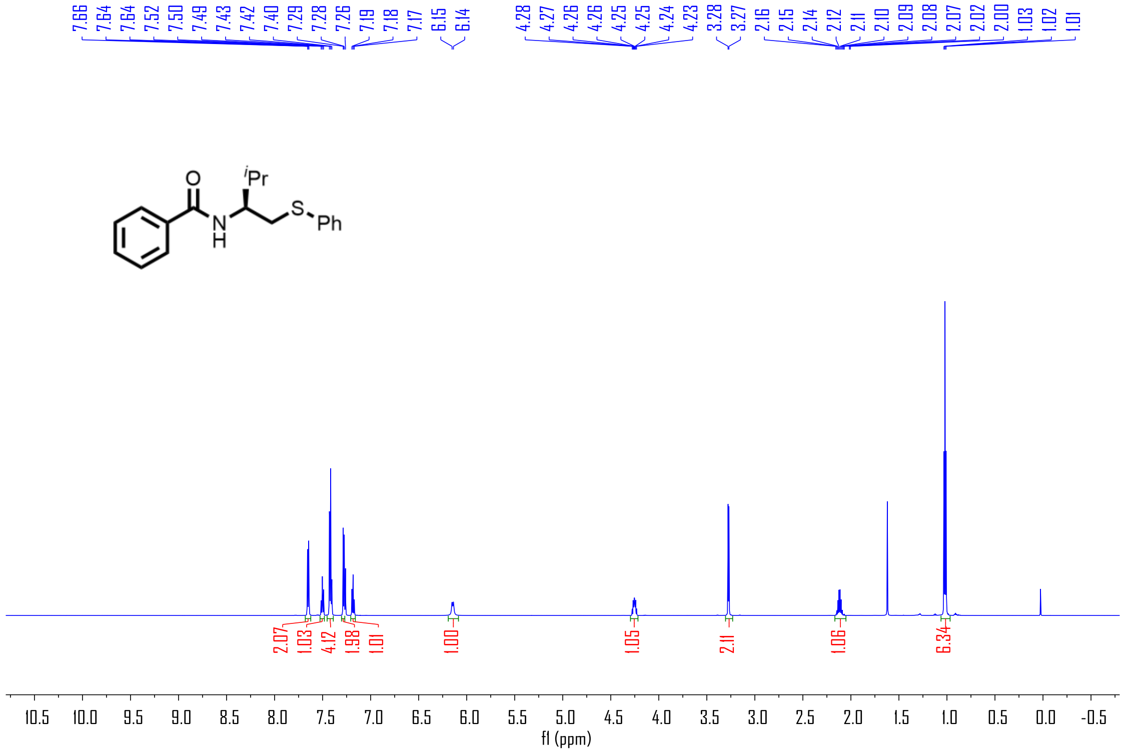
7.66
7.64
7.64
7.52
7.50
7.49
7.43
7.42
7.40
7.29
7.28
7.26
7.19
7.18
7.17
6.15
6.14
4.28
4.27
4.26
4.26
4.25
4.25
4.24
4.23
3.28
3.27
2.16
2.15
2.14
2.12
2.11
2.10
2.09
2.08
2.07
2.02
2.00
1.03
1.02
1.01
O
N
H
iPr
S
Ph
2.07
1.03
4.12
1.98
1.01
1.00
1.05
2.11
1.06
6.34
fl (ppm)

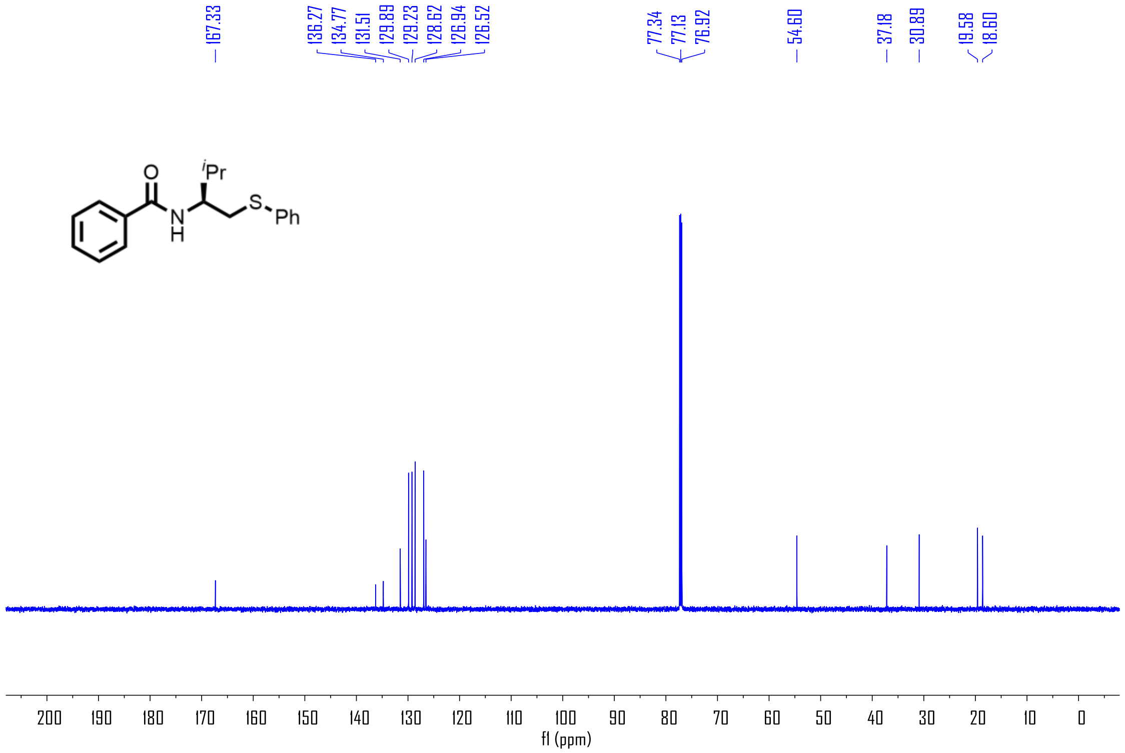
167.33
136.27
134.77
131.51
129.89
129.23
128.62
126.94
126.52
77.34
77.13
76.92
54.60
37.18
30.89
19.58
18.60
O
iPr
N
H
S
Ph
200 190 180 170 160 150 140 130 120 110 100 90 80 70 60 50 40 30 20 10 0
f1 (ppm)

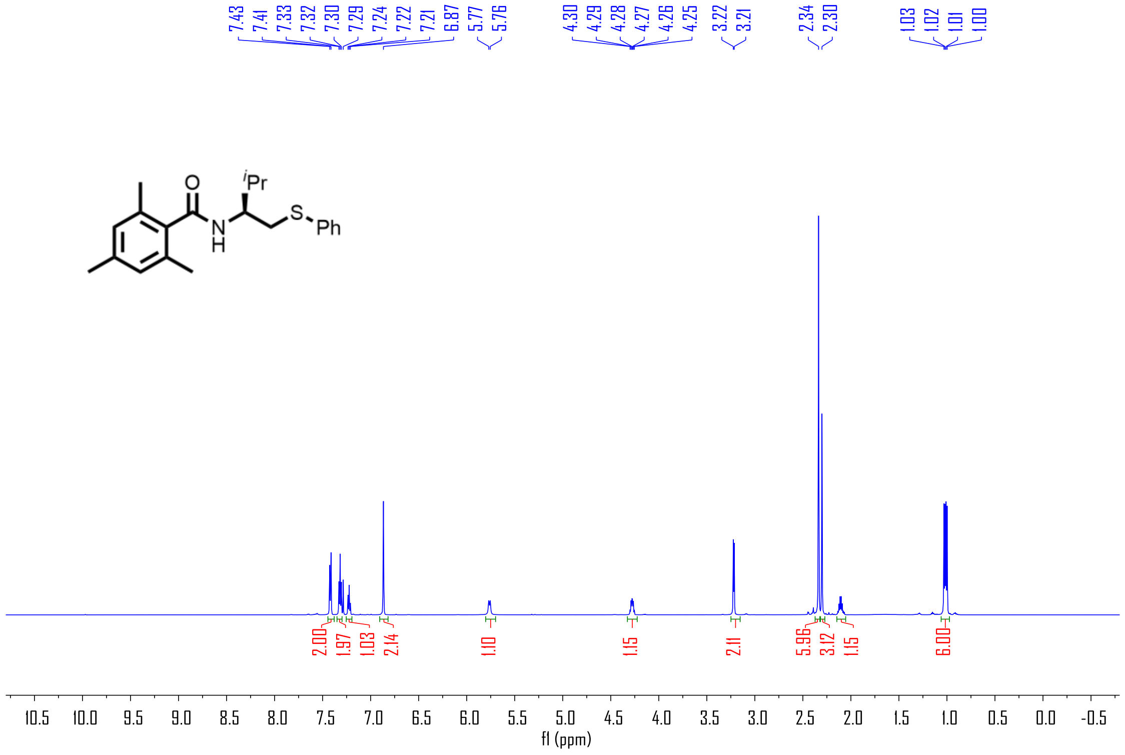
7.43
7.41
7.33
7.32
7.30
7.29
7.24
7.22
7.21
6.87
5.77
5.76
4.30
4.29
4.28
4.27
4.26
4.25
3.22
3.21
2.34
2.30
1.03
1.02
1.01
1.00
O
iPr
N
H
S
Ph
2.00
1.97
1.03
2.14
1.10
1.15
2.11
5.96
3.12
1.15
6.00
10.5 10.0 9.5 9.0 8.5 8.0 7.5 7.0 6.5 6.0 5.5 5.0 4.5 4.0 3.5 3.0 2.5 2.0 1.5 1.0 0.5 0.0 -0.5
f1 (ppm)

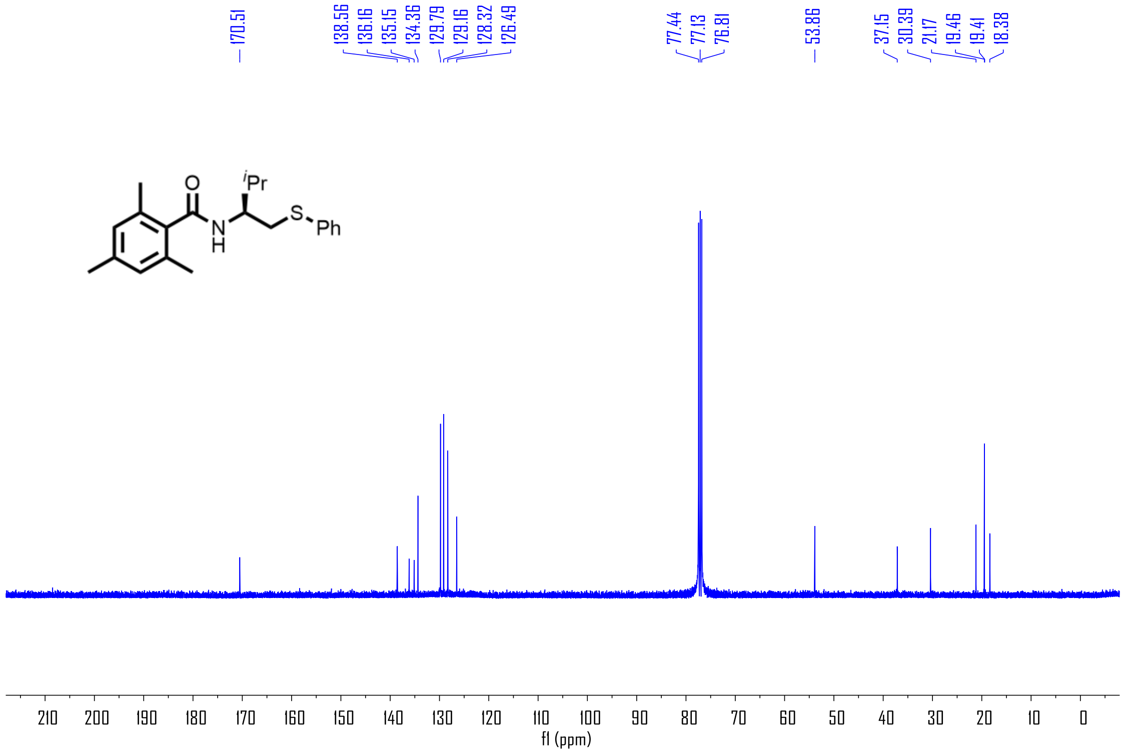
170.51
138.56
136.16
135.15
134.36
129.79
129.16
128.32
126.49
77.44
77.13
76.81
53.86
37.15
30.39
21.17
19.46
19.41
18.38
O
iPr
N
H
S
Ph
210
200
190
180
170
160
150
140
130
120
110
100
90
80
70
60
50
40
30
20
10
0
f1 (ppm)

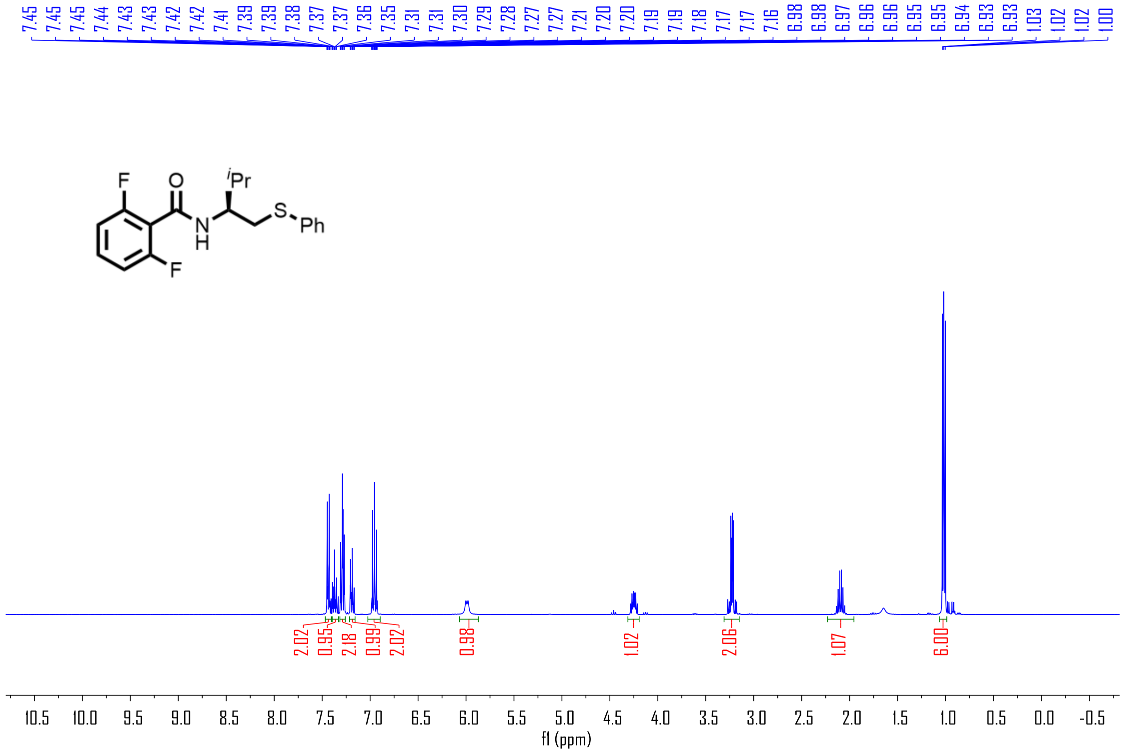
7.45
7.45
7.45
7.44
7.43
7.43
7.42
7.42
7.41
7.39
7.39
7.38
7.37
7.37
7.36
7.35
7.31
7.31
7.30
7.29
7.28
7.27
7.27
7.21
7.20
7.20
7.19
7.19
7.18
7.17
7.17
7.16
6.98
6.98
6.97
6.96
6.96
6.95
6.95
6.94
6.93
6.93
1.03
1.02
1.02
1.00
F
O
iPr
N
H
S
Ph
F
2.02
0.95
2.18
0.99
2.02
0.98
1.02
2.06
1.07
6.00
10.5
10.0
9.5
9.0
8.5
8.0
7.5
7.0
6.5
6.0
5.5
5.0
4.5
4.0
3.5
3.0
2.5
2.0
1.5
1.0
0.5
0.0
-0.5
f1 (ppm)

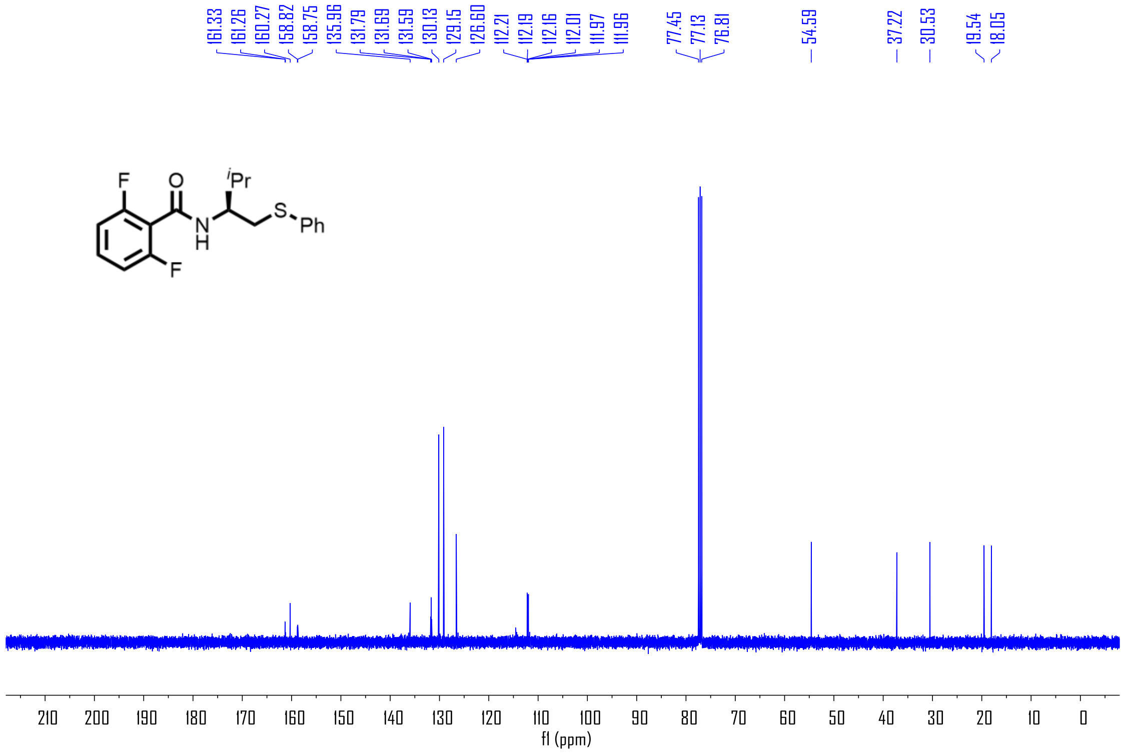
161.33
161.26
160.27
158.82
158.75
135.96
131.79
131.69
131.59
130.13
129.15
126.60
112.21
112.19
112.16
112.01
111.97
111.96
77.45
77.13
76.81
54.59
37.22
30.53
19.54
18.05
F
O
iPr
N
H
S
Ph
F
210 200 190 180 170 160 150 140 130 120 110 100 90 80 70 60 50 40 30 20 10 0
f1 (ppm)

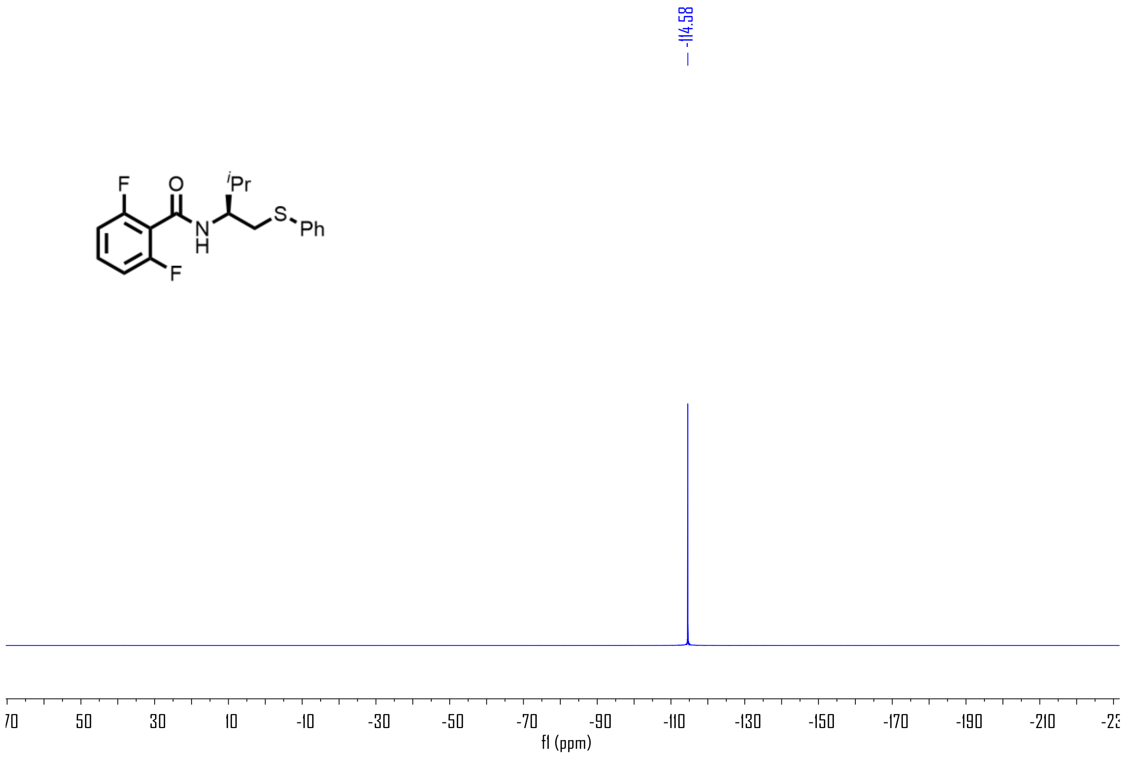
-114.58
F
O
iPr
N
H
S
Ph
F
50 30 10 -10 -30 -50 -70 -90 -110 -130 -150 -170 -190 -210
f1 (ppm)

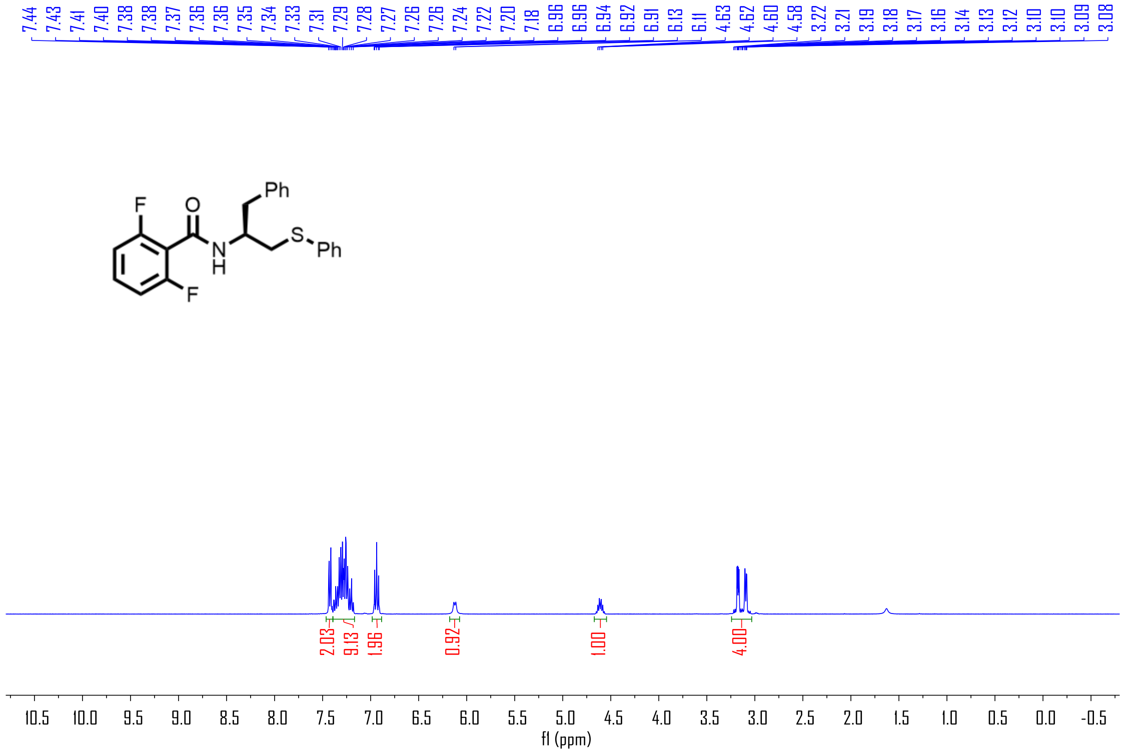

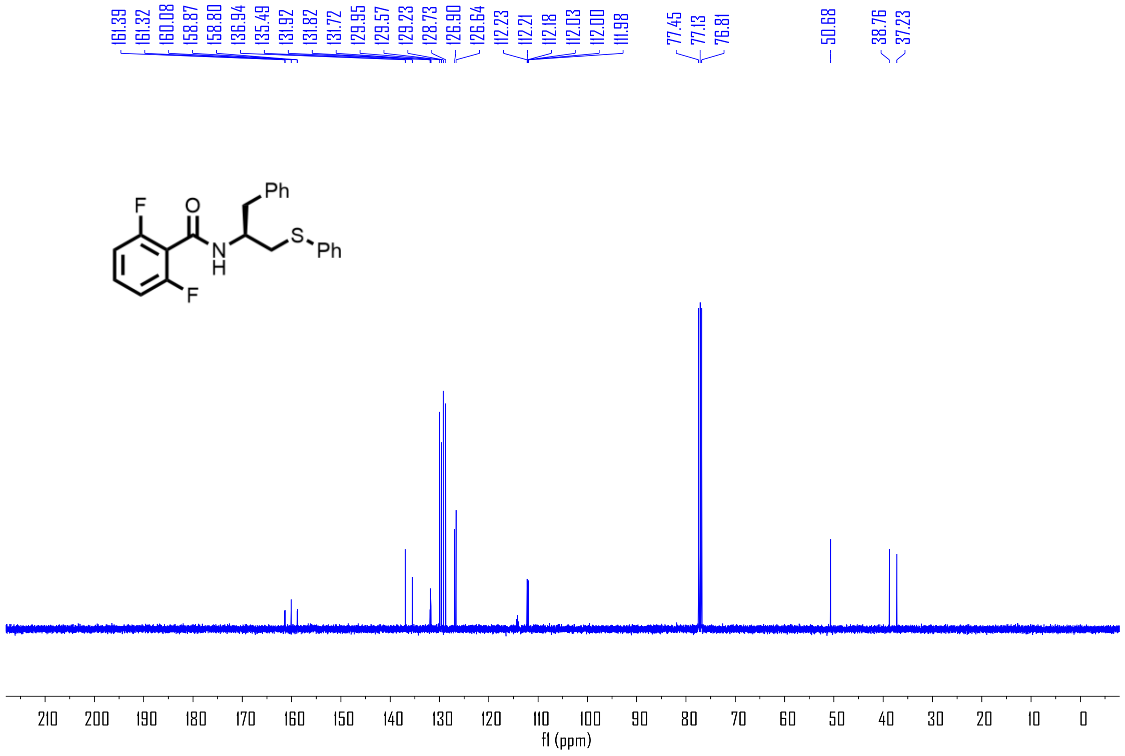

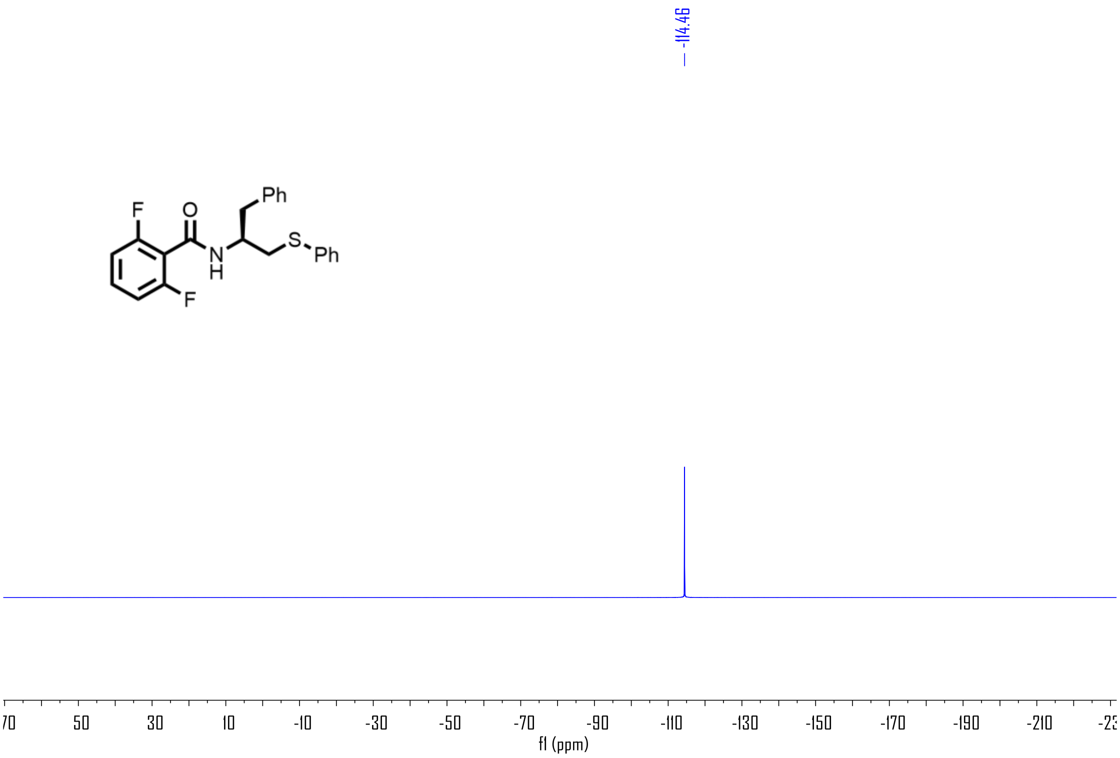

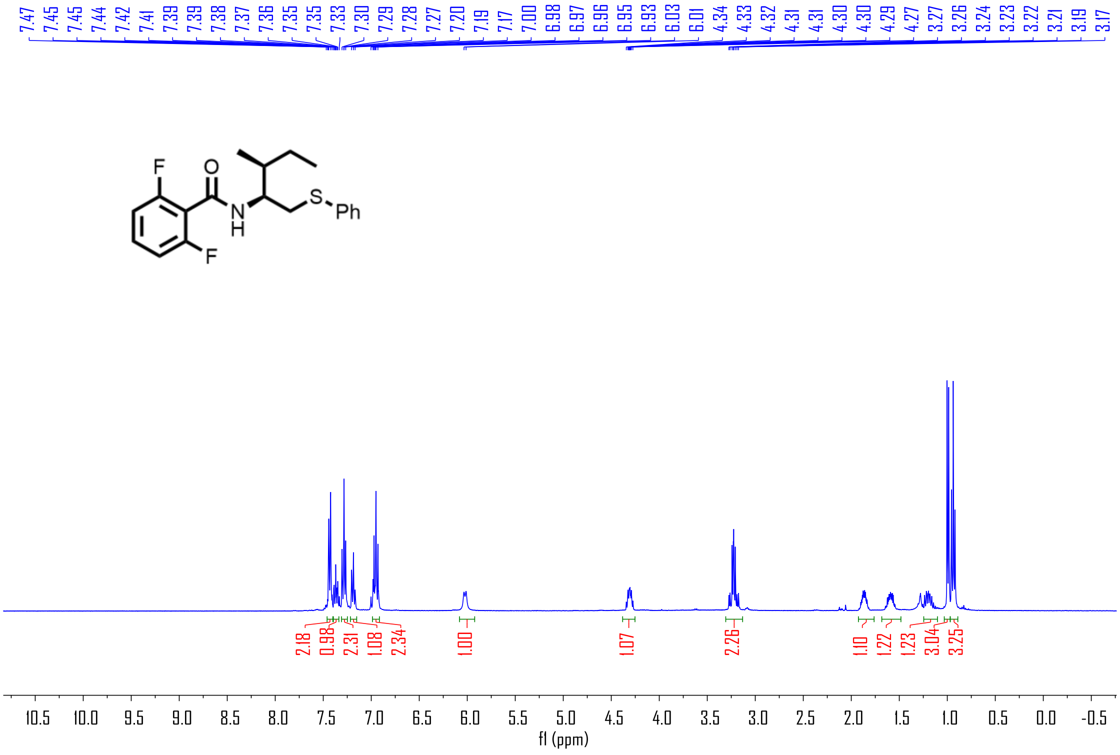

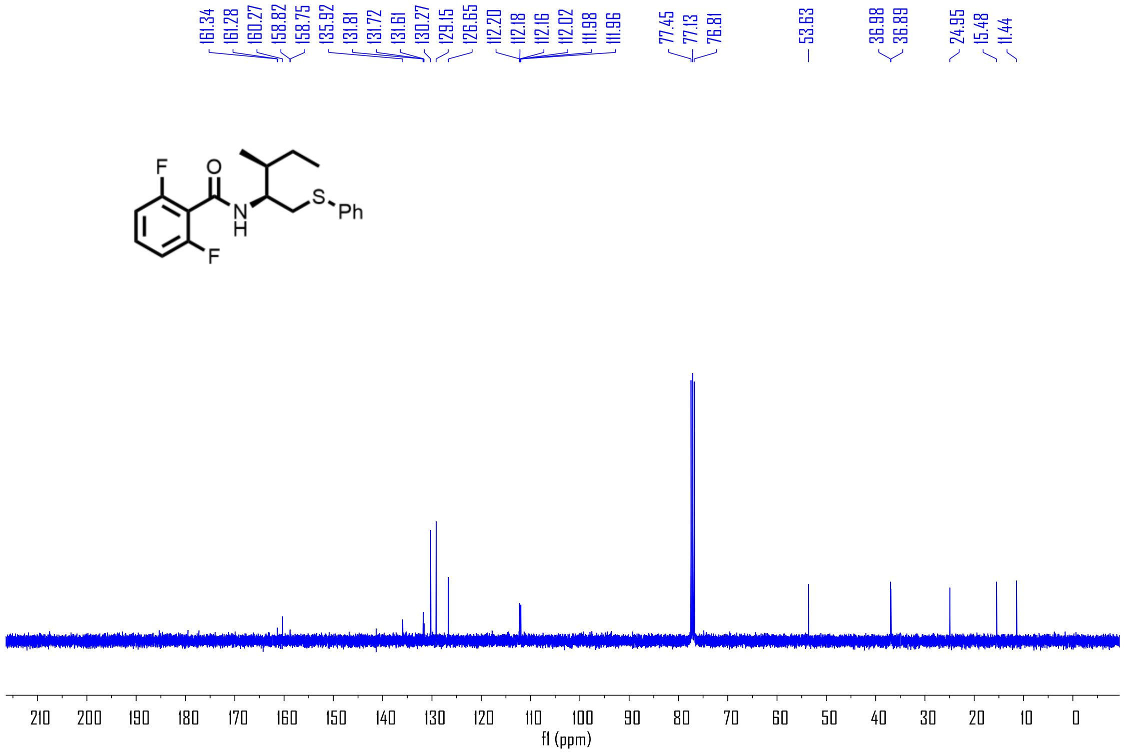
161.34
161.28
160.27
158.82
158.75
135.92
131.81
131.72
131.61
130.27
129.15
126.65
112.20
112.18
112.16
112.02
111.98
111.96
77.45
77.13
76.81
53.63
36.98
36.89
24.95
15.48
11.44
F
O
N
H
S
Ph
F
f1 (ppm)

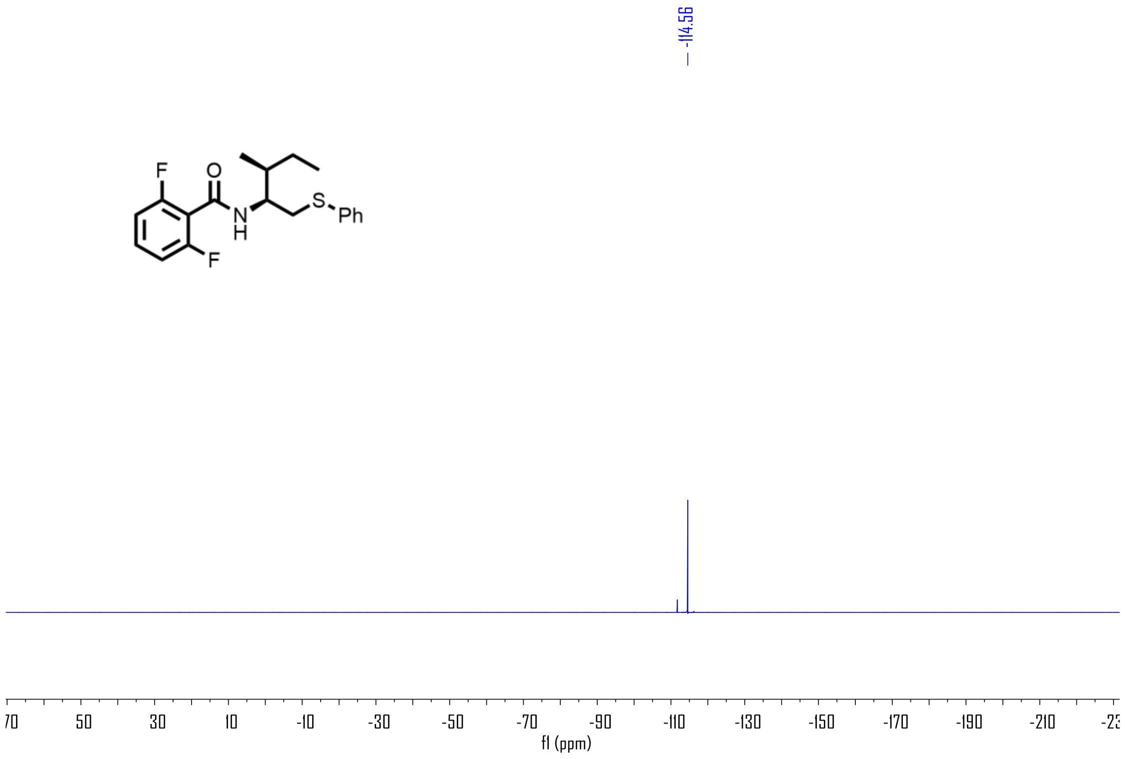
-114.56
F
O
N
H
S
Ph
F
f1 (ppm)

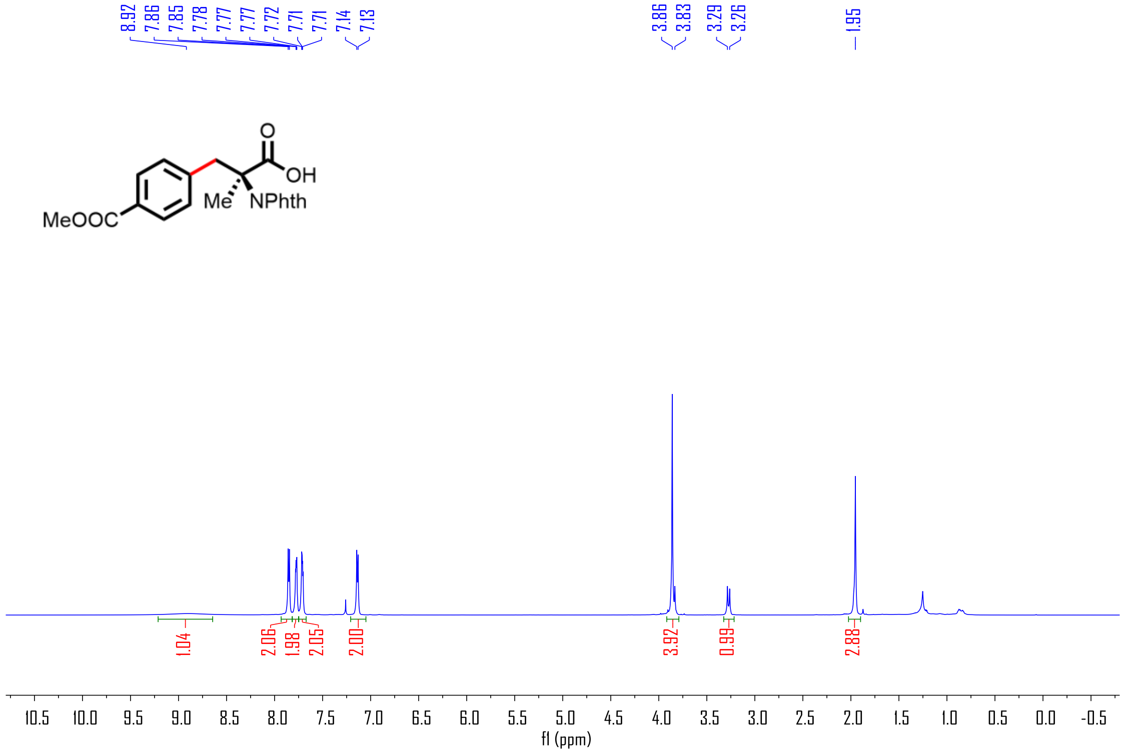
8.92
7.86
7.85
7.78
7.77
7.77
7.72
7.71
7.71
7.14
7.13
3.86
3.83
3.29
3.26
1.95
MeOOC
O
OH
Me
NPhth
1.04
2.06
1.98
2.05
2.00
3.92
0.99
2.88
10.5 10.0 9.5 9.0 8.5 8.0 7.5 7.0 6.5 6.0 5.5 5.0 4.5 4.0 3.5 3.0 2.5 2.0 1.5 1.0 0.5 0.0 -0.5
f1 (ppm)

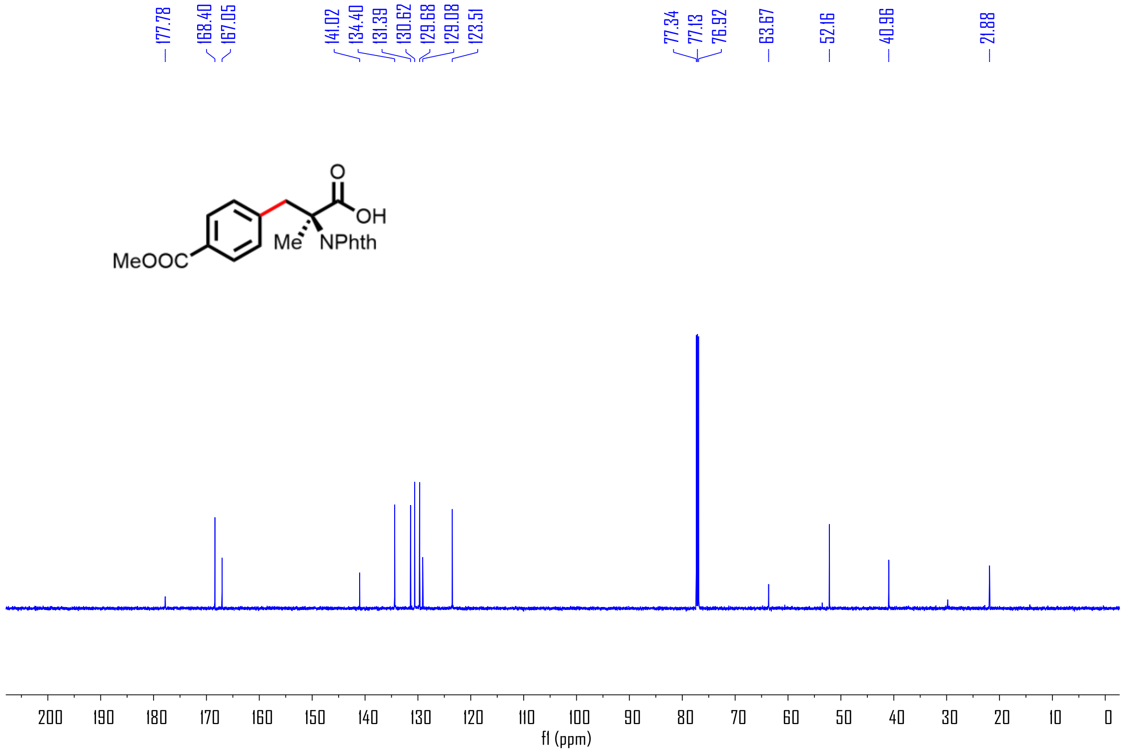
177.78
168.40
167.05
141.02
134.40
131.39
130.62
129.68
129.08
123.51
77.34
77.13
76.92
63.67
52.16
40.96
21.88
MeOOC
O
OH
Me
NPhth
200 190 180 170 160 150 140 130 120 110 100 90 80 70 60 50 40 30 20 10 0
f1 (ppm)

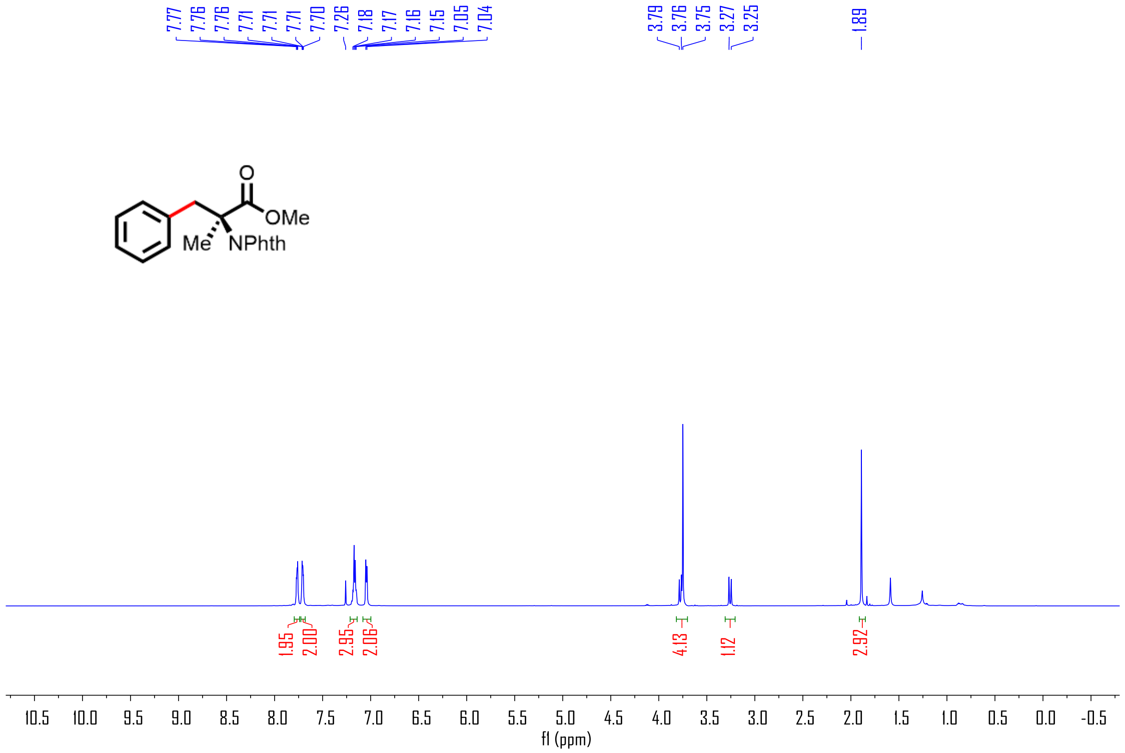
OMe
Me
NPhth
7.77
7.76
7.76
7.71
7.71
7.71
7.70
7.26
7.18
7.17
7.16
7.15
7.05
7.04
3.79
3.76
3.75
3.27
3.25
1.89
1.95
2.00
2.95
2.06
4.13
1.12
2.92
f1 (ppm)

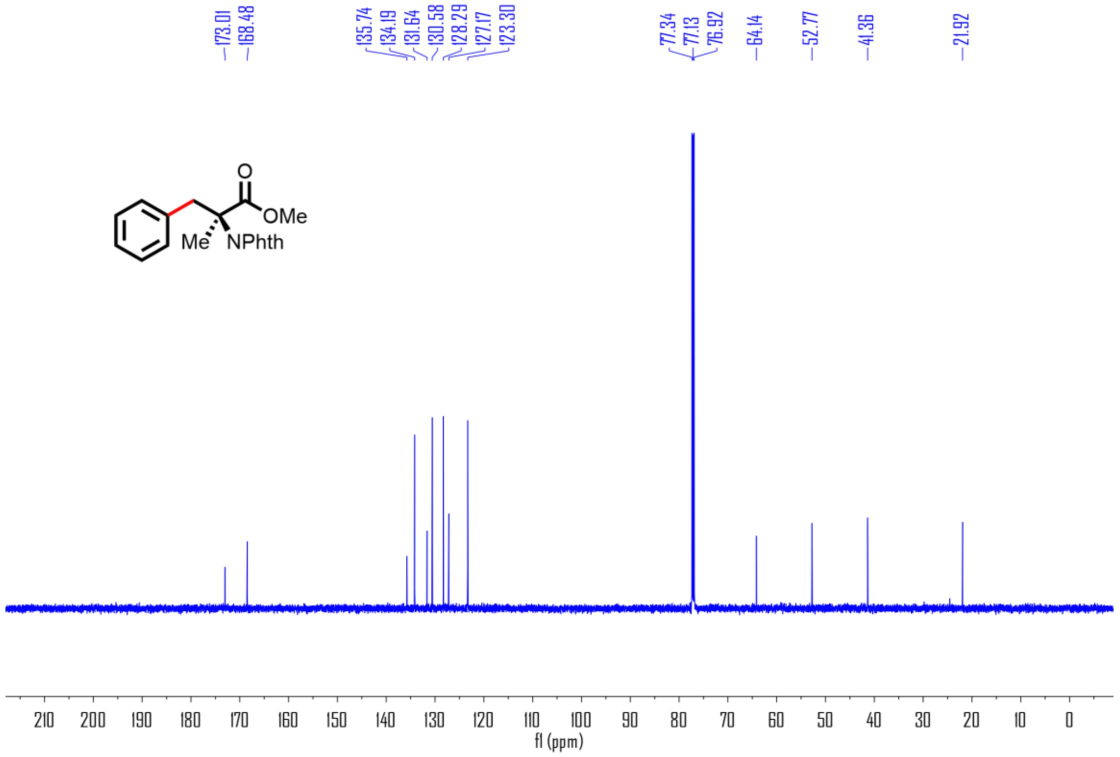
OMe
Me
NPhth
173.01
168.48
135.74
134.19
131.64
130.58
128.29
127.17
123.30
77.34
77.13
76.92
64.14
52.77
41.36
21.92
f1 (ppm)

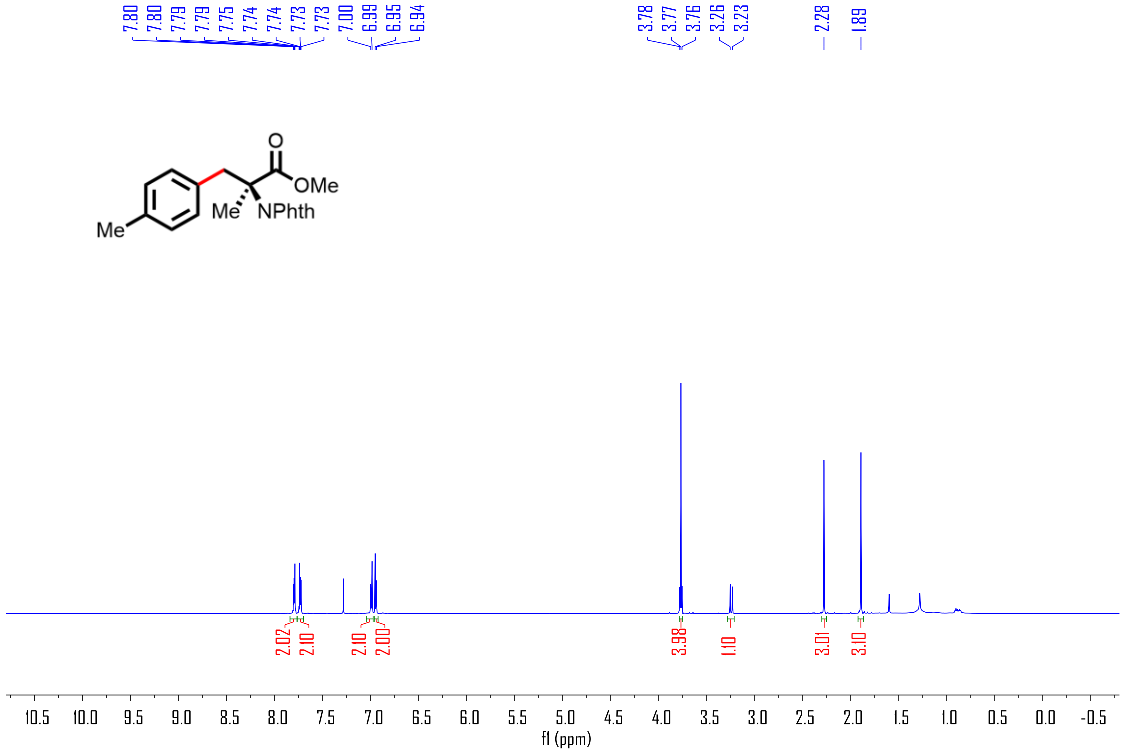
7.80
7.80
7.79
7.79
7.75
7.74
7.74
7.73
7.73
7.00
6.99
6.95
6.94
3.78
3.77
3.76
3.26
3.23
2.28
1.89
O
OMe
Me
NPhth
Me
2.02
2.10
2.10
2.00
3.98
1.10
3.01
3.10
10.5 10.0 9.5 9.0 8.5 8.0 7.5 7.0 6.5 6.0 5.5 5.0 4.5 4.0 3.5 3.0 2.5 2.0 1.5 1.0 0.5 0.0 -0.5
f1 (ppm)

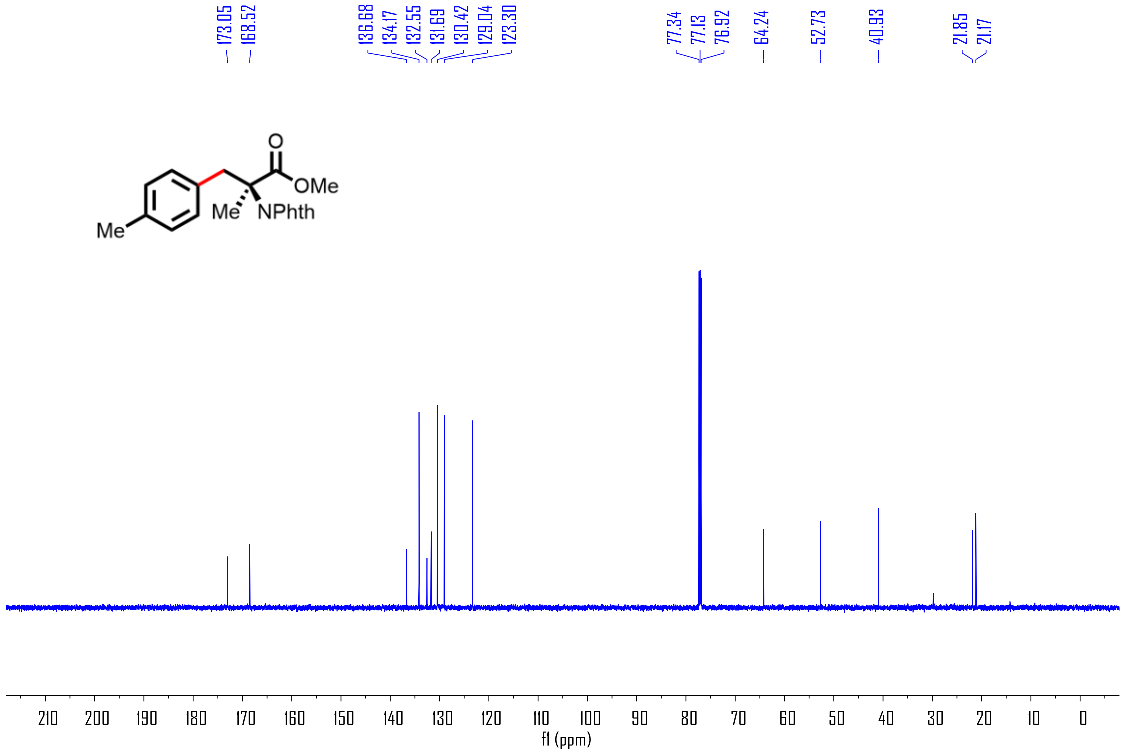
173.05
168.52
136.68
134.17
132.55
131.69
130.42
129.04
123.30
77.34
77.13
76.92
64.24
52.73
40.93
21.85
21.17
O
OMe
Me
NPhth
Me
210 200 190 180 170 160 150 140 130 120 110 100 90 80 70 60 50 40 30 20 10 0
f1 (ppm)

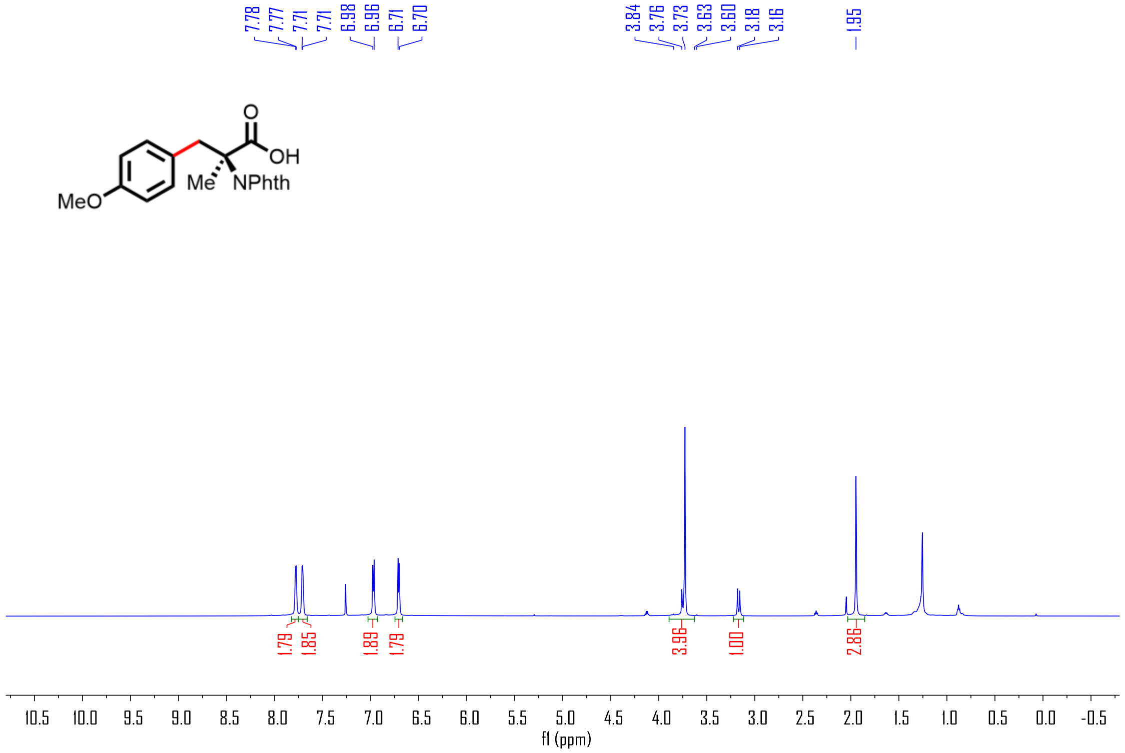
7.78
7.77
7.71
7.71
6.98
6.96
6.71
6.70
3.84
3.76
3.73
3.63
3.60
3.18
3.16
1.95
MeO
Me
NPhth
O
OH
1.79
1.85
1.89
1.79
3.96
1.00
2.86
fl (ppm)

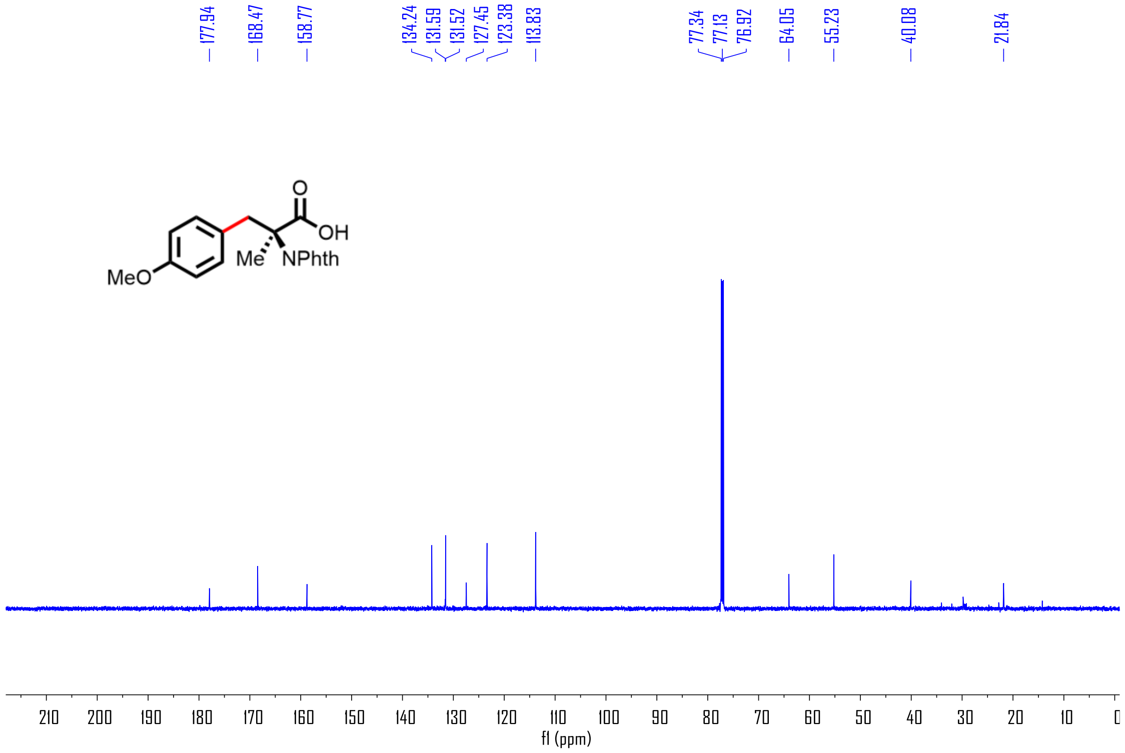
177.94
168.47
158.77
134.24
131.59
131.52
127.45
123.38
113.83
77.34
77.13
76.92
64.05
55.23
40.08
21.84
MeO
Me
NPhth
O
OH
fl (ppm)

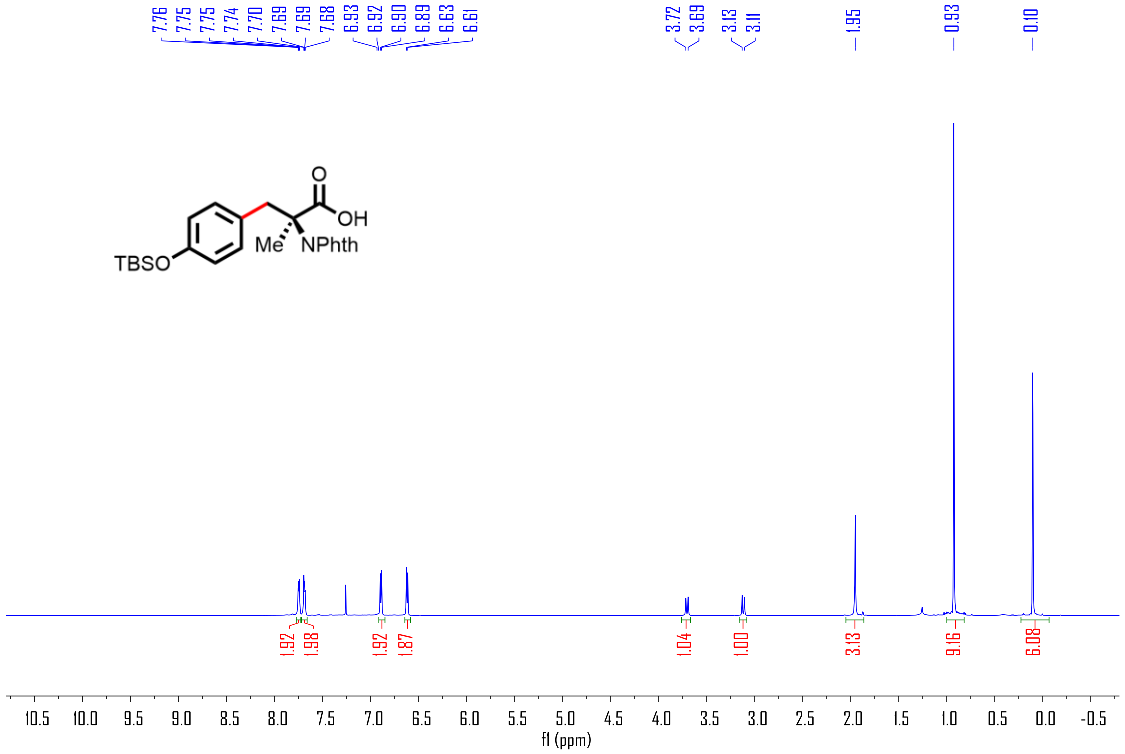
7.76
7.75
7.75
7.74
7.70
7.69
7.69
7.68
6.93
6.92
6.90
6.89
6.63
6.61
3.72
3.69
3.13
3.11
1.95
0.93
0.10
TBSO
Me
NPhth
O
OH
1.92
1.98
1.92
1.87
1.04
1.00
3.13
9.16
6.08
f1 (ppm)

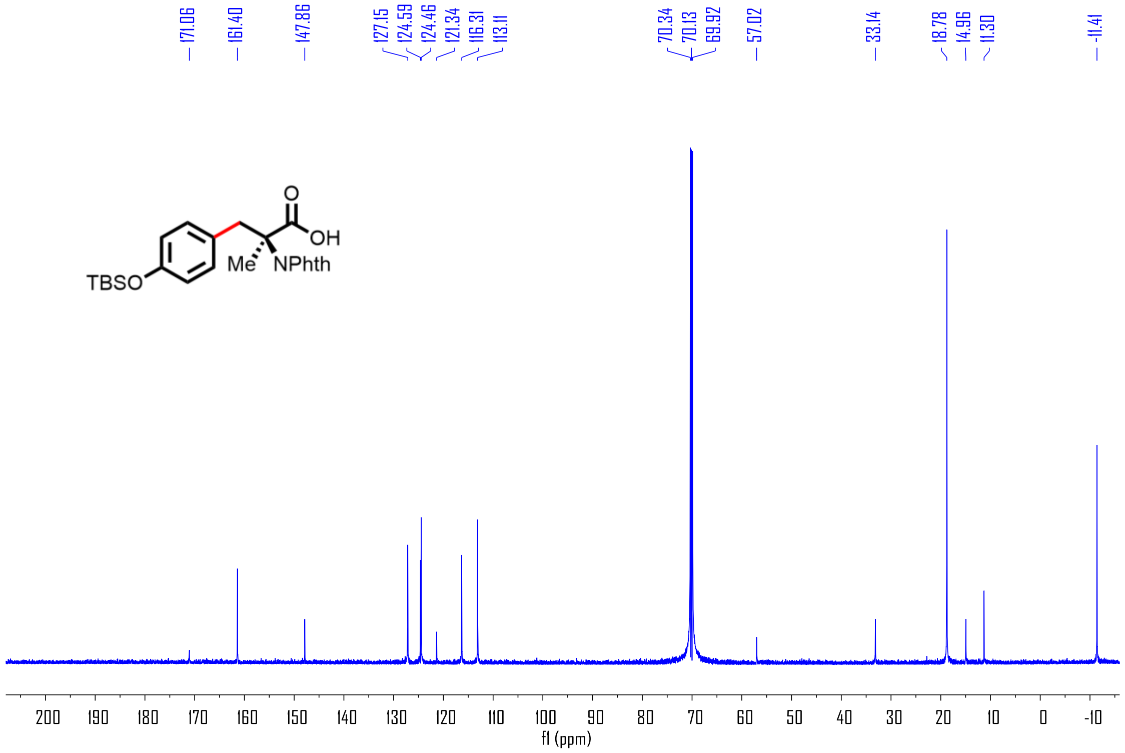
171.06
161.40
147.86
127.15
124.59
124.46
121.34
116.31
113.11
70.34
70.13
69.92
57.02
33.14
18.78
14.96
11.30
-11.41
TBSO
Me
NPhth
O
OH
f1 (ppm)

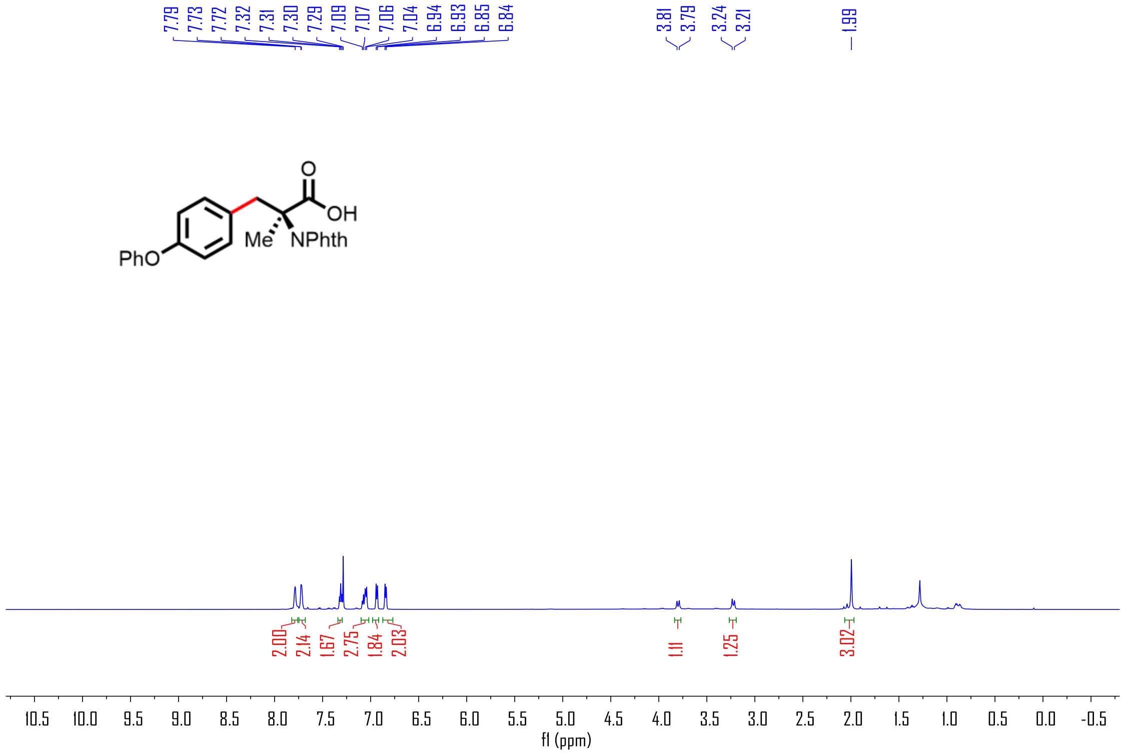
7.79 7.73 7.72 7.32 7.31 7.30 7.29 7.09 7.07 7.06 7.04 6.94 6.93 6.85 6.84
3.81 3.79 3.24 3.21
1.99
OH
O
PhO
Me
NPhth
2.00 2.14 1.67 2.75 1.84 2.03
1.11
1.25
3.02
10.5 10.0 9.5 9.0 8.5 8.0 7.5 7.0 6.5 6.0 5.5 5.0 4.5 4.0 3.5 3.0 2.5 2.0 1.5 1.0 0.5 0.0 -0.5
f1 (ppm)

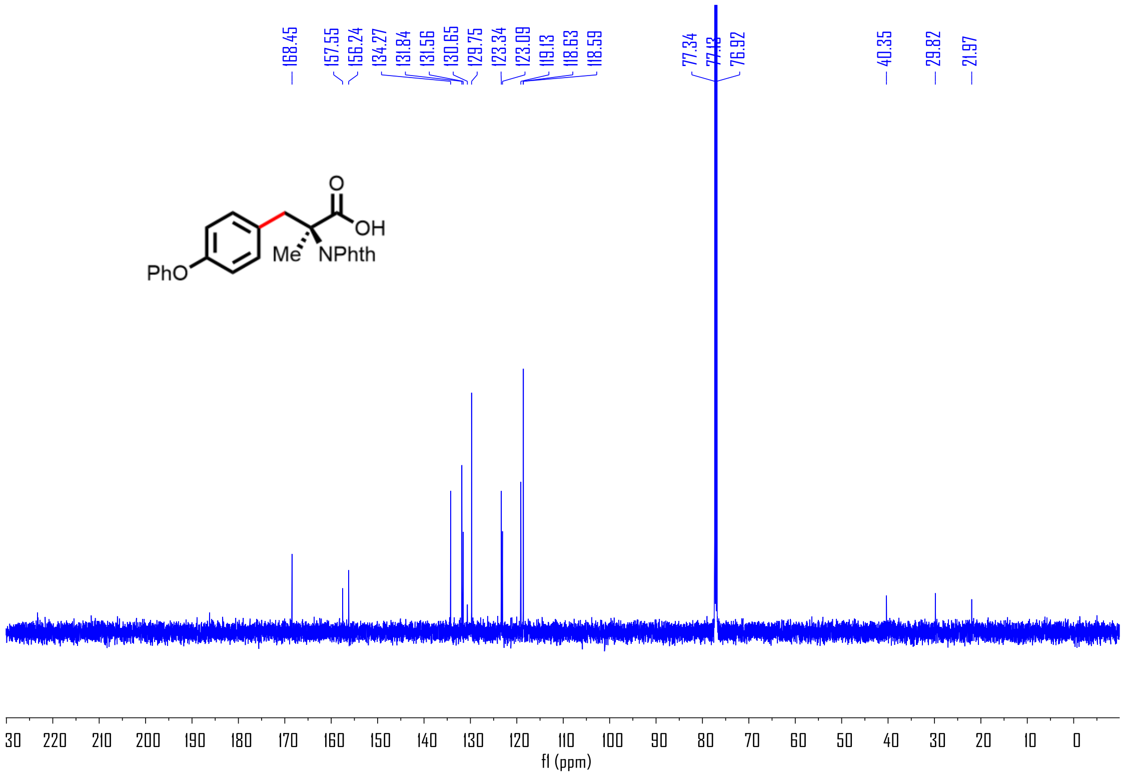
168.45
157.55 156.24 134.27 131.84 131.56 130.65 129.75 123.34 123.09 119.13 118.63 118.59
77.34 77.13 76.92
40.35
29.82
21.97
OH
O
PhO
Me
NPhth
30 220 210 200 190 180 170 160 150 140 130 120 110 100 90 80 70 60 50 40 30 20 10 0
f1 (ppm)

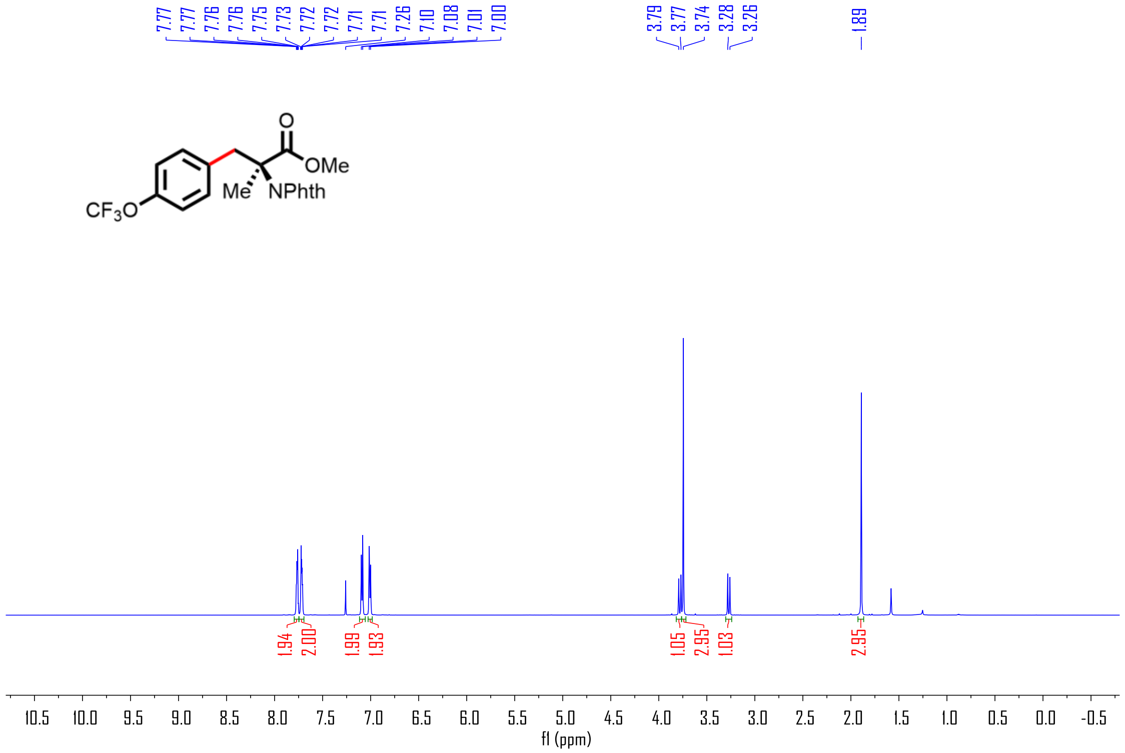
7.77
7.77
7.76
7.76
7.75
7.73
7.72
7.72
7.71
7.71
7.26
7.10
7.08
7.01
7.00
3.79
3.77
3.74
3.28
3.26
1.89
OMe
Me
NPhth
1.94
2.00
1.99
1.93
1.05
2.95
1.03
2.95
fl (ppm)

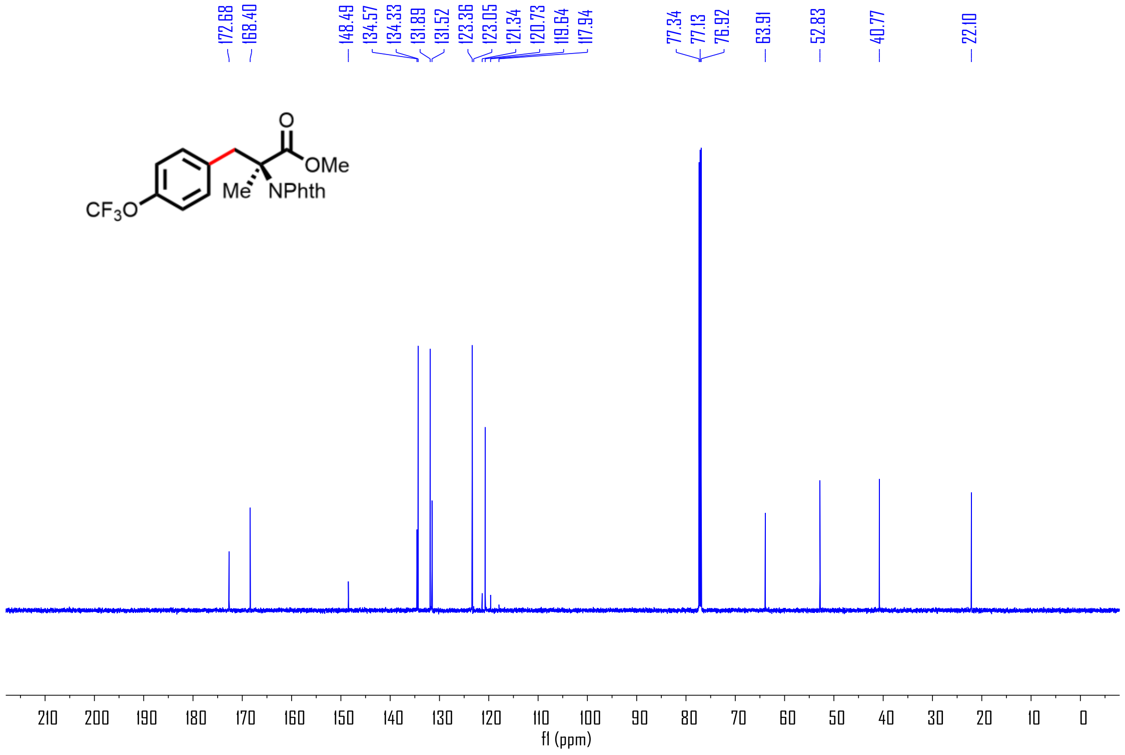
172.68
168.40
148.49
134.57
134.33
131.89
131.52
123.36
123.05
121.34
120.73
119.64
117.94
77.34
77.13
76.92
63.91
52.83
40.77
22.10
OMe
Me
NPhth
fl (ppm)

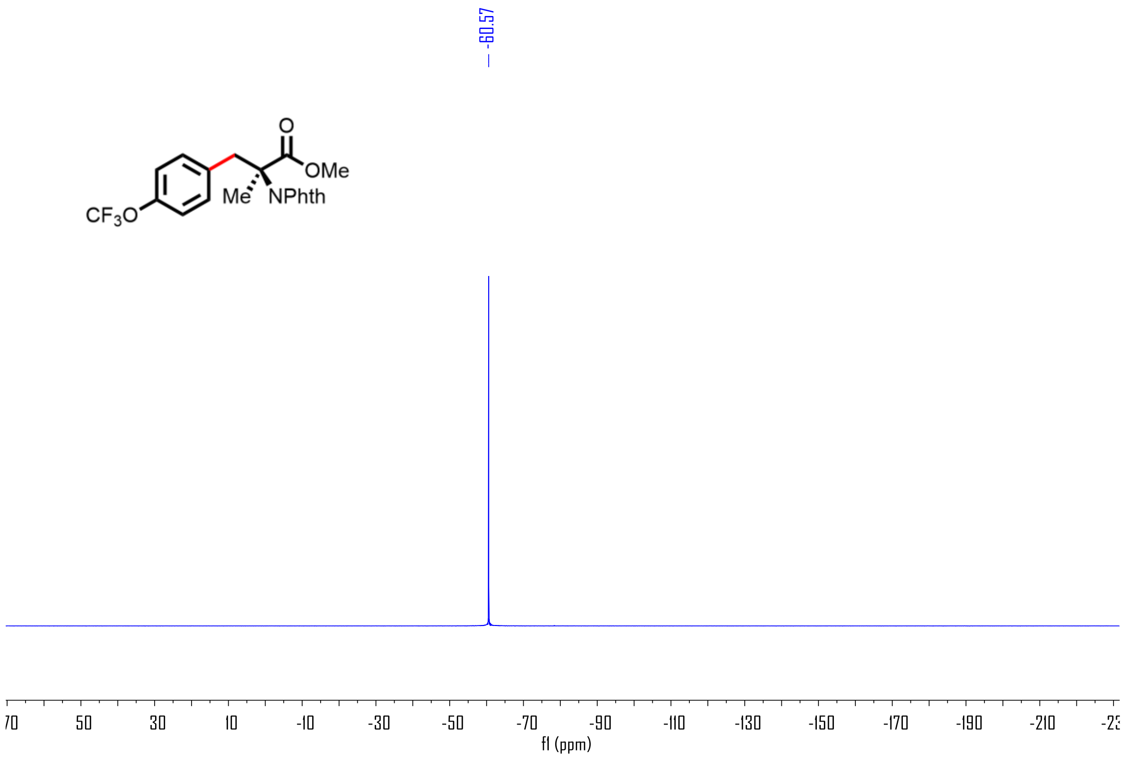

-60.57
CF3O
O
OMe
Me
NPhth
fl (ppm)

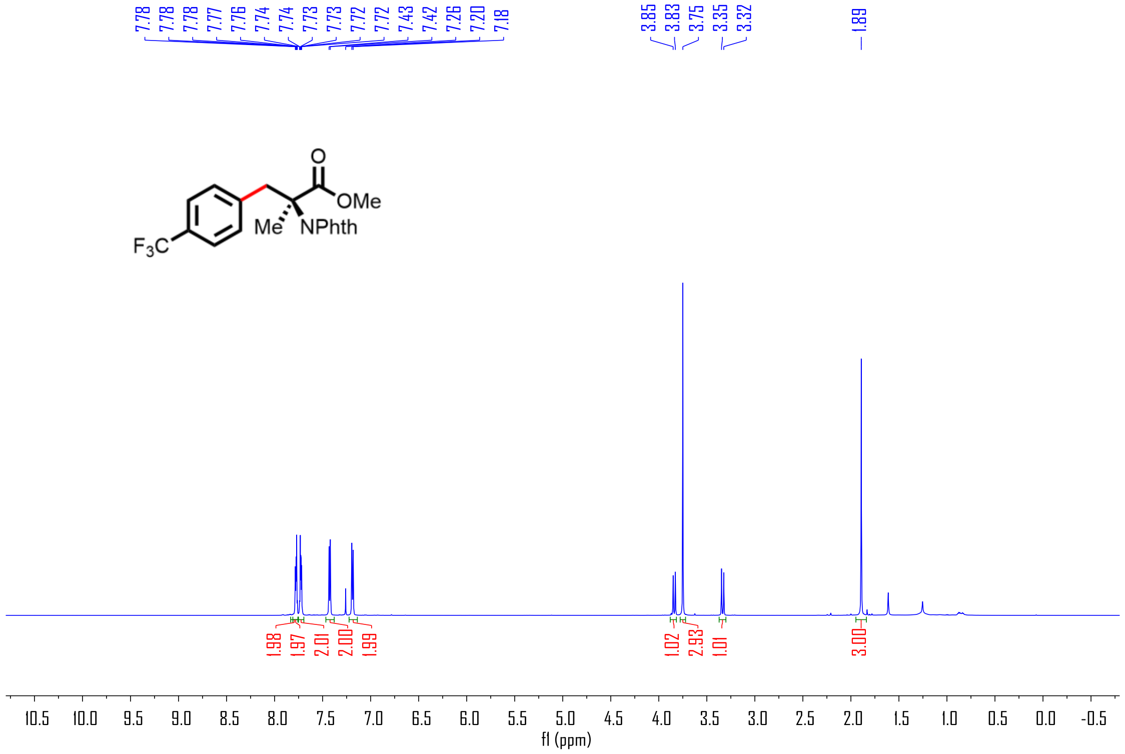

7.78 7.78 7.78 7.77 7.76 7.74 7.74 7.73 7.73 7.72 7.72 7.43 7.42 7.26 7.20 7.18
3.85 3.83 3.75 3.35 3.32
1.89
F3C
O
OMe
Me
NPhth
1.98 1.97 2.01 2.00 1.99
1.02 2.93 1.01
3.00
fl (ppm)

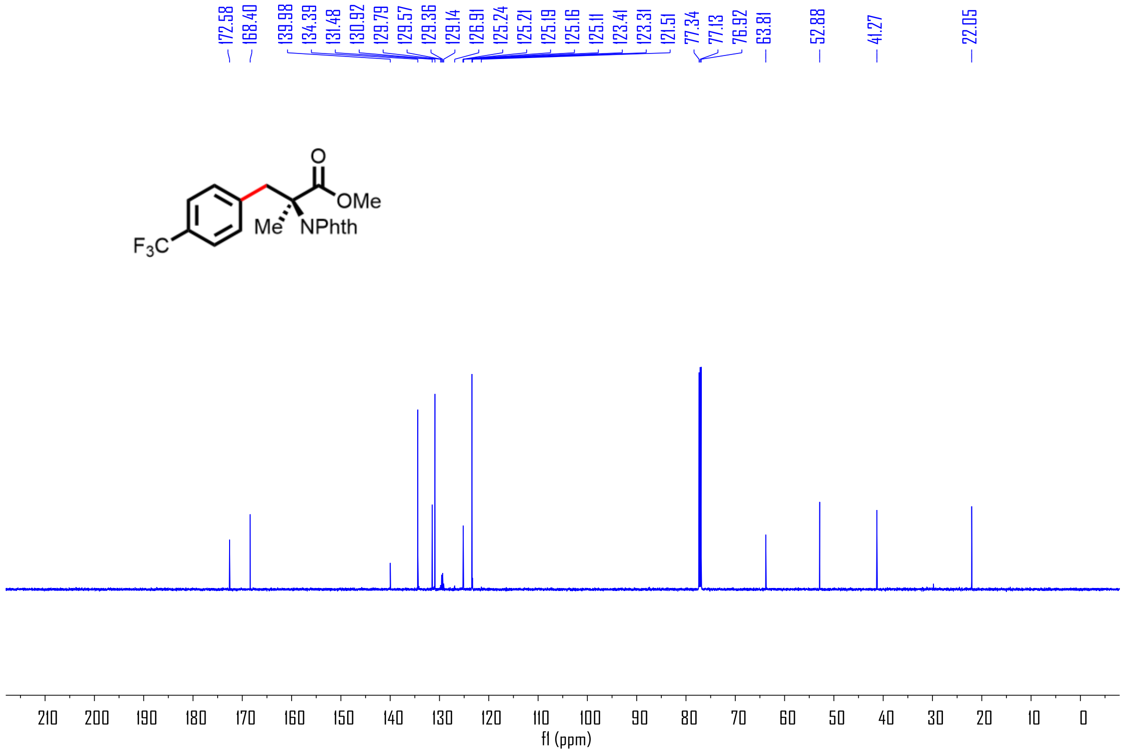
172.58
168.40
139.98
134.39
131.48
130.92
129.79
129.57
129.36
129.14
126.91
125.24
125.21
125.19
125.16
125.11
123.41
123.31
121.51
77.34
77.13
76.92
63.81
52.88
41.27
22.05
OMe
Me
NPhth
F3C
fl (ppm)

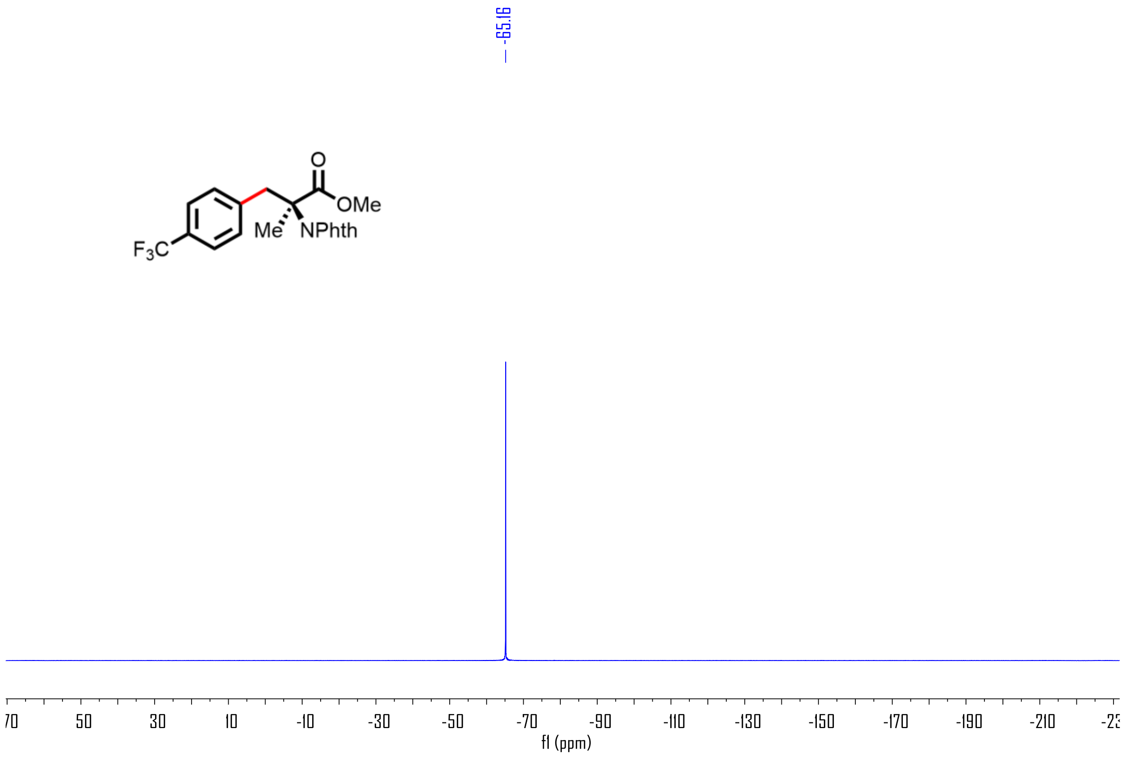
-65.16
OMe
Me
NPhth
F3C
fl (ppm)

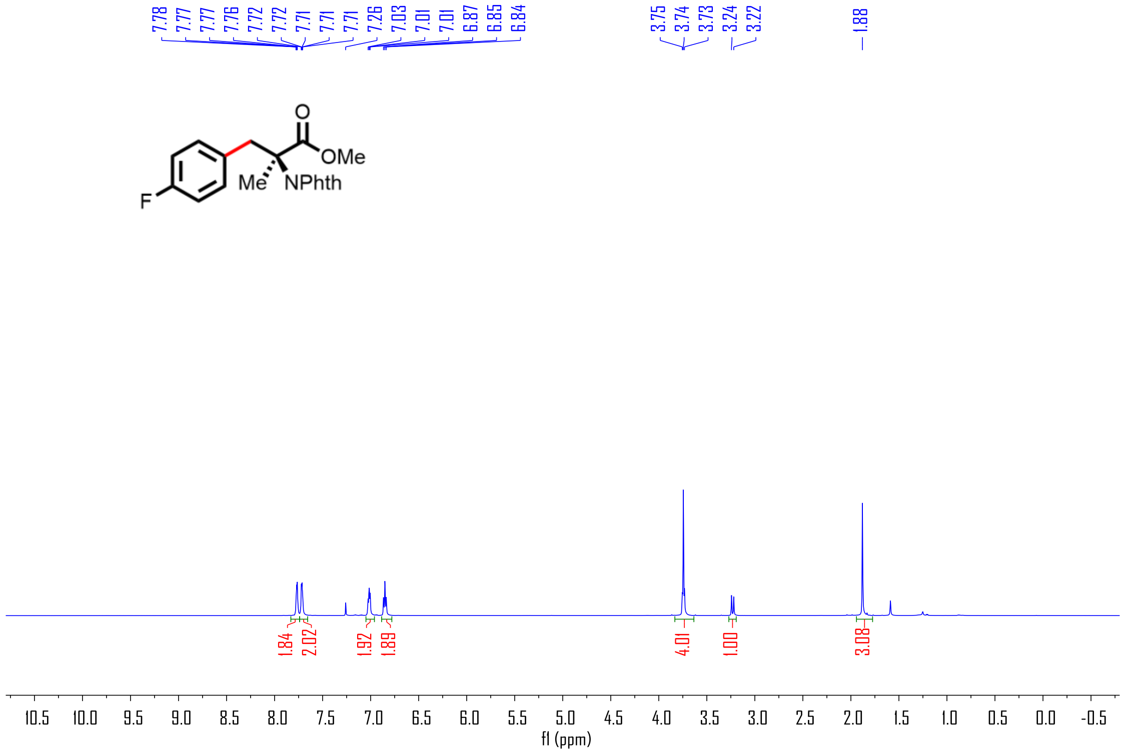

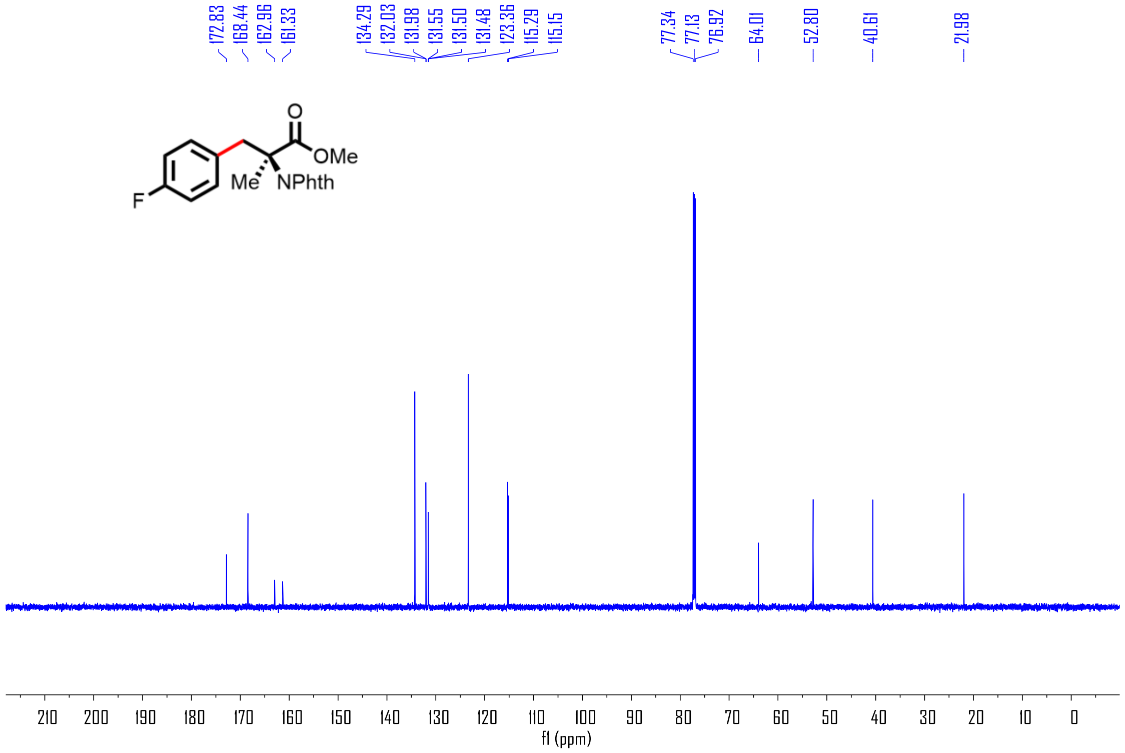

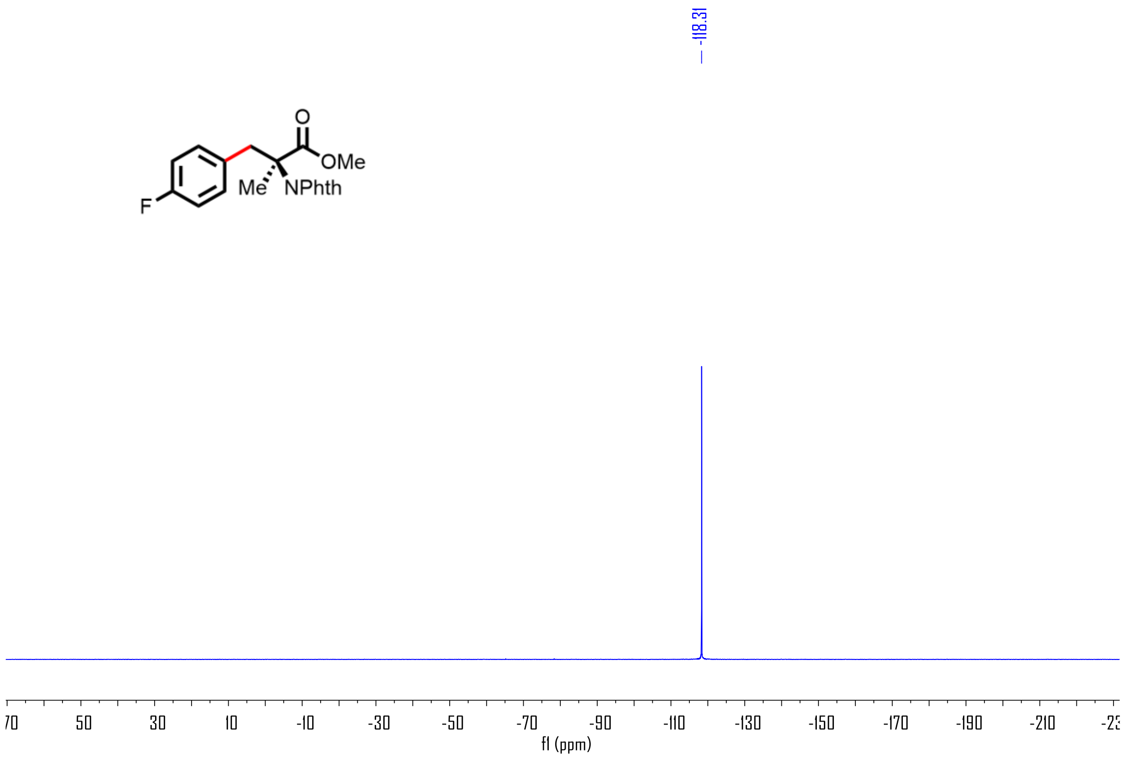

-118.31
OMe
Me
NPhth
F
fl (ppm)

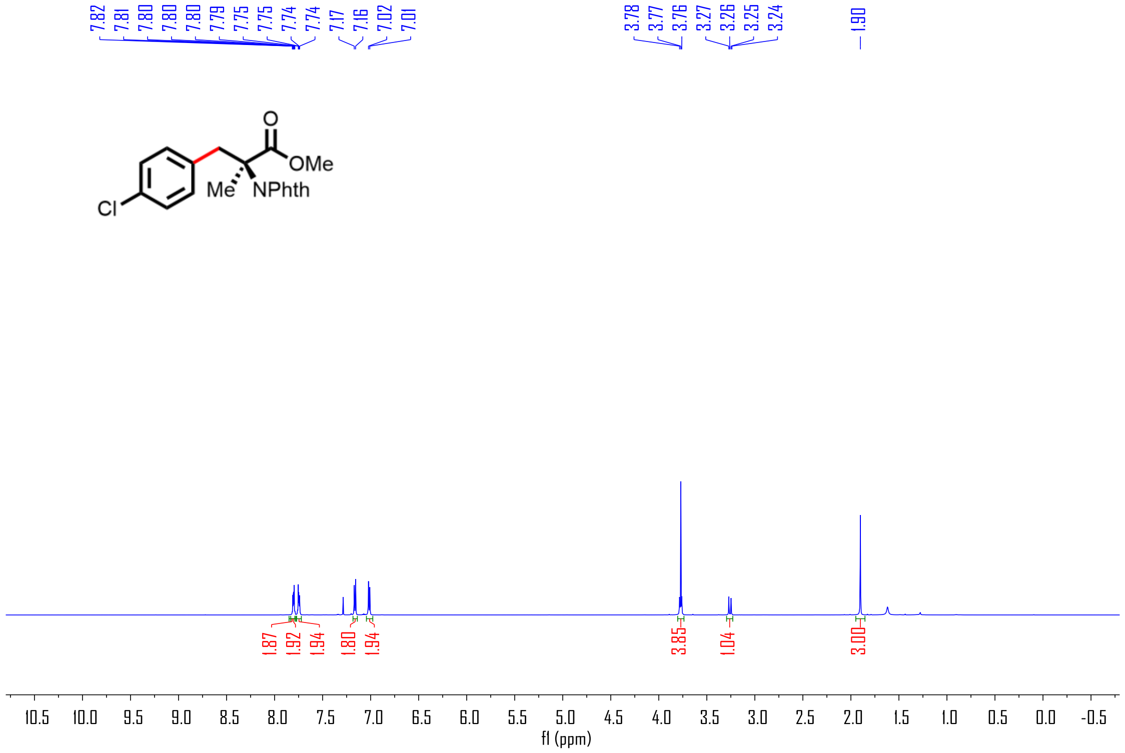

7.82
7.81
7.80
7.80
7.80
7.79
7.75
7.75
7.75
7.74
7.74
7.17
7.16
7.02
7.01
3.78
3.77
3.76
3.27
3.26
3.25
3.24
1.90
OMe
Me
NPhth
Cl
1.87
1.92
1.94
1.80
1.94
3.85
1.04
3.00
fl (ppm)

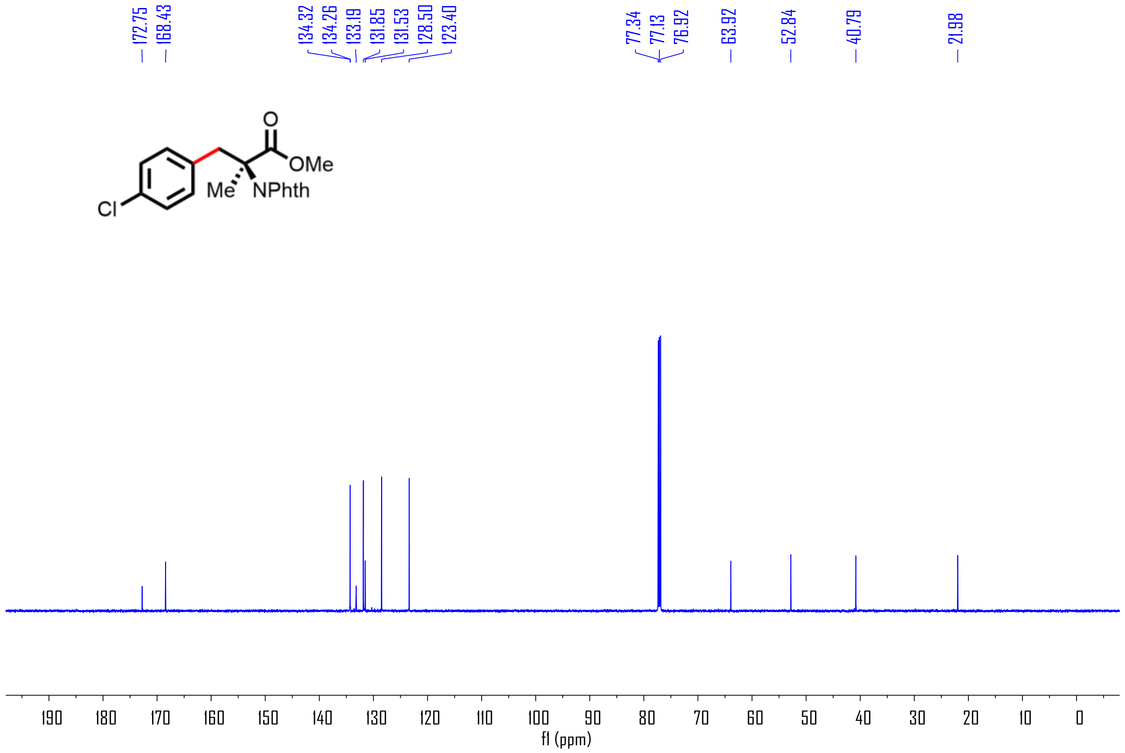
172.75
168.43
134.32
134.26
133.19
131.85
131.53
128.50
123.40
77.34
77.13
76.92
63.92
52.84
40.79
21.98
O
OMe
Cl
Me
NPhth
f1 (ppm)

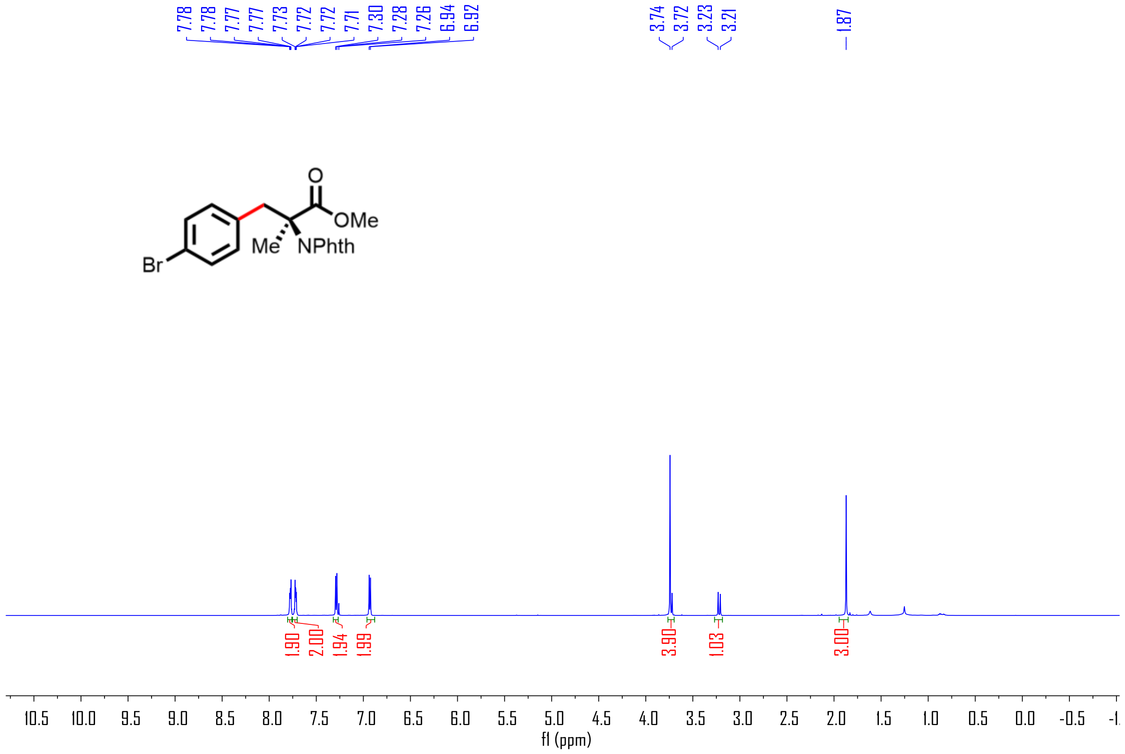
7.78
7.78
7.77
7.77
7.73
7.72
7.72
7.71
7.30
7.28
7.26
6.94
6.92
3.74
3.72
3.23
3.21
1.87
O
OMe
Br
Me
NPhth
1.90
2.00
1.94
1.99
3.90
1.03
3.00
f1 (ppm)

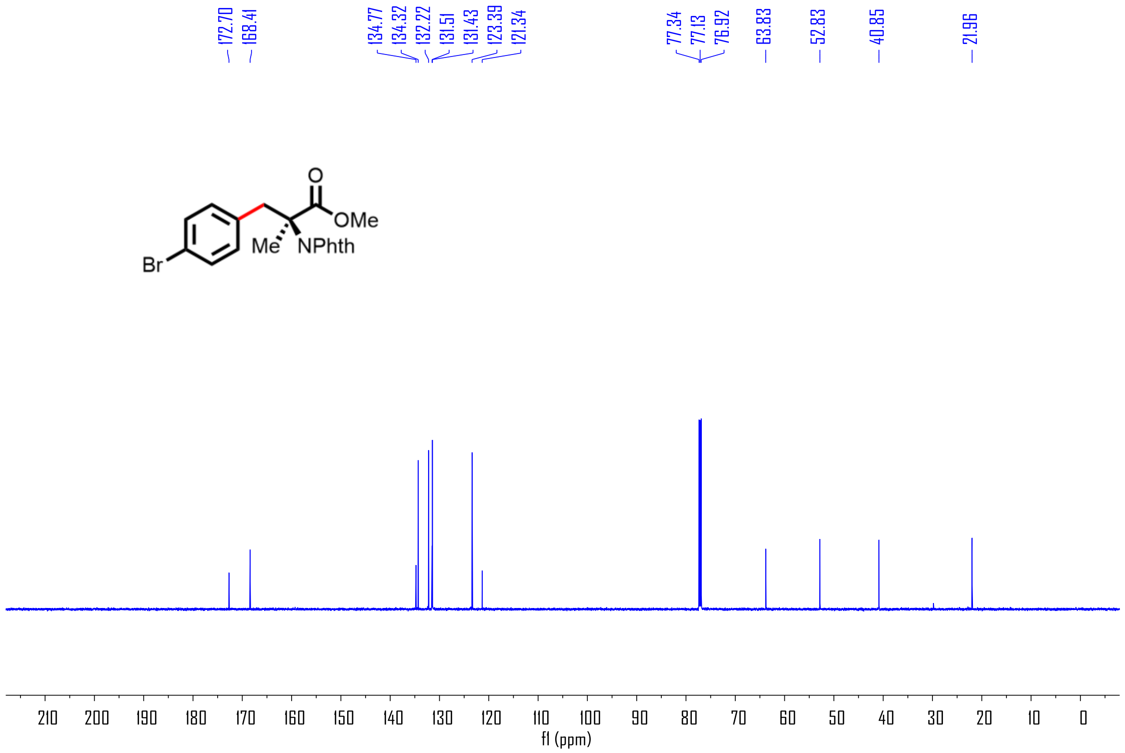
172.70
168.41
134.77
134.32
132.22
131.51
131.43
123.39
121.34
77.34
77.13
76.92
63.83
52.83
40.85
21.96
OMe
Me
NPhth
Br
fl (ppm)

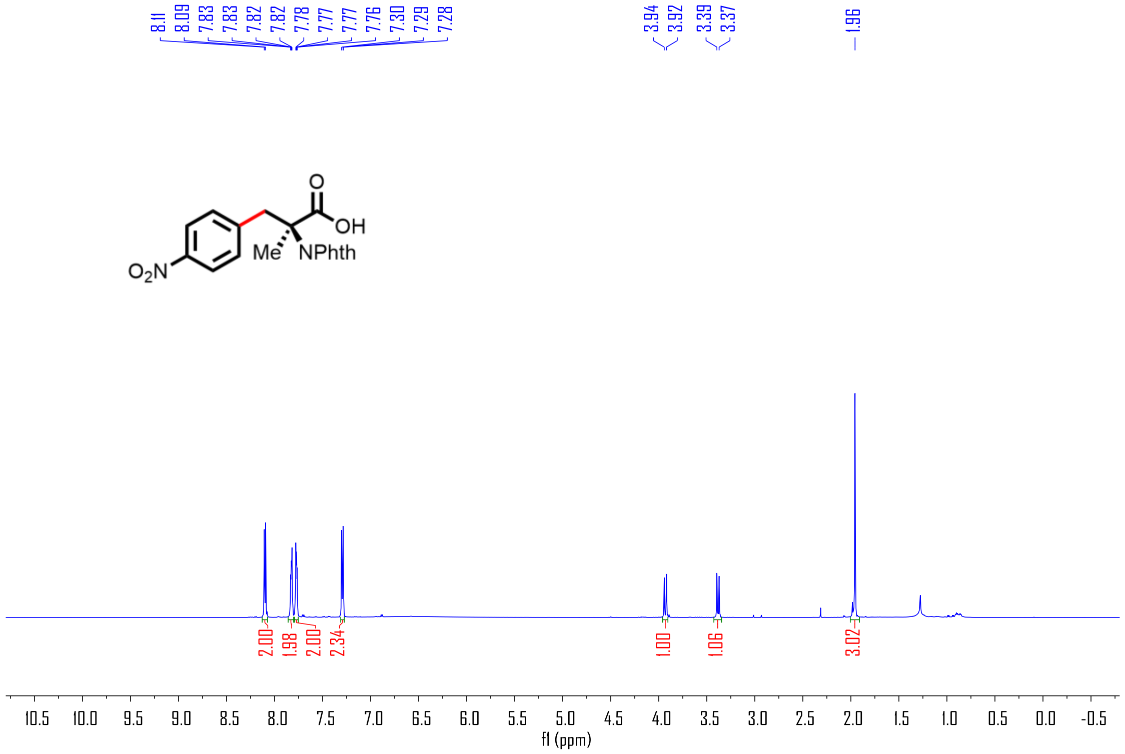
8.11
8.09
7.83
7.83
7.82
7.82
7.78
7.77
7.77
7.76
7.30
7.29
7.28
3.94
3.92
3.39
3.37
1.96
OH
Me
NPhth
O_2N
2.00
1.98
2.00
2.34
1.00
1.06
3.02
fl (ppm)

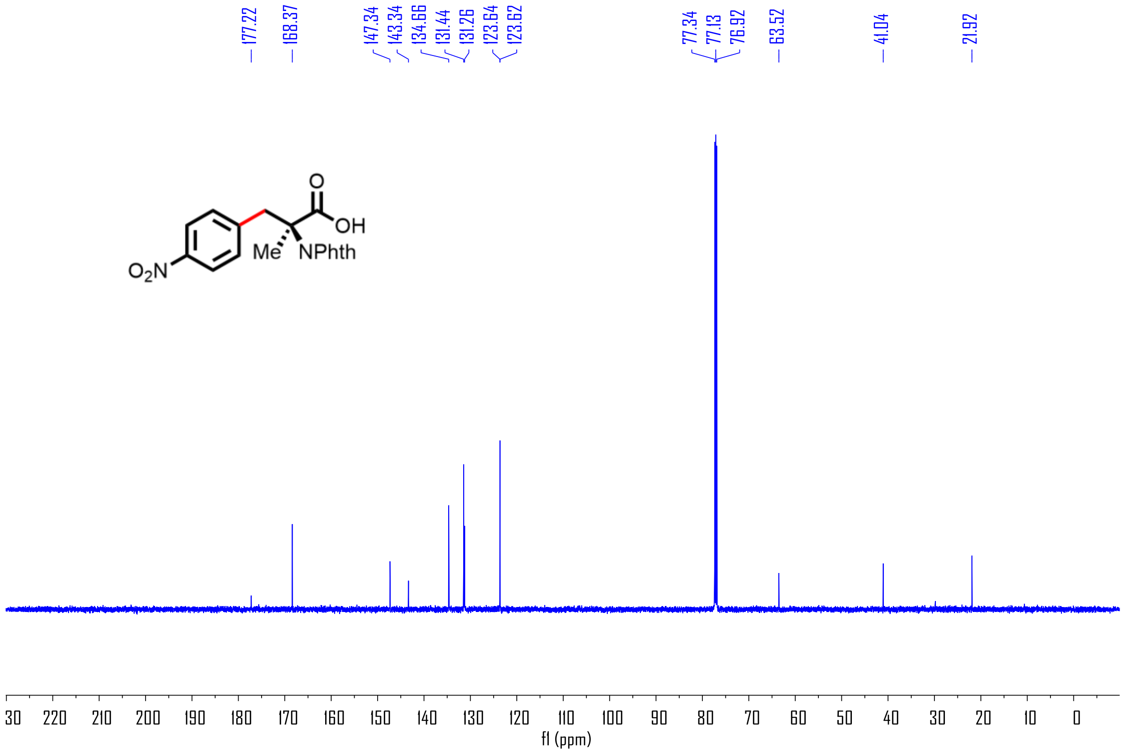
177.22
168.37
147.34
143.34
134.66
131.44
131.26
123.64
123.62
77.34
77.13
76.92
63.52
41.04
21.92
O
OH
O_2N
Me
NPhth
fl (ppm)

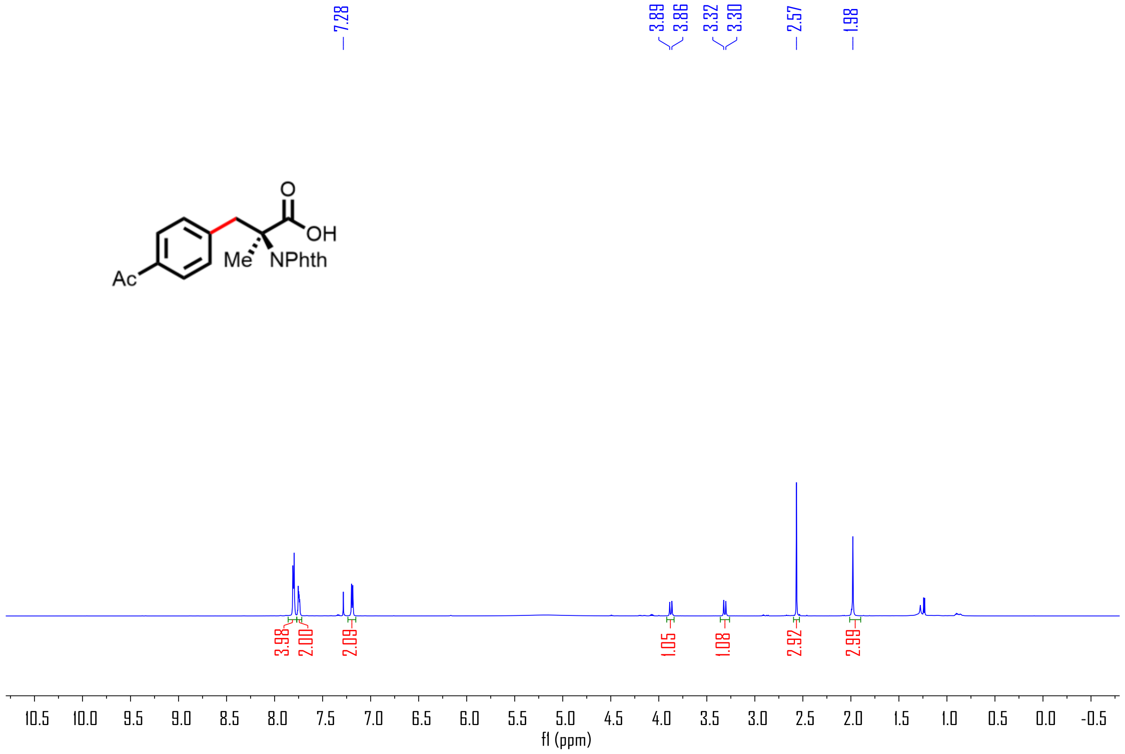
7.28
3.89
3.86
3.32
3.30
2.57
1.98
O
OH
Ac
Me
NPhth
3.98
2.00
2.09
1.05
1.08
2.92
2.99
fl (ppm)

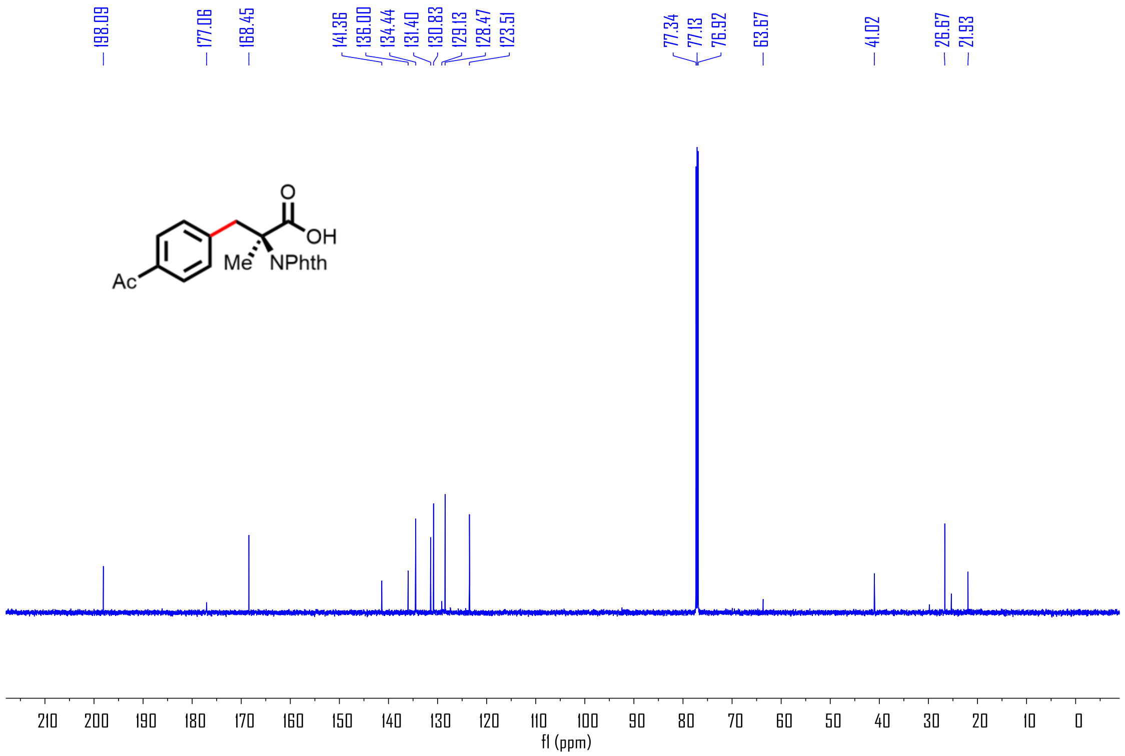
198.09
177.06
168.45
141.36
136.00
134.44
131.40
130.83
129.13
128.47
123.51
77.34
77.13
76.92
63.67
41.02
26.67
21.93
O
OH
Me
NPhth
Ac
fl (ppm)

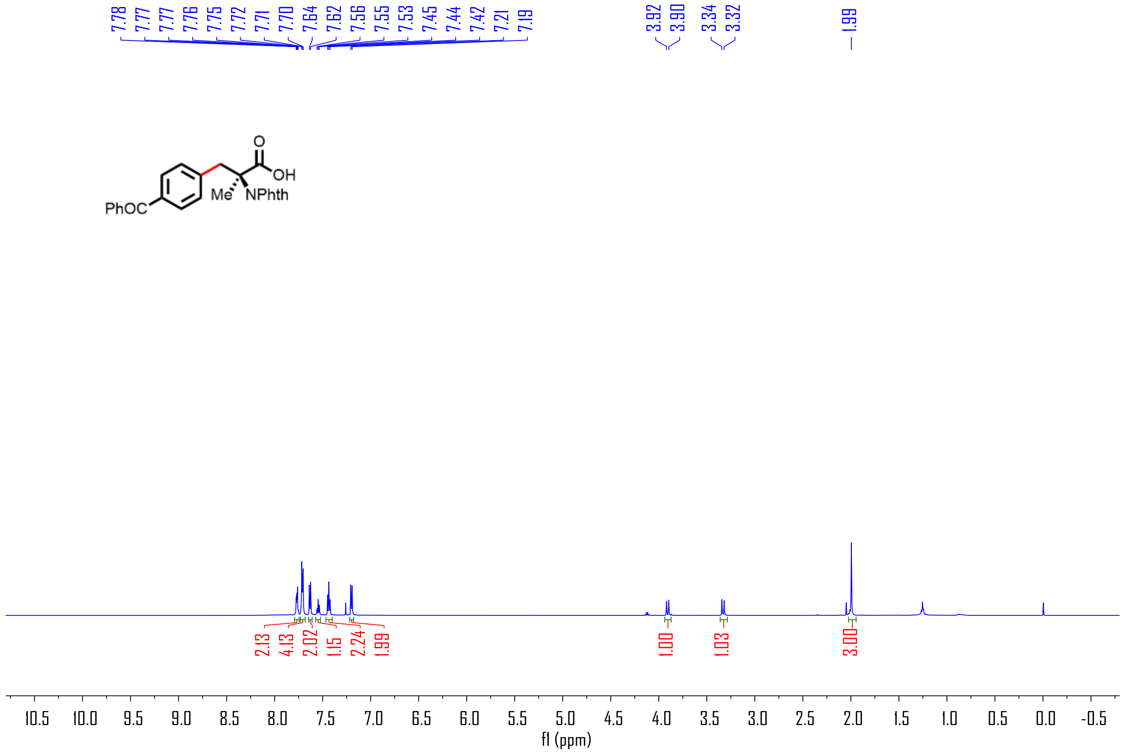
7.78
7.77
7.77
7.76
7.75
7.72
7.71
7.70
7.64
7.62
7.56
7.55
7.53
7.45
7.44
7.42
7.21
7.19
3.92
3.90
3.34
3.32
1.99
O
OH
Me
NPhth
PhOC
2.13
4.13
2.02
1.15
2.24
1.99
1.00
1.03
3.00
fl (ppm)

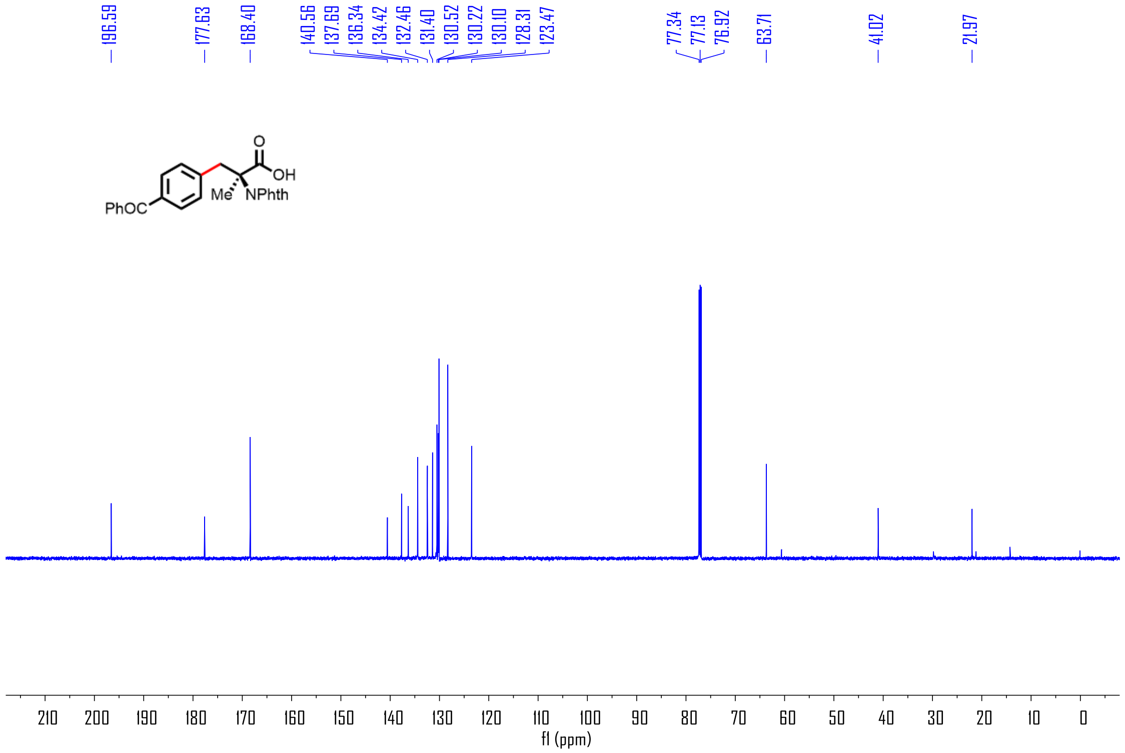
196.59
177.63
168.40
140.56
137.69
136.34
134.42
132.46
131.40
130.52
130.22
130.10
128.31
123.47
77.34
77.13
76.92
63.71
41.02
21.97
O
OH
PhOC
Me
NPhth
f1 (ppm)

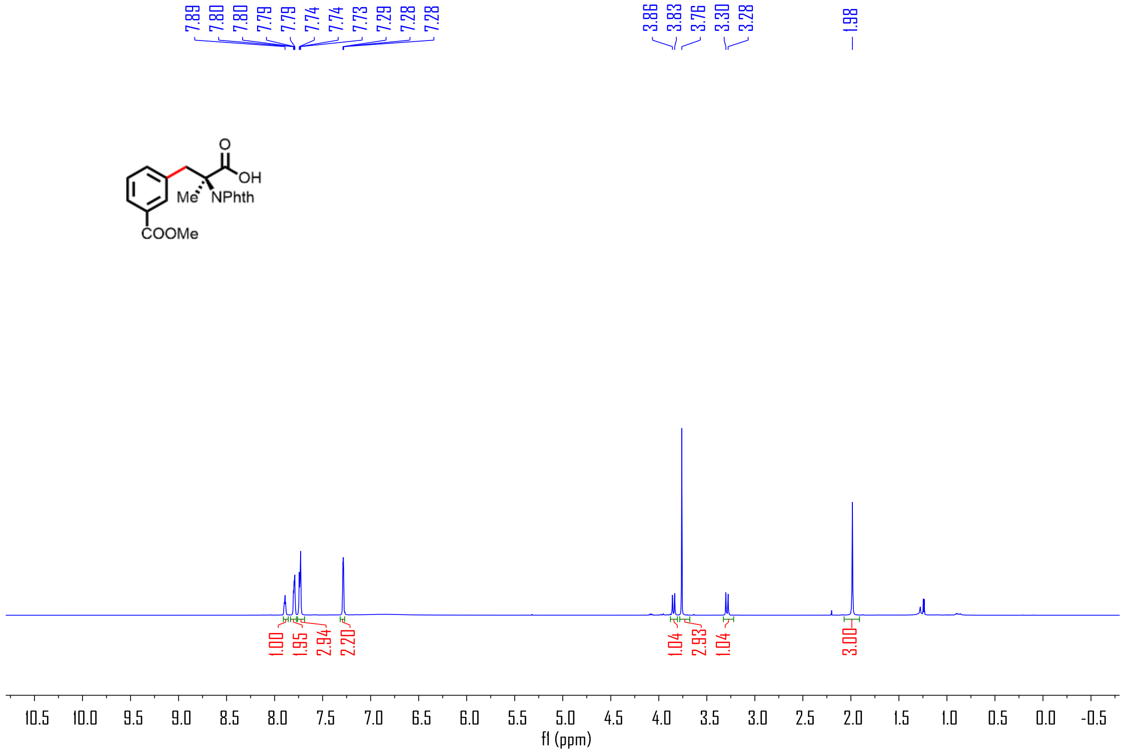
7.89
7.80
7.80
7.79
7.79
7.74
7.74
7.73
7.29
7.28
7.28
3.86
3.83
3.76
3.30
3.28
1.98
O
OH
Me
NPhth
COOMe
1.00
1.95
2.94
2.20
1.04
2.93
1.04
3.00
f1 (ppm)

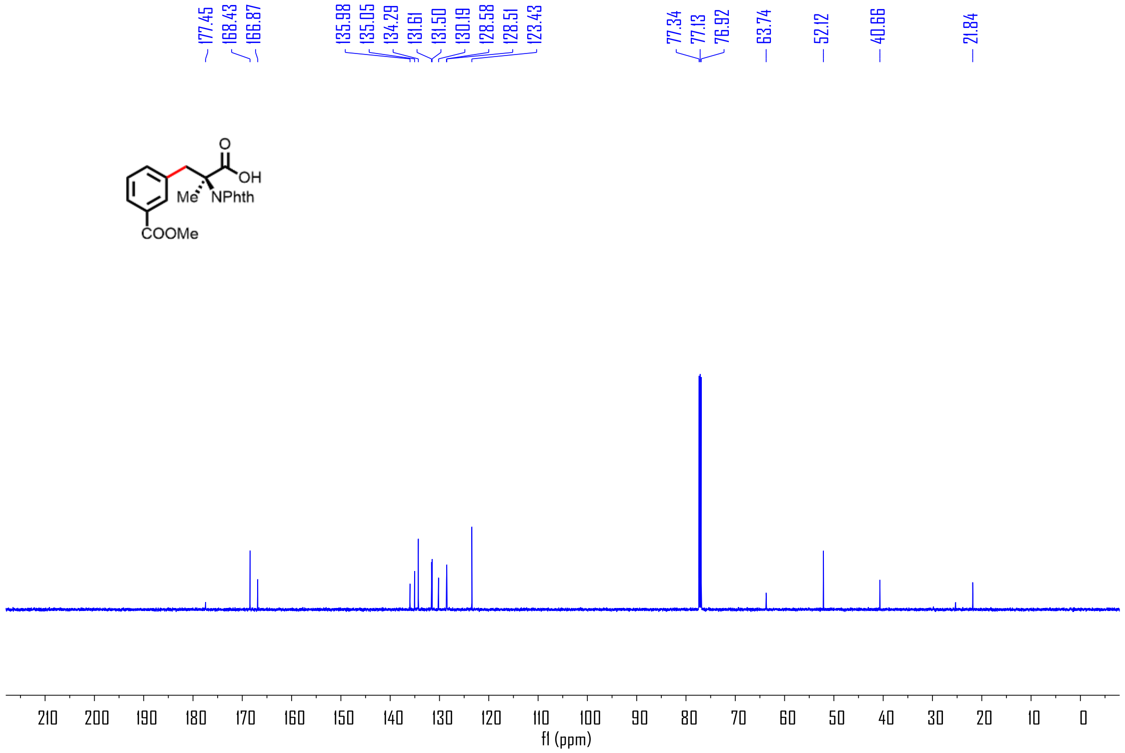
177.45
168.43
166.87
135.98
135.05
134.29
131.61
131.50
130.19
128.58
128.51
123.43
77.34
77.13
76.92
63.74
52.12
40.66
21.84
OH
Me
NPhth
COOMe
f1 (ppm)

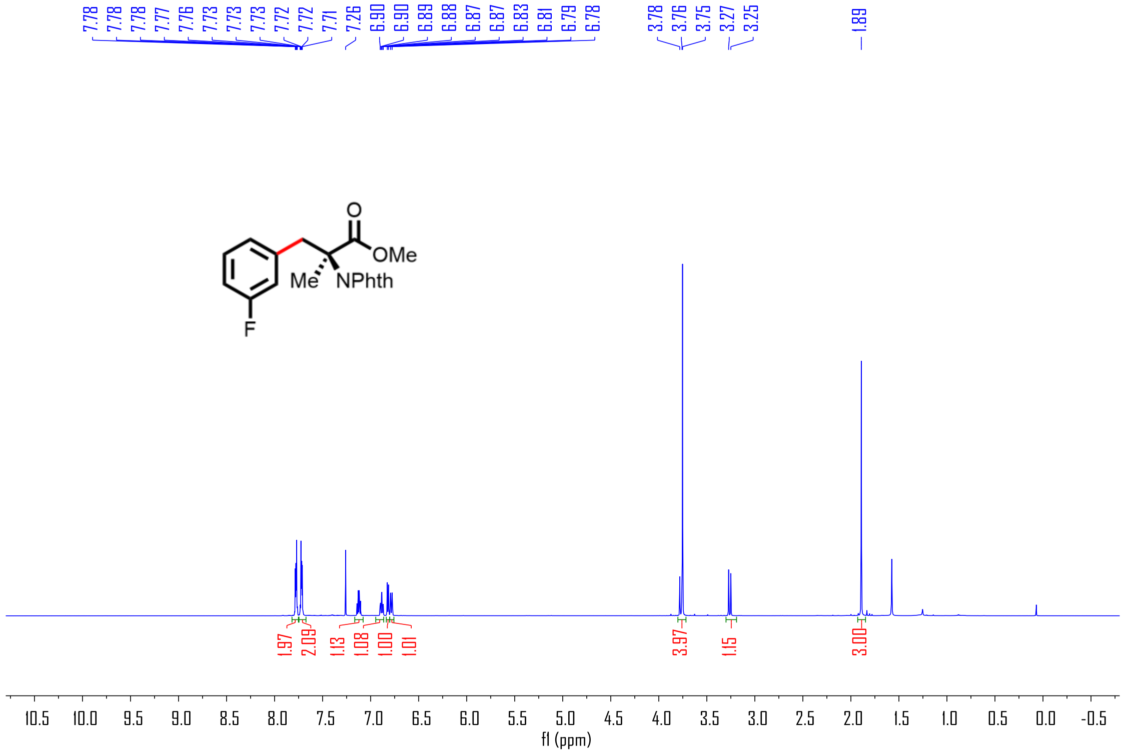
7.78
7.78
7.78
7.77
7.76
7.73
7.73
7.73
7.72
7.72
7.71
7.26
6.90
6.90
6.89
6.88
6.87
6.87
6.83
6.81
6.79
6.78
3.78
3.76
3.75
3.27
3.25
1.89
OMe
Me
NPhth
F
1.97
2.09
1.13
1.08
1.00
1.01
3.97
1.15
3.00
f1 (ppm)

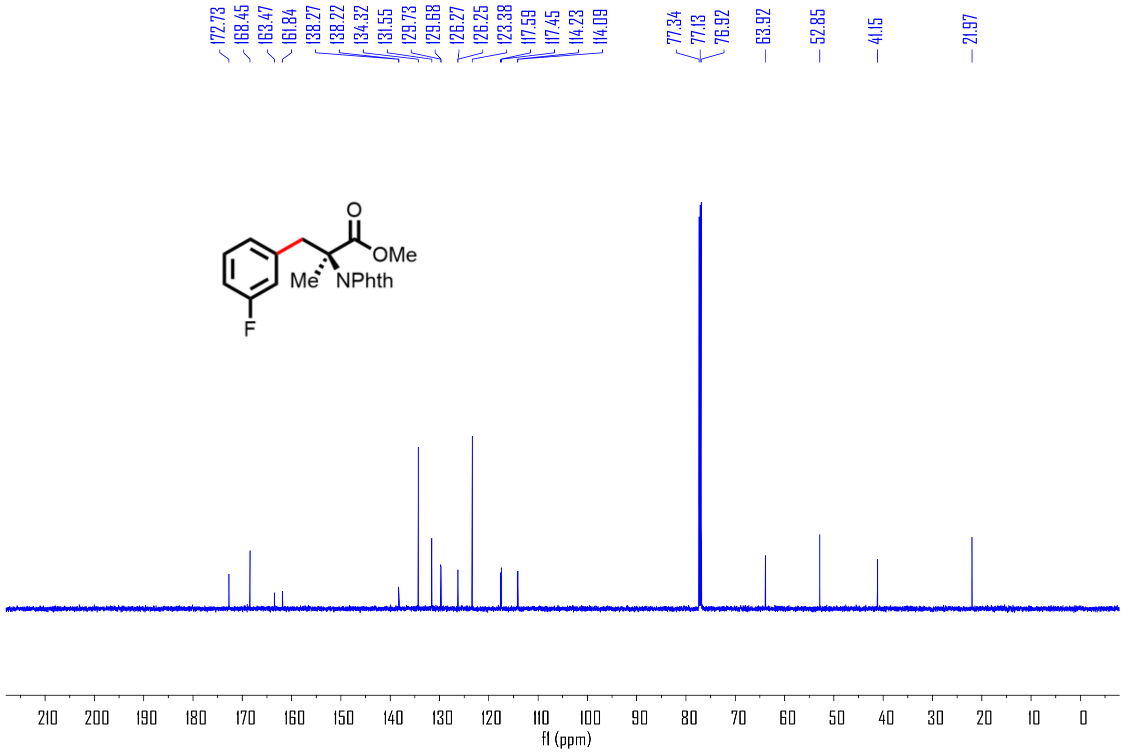
172.73
168.45
163.47
161.84
138.27
138.22
134.32
131.55
129.73
129.68
126.27
126.25
123.38
117.59
117.45
114.23
114.09
77.34
77.13
76.92
63.92
52.85
41.15
21.97
OMe
Me
NPhth
F
f1 (ppm)

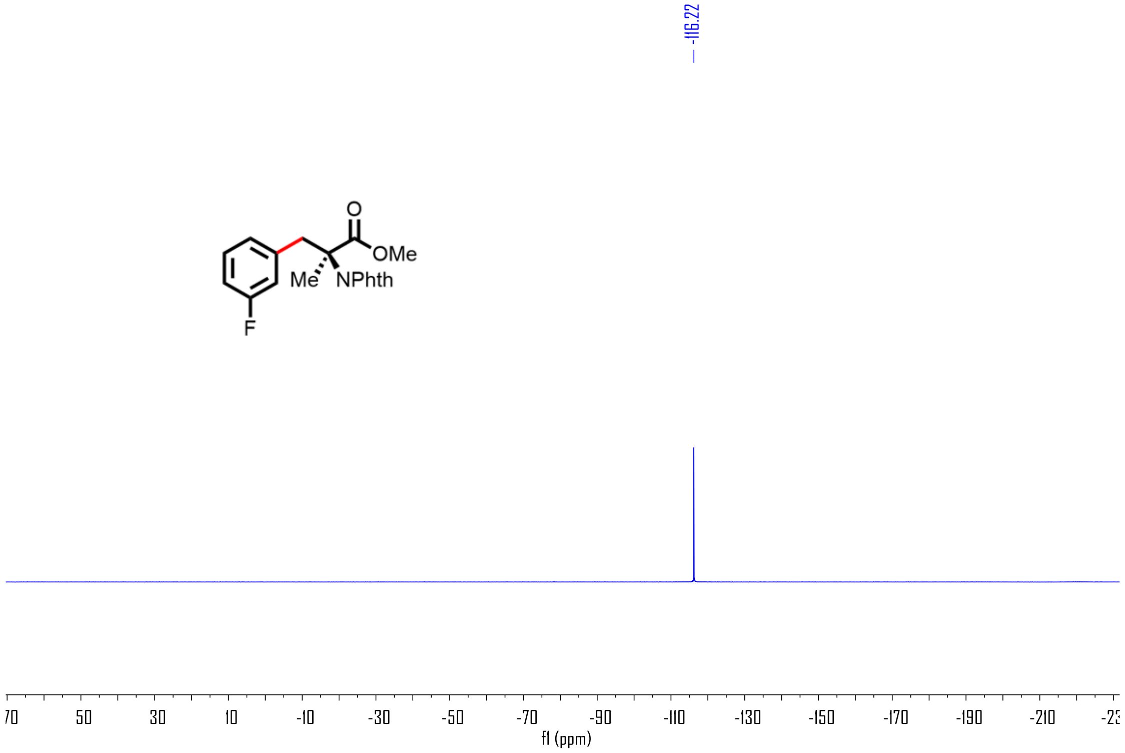
-116.22
OMe
Me
NPhth
F
f1 (ppm)

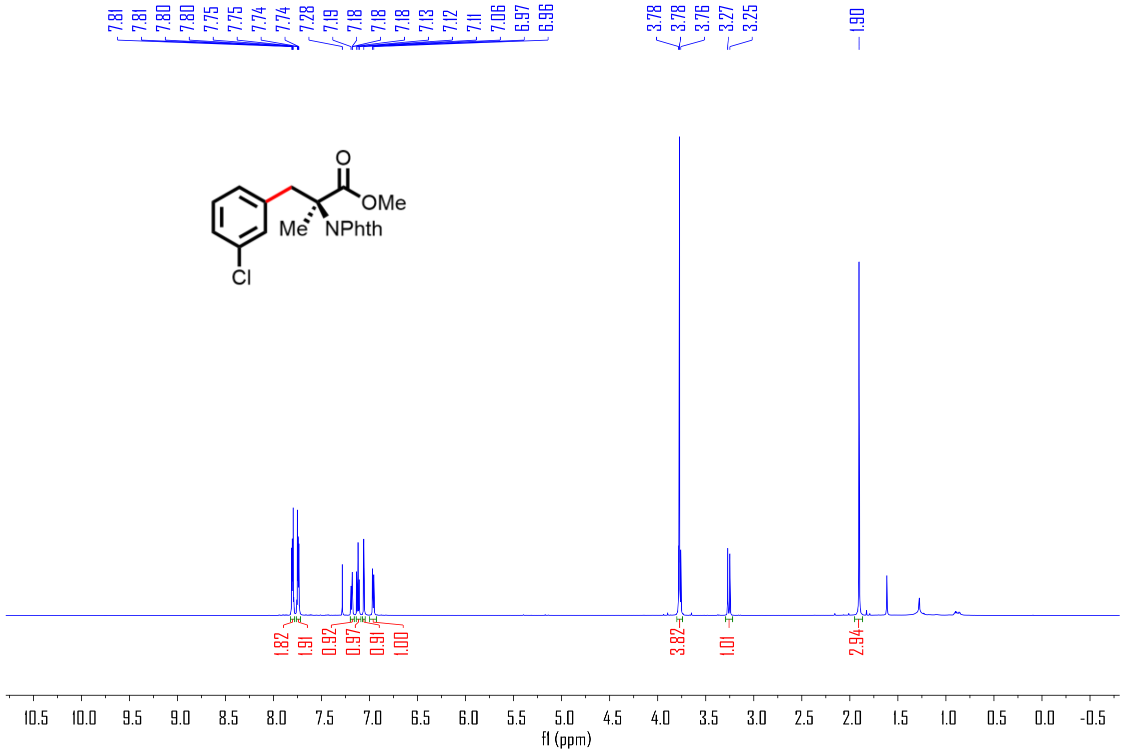

7.81
7.81
7.80
7.80
7.75
7.75
7.74
7.74
7.28
7.19
7.18
7.18
7.18
7.13
7.12
7.11
7.06
6.97
6.96
3.78
3.78
3.76
3.27
3.25
1.90
OMe
Me
NPhth
Cl
1.82
1.91
0.92
0.97
0.91
1.00
3.82
1.01
2.94
fl (ppm)

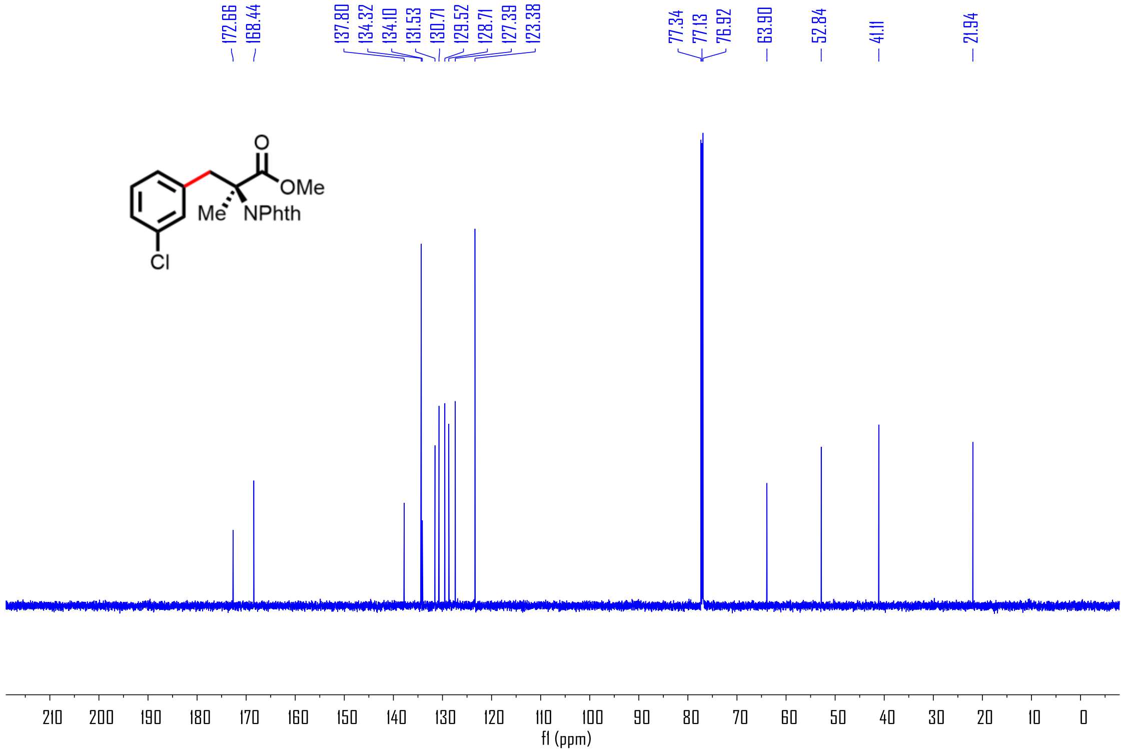

172.66
168.44
137.80
134.32
134.10
131.53
130.71
129.52
128.71
127.39
123.38
77.34
77.13
76.92
63.90
52.84
41.11
21.94
OMe
Me
NPhth
Cl
fl (ppm)

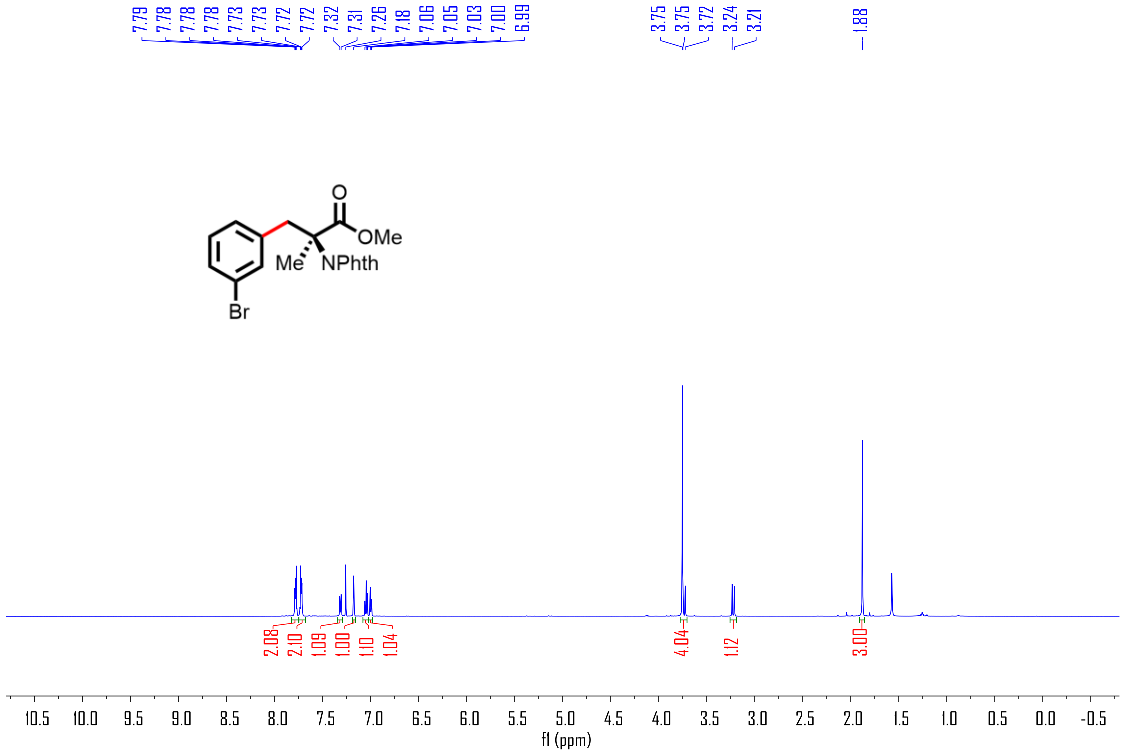
7.79 7.78 7.78 7.78 7.73 7.73 7.72 7.72 7.32 7.31 7.26 7.18 7.06 7.05 7.03 7.00 6.99
3.75 3.75 3.72 3.24 3.21
1.88
O
OMe
Me
NPhth
Br
2.08 2.10 1.09 1.00 1.10 1.04
4.04 1.12 3.00
10.5 10.0 9.5 9.0 8.5 8.0 7.5 7.0 6.5 6.0 5.5 5.0 4.5 4.0 3.5 3.0 2.5 2.0 1.5 1.0 0.5 0.0 -0.5
f1 (ppm)

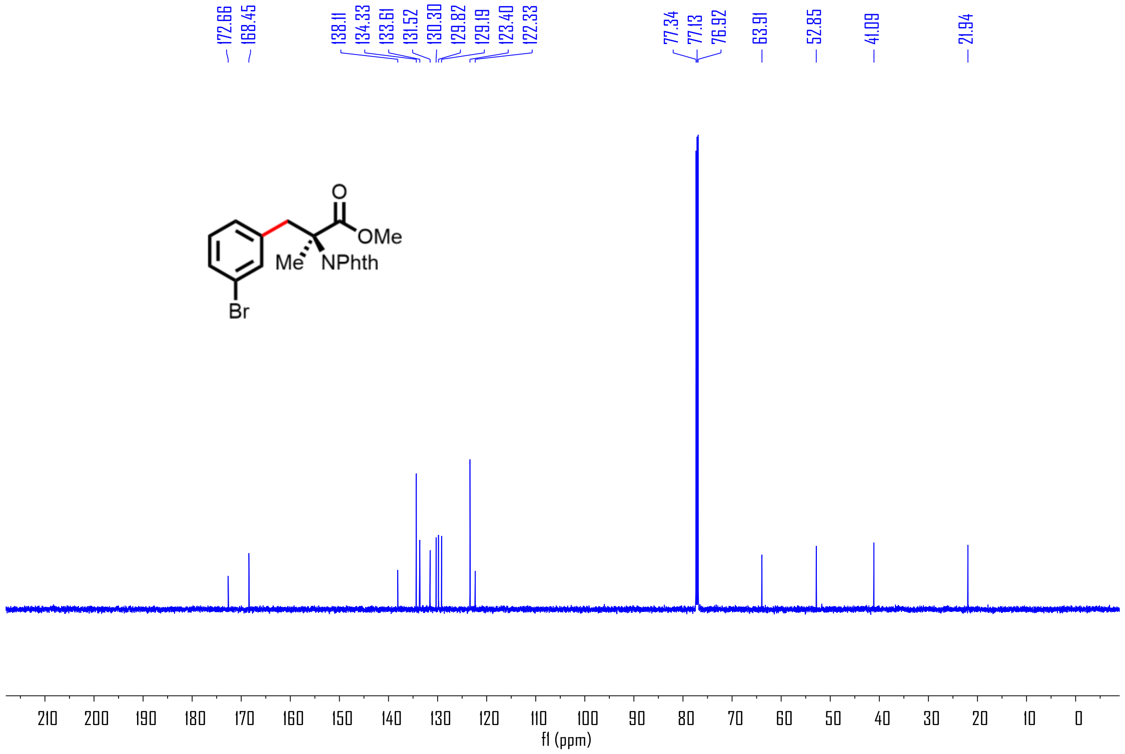
172.66 168.45
138.11 134.33 133.61 131.52 130.30 129.82 129.19 123.40 122.33
77.34 77.13 76.92
63.91
52.85
41.09
21.94
O
OMe
Me
NPhth
Br
210 200 190 180 170 160 150 140 130 120 110 100 90 80 70 60 50 40 30 20 10 0
f1 (ppm)

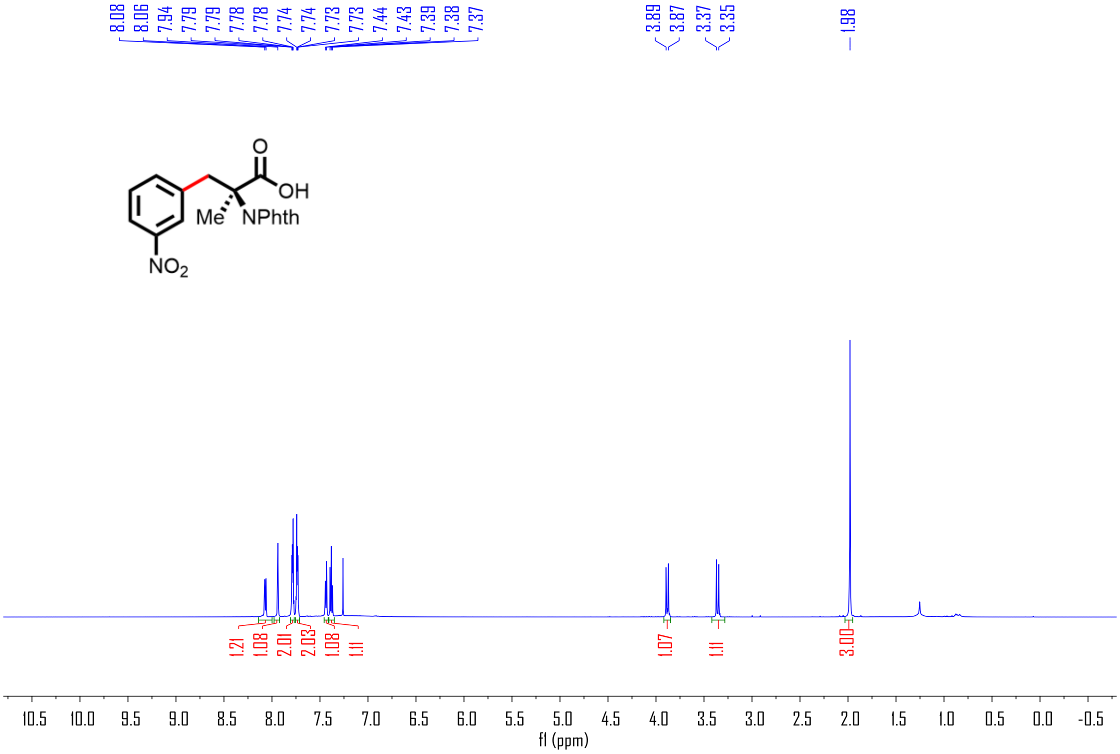
8.08
8.06
7.94
7.79
7.79
7.78
7.78
7.74
7.74
7.73
7.73
7.44
7.43
7.39
7.38
7.37
3.89
3.87
3.37
3.35
1.98
OH
O
Me
NPhth
NO2
1.21
1.08
2.01
2.03
1.08
1.11
1.07
1.11
3.00
10.5 10.0 9.5 9.0 8.5 8.0 7.5 7.0 6.5 6.0 5.5 5.0 4.5 4.0 3.5 3.0 2.5 2.0 1.5 1.0 0.5 0.0 -0.5
f1 (ppm)

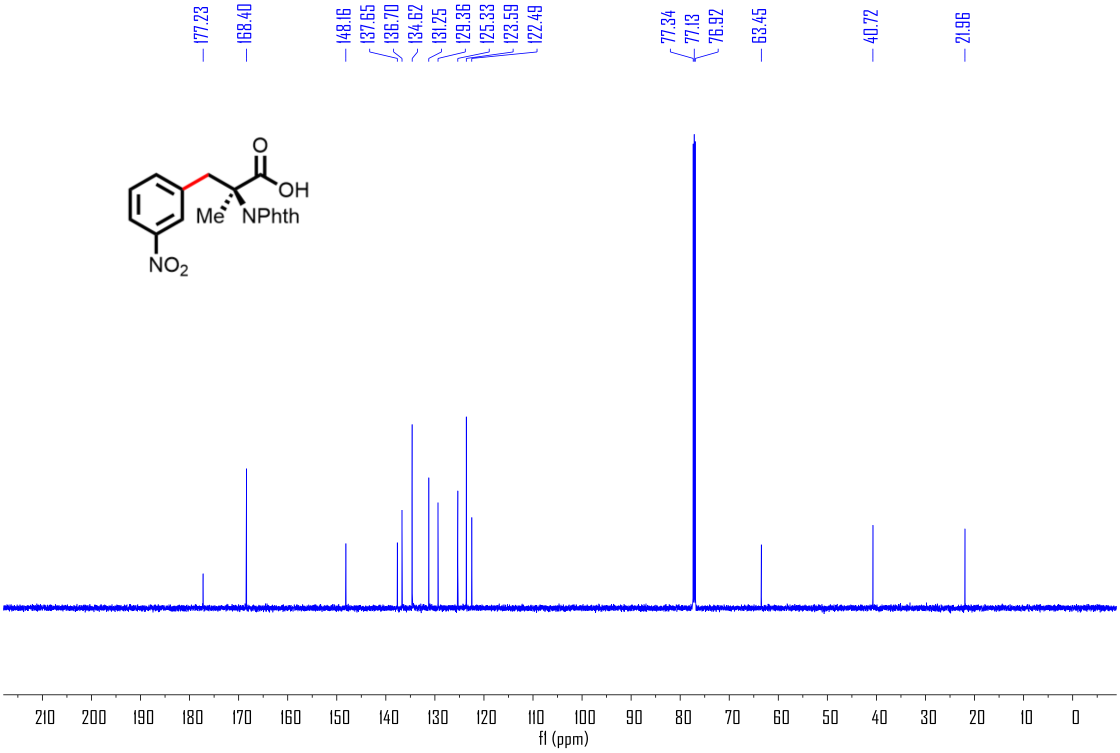
177.23
168.40
148.16
137.65
136.70
134.62
131.25
129.36
125.33
123.59
122.49
77.34
77.13
76.92
63.45
40.72
21.96
OH
O
Me
NPhth
NO2
210 200 190 180 170 160 150 140 130 120 110 100 90 80 70 60 50 40 30 20 10 0
f1 (ppm)

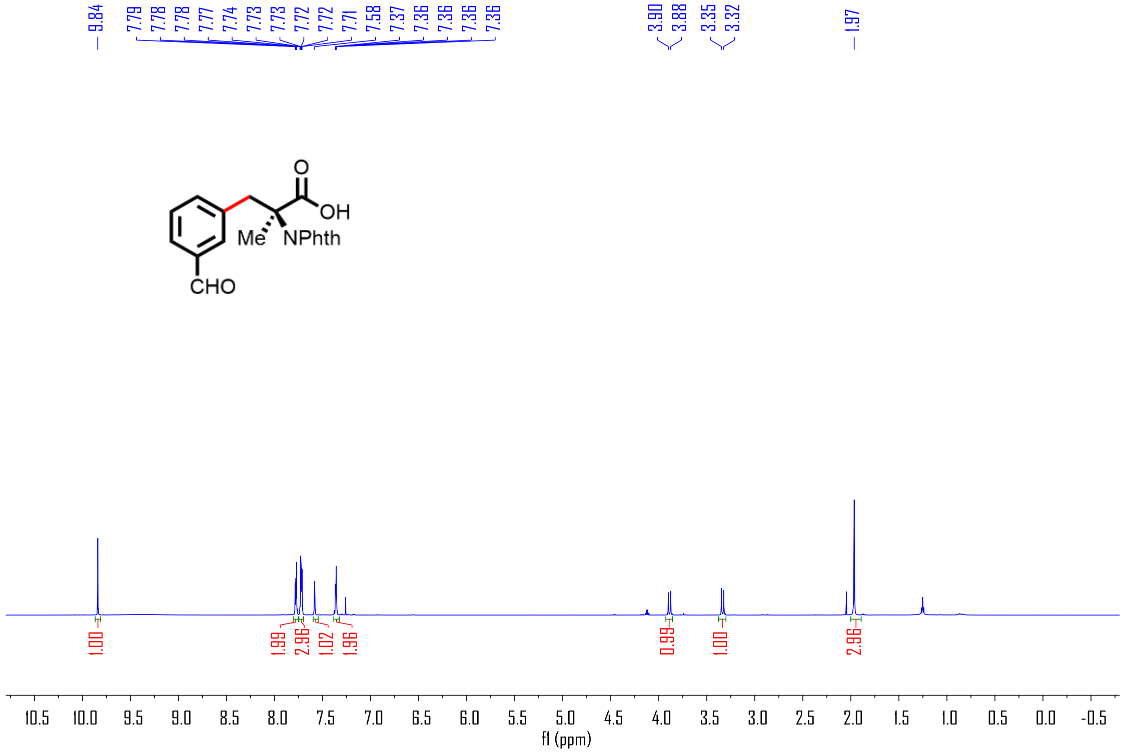
9.84
7.79
7.78
7.78
7.77
7.74
7.73
7.73
7.72
7.72
7.71
7.58
7.37
7.36
7.36
7.36
7.36
3.90
3.88
3.35
3.32
1.97
OH
O
Me
NPhth
CHO
1.00
1.99
2.96
1.02
1.96
0.99
1.00
2.96
10.5 10.0 9.5 9.0 8.5 8.0 7.5 7.0 6.5 6.0 5.5 5.0 4.5 4.0 3.5 3.0 2.5 2.0 1.5 1.0 0.5 0.0 -0.5
f1 (ppm)

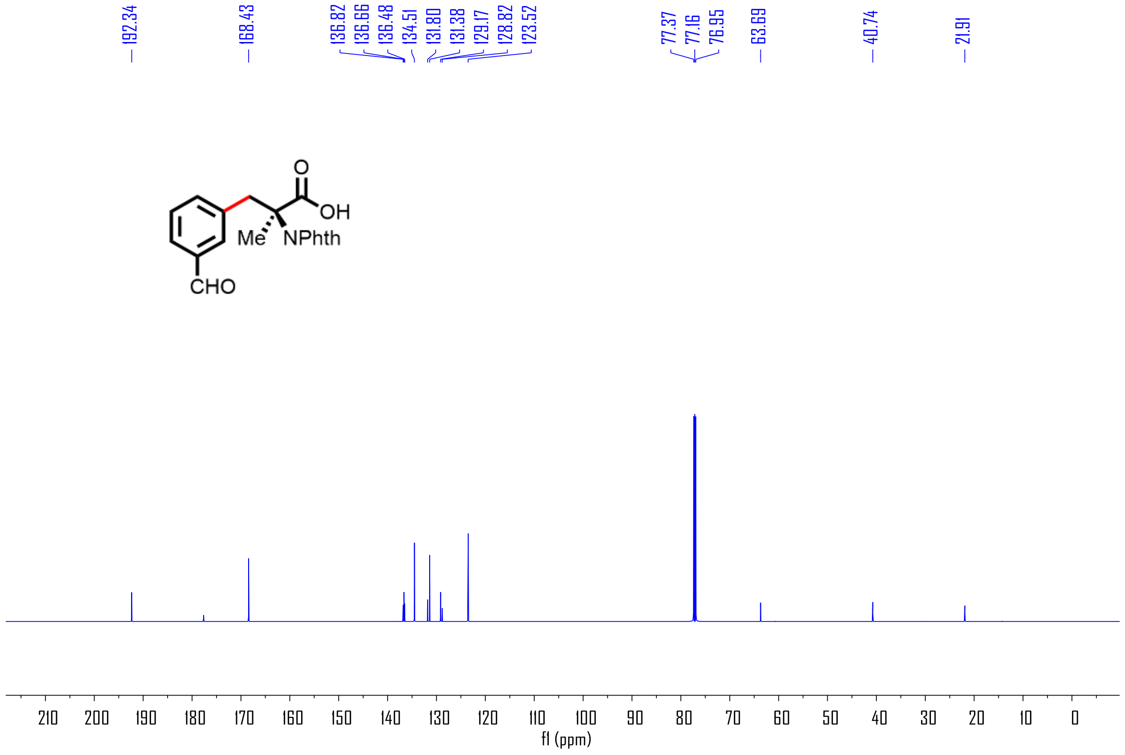
192.34
168.43
136.82
136.66
136.48
134.51
131.80
131.38
129.17
128.82
123.52
77.37
77.16
76.95
63.69
40.74
21.91
OH
O
Me
NPhth
CHO
210 200 190 180 170 160 150 140 130 120 110 100 90 80 70 60 50 40 30 20 10 0
f1 (ppm)

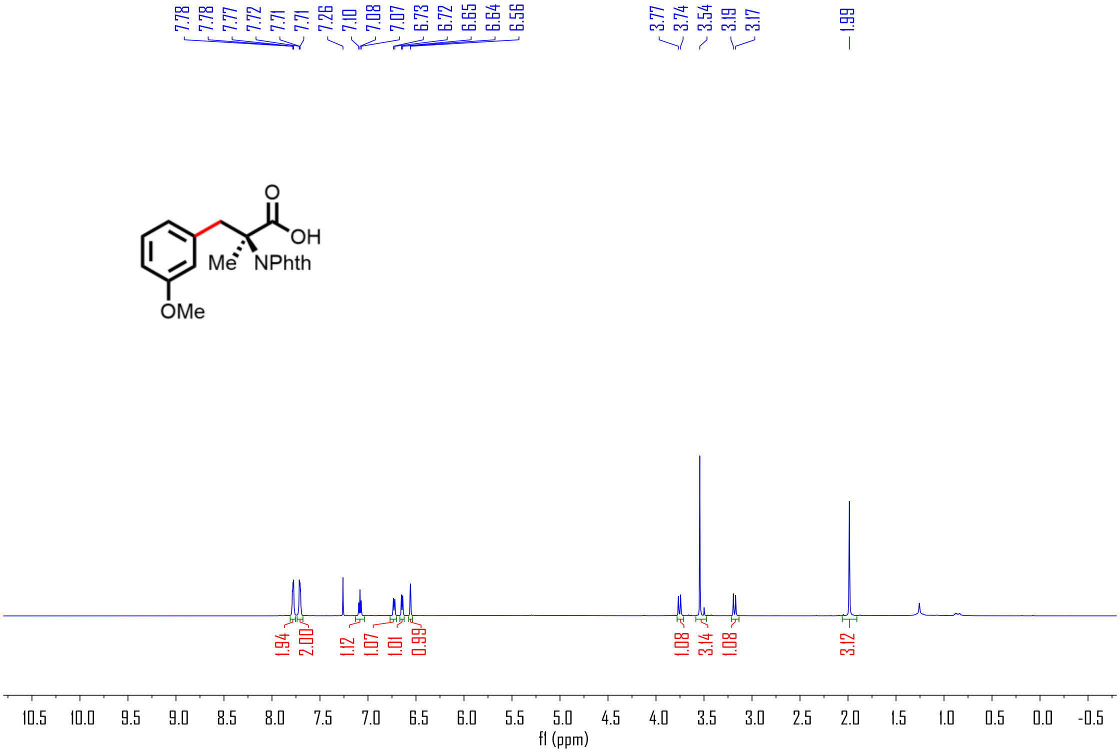
7.78
7.78
7.77
7.72
7.71
7.71
7.26
7.10
7.08
7.07
6.73
6.72
6.65
6.64
6.56
3.77
3.74
3.54
3.19
3.17
1.99
O
OH
Me
NPhth
OMe
1.94
2.00
1.12
1.07
1.01
0.99
1.08
3.14
1.08
3.12
10.5 10.0 9.5 9.0 8.5 8.0 7.5 7.0 6.5 6.0 5.5 5.0 4.5 4.0 3.5 3.0 2.5 2.0 1.5 1.0 0.5 0.0 -0.5
f1 (ppm)

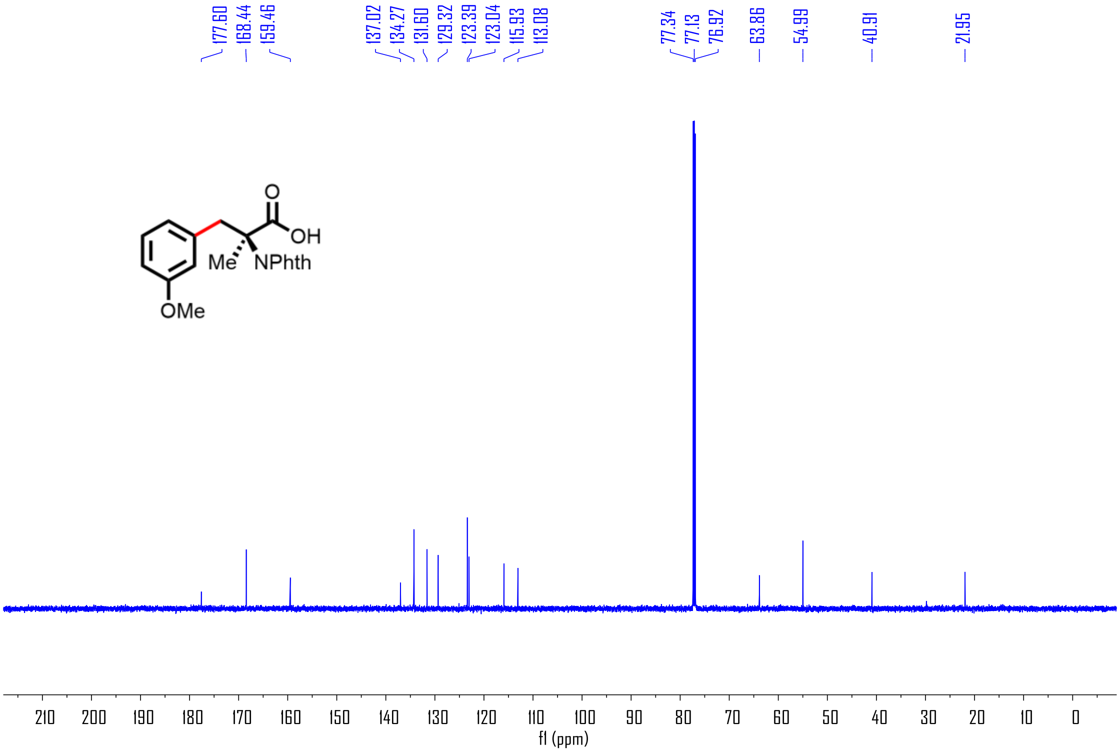
177.60
168.44
159.46
137.02
134.27
131.60
129.32
123.39
123.04
115.93
113.08
77.34
77.13
76.92
63.86
54.99
40.91
21.95
O
OH
Me
NPhth
OMe
210 200 190 180 170 160 150 140 130 120 110 100 90 80 70 60 50 40 30 20 10 0
f1 (ppm)

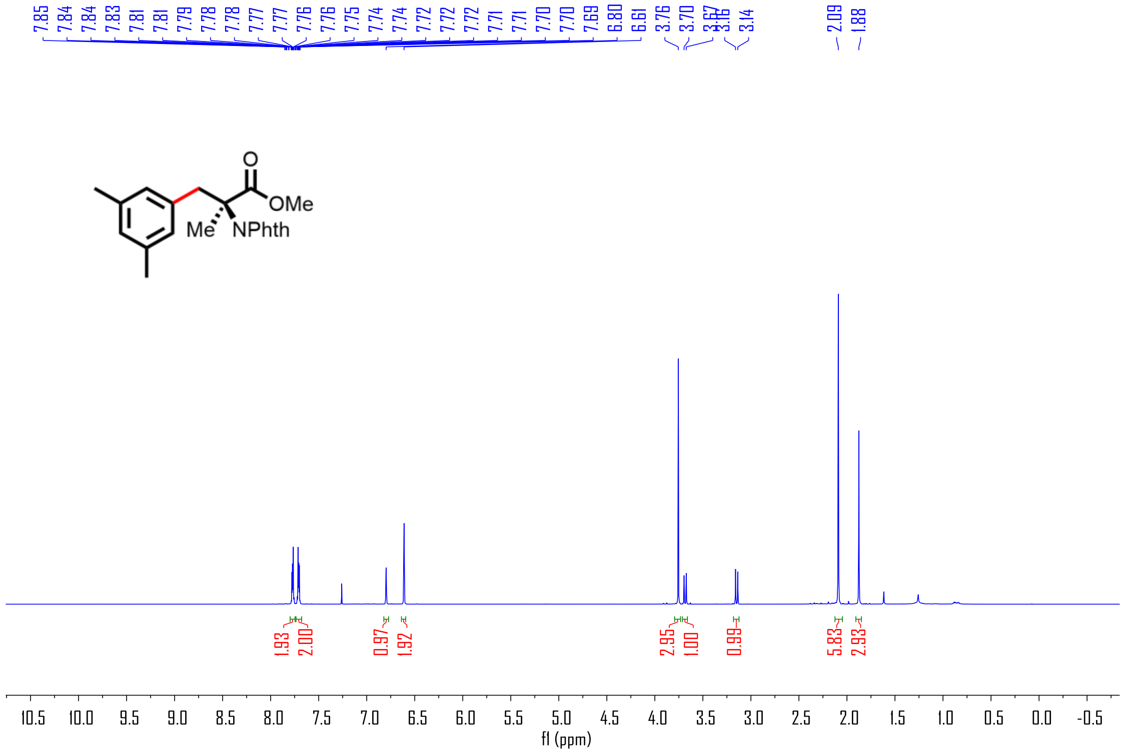
OMe
Me
NPhth
fl (ppm)

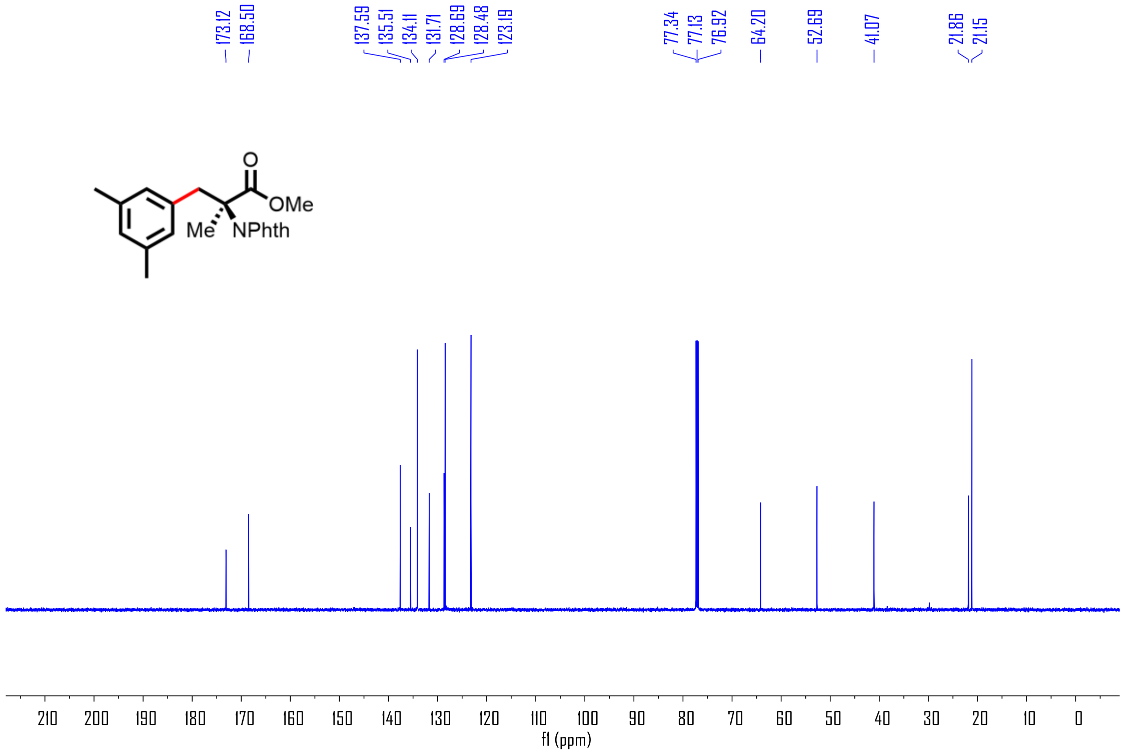
OMe
Me
NPhth
fl (ppm)

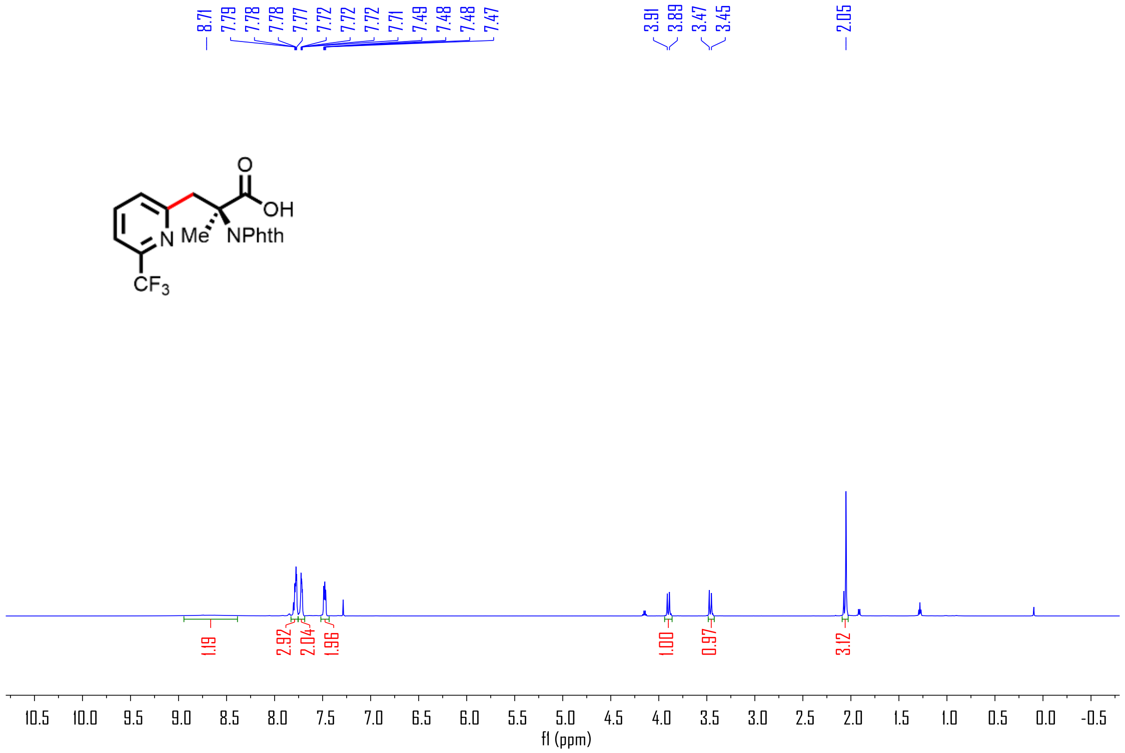
OH
N
Me
NPhth
CF3
8.71
7.79
7.78
7.78
7.77
7.72
7.72
7.72
7.71
7.49
7.48
7.48
7.47
3.91
3.89
3.47
3.45
2.05
1.19
2.92
2.04
1.96
1.00
0.97
3.12
fl (ppm)

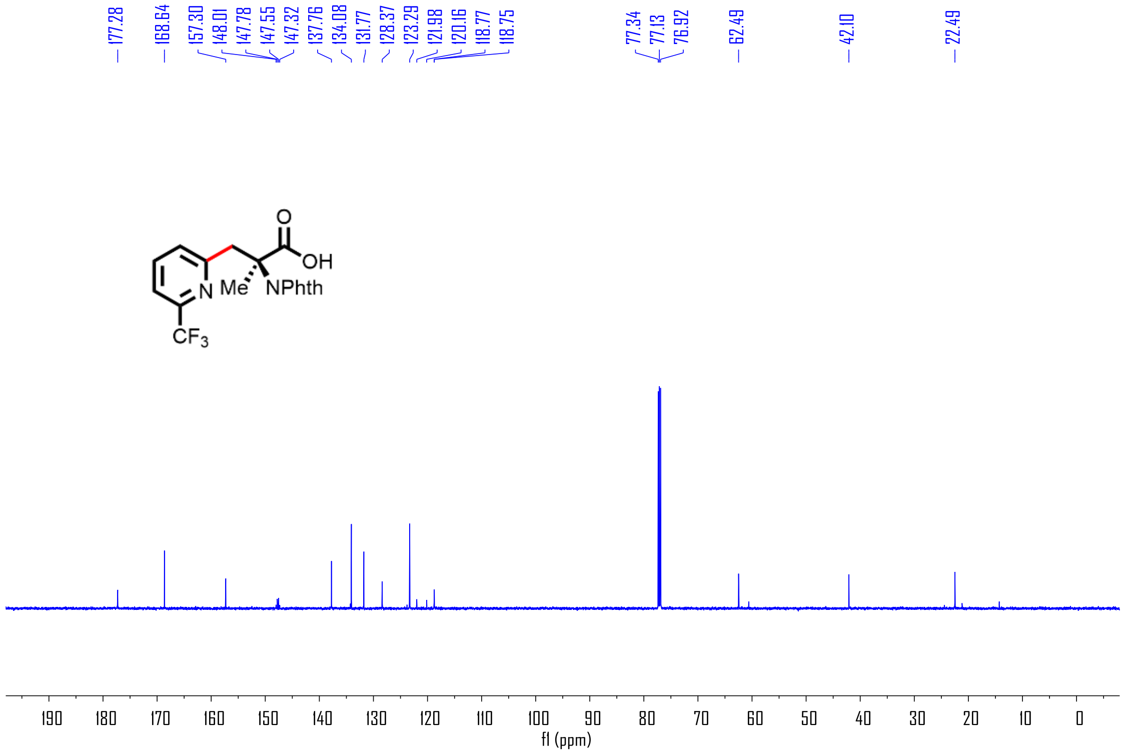
OH
N
Me
NPhth
CF3
177.28
168.64
157.30
148.01
147.78
147.55
147.32
137.76
134.08
131.77
128.37
123.29
121.98
120.16
118.77
118.75
77.34
77.13
76.92
62.49
42.10
22.49
fl (ppm)

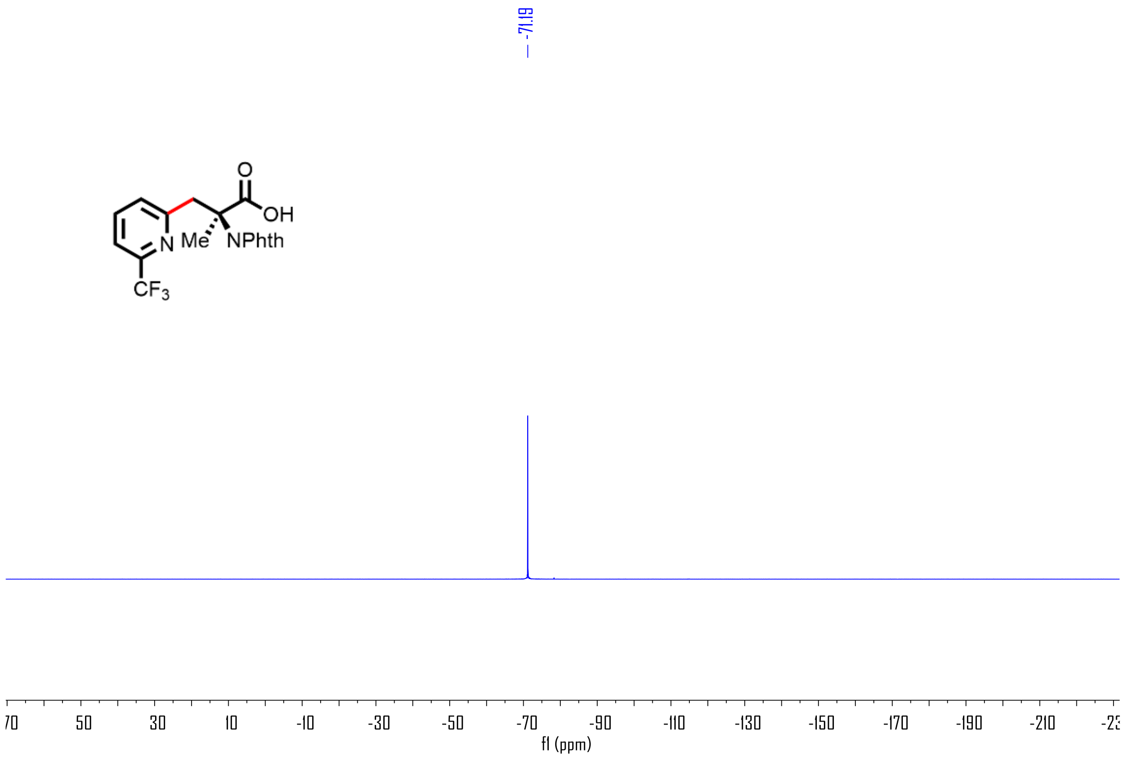

-71.19
O
OH
N
Me
NPhth
CF_3
70 50 30 10 -10 -30 -50 -70 -90 -110 -130 -150 -170 -190 -210
f1 (ppm)

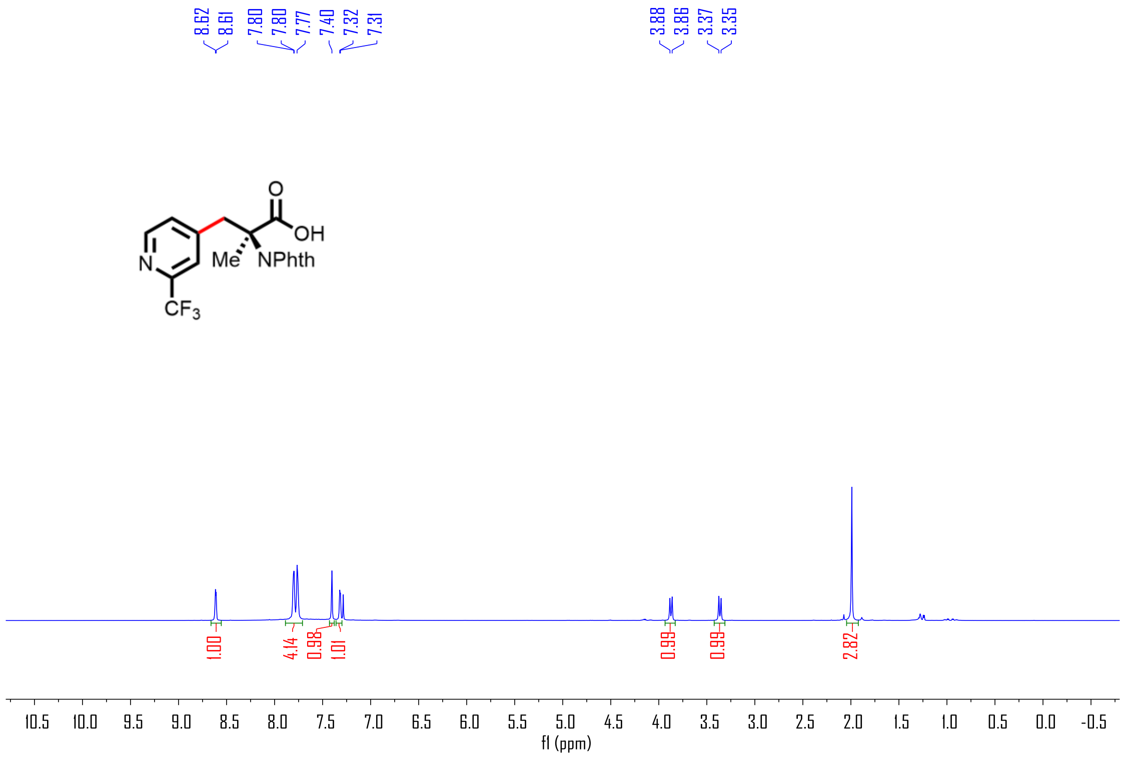

8.62
8.61
7.80
7.80
7.77
7.40
7.32
7.31
3.88
3.86
3.37
3.35
O
OH
N
Me
NPhth
CF_3
1.00
4.14
0.98
1.01
0.99
0.99
2.82
10.5 10.0 9.5 9.0 8.5 8.0 7.5 7.0 6.5 6.0 5.5 5.0 4.5 4.0 3.5 3.0 2.5 2.0 1.5 1.0 0.5 0.0 -0.5
f1 (ppm)

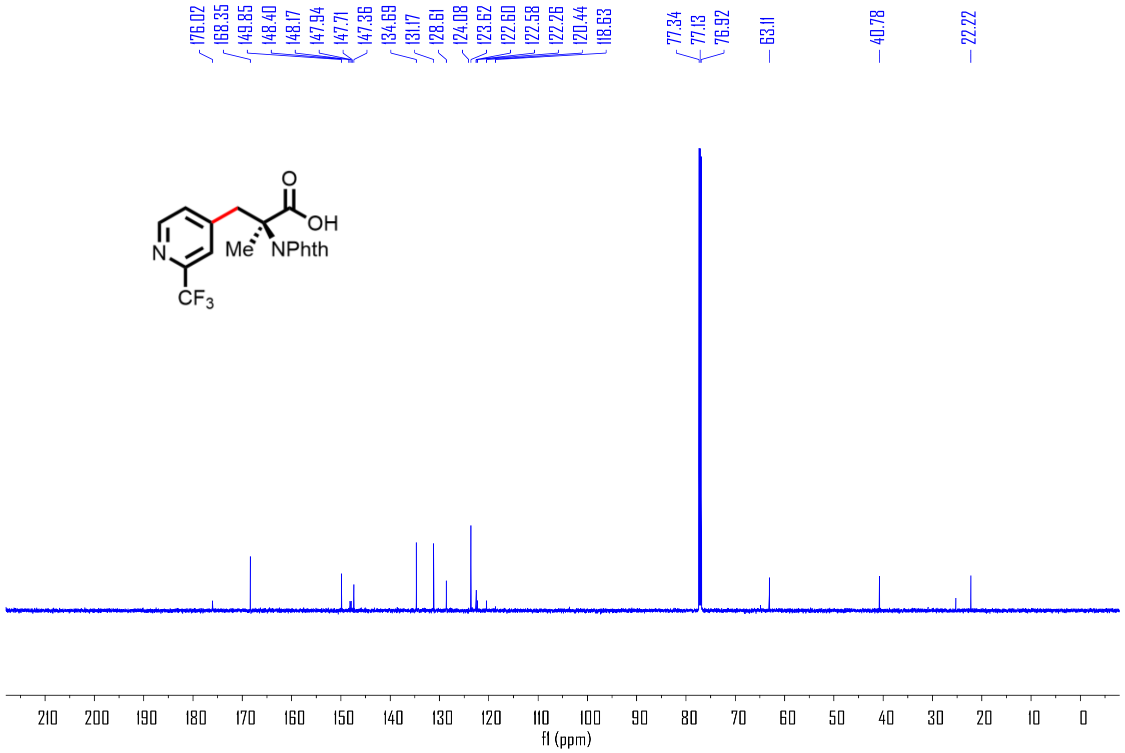
176.02
168.35
149.85
148.40
148.17
147.94
147.71
147.36
134.69
131.17
128.61
124.08
123.62
122.60
122.58
122.26
120.44
118.63
77.34
77.13
76.92
63.11
40.78
22.22
Me
NPhth
OH
O
N
CF3
f1 (ppm)

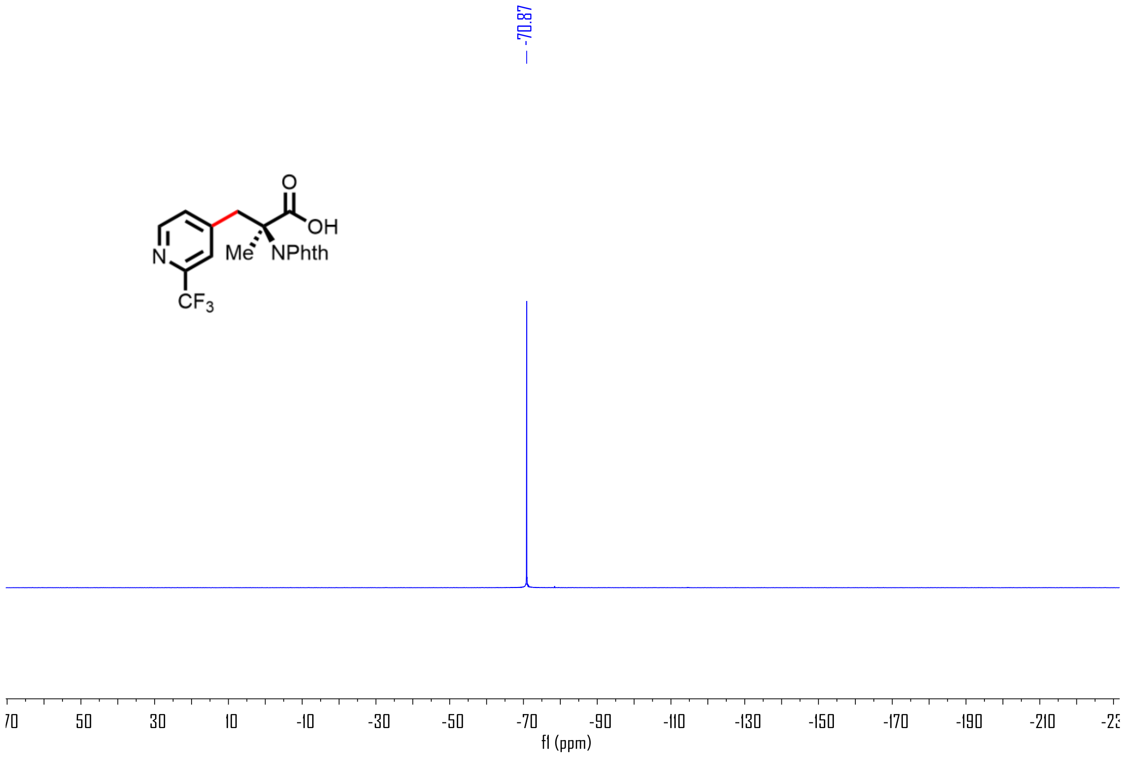
-70.87
Me
NPhth
OH
O
N
CF3
f1 (ppm)

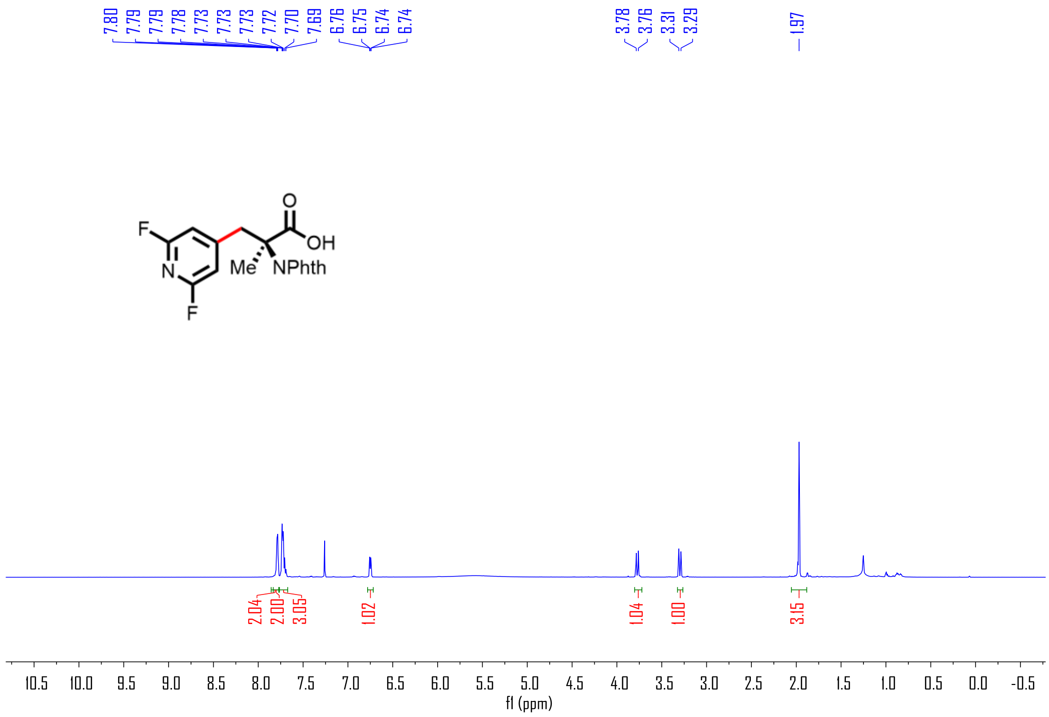

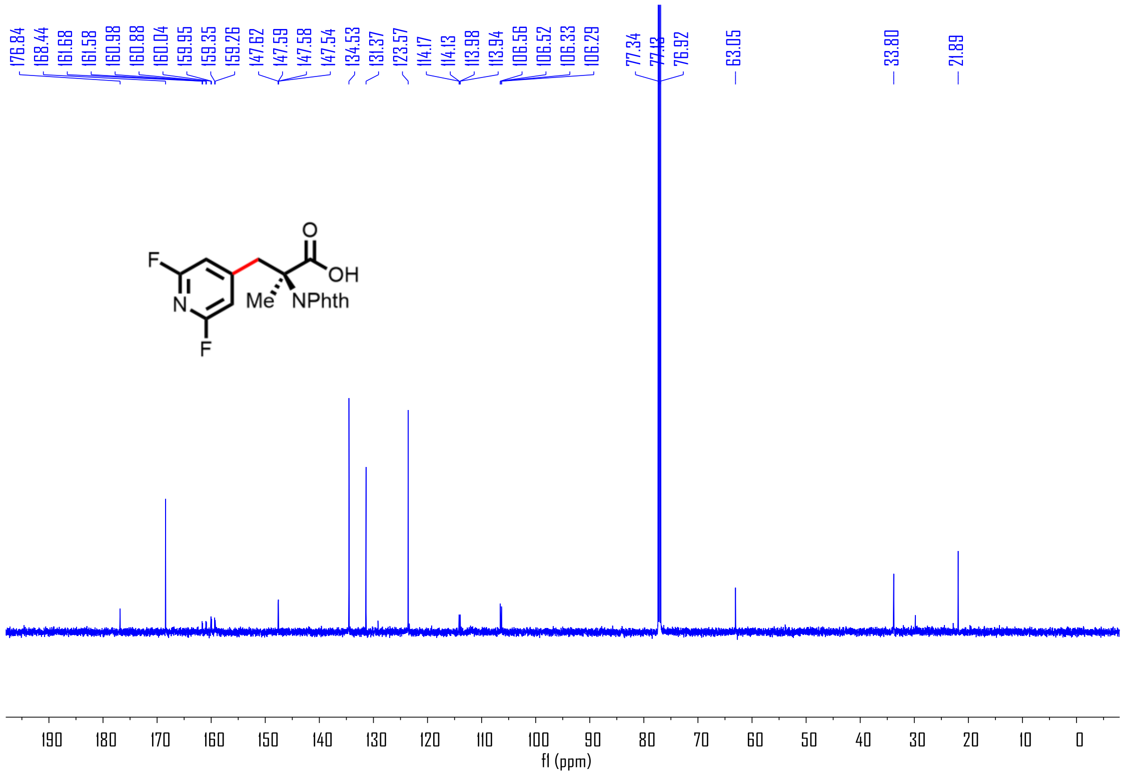

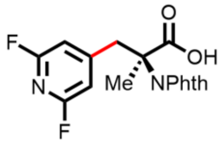

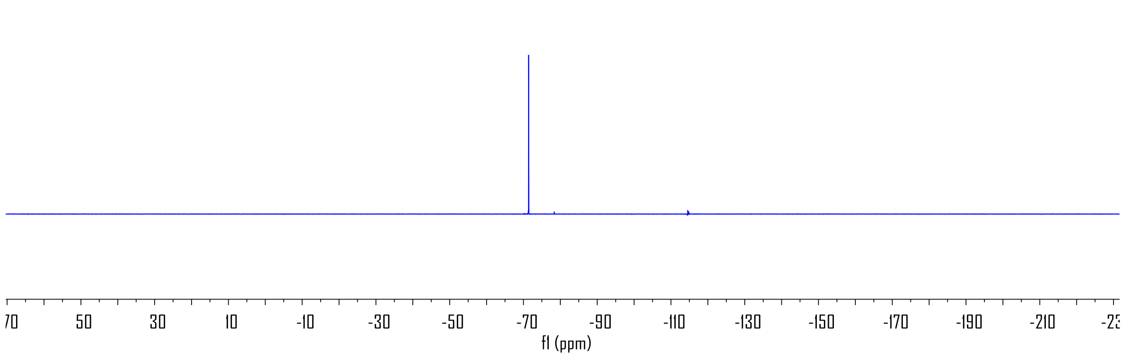

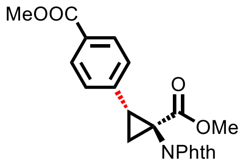

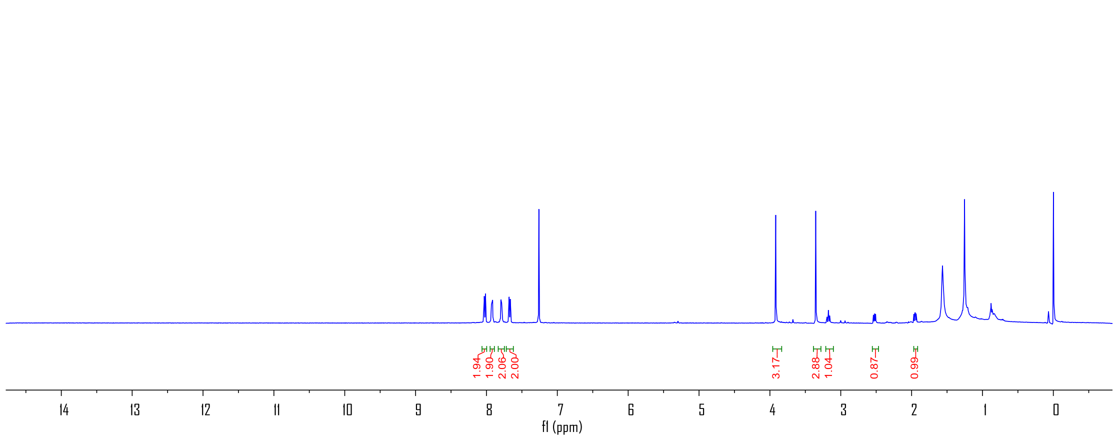

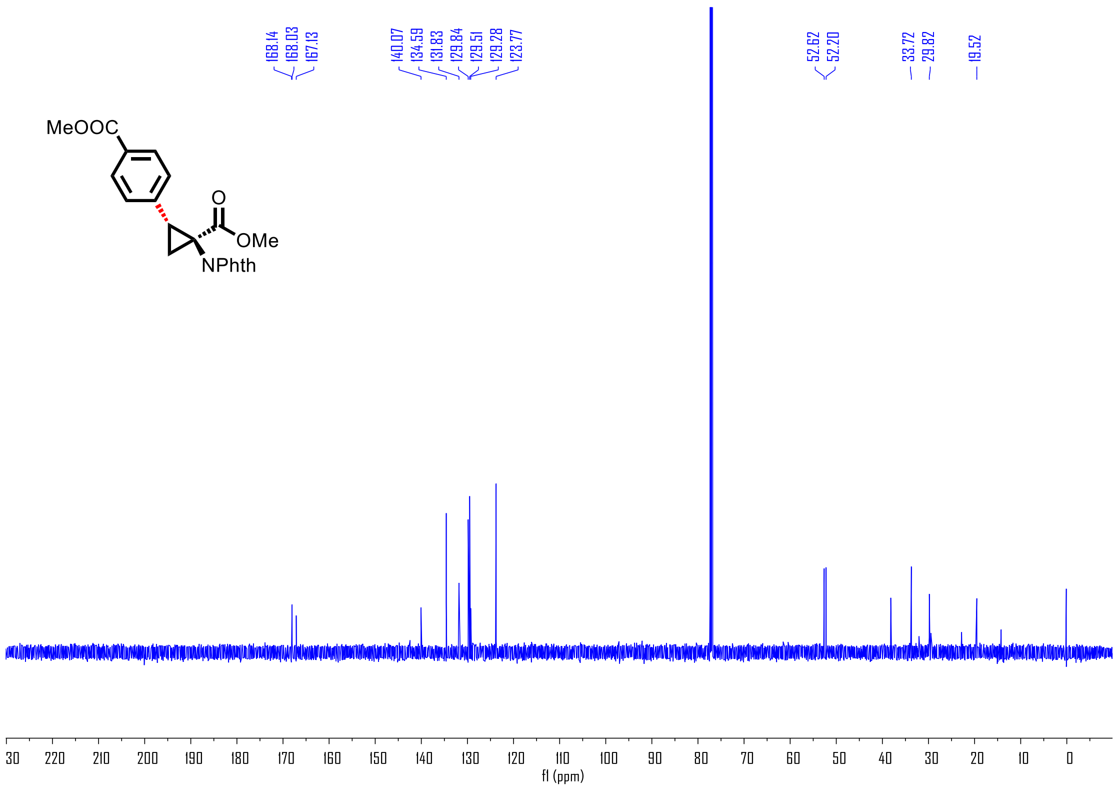
MeOOC
O
OMe
NPhth
168.14
168.03
167.13
140.07
134.59
131.83
129.84
129.51
129.28
123.77
52.62
52.20
33.72
29.82
19.52
f1 (ppm)

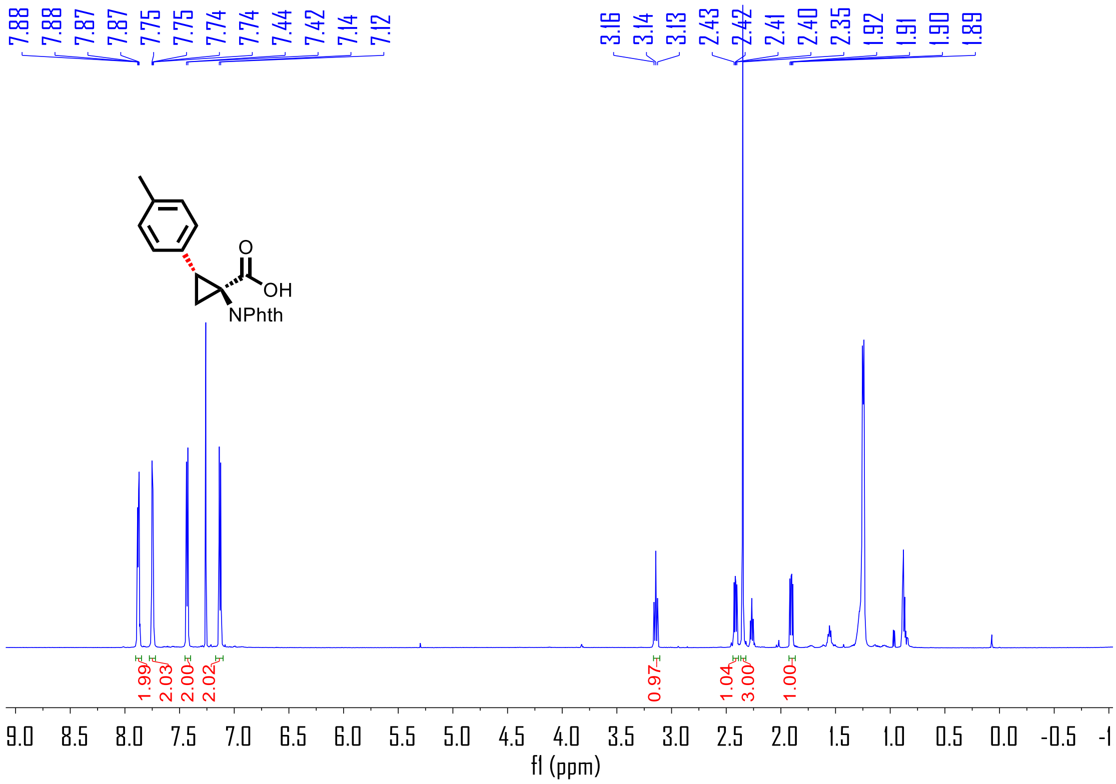
O
OH
NPhth
7.88
7.88
7.87
7.87
7.75
7.75
7.74
7.74
7.44
7.42
7.14
7.12
3.16
3.14
3.13
2.43
2.42
2.41
2.40
2.35
1.92
1.91
1.90
1.89
1.99
2.03
2.00
2.02
0.97
1.04
3.00
1.00
f1 (ppm)

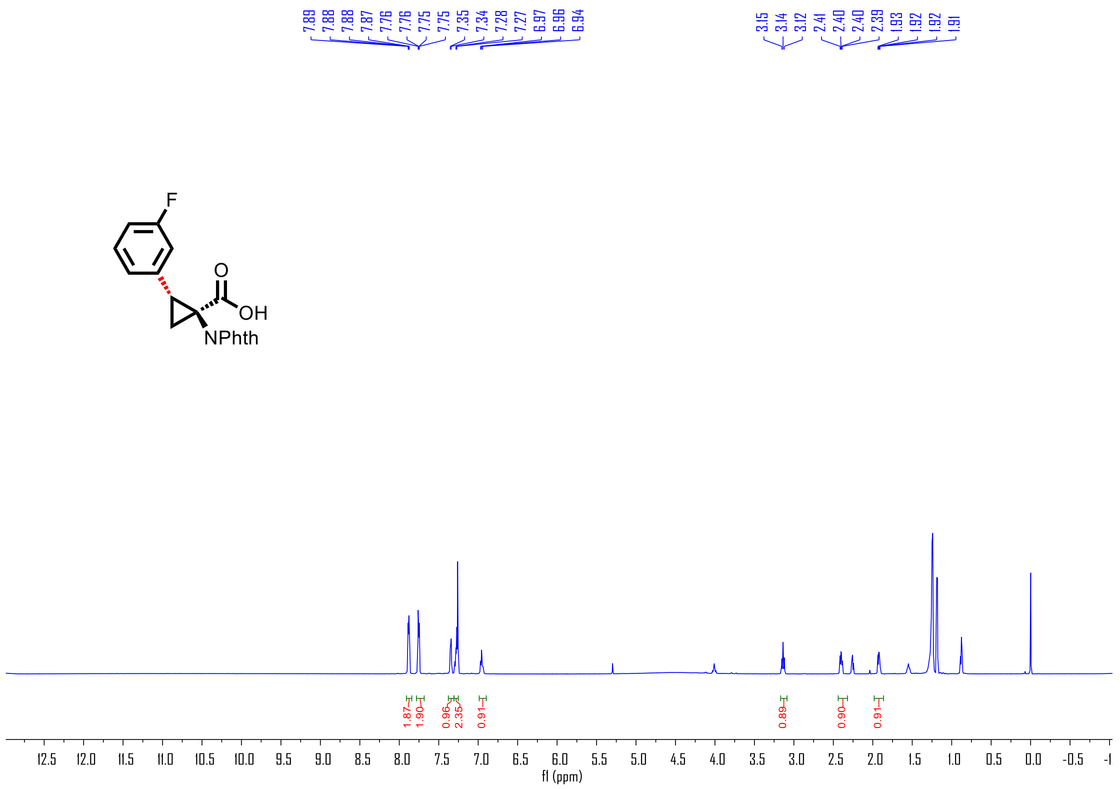
7.89
7.88
7.88
7.87
7.76
7.76
7.75
7.75
7.35
7.34
7.28
7.27
6.97
6.96
6.94
3.15
3.14
3.12
2.41
2.40
2.40
2.39
1.93
1.92
1.92
1.91
F
O
OH
NPhth
1.87
1.90
0.96
2.35
0.91
0.89
0.90
0.91
fl (ppm)

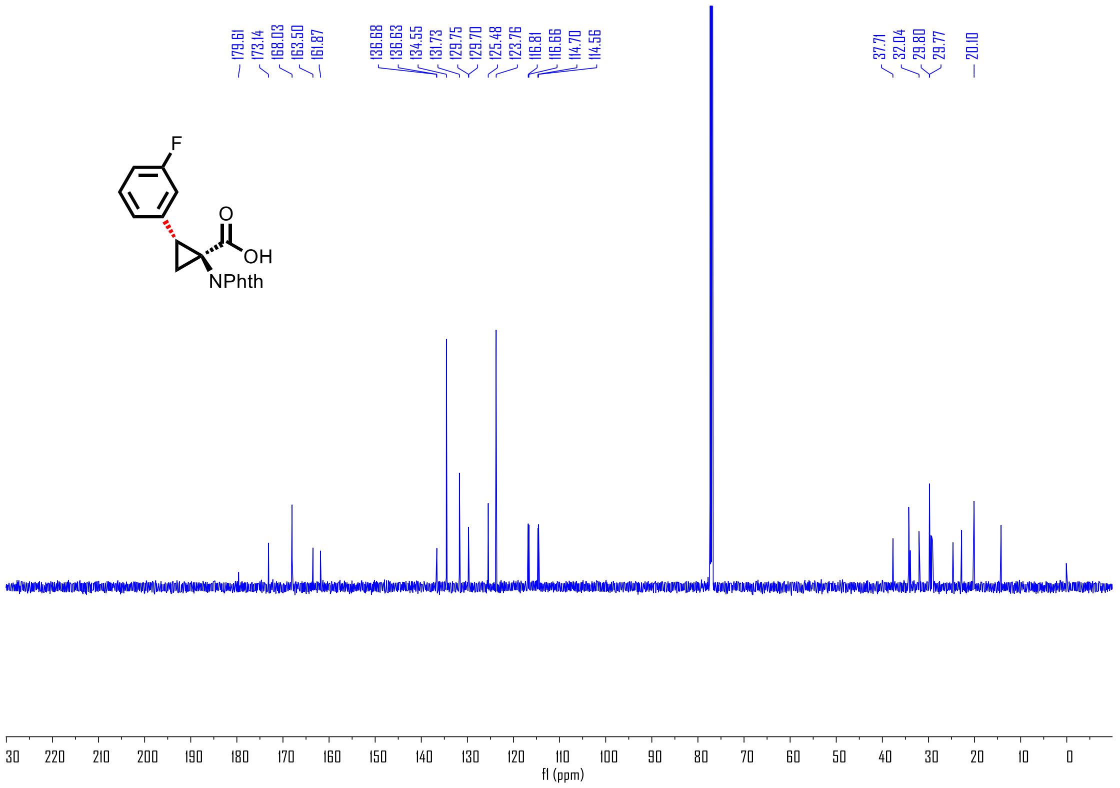
179.61
173.14
168.03
163.50
161.87
136.68
136.63
134.55
131.73
129.75
129.70
125.48
123.76
116.81
116.66
114.70
114.56
37.71
32.04
29.80
29.77
20.10
F
O
OH
NPhth
fl (ppm)

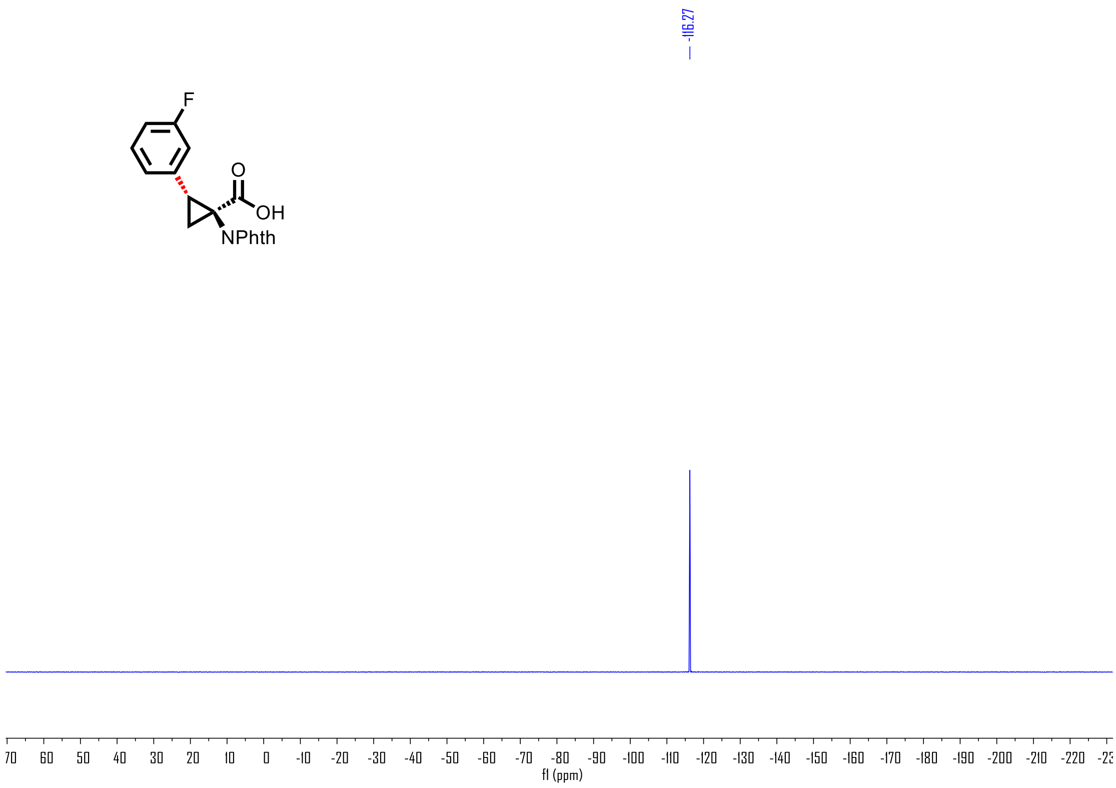
-116.27
F
O
OH
NPhth
70 60 50 40 30 20 10 0 -10 -20 -30 -40 -50 -60 -70 -80 -90 -100 -110 -120 -130 -140 -150 -160 -170 -180 -190 -200 -210 -220
f1 (ppm)

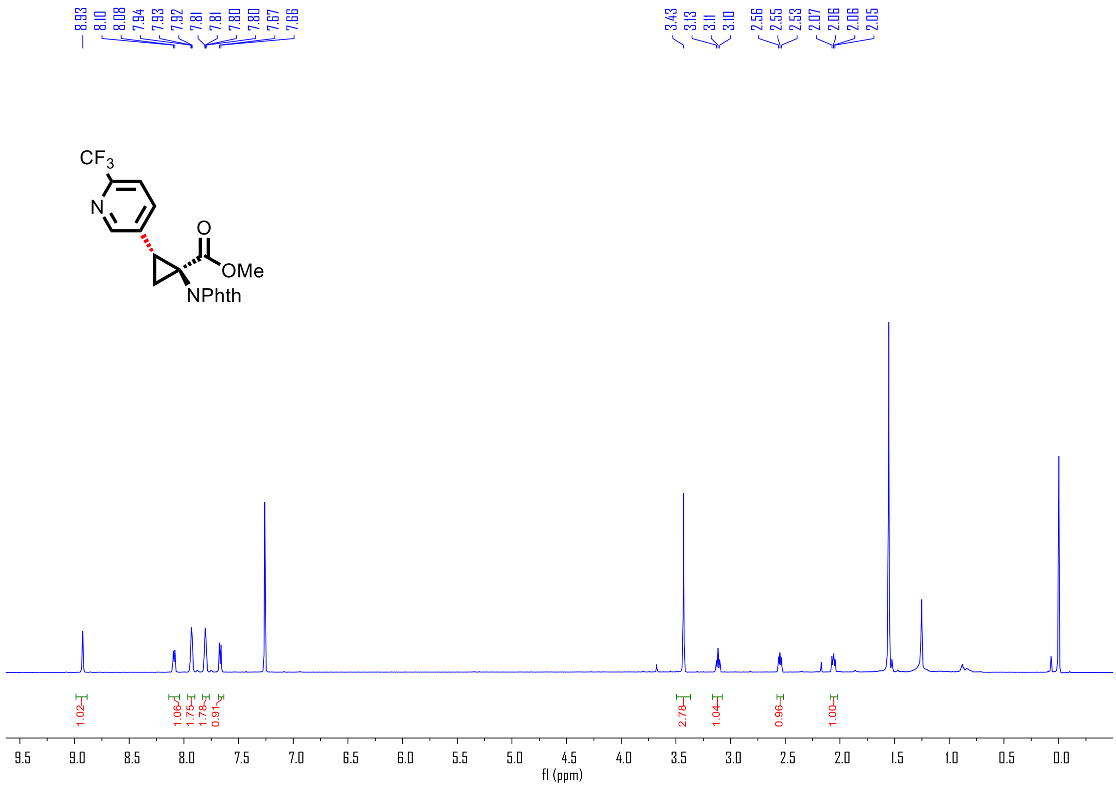
8.93 8.10 8.08 7.94 7.93 7.92 7.81 7.81 7.80 7.80 7.67 7.66
3.43 3.13 3.11 3.10 2.56 2.55 2.53 2.07 2.06 2.06 2.05
CF3
N
O
OMe
NPhth
1.02 1.06 1.75 1.78 0.91 2.78 1.04 0.96 1.00
9.5 9.0 8.5 8.0 7.5 7.0 6.5 6.0 5.5 5.0 4.5 4.0 3.5 3.0 2.5 2.0 1.5 1.0 0.5 0.0
f1 (ppm)

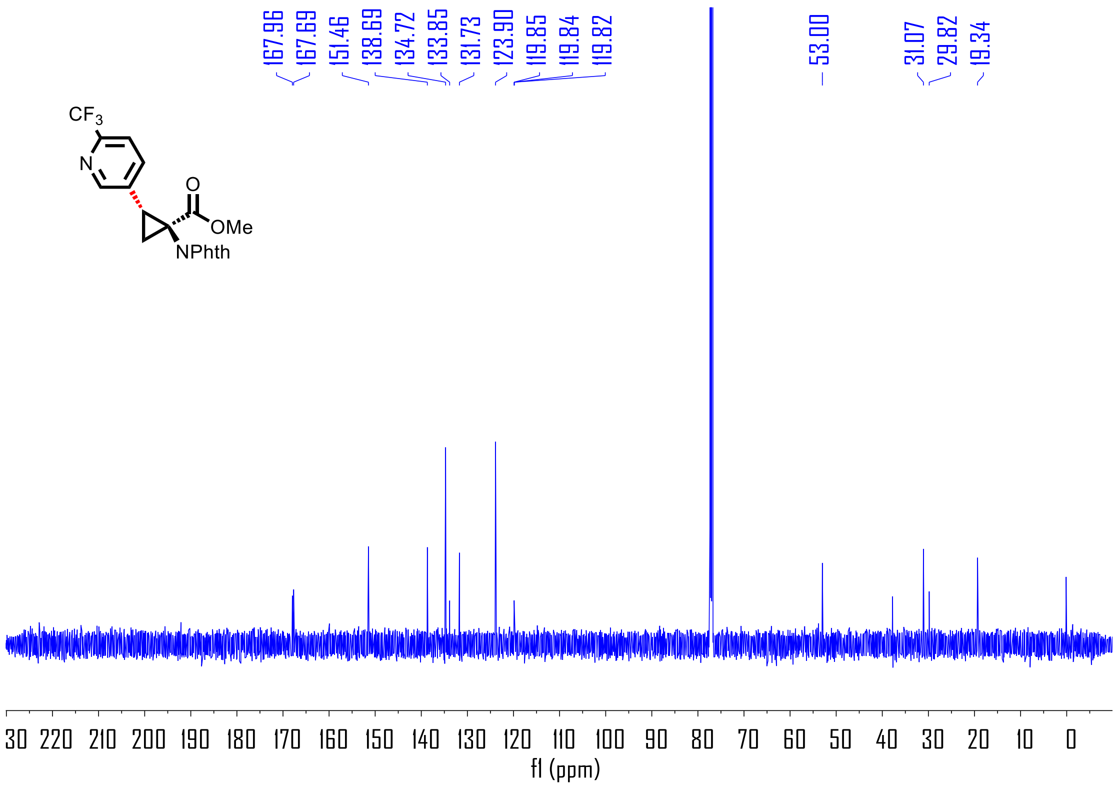
CF3
N
O
OMe
NPhth
167.96
167.69
151.46
138.69
134.72
133.85
131.73
123.90
119.85
119.84
119.82
53.00
31.07
29.82
19.34
f1 (ppm)

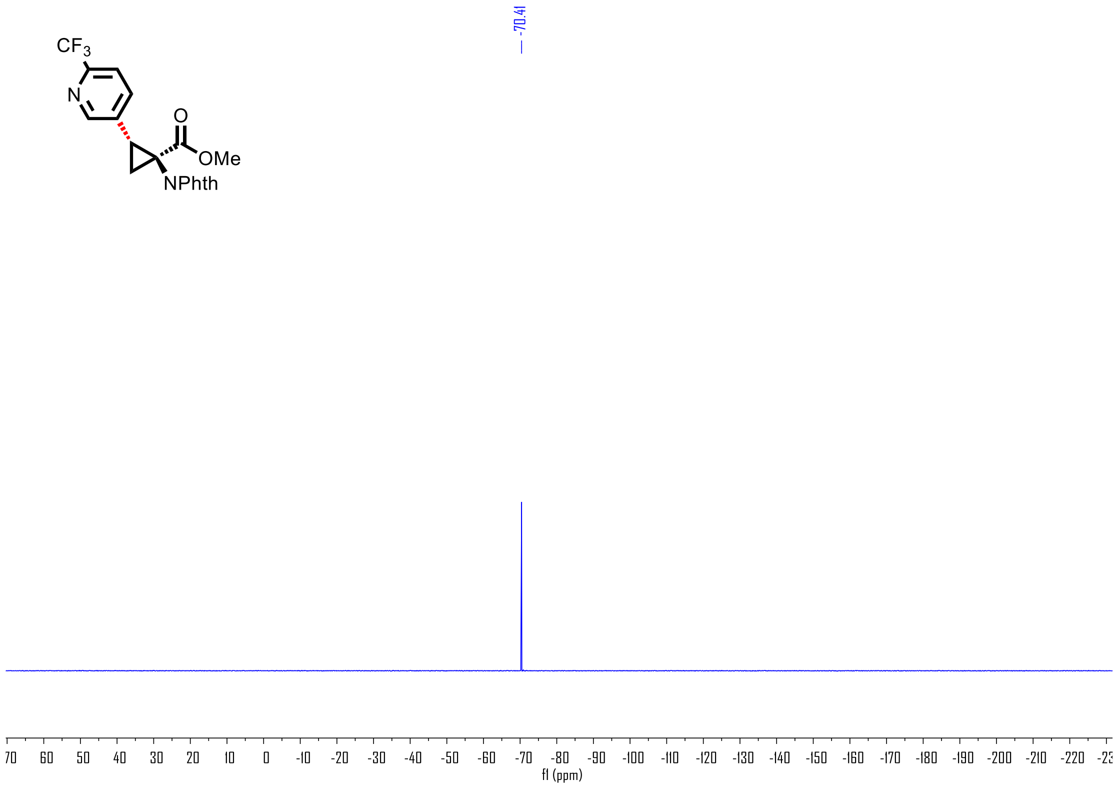
CF3
N
O
OMe
NPhth
-70.41
f1 (ppm)

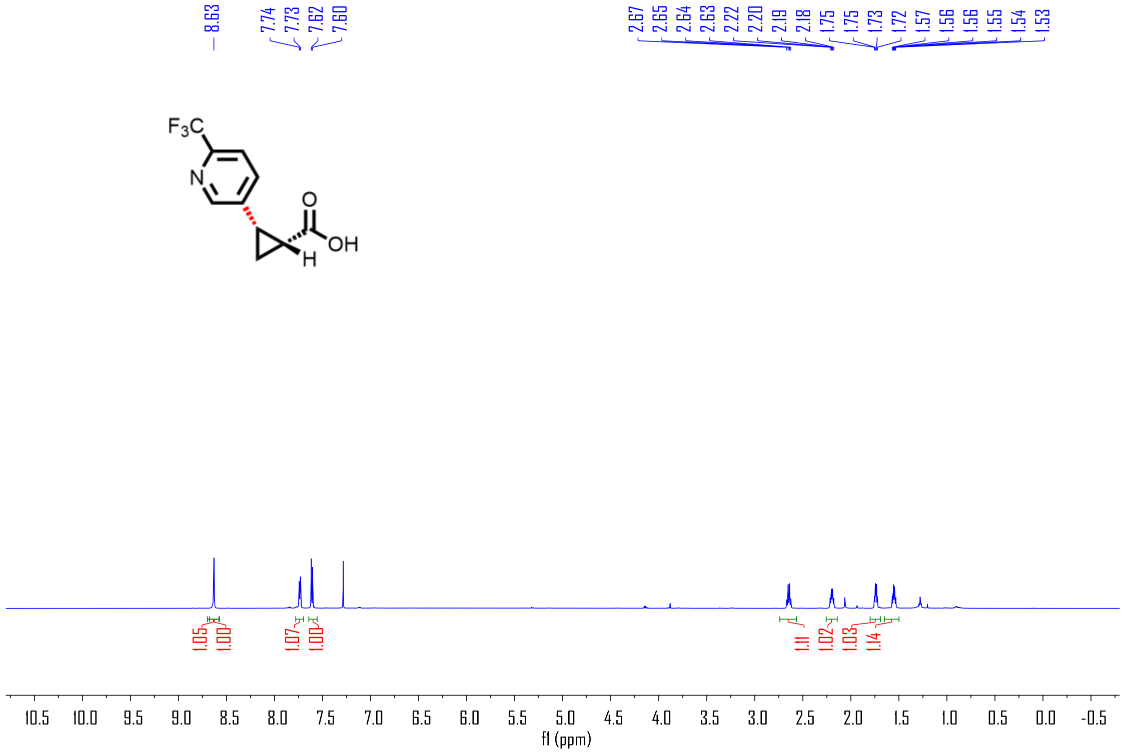
8.63
7.74 7.73 7.62 7.60
2.67 2.65 2.64 2.63 2.22 2.20 2.19 2.18 1.75 1.75 1.73 1.72 1.57 1.56 1.56 1.55 1.54 1.53
F3C
N
O
OH
H
1.05 1.00 1.07 1.00 1.11 1.02 1.03 1.14
f1 (ppm)

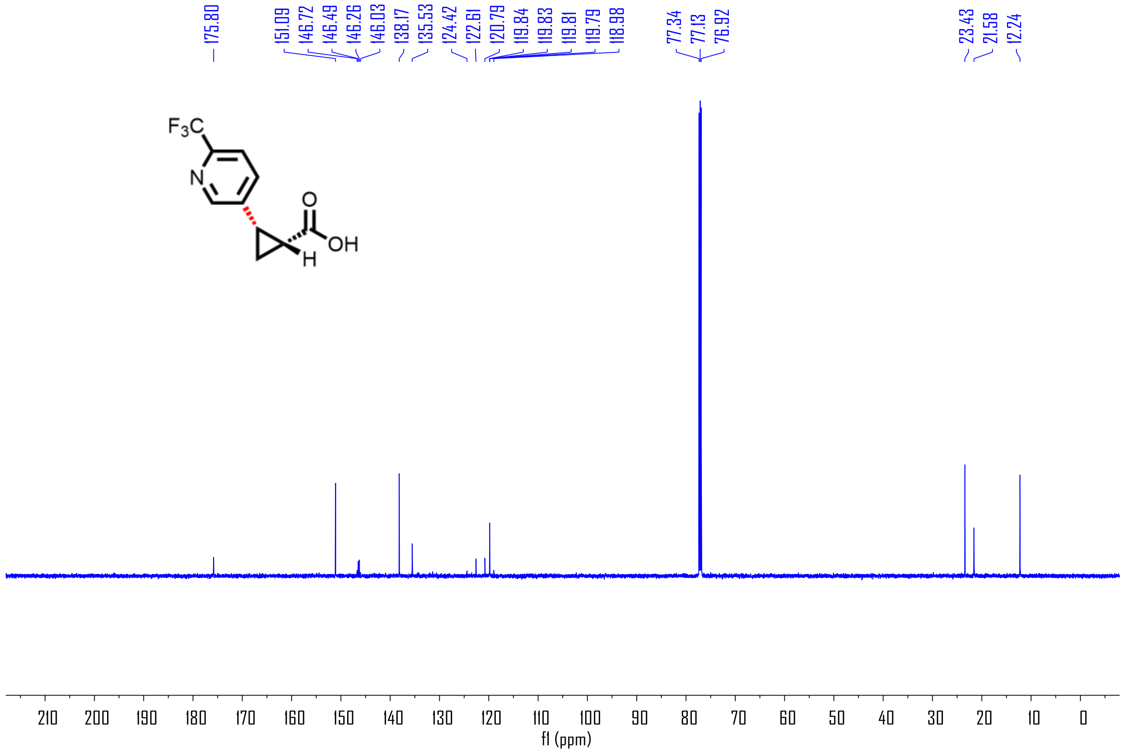
175.80
151.09 146.72 146.49 146.26 146.03 138.17 135.53 124.42 122.61 120.79 119.84 119.83 119.81 119.79 118.98
77.34 77.13 76.92
23.43 21.58 12.24
F3C
N
O
OH
H
f1 (ppm)

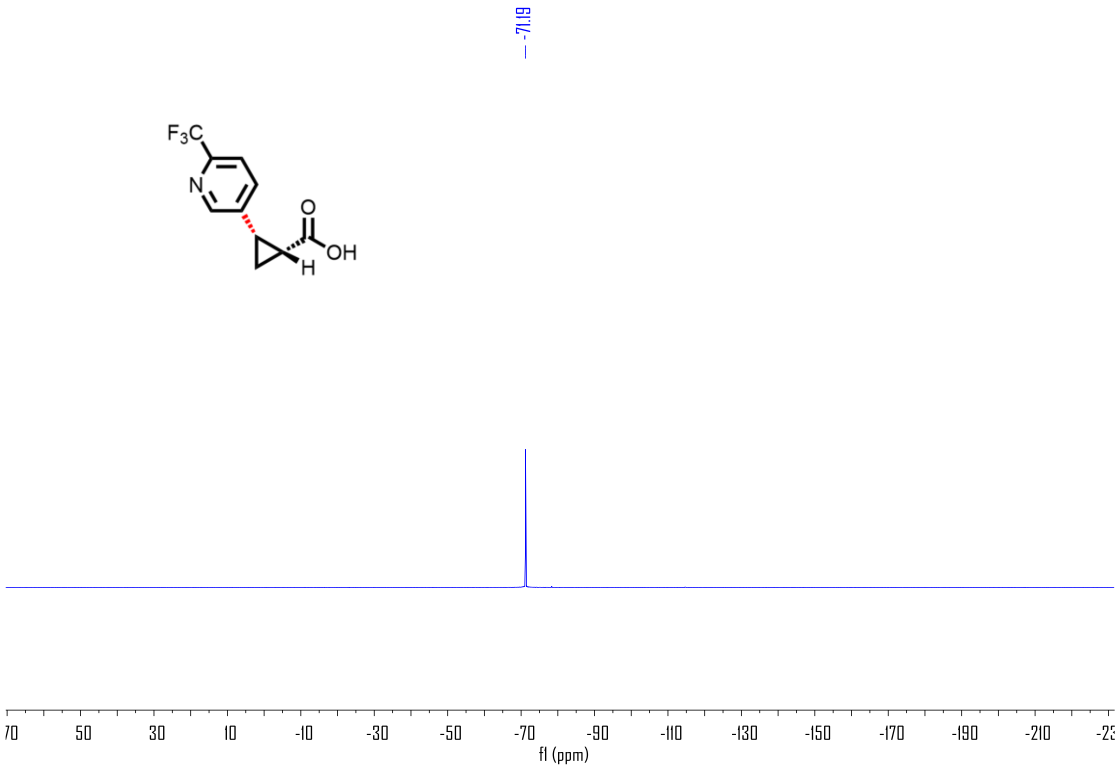

-71.19
F_3C
N
O
OH
H
70
50
30
10
-10
-30
-50
-70
-90
-110
-130
-150
-170
-190
-210
f1 (ppm)

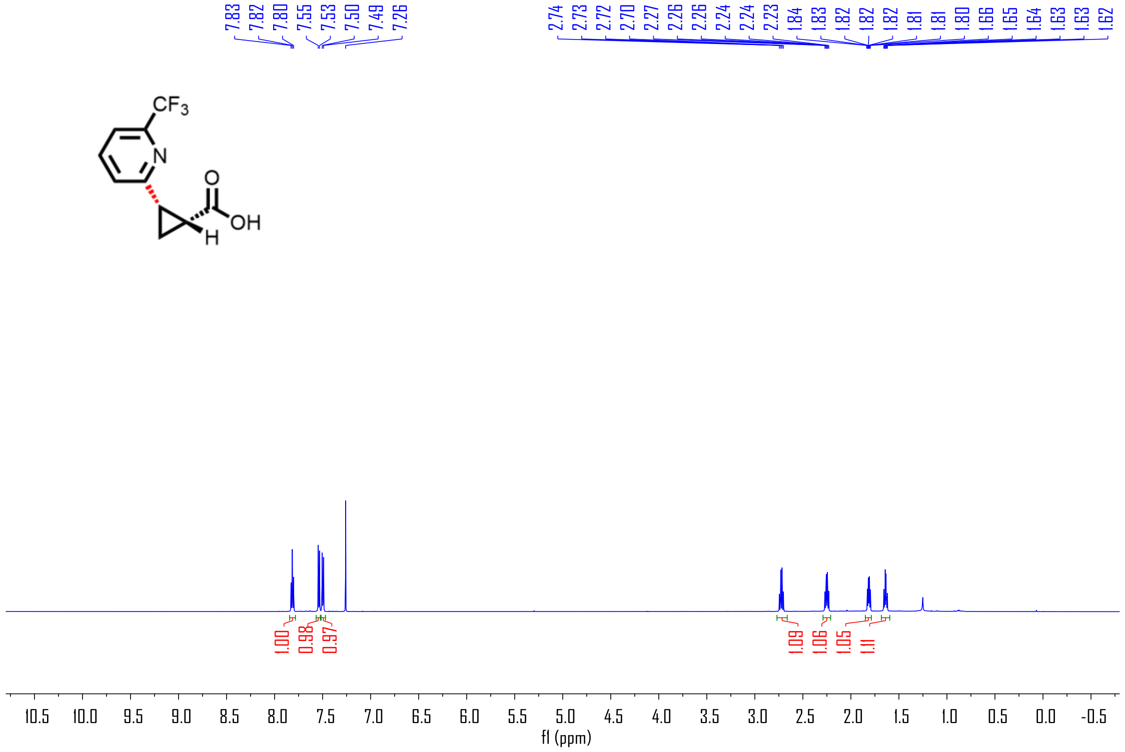

7.83 7.82 7.80 7.55 7.53 7.50 7.49 7.26
2.74 2.73 2.72 2.70 2.27 2.26 2.26 2.24 2.24 2.23 1.84 1.83 1.82 1.82 1.82 1.81 1.81 1.80 1.66 1.65 1.64 1.63 1.63 1.62
CF_3
N
O
OH
H
1.00 0.98 0.97
1.09 1.06 1.05 1.11
10.5 10.0 9.5 9.0 8.5 8.0 7.5 7.0 6.5 6.0 5.5 5.0 4.5 4.0 3.5 3.0 2.5 2.0 1.5 1.0 0.5 0.0 -0.5
f1 (ppm)

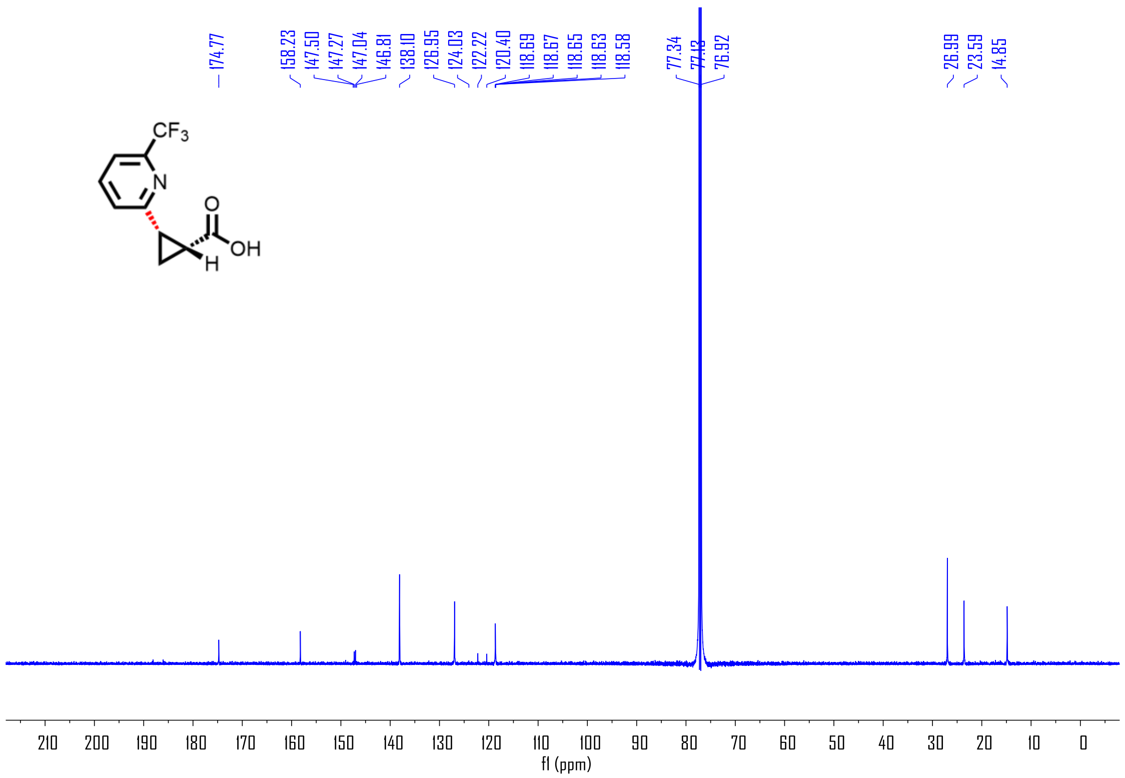
174.77
158.23
147.50
147.27
147.04
146.81
138.10
126.95
124.03
122.22
120.40
118.69
118.67
118.65
118.63
118.58
77.34
77.13
76.92
26.99
23.59
14.85
CF3
N
O
OH
H
f1 (ppm)

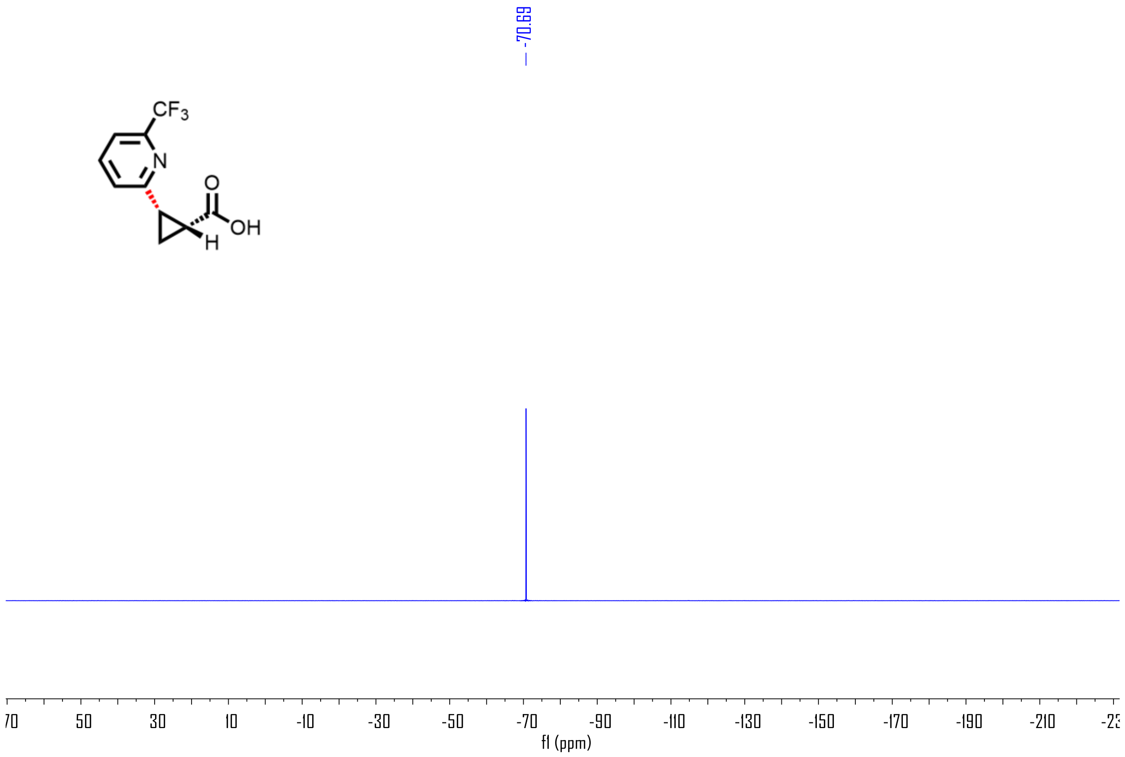
-70.69
CF3
N
O
OH
H
f1 (ppm)

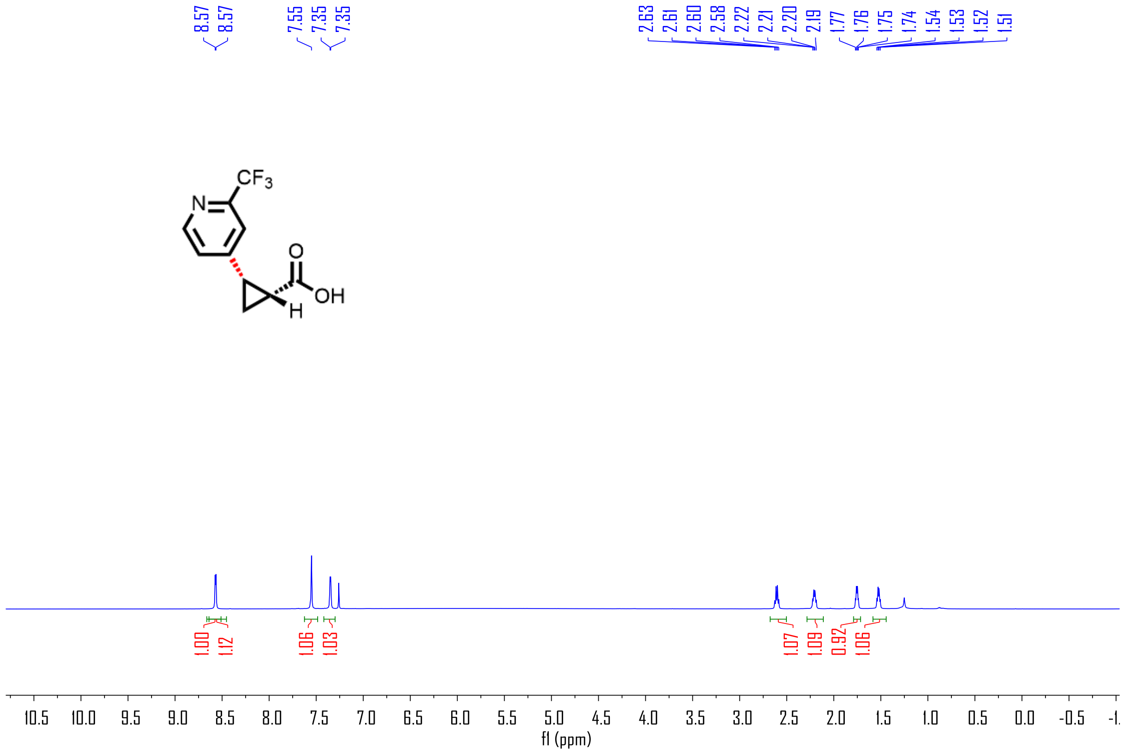

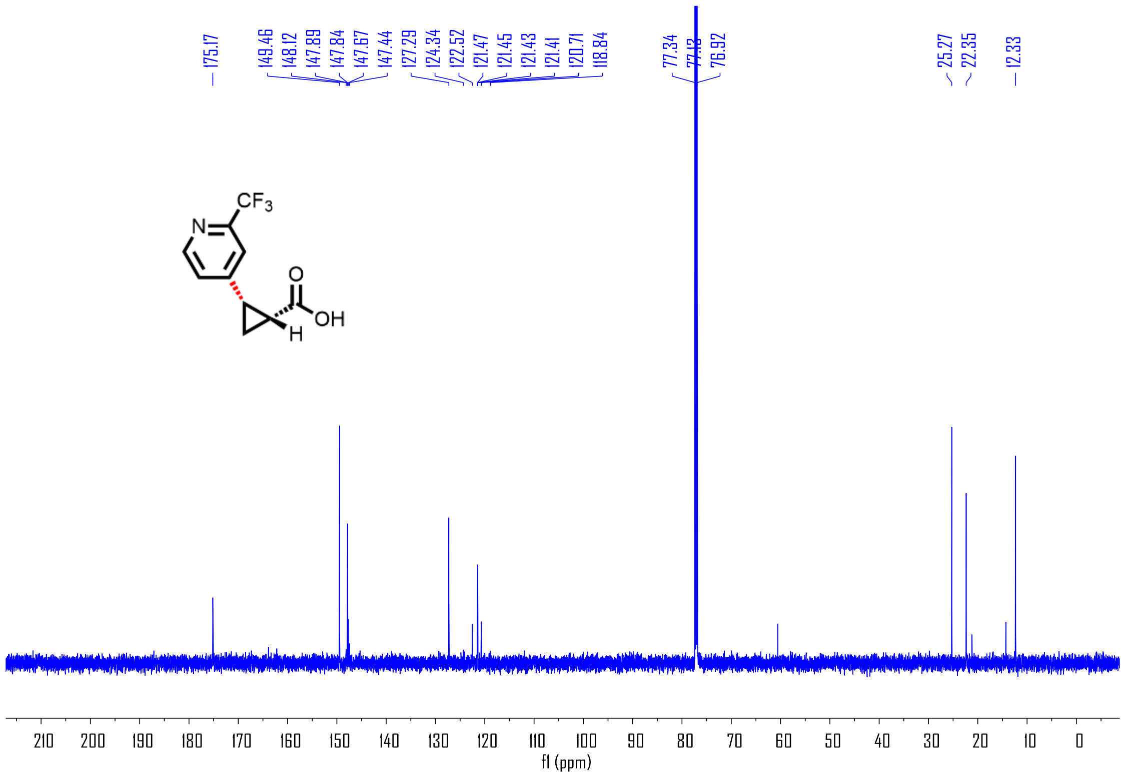

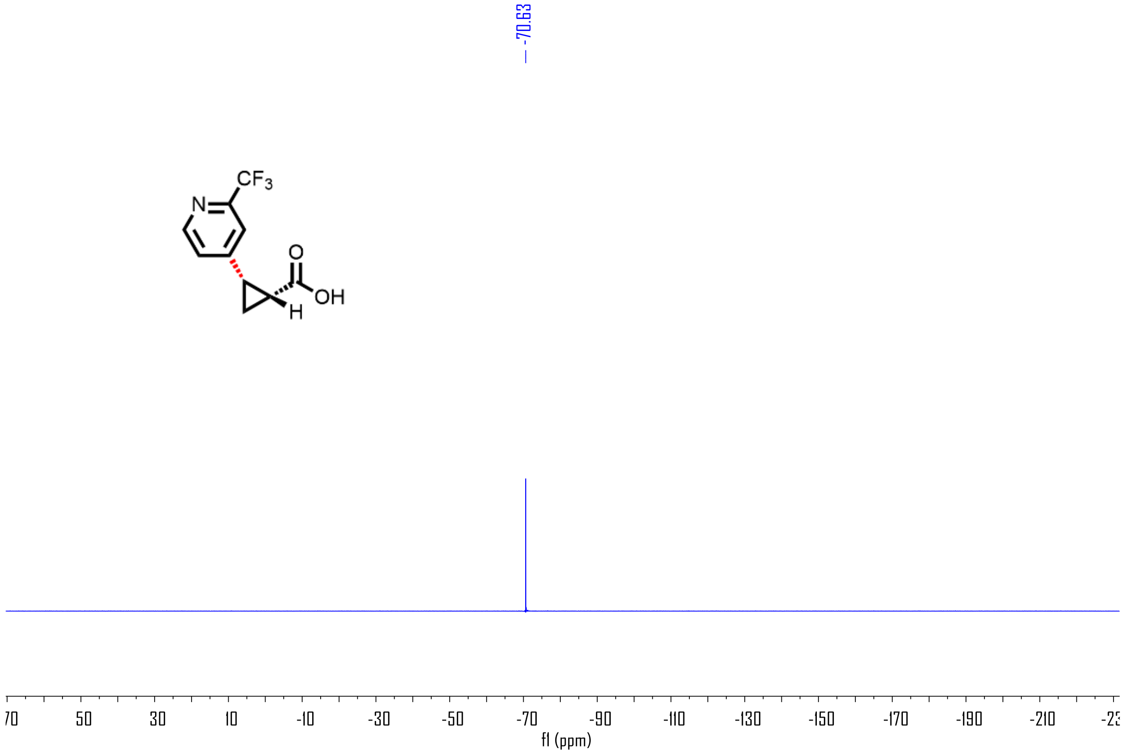
-70.63
CF3
N
O
OH
H
70
50
30
10
-10
-30
-50
-70
-90
-110
-130
-150
-170
-190
-210
f1 (ppm)

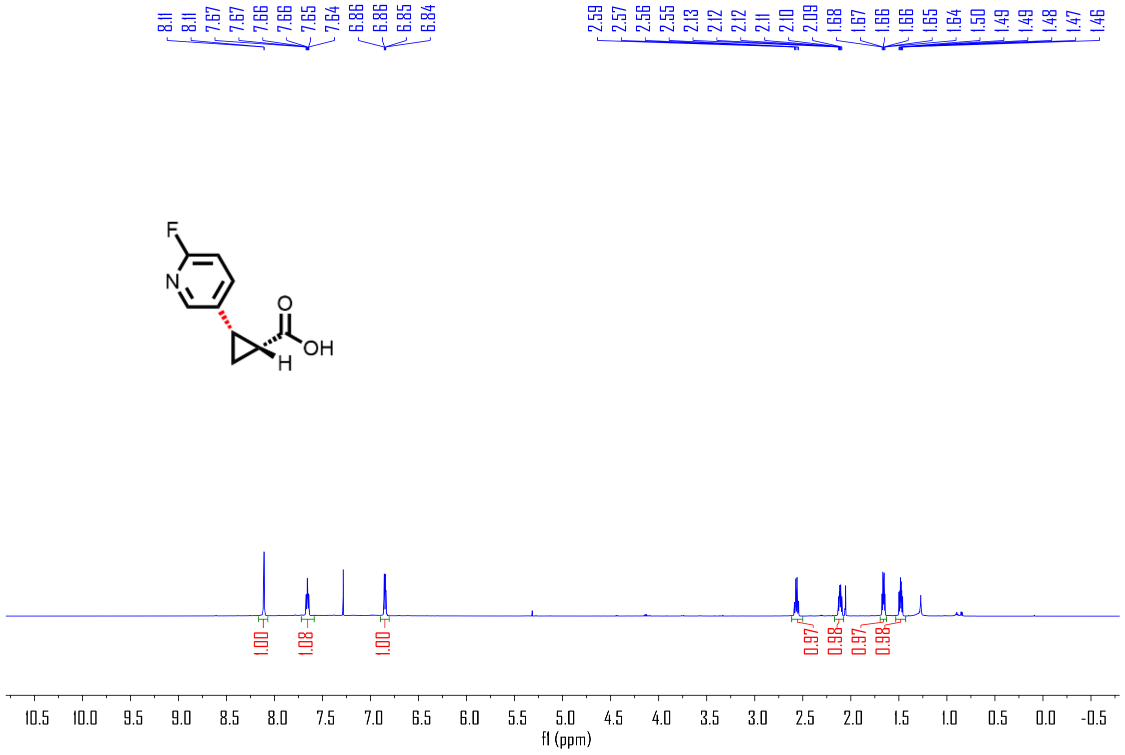
8.11
8.11
7.67
7.67
7.66
7.66
7.65
7.64
6.86
6.86
6.85
6.84
2.59
2.57
2.56
2.55
2.13
2.12
2.12
2.11
2.10
2.09
1.68
1.67
1.66
1.66
1.65
1.64
1.50
1.49
1.49
1.48
1.47
1.46
F
N
O
OH
H
1.00
1.08
1.00
0.97
0.98
0.97
0.98
10.5
10.0
9.5
9.0
8.5
8.0
7.5
7.0
6.5
6.0
5.5
5.0
4.5
4.0
3.5
3.0
2.5
2.0
1.5
1.0
0.5
0.0
-0.5
f1 (ppm)

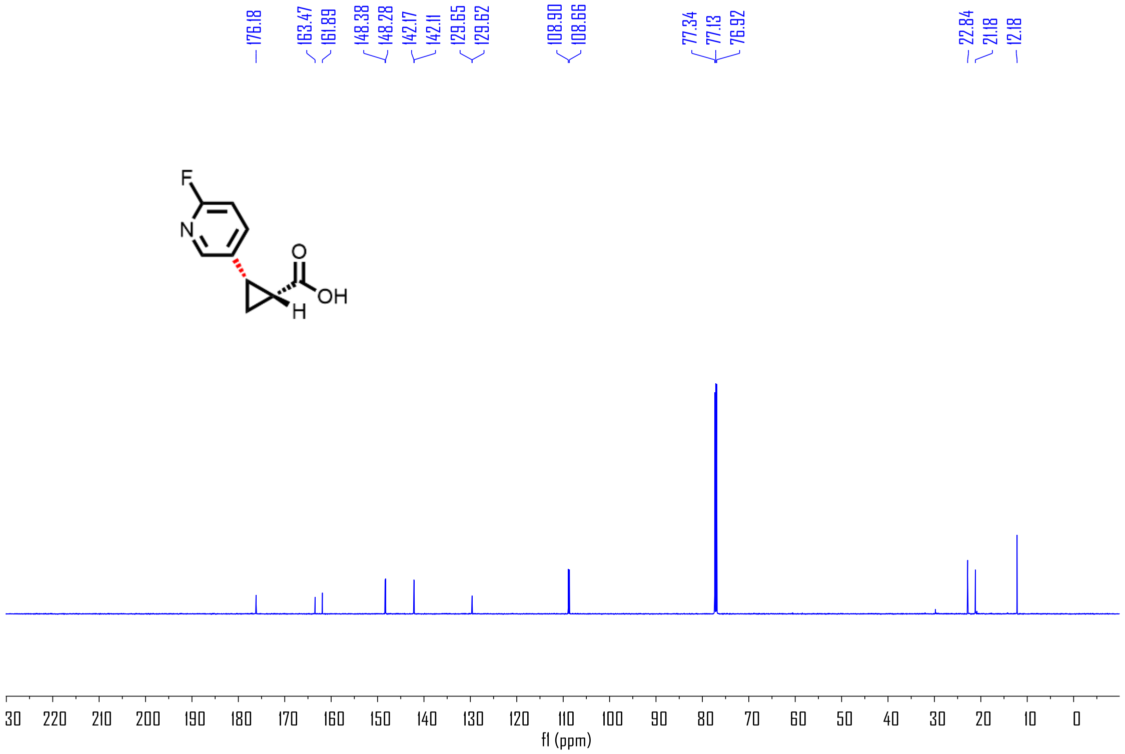
176.18
163.47
161.89
148.38
148.28
142.17
142.11
129.65
129.62
108.90
108.66
77.34
77.13
76.92
22.84
21.18
12.18
F
N
O
OH
H
fl (ppm)

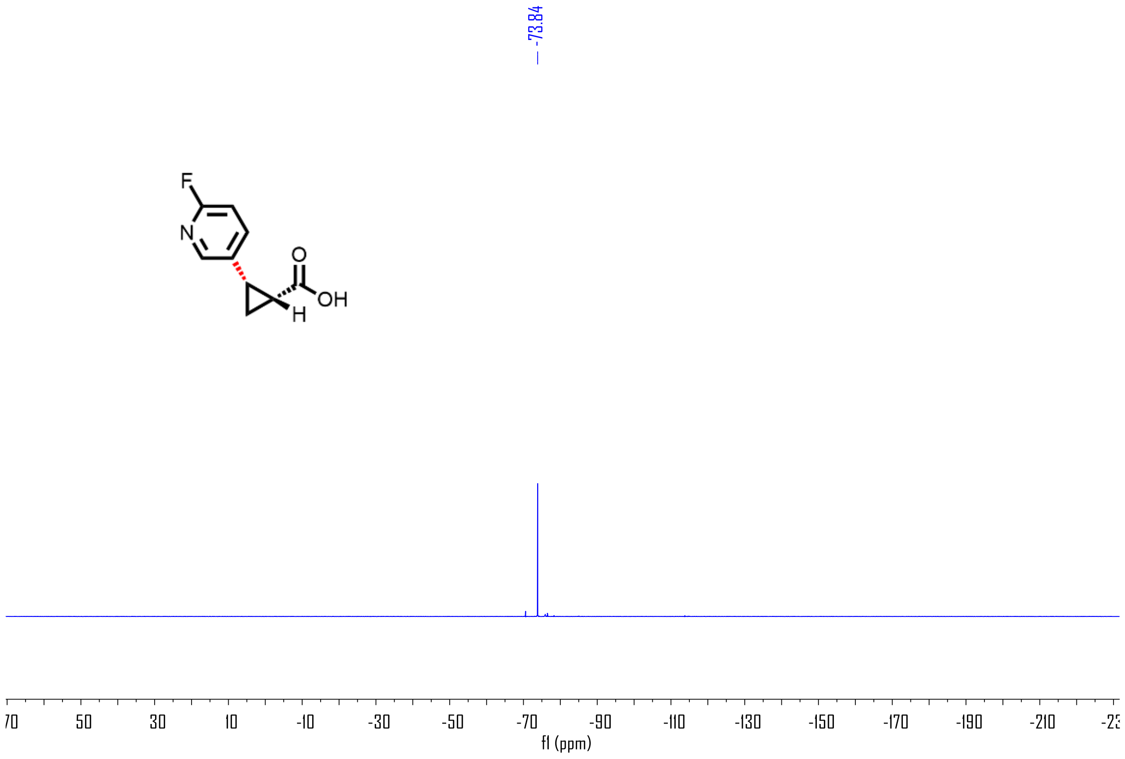
-73.84
F
N
O
OH
H
fl (ppm)

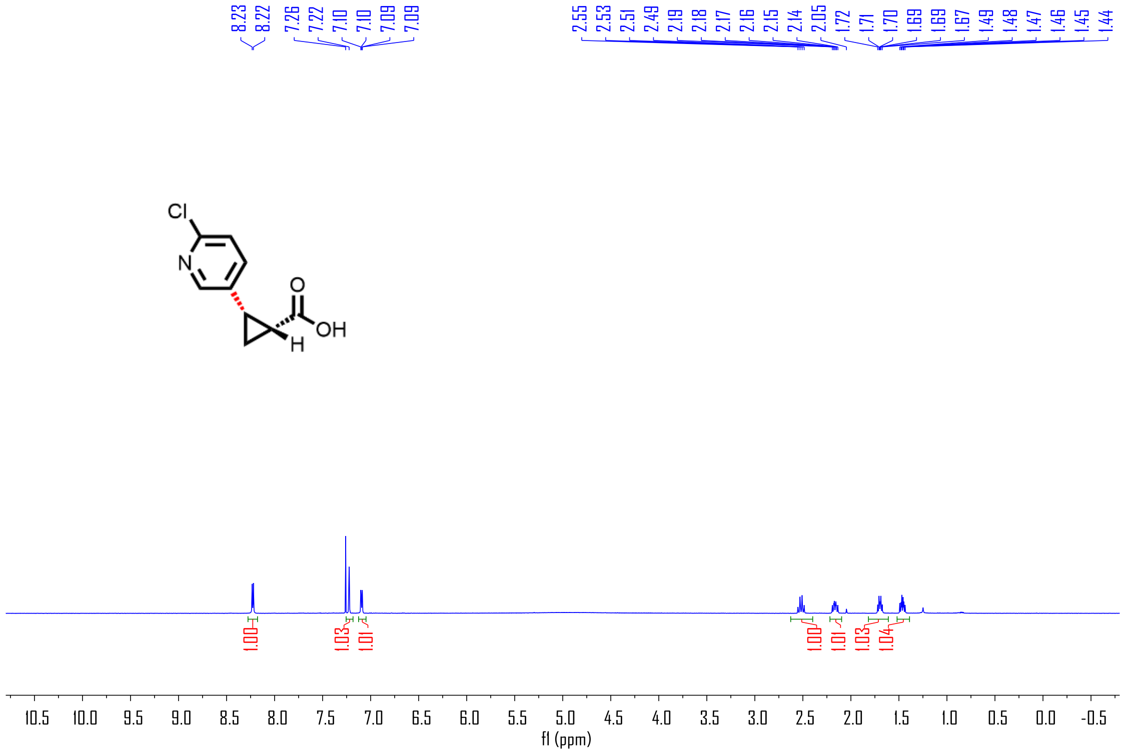
8.23
8.22
7.26
7.22
7.10
7.10
7.09
7.09
2.55
2.53
2.51
2.49
2.19
2.18
2.17
2.16
2.15
2.14
2.05
1.72
1.71
1.70
1.69
1.69
1.67
1.49
1.48
1.47
1.46
1.45
1.44
Cl
N
O
OH
H
1.00
1.03
1.01
1.00
1.01
1.03
1.04
10.5 10.0 9.5 9.0 8.5 8.0 7.5 7.0 6.5 6.0 5.5 5.0 4.5 4.0 3.5 3.0 2.5 2.0 1.5 1.0 0.5 0.0 -0.5
f1 (ppm)

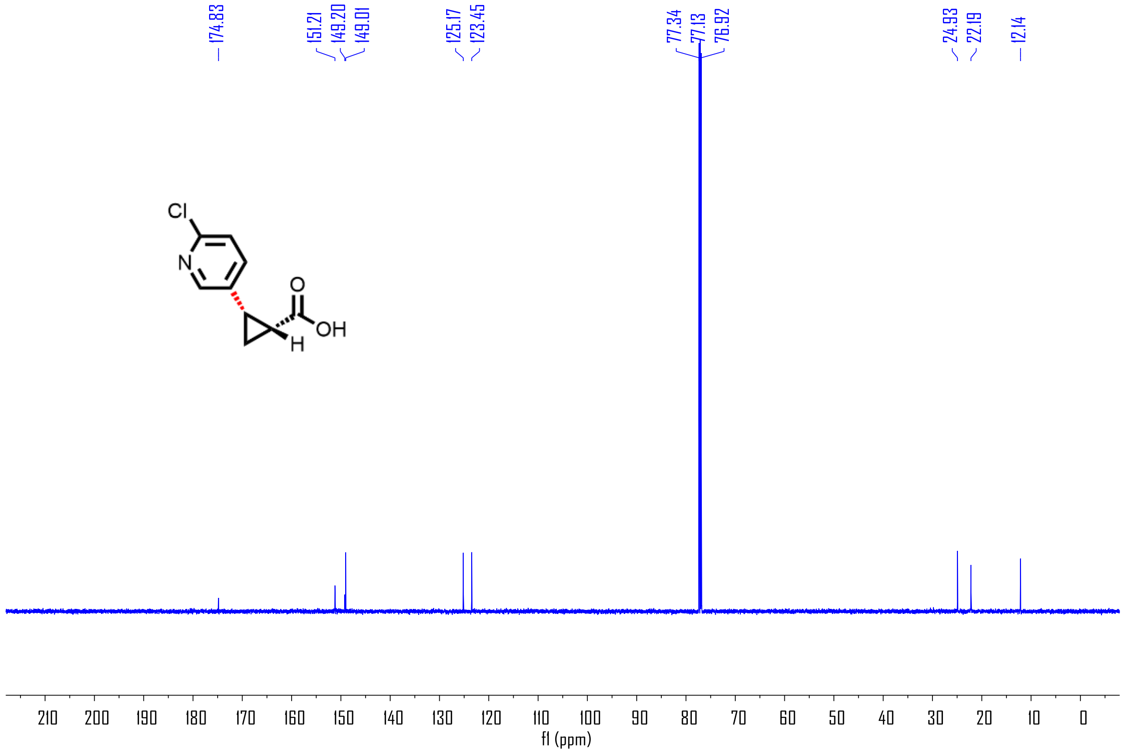
174.83
151.21
149.20
149.01
125.17
123.45
77.34
77.13
76.92
24.93
22.19
12.14
Cl
N
O
OH
H
210 200 190 180 170 160 150 140 130 120 110 100 90 80 70 60 50 40 30 20 10 0
f1 (ppm)

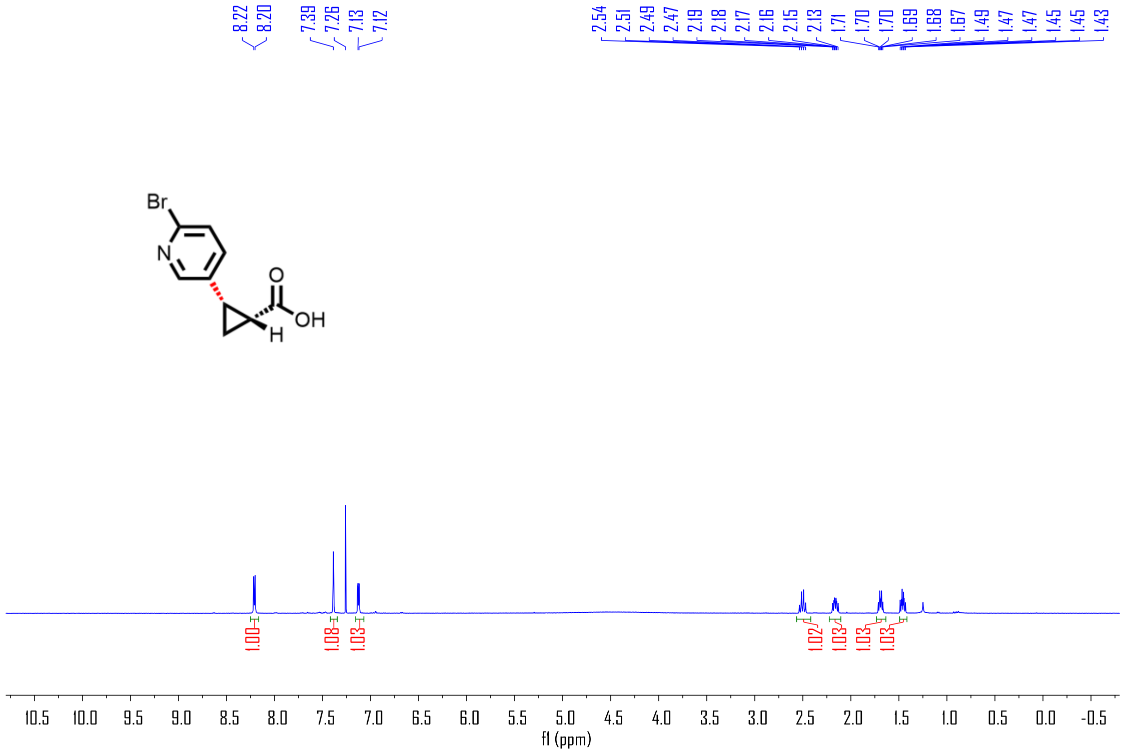
8.22
8.20
7.39
7.26
7.13
7.12
2.54
2.51
2.49
2.47
2.19
2.18
2.17
2.16
2.15
2.13
1.71
1.70
1.70
1.69
1.68
1.67
1.49
1.47
1.47
1.45
1.45
1.43
Br
N
O
OH
H
1.00
1.08
1.03
1.02
1.03
1.03
1.03
fl (ppm)

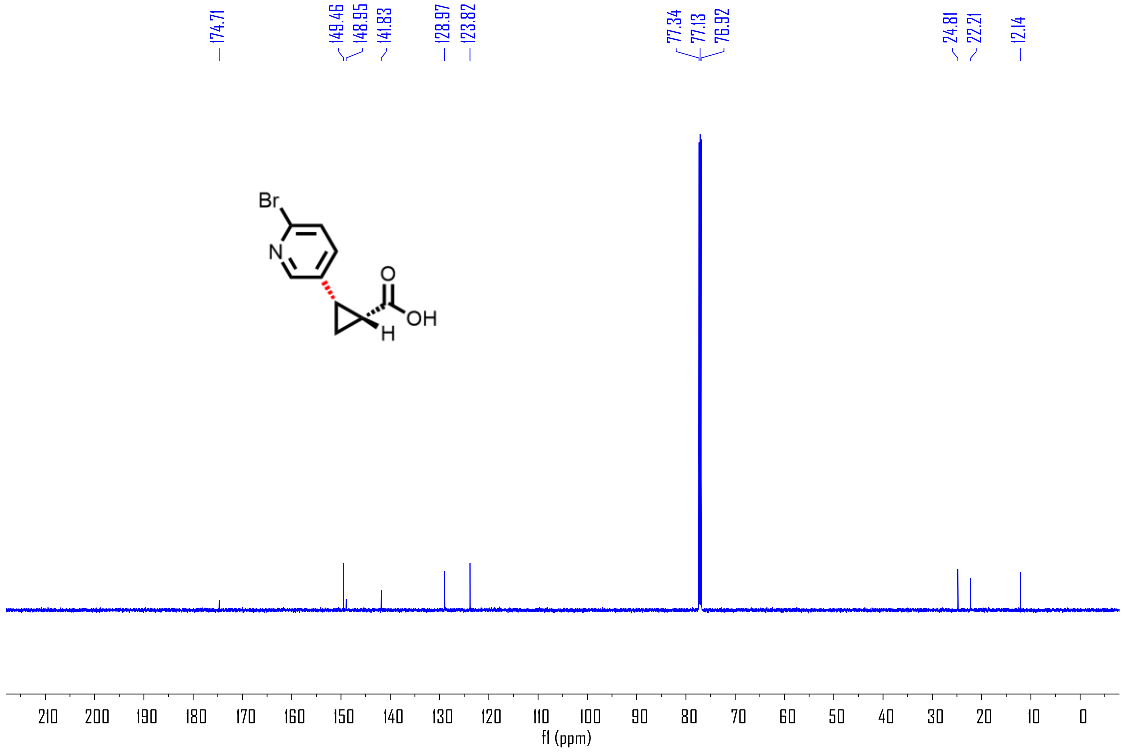
174.71
149.46
148.95
141.83
128.97
123.82
77.34
77.13
76.92
24.81
22.21
12.14
Br
N
O
OH
H
fl (ppm)

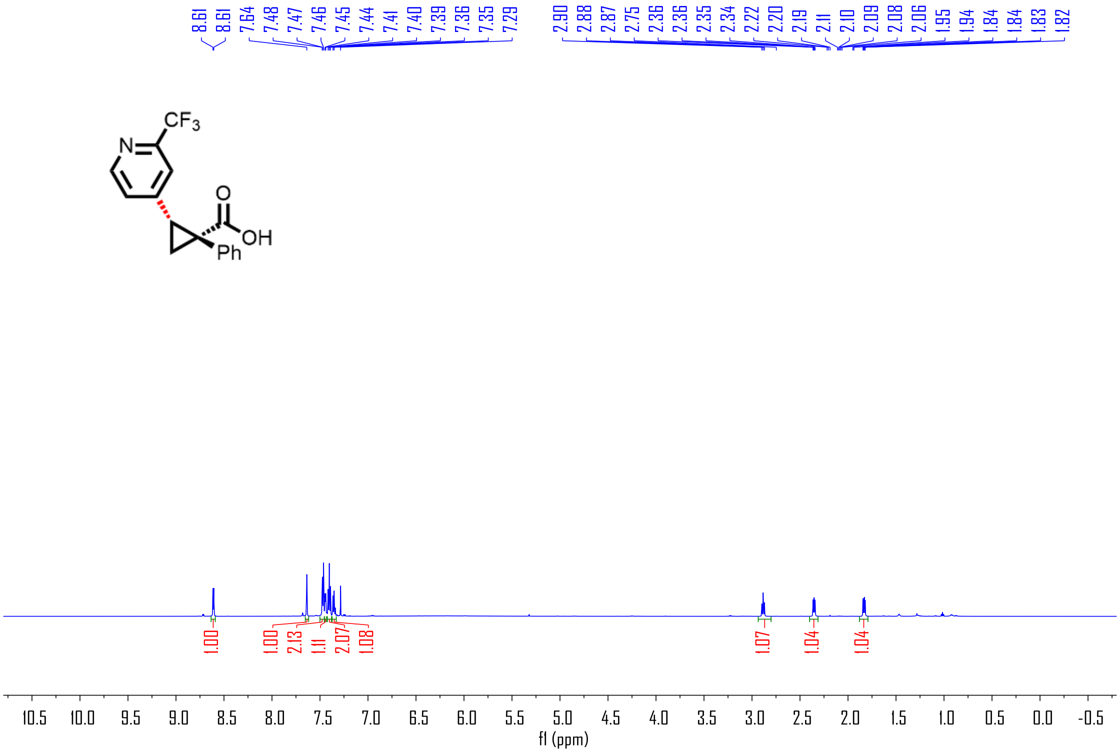
8.61
8.61
7.64
7.48
7.47
7.46
7.45
7.44
7.41
7.40
7.39
7.36
7.35
7.29
2.90
2.88
2.87
2.75
2.36
2.36
2.35
2.34
2.22
2.20
2.19
2.11
2.10
2.09
2.08
2.06
1.95
1.94
1.84
1.84
1.83
1.82
CF3
N
O
OH
Ph
1.00
1.00
2.13
1.11
2.07
1.08
1.07
1.04
1.04
10.5 10.0 9.5 9.0 8.5 8.0 7.5 7.0 6.5 6.0 5.5 5.0 4.5 4.0 3.5 3.0 2.5 2.0 1.5 1.0 0.5 0.0 -0.5
fl (ppm)

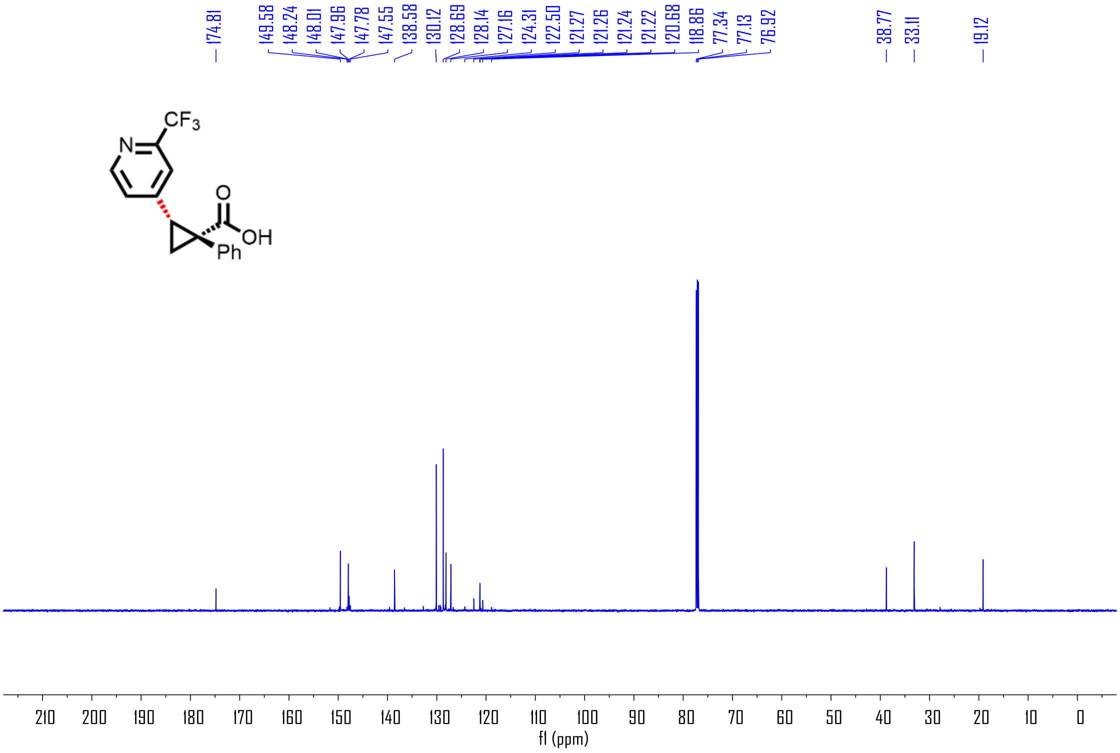
174.81
149.58
148.24
148.01
147.96
147.78
147.55
138.58
130.12
128.69
128.14
127.16
124.31
122.50
121.27
121.26
121.24
121.22
120.68
118.86
77.34
77.13
76.92
38.77
33.11
19.12
CF3
N
O
OH
Ph
210 200 190 180 170 160 150 140 130 120 110 100 90 80 70 60 50 40 30 20 10 0
fl (ppm)

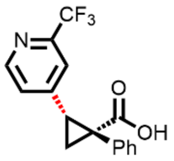

CF3
N
O
OH
Ph

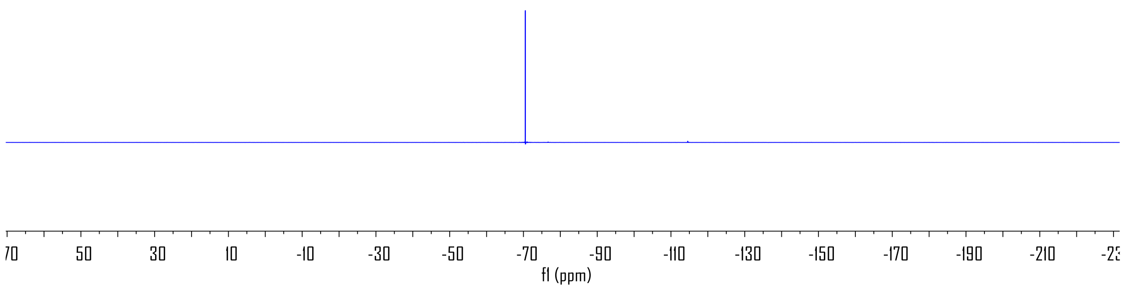

-70.52
50
30
10
-10
-30
-50
-70
-90
-110
-130
-150
-170
-190
-210
f1 (ppm)

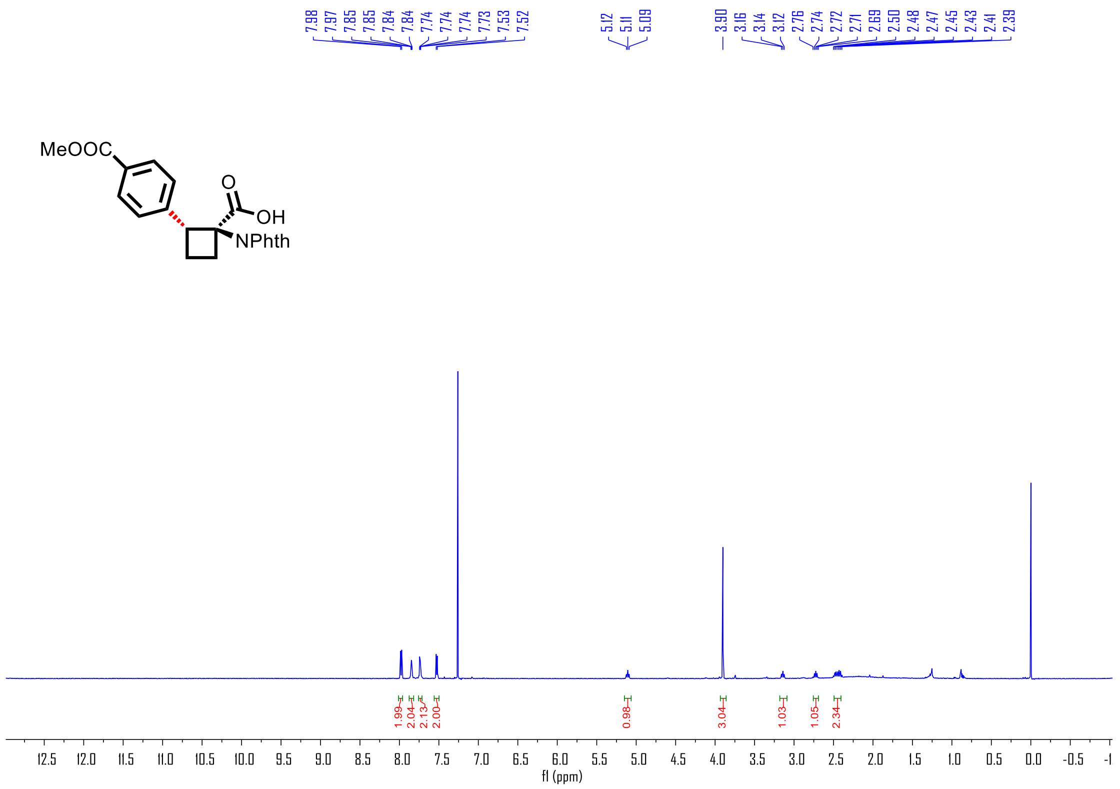

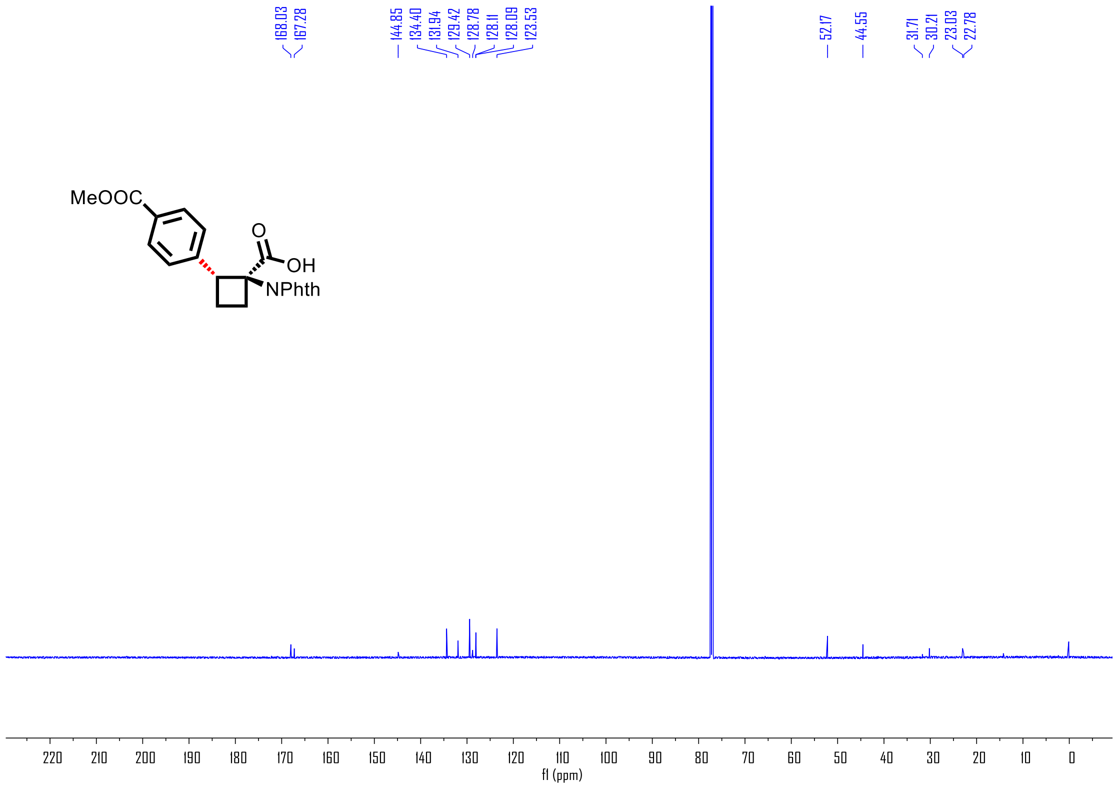

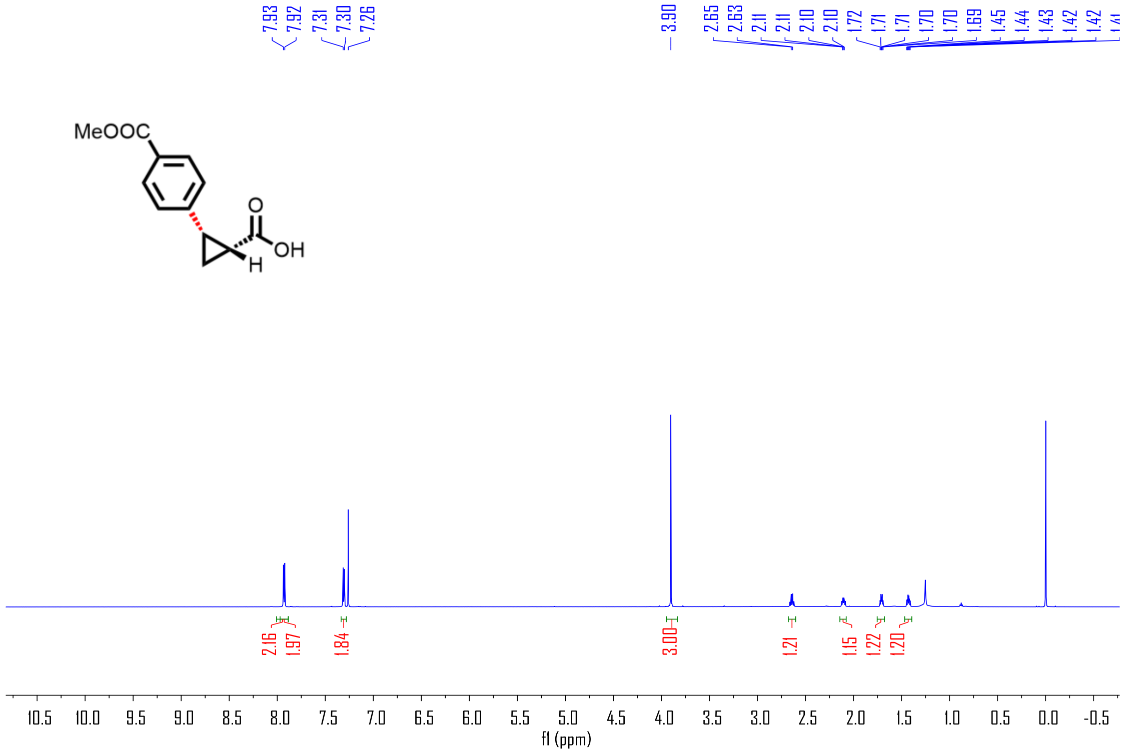
7.93
7.92
7.31
7.30
7.26
3.90
2.65
2.63
2.11
2.11
2.10
2.10
1.72
1.71
1.71
1.70
1.70
1.69
1.45
1.44
1.43
1.42
1.42
MeOOC
O
OH
H
2.16
1.97
1.84
3.00
1.21
1.15
1.22
1.20
10.5
10.0
9.5
9.0
8.5
8.0
7.5
7.0
6.5
6.0
5.5
5.0
4.5
4.0
3.5
3.0
2.5
2.0
1.5
1.0
0.5
0.0
-0.5
f1 (ppm)

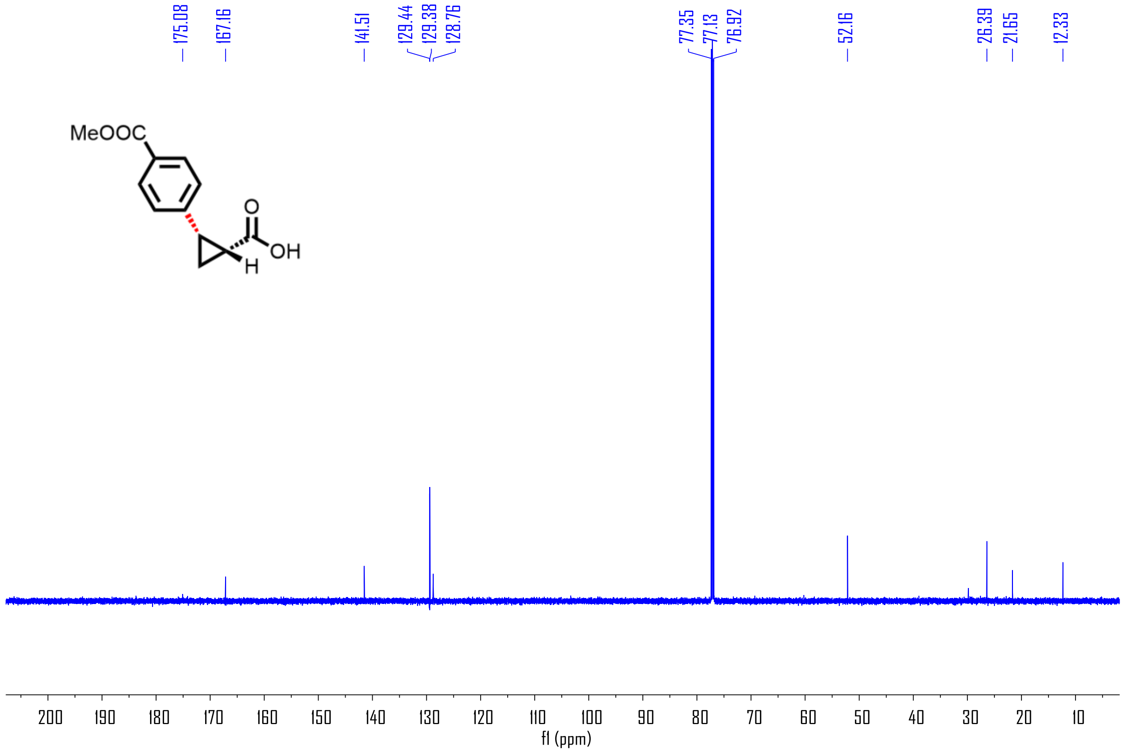
175.08
167.16
141.51
129.44
129.38
128.76
77.35
77.13
76.92
52.16
26.39
21.65
12.33
MeOOC
O
OH
H
200
190
180
170
160
150
140
130
120
110
100
90
80
70
60
50
40
30
20
10
f1 (ppm)

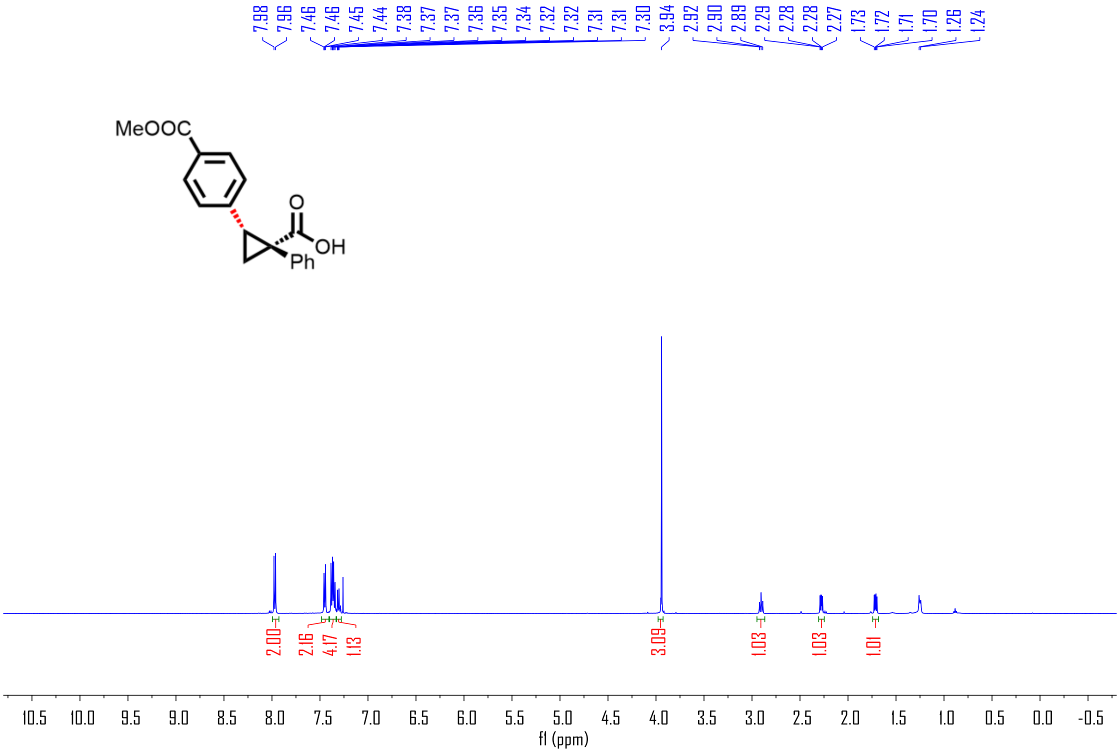
7.98 7.96 7.46 7.46 7.45 7.44 7.38 7.37 7.37 7.36 7.35 7.34 7.32 7.32 7.31 7.31 7.30 3.94 2.92 2.90 2.89 2.29 2.28 2.28 2.27 1.73 1.72 1.71 1.70 1.26 1.24
MeOOC
O
OH
Ph
2.00 2.16 4.17 1.13 3.09 1.03 1.03 1.01
10.5 10.0 9.5 9.0 8.5 8.0 7.5 7.0 6.5 6.0 5.5 5.0 4.5 4.0 3.5 3.0 2.5 2.0 1.5 1.0 0.5 0.0 -0.5
f1 (ppm)

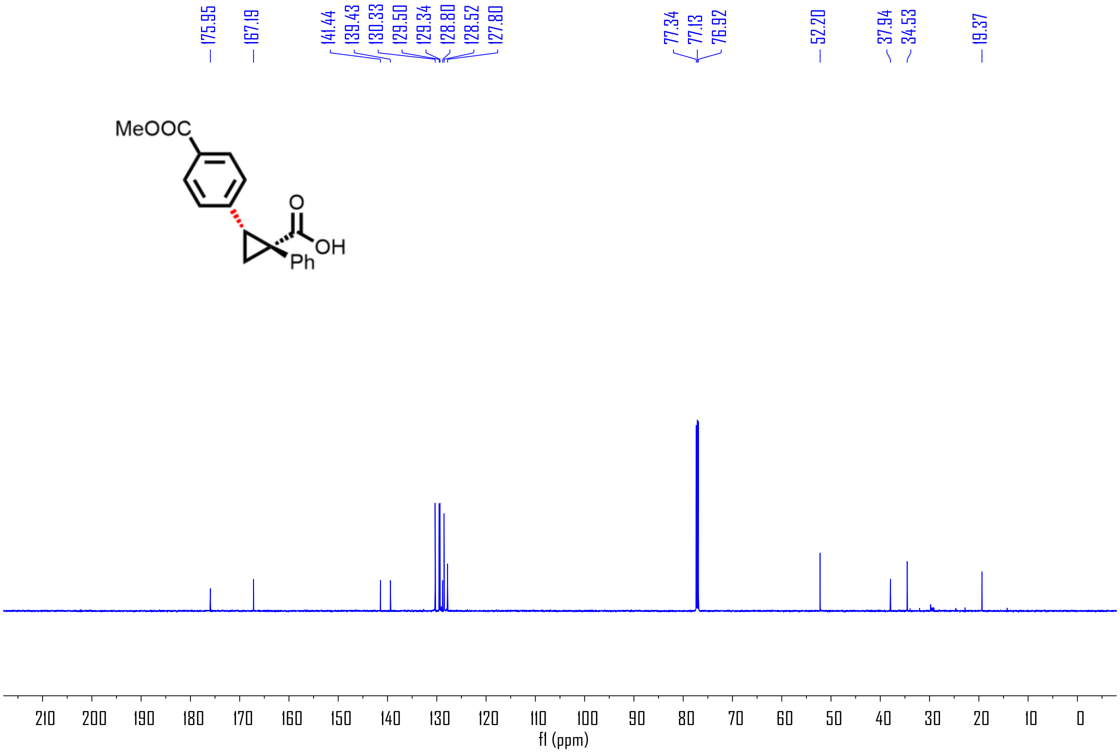
175.95 167.19 141.44 139.43 130.33 129.50 129.34 128.80 128.52 127.80 77.34 77.13 76.92 52.20 37.94 34.53 19.37
MeOOC
O
OH
Ph
210 200 190 180 170 160 150 140 130 120 110 100 90 80 70 60 50 40 30 20 10 0
f1 (ppm)

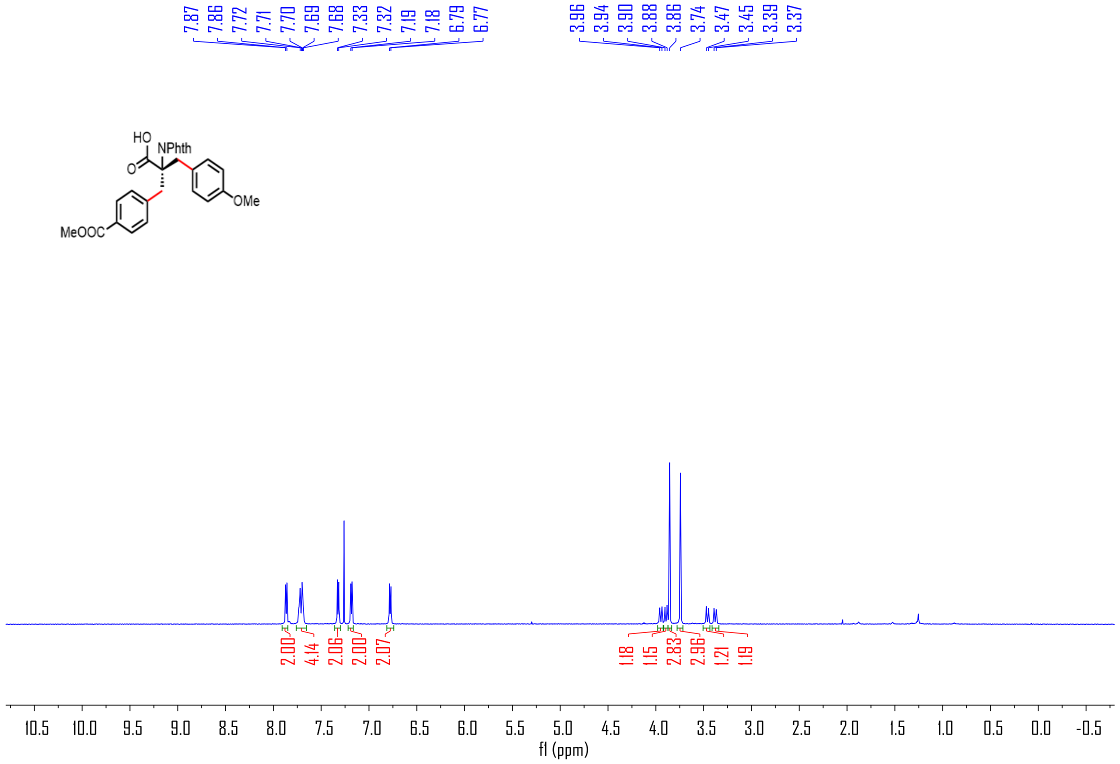
7.87
7.86
7.72
7.71
7.70
7.69
7.68
7.33
7.32
7.19
7.18
6.79
6.77
3.96
3.94
3.90
3.88
3.86
3.74
3.47
3.45
3.39
3.37
HO
NPhth
O
OMe
MeOOC
2.00
4.14
2.06
2.00
2.07
1.18
1.15
2.83
2.96
1.21
1.19
f1 (ppm)

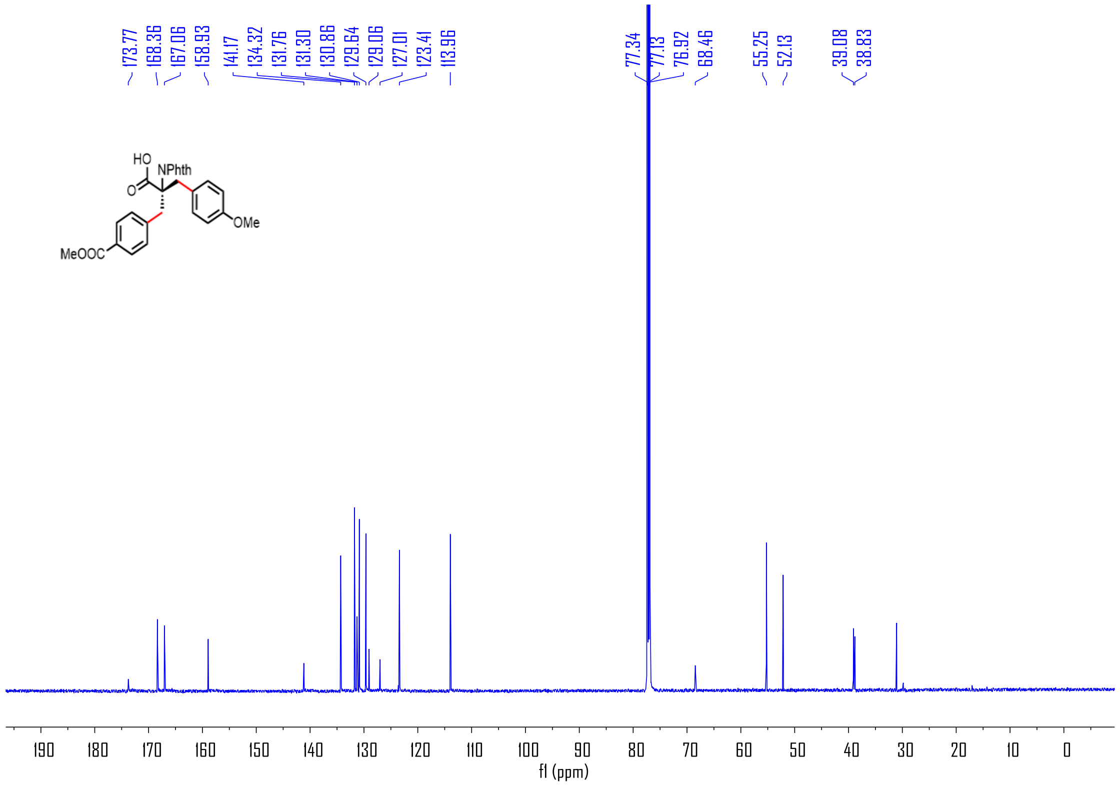
173.77
168.36
167.06
158.93
141.17
134.32
131.76
131.30
130.86
129.64
129.06
127.01
123.41
113.96
77.34
77.13
76.92
68.46
55.25
52.13
39.08
38.83
HO
NPhth
O
OMe
MeOOC
f1 (ppm)

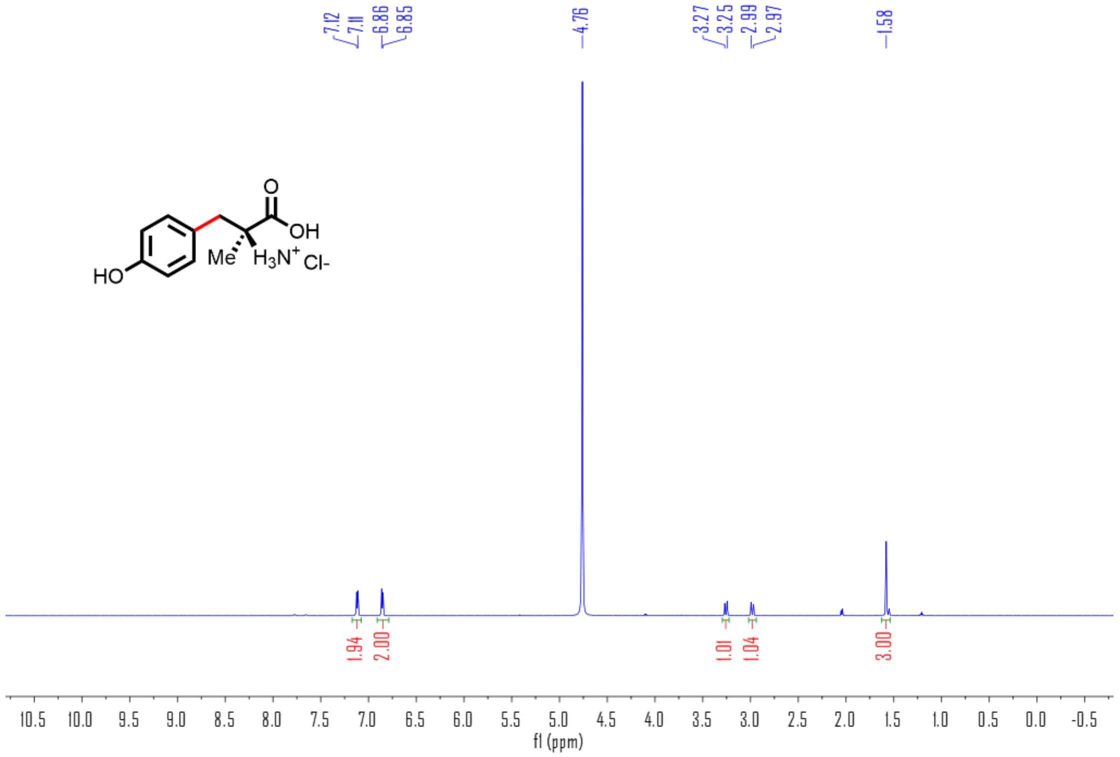
7.12
7.11
6.86
6.85
4.76
3.27
3.25
2.99
2.97
1.58
HO
Me
H3N+ Cl-
OH
O
1.94
2.00
1.01
1.04
3.00
f1 (ppm)

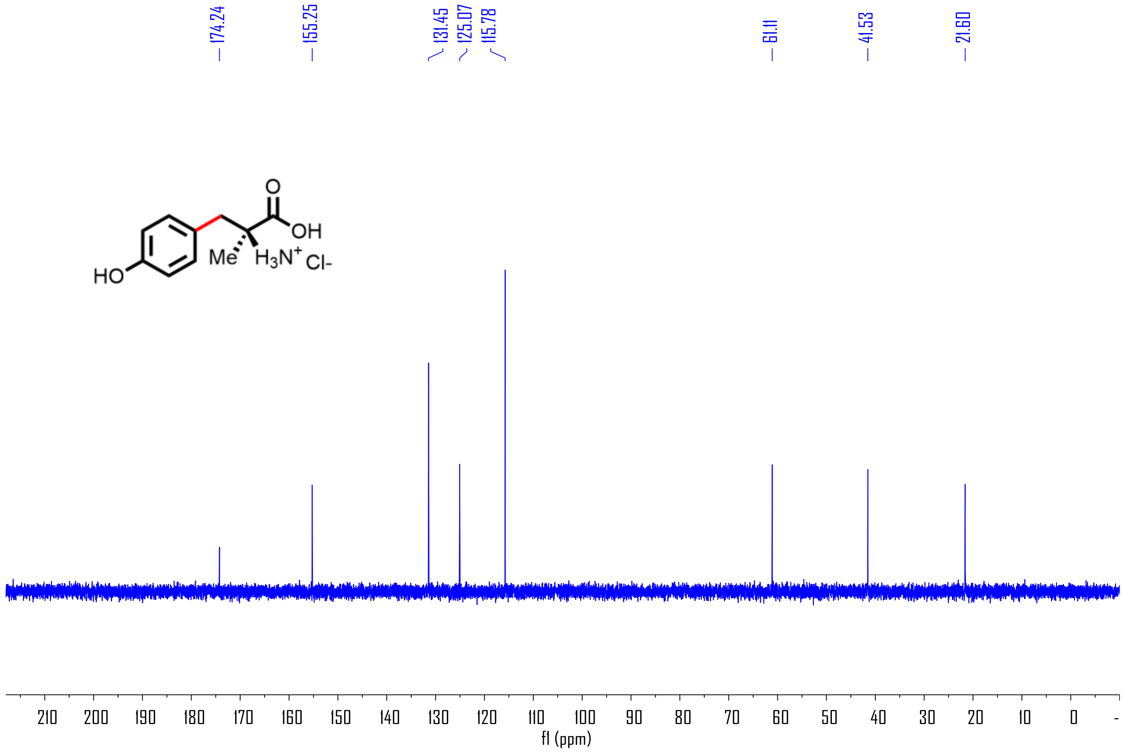
174.24
155.25
131.45
125.07
115.78
61.11
41.53
21.60
HO
Me
H3N+ Cl-
OH
O
f1 (ppm)

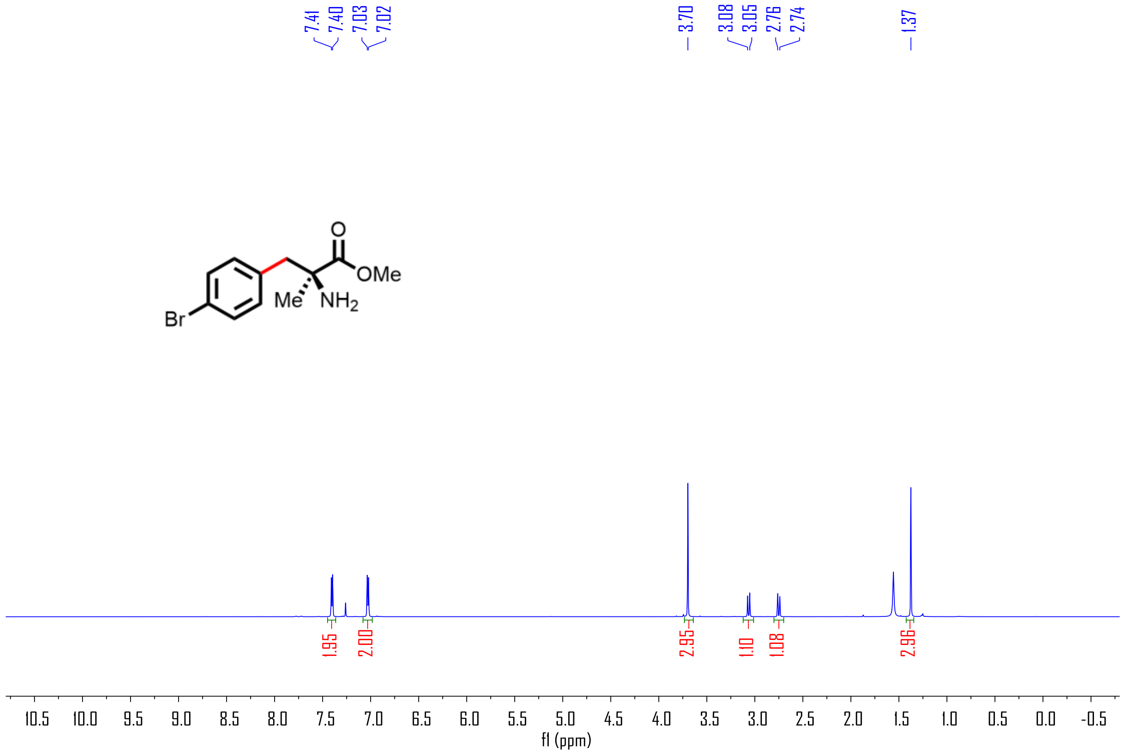
7.41
7.40
7.03
7.02
3.70
3.08
3.05
2.76
2.74
1.37
O
OMe
Br
Me
NH2
1.95
2.00
2.95
1.10
1.08
2.96
10.5 10.0 9.5 9.0 8.5 8.0 7.5 7.0 6.5 6.0 5.5 5.0 4.5 4.0 3.5 3.0 2.5 2.0 1.5 1.0 0.5 0.0 -0.5
f1 (ppm)

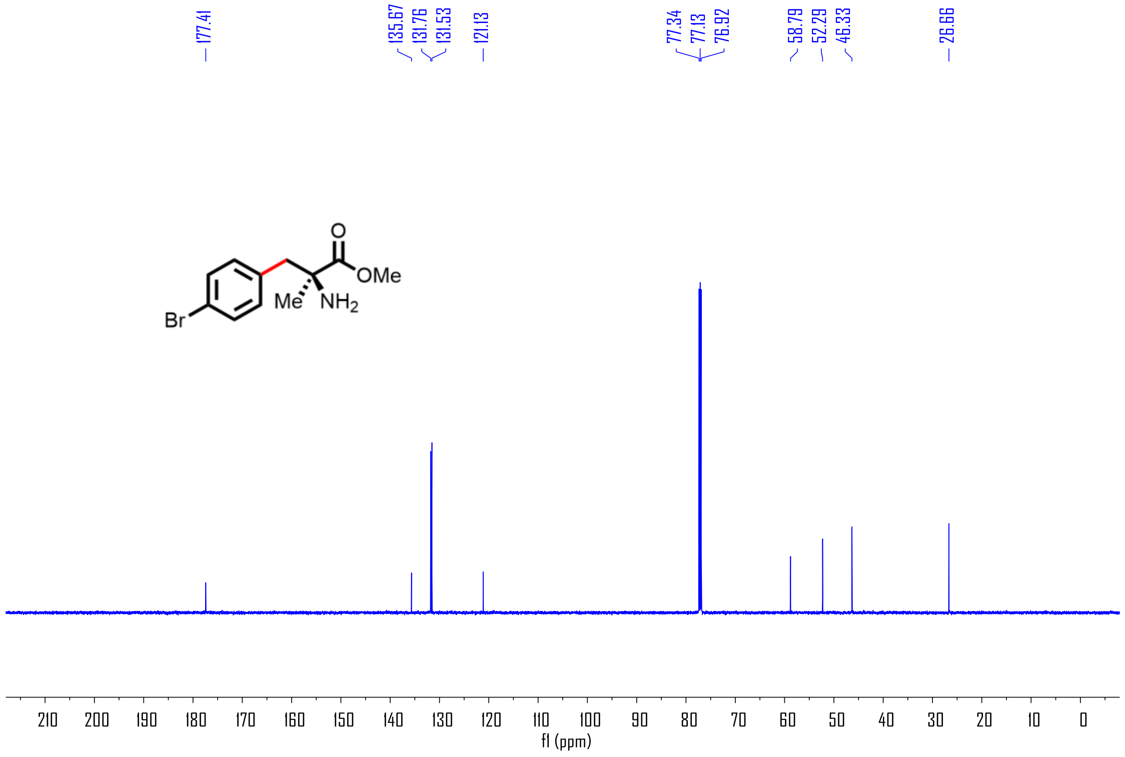
177.41
135.67
131.76
131.53
121.13
77.34
77.13
76.92
58.79
52.29
46.33
26.66
O
OMe
Br
Me
NH2
210 200 190 180 170 160 150 140 130 120 110 100 90 80 70 60 50 40 30 20 10 0
f1 (ppm)

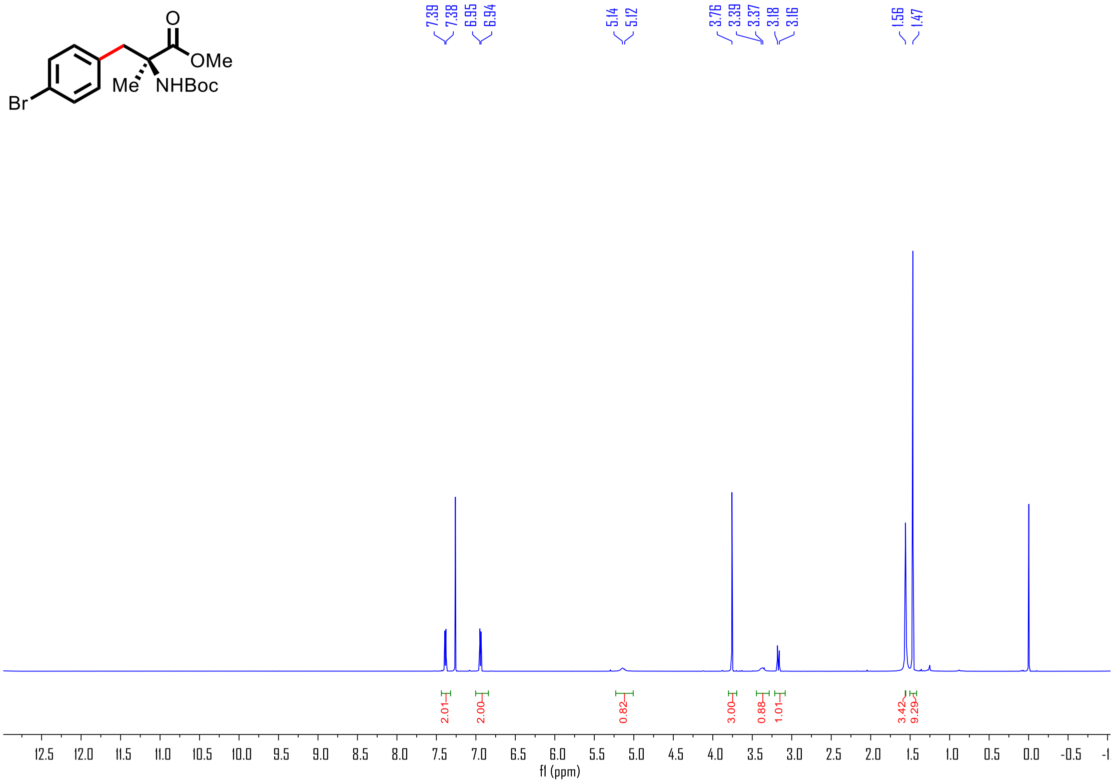
O
OMe
Me
NHBoc
Br
7.39
7.38
6.95
6.94
5.14
5.12
3.76
3.39
3.37
3.18
3.16
1.56
1.47
2.01
2.00
0.82
3.00
0.88
1.01
3.42
9.29
12.5 12.0 11.5 11.0 10.5 10.0 9.5 9.0 8.5 8.0 7.5 7.0 6.5 6.0 5.5 5.0 4.5 4.0 3.5 3.0 2.5 2.0 1.5 1.0 0.5 0.0 -0.5
f1 (ppm)

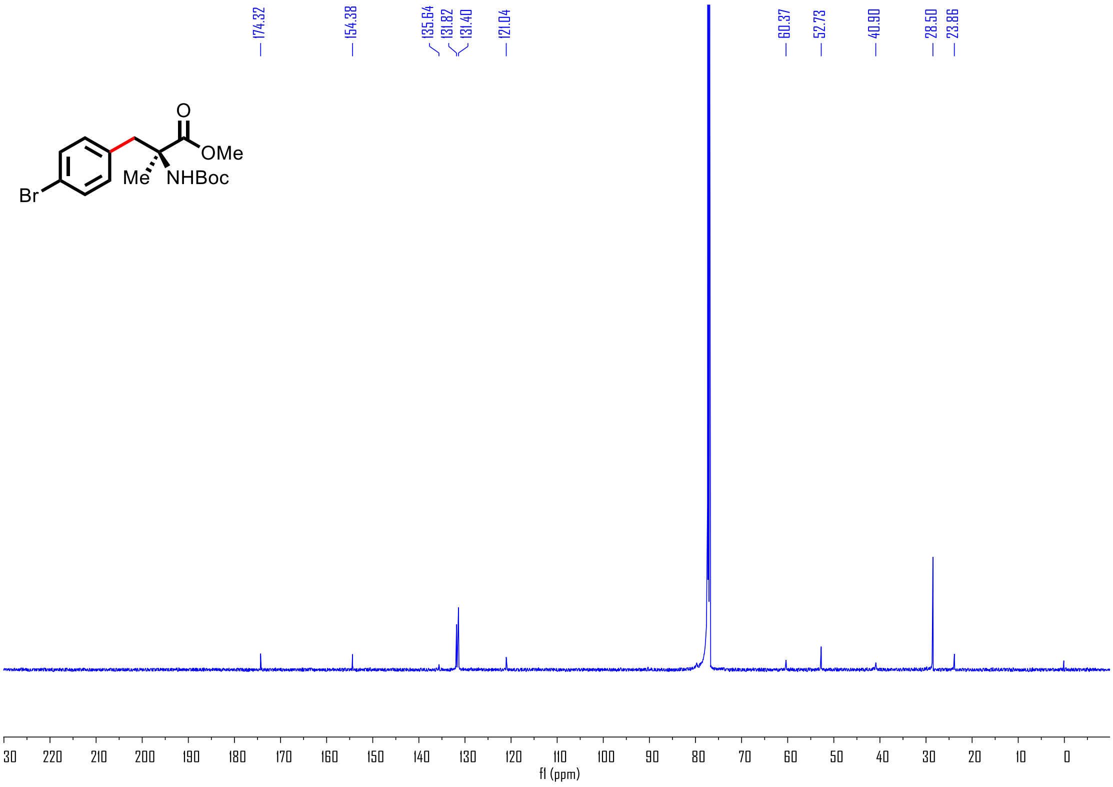
174.32
154.38
135.64
131.82
131.40
121.04
60.37
52.73
40.90
28.50
23.86
O
OMe
Me
NHBoc
Br
220 210 200 190 180 170 160 150 140 130 120 110 100 90 80 70 60 50 40 30 20 10 0
f1 (ppm)
